# Checklist of the marine malacofauna of Culuccia Peninsula (NW Sardinia, Italy), with notes on relevant species

**DOI:** 10.3897/BDJ.12.e115051

**Published:** 2024-02-28

**Authors:** Paolo Mariottini, Carlo Smriglio, Marco Oliverio, Sabrina Rossi, Andrea Di Giulio

**Affiliations:** 1 Department of Science, Roma Tre University, Rome, Italy Department of Science, Roma Tre University Rome Italy; 2 Dept. of Biology & Biotechnologies 'Charles Darwin', Sapienza University of Rome, Rome, Italy Dept. of Biology & Biotechnologies 'Charles Darwin', Sapienza University of Rome Rome Italy; 3 Biru S.r.l. Agricola, S.Teresa di Gallura (SS), Italy Biru S.r.l. Agricola S.Teresa di Gallura (SS) Italy; 4 NBFC - National Biodiversity Future Center, Palermo, Italy NBFC - National Biodiversity Future Center Palermo Italy

**Keywords:** Catalogue, Mollusca, Bivalvia, Gastropoda, Polyplacophora, Scaphopoda

## Abstract

**Background:**

Culuccia is a small peninsula of about 3 km^2^ placed in north-western Sardinia (Italy) at the margin of the Maddalena Archipelago. The marine area surrounding this Peninsula is a Special Area of Conservation, included in the European Natura 2000 Ecological Network of protected areas, but until now, no information on biodiversity of this area is available. In 2021, a research project to study both terrestrial and marine biodiversity of Culuccia has started in order to fill this gap of knowledge.

**New information:**

This work provides the first inventory of the marine malacofauna of the coast of Culuccia. Fifteen sites were sampled seasonally for one-year by using different sampling methods and the present study shows the results from approximately 50 scientific SCUBA and free dive surveys, carried out in all main marine habitats of the studied area. In total, 259 species of molluscs were recorded along the coasts of the Culuccia Peninsula (0-25 m depth), belonging to the classes Bivalvia, Gastropoda, Polyplacophora and Scaphopoda. Amongst the four classes recorded, gastropods were the most represented (66.90%; 173 species), followed by bivalves (28.10%; 73 species), polyplacophorans (4.60%; 12 species) and scapophods (0.40%; 1 species). Notes about distribution, conservation status and ecology for some valuable species are provided, together with images of representative species, consisting mainly of *in situ* photographs. Additionally, the present investigation recorded the presence of four alien species, whose Mediterranean distribution was extended to north-western Sardinia.

## Introduction

**Figure 1.**
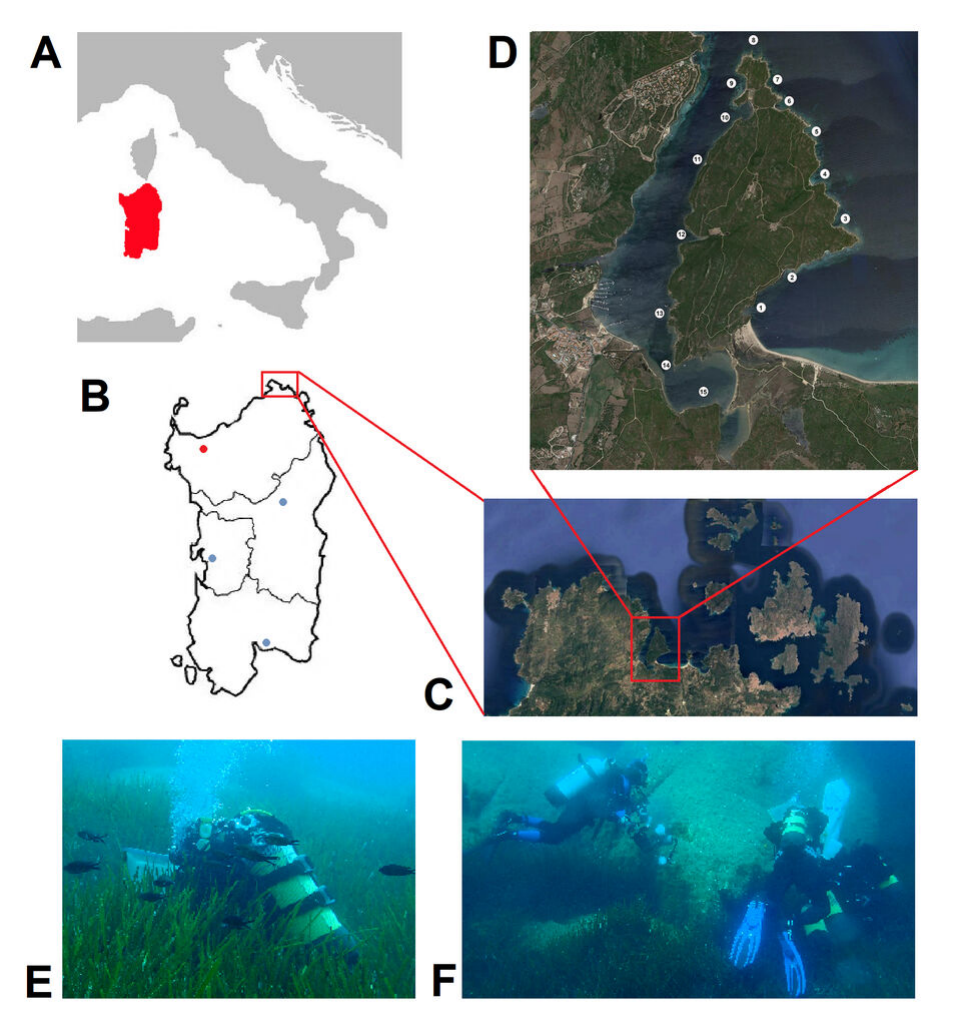
Geographic map of the Culuccia Peninsula and localisation of the sampling sites with illustration of field collecting methods. **A-D** Different geographic scales indicating the position of the Culuccia Peninsula and distribution of sampling sites; **E-F** Scuba divers sampling on *Posidoniaoceanica* meadow and rocky substrate.

Baseline studies of the marine molluscan fauna are essential steps to describe the biodiversity occurring along the Mediterranean coasts and to monitor the possible presence of alien species, especially nowadays when the marine biodiversity alteration in the Mediterranean Sea is a major concern. This marine basin, one of the world’s most biodiverse, faces major threats, such as overexploitation of resources, pollution and the current climate change. Albeit the Mediterranean Sea is characterised by a high degree of endemism and specific communities, it is also the sea receiving the highest number of alien species (Galil et al. 2018). In the Mediterranean Sea, molluscan communities play a crucial role in the dynamics of the benthic ecosystems and are amongst the dominant and most important components of the benthic macrofauna (Barnes 1980, Guelorget and Perthuisot 1992). Culuccia is a small peninsula of about 3 km^2^ placed in north-western Sardinia (Italy) at the margin of the Maddalena Archipelago, between Santa Teresa di Gallura and Palau (Fig. 1). The marine area surrounding this Peninsula is a Special Area of Conservation (SAC), included in the European Natura 2000 Ecological Network of protected areas, but so far, no information on the biodiversity of this area is available. In 2021, a research project to study both terrestrial and marine biodiversity of Culuccia has started in order to fill this gap of knowledge. This study presents the checklist of the molluscs observed in their habitats from the intertidal to the shallow sublittoral waters surrounding the coastline of the Peninsula. The lower depth coverage was limited to 25 m. The research was carried out by SCUBA diving and free-diving survey over 1 year (from July 2022 to July 2023), at 15 sampling sites (SS). The molluscan classes taken into account were Polyplacophora, Gastropoda, Scaphopoda and Bivalvia. For some species, *in situ* photographs are provided together with relative notes. Furthermore, we also report the records of four Non-Indigenous Species (NIS) from in this marine spot, which widen their Mediterranean distributions to north-western Sardinia.

## Materials and methods

### Study area

The Culuccia Peninsula is located in north-western Sardinia, within the Santa Teresa di Gallura territory, facing the Conca Verde at the west and the Isola dei Gabbiani at the east (Fig. 1A-C). It is characterised mainly by rocky coasts and a few sandy beaches; relatively quiet weather conditions are met during the Summer, whereas strong currents and high-energy waves feature the Winter season. The western and eastern coasts of Culuccia are different. The western coast faces a narrow fjord, which hosts a small touristic harbour (Porto Pozzo, with a small Marina), minor stream mouths and a suspended aquaculture farm rearing the oyster *Magallanagigas* (Thunberg, 1793). The southernmost part of this fjiord encloses a small shallow water brackish lagoon, which is connected to internal salt marshes. The north-eastern side faces the open sea; the south-eastern coast forms, together with the Isola dei Gabbiani, a small bay where the Liscia River flows. Due to the different marine conditions and habitats of the shores around the Peninsula, 15 sampling sites (SS) regularly spaced were selected (Fig. 1). Habitat descriptions, geographic coordinates and depth range of the SS are provided in Table 1.

### Field sampling

Specimens were collected either by direct hand or using an air-lift suction sampler during diving (SCUBA and free diving, depending on the depth) in benthic environments, on both rocky and soft substrates, particularly on/under rocks, rubble, amidst algae and in *Posidoniaoceanica* L. (Delile) meadows. A total of 50 SCUBA dives (1 hour each) were performed by five divers down to 25 m depth, during about one-year monitoring (2022-2023). An ethical approach in this research was carried out by complying with the restrictions in term of collected specimen size, environmental survey of the collection sites, as well as complying with local, regional, national and international rules and regulations for access to biodiversity, sustainable use and benefit sharing (Convention on Biological Diversity and its Nagoya Protocol, national regulations). Species identification was initially made by each collector, but all specimens were then cross-checked by all authors who are experienced malacological taxonomists. Live animals were photographed *in situ* or in the Coluccia’s laboratory (Osservatorio Naturalistico) with the digital cameras Canon Powershot G11 and Olympus TG-6, equipped with housing for marine photographs. Specimens of particular interest were anaesthetised with 7.5% magnesium chloride (MgCl_2_) and preserved in 95% ethanol as reference material and for future molecular studies. All sampled material was deposited in the Malacology Collection of the Department of Biology and Biotechnologies Charles Darwin, Sapienza University of Rome (Rome, Italy).

### Morphological analyses

Sorting and identifications of the material was performed by using stereomicroscopes. Representative specimens were photographed by using a ZEISS Axio Zoom.V16 steremicroscope, equipped with a ZEISS AxioCam 503 camera. For Scanning Electron Microscopy (SEM) analyses, dried shells were mounted on aluminium stubs using self-adhesive carbon discs, sputtered with gold (Emitech k550 Sputter Coater) and analysed by a Zeiss Gemini 300 SEM at L.I.M.E. (Laboratorio Interdipartimentale di Microscopia Elettronica, Roma Tre University, Rome, Italy) laboratory.

## Checklists

### Class Polyplacophora

#### Callochitonida

8D936223-B241-5F80-AB1E-BE872F3B785B

#### Callochitonidae

91EFDEE2-1EFD-5AFD-A2AB-F230E375BEB3

#### Callochiton septemvalvis

(Montagu, 1803)

F5440B5E-AB62-59CD-AD1D-EF02C16EBA9F

##### Materials

- **Type status:** Other material. **Occurrence:** occurrenceID: E24EB0B4-A1D4-5584-A897-5A55F3234FED; **Location:** country: Italy; countryCode: IT; stateProvince: Sassari; locality: Island of Culuccia; verbatimLatitude: 41 13 17.70N; verbatimLongitude: 9 17 21.41E; geodeticDatum: WGS84

##### Notes

**Figure 2.**
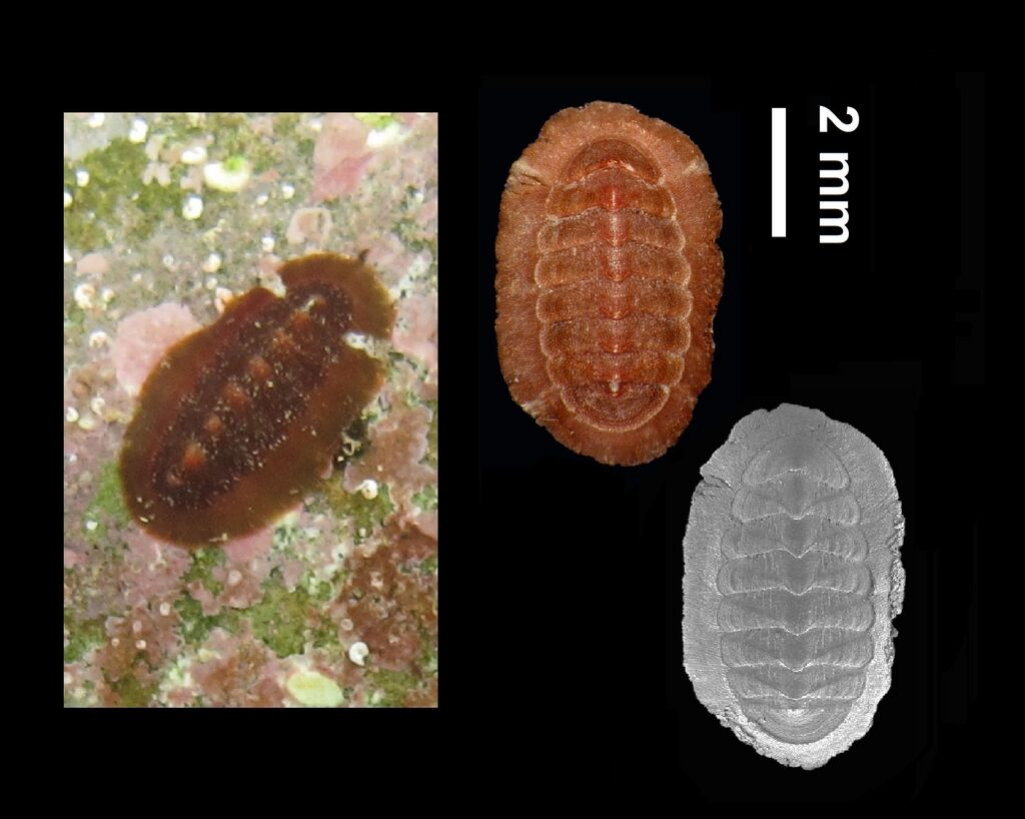
*Callochitonseptemvalvis* (Montagu, 1803). On the left, *in situ* photograph of specimen A; on the right, optical and SEM photographs of specimen B.

Alive, Fig. 2; new record.

#### Chitonida

7C94294F-9631-5C74-B85E-B30A100CDE4F

#### Acanthochitonidae

44EA7516-838C-5999-88D8-6E25CE0A8224

#### Acantochitona crinita

(Pennant, 1777)

1977E2AD-5DC2-576B-B584-267C01F3FB58

##### Materials

- **Type status:** Other material. **Occurrence:** occurrenceID: 2B675D4D-88D7-57E6-8673-F00D43749A0D; **Location:** country: Italy; countryCode: IT; stateProvince: Sassari; locality: Island of Culuccia; verbatimLatitude: 41 13 17.70N; verbatimLongitude: 9 17 21.41E; geodeticDatum: WGS84

##### Notes

Shell.

#### Acantochitona fascicularis

(Linnaeus, 1767)

707A66DD-D902-5BB8-9B79-E44BEDA73927

##### Materials

- **Type status:** Other material. **Occurrence:** occurrenceID: 3645FE7E-0B64-5BEB-9D30-E20FE6C7D812; **Location:** country: Italy; countryCode: IT; stateProvince: Sassari; locality: Island of Culuccia; verbatimLatitude: 41 11 46.79N; verbatimLongitude: 9 17 20.34E; geodeticDatum: WGS84
- **Type status:** Other material. **Occurrence:** occurrenceID: 6E230691-029A-5558-ADE0-D415D931EAF2; **Location:** country: Italy; countryCode: IT; stateProvince: Sassari; locality: Island of Culuccia; verbatimLatitude: 41 11 56.04N; verbatimLongitude: 9 17 33.63E; geodeticDatum: WGS84
- **Type status:** Other material. **Occurrence:** occurrenceID: 62159682-4B11-5EF7-9B71-060E5B457A7F; **Location:** country: Italy; countryCode: IT; stateProvince: Sassari; locality: Island of Culuccia; verbatimLatitude: 41 12 28.07N; verbatimLongitude: 9 17 47.62E; geodeticDatum: WGS84
- **Type status:** Other material. **Occurrence:** occurrenceID: 3C9201F6-6D66-5ECD-85B9-1382F0A819D7; **Location:** country: Italy; countryCode: IT; stateProvince: Sassari; locality: Island of Culuccia; verbatimLatitude: 41 12 42.40N; verbatimLongitude: 9 17 45.35E; geodeticDatum: WGS84

##### Notes

Alive, Fig. 3.

#### Chitonidae

D15B3754-0BA3-5C8F-A7E6-444B9CDD7C85

#### Rhyssoplax olivacea

Spengler, 1798

B98932BD-E766-5D4C-9308-E32D21DB6768

##### Materials

- **Type status:** Other material. **Occurrence:** occurrenceID: 12EFA212-6483-5315-9EA4-D6D43B1423E9; **Location:** country: Italy; countryCode: IT; stateProvince: Sassari; locality: Island of Culuccia; verbatimLatitude: 41 11 56.04N; verbatimLongitude: 9 17 33.63E; geodeticDatum: WGS84
- **Type status:** Other material. **Occurrence:** occurrenceID: FF2D7939-7798-5218-8B86-A7CE2E12AC32; **Location:** country: Italy; countryCode: IT; stateProvince: Sassari; locality: Island of Culuccia; verbatimLatitude: 41 12 28.07N; verbatimLongitude: 9 17 47.62E; geodeticDatum: WGS84
- **Type status:** Other material. **Occurrence:** occurrenceID: 2D214B34-D5B7-549C-B433-91F9BAA41750; **Location:** country: Italy; countryCode: IT; stateProvince: Sassari; locality: Island of Culuccia; verbatimLatitude: 41 12 42.40N; verbatimLongitude: 9 17 45.35E; geodeticDatum: WGS84
- **Type status:** Other material. **Occurrence:** occurrenceID: 1CF96E6D-BF23-5813-BE90-7293DFE29CD4; **Location:** country: Italy; countryCode: IT; stateProvince: Sassari; locality: Island of Culuccia; verbatimLatitude: 41 12 58.16N; verbatimLongitude: 9 17 09.66E; geodeticDatum: WGS84

##### Notes

Alive, Fig. 4.

#### Ischnochitonidae

FDC7A8A3-FA19-586C-AB7A-1B297A742F09

#### Ischnochiton rissoi

(Payraudeau, 1826)

4E7A7223-615D-5964-A55A-4374C6DDAF9D

##### Materials

- **Type status:** Other material. **Occurrence:** occurrenceID: E7E2F4ED-8A95-5795-A9B6-225F9069D1B1; **Location:** country: Italy; countryCode: IT; stateProvince: Sassari; locality: Island of Culuccia; verbatimLatitude: 41 11 46.79N; verbatimLongitude: 9 17 20.34E; geodeticDatum: WGS84
- **Type status:** Other material. **Occurrence:** occurrenceID: A631A64A-4343-557D-91D1-DF926295387B; **Location:** country: Italy; countryCode: IT; stateProvince: Sassari; locality: Island of Culuccia; verbatimLatitude: 41 11 56.04N; verbatimLongitude: 9 17 33.63E; geodeticDatum: WGS84

##### Notes

Alive, Fig. 5.

#### Tonicellidae

19600E27-3821-53B6-93F0-C2902AA5723E

#### Lepidochitona cinerea

(Linnaeus, 1767)

735E5688-ABD8-5688-AAC4-59205DF47CB2

##### Materials

- **Type status:** Other material. **Occurrence:** occurrenceID: 081E996A-451B-5861-B3E8-10448CBD689B; **Location:** country: Italy; countryCode: IT; stateProvince: Sassari; locality: Island of Culuccia; verbatimLatitude: 41 11 46.79N; verbatimLongitude: 9 17 20.34E; geodeticDatum: WGS84

##### Notes

Alive, Fig. 6.

#### Lepidochitona caprearum

(Scacchi, 1836)

85B5EABD-49E1-5ABF-A62E-793A7D2301F6

##### Materials

- **Type status:** Other material. **Occurrence:** occurrenceID: B8EE8D4F-E71E-56D0-9AB2-5E4E06141F9B; **Location:** country: Italy; countryCode: IT; stateProvince: Sassari; locality: Island of Culuccia; verbatimLatitude: 41 13 17.70N; verbatimLongitude: 9 17 21.41E; geodeticDatum: WGS84

##### Notes

Shell.

#### Lepidopleurus cajetanus

(Poli, 1791)

CA0FF235-62D6-54CF-A6B4-79964A66BE1A

##### Materials

- **Type status:** Other material. **Occurrence:** occurrenceID: FF837056-CFE8-57C0-9B37-9A979F28B6EF; **Location:** country: Italy; countryCode: IT; stateProvince: Sassari; locality: Island of Culuccia; verbatimLatitude: 41 11 46.79N; verbatimLongitude: 9 17 20.34E; geodeticDatum: WGS84
- **Type status:** Other material. **Occurrence:** occurrenceID: 88A5990B-51DA-5B82-B571-26EF82390AF3; **Location:** country: Italy; countryCode: IT; stateProvince: Sassari; locality: Island of Culuccia; verbatimLatitude: 41 11 56.04N; verbatimLongitude: 9 17 33.63E; geodeticDatum: WGS84
- **Type status:** Other material. **Occurrence:** occurrenceID: 83D0E679-75C5-5AC0-9E23-6D6C91D47F62; **Location:** country: Italy; countryCode: IT; stateProvince: Sassari; locality: Island of Culuccia; verbatimLatitude: 41 12 28.07N; verbatimLongitude: 9 17 47.62E; geodeticDatum: WGS84
- **Type status:** Other material. **Occurrence:** occurrenceID: 39147A89-3A3B-5EA5-870E-5993D9705005; **Location:** country: Italy; countryCode: IT; stateProvince: Sassari; locality: Island of Culuccia; verbatimLatitude: 41 12 42.40N; verbatimLongitude: 9 17 45.35E; geodeticDatum: WGS84

##### Notes

Alive, Fig. 7.

#### Leptochiton algesirensis

(Capellini, 1859)

C8311C50-111D-529B-96E2-089F70CA09BF

##### Materials

- **Type status:** Other material. **Occurrence:** occurrenceID: 958F57ED-EE8B-599C-9932-5E912FFF06E9; **Location:** country: Italy; countryCode: IT; stateProvince: Sassari; locality: Island of Culuccia; verbatimLatitude: 41 12 42.40N; verbatimLongitude: 9 17 45.35E; geodeticDatum: WGS84

##### Notes

Alive, Fig. 8.

#### Leptochiton asellus

(Gmelin, 1791)

F56DE440-585B-5F60-9401-C135D94408AA

##### Materials

- **Type status:** Other material. **Occurrence:** occurrenceID: 4CD88992-ABCB-53AE-8DFA-B614B8CE719B; **Location:** country: Italy; countryCode: IT; stateProvince: Sassari; locality: Island of Culuccia; verbatimLatitude: 41 13 17.70N; verbatimLongitude: 9 17 21.41E; geodeticDatum: WGS84

##### Notes

Shell; new record.

#### Leptochiton cancellatus

(Sowerby, 1840)

AAF65E32-79E2-501E-90D2-E3C5BE955921

##### Materials

- **Type status:** Other material. **Occurrence:** occurrenceID: A123168F-DD22-5021-98B9-F4FA43BE355C; **Location:** country: Italy; countryCode: IT; stateProvince: Sassari; locality: Island of Culuccia; verbatimLatitude: 41 13 17.70N; verbatimLongitude: 9 17 21.41E; geodeticDatum: WGS84

##### Notes

Shell.

#### Leptochiton scabridus

(Jeffreys, 1880)

CB5EF47D-FD41-5CAC-B276-834CBDFF0C80

##### Materials

- **Type status:** Other material. **Occurrence:** occurrenceID: 07B29370-EF6A-5F39-AF4B-740E7AE489E9; **Location:** country: Italy; countryCode: IT; stateProvince: Sassari; locality: Island of Culuccia; verbatimLatitude: 41 13 17.70N; verbatimLongitude: 9 17 21.41E; geodeticDatum: WGS84

##### Notes

Shell.

### Class Gastropoda

#### Patellagastropoda

F19A157E-4EA7-560D-B6AA-B52DAC95F311

#### Patellidae

5FB41064-053D-5BD7-9181-7973F68371BB

#### Patella caerulea

Linnaeus, 1758

F5A82905-A378-528D-80A1-B8E90B0E2DF0

##### Materials

- **Type status:** Other material. **Occurrence:** occurrenceID: DCDDCA70-2EC5-5386-8885-7AEFD6FAB598; **Location:** country: Italy; countryCode: IT; stateProvince: Sassari; locality: Island of Culuccia; verbatimLatitude: 41 11 46.79N; verbatimLongitude: 9 17 20.34E; geodeticDatum: WGS84
- **Type status:** Other material. **Occurrence:** occurrenceID: 77DE7AF3-3FD3-56A9-9AC1-6DB09CB9F254; **Location:** country: Italy; countryCode: IT; stateProvince: Sassari; locality: Island of Culuccia; verbatimLatitude: 41 12 51.58N; verbatimLongitude: 9 17 31.46E; geodeticDatum: WGS84
- **Type status:** Other material. **Occurrence:** occurrenceID: A3793666-7794-5FF2-90CE-478ECD363BCE; **Location:** country: Italy; countryCode: IT; stateProvince: Sassari; locality: Island of Culuccia; verbatimLatitude: 41 12 13.88N; verbatimLongitude: 9 17 56.14E; geodeticDatum: WGS84
- **Type status:** Other material. **Occurrence:** occurrenceID: 74E1CD41-7AD3-528F-A41B-EBE1AC89FA49; **Location:** country: Italy; countryCode: IT; stateProvince: Sassari; locality: Island of Culuccia; verbatimLatitude: 41 12 28.07N; verbatimLongitude: 9 17 47.62E; geodeticDatum: WGS84
- **Type status:** Other material. **Occurrence:** occurrenceID: F23EF0B5-6888-59C6-B05E-4B9BD485BE61; **Location:** country: Italy; countryCode: IT; stateProvince: Sassari; locality: Island of Culuccia; verbatimLatitude: 41 12 51.58N; verbatimLongitude: 9 17 31.46E; geodeticDatum: WGS84
- **Type status:** Other material. **Occurrence:** occurrenceID: CB36A960-13D2-5F9C-BA2D-34B71E4A15B2; **Location:** country: Italy; countryCode: IT; stateProvince: Sassari; locality: Island of Culuccia; verbatimLatitude: 41 12 58.68N; verbatimLongitude: 9 17 26.51E; geodeticDatum: WGS84
- **Type status:** Other material. **Occurrence:** occurrenceID: E94F9EB8-99AD-5F79-AD3C-722027184953; **Location:** country: Italy; countryCode: IT; stateProvince: Sassari; locality: Island of Culuccia; verbatimLatitude: 41 12 58.16N; verbatimLongitude: 9 17 09.66E; geodeticDatum: WGS84

##### Notes

Alive, Fig. 9.

#### Patella ferruginea

Gmelin, 1791

1CE8EBAD-4453-5E0D-9692-FBD95338FFFA

##### Materials

- **Type status:** Other material. **Occurrence:** occurrenceID: 6FF8E588-4680-5E71-8317-B0C1570F01E1; **Location:** country: Italy; countryCode: IT; stateProvince: Sassari; locality: Island of Culuccia; verbatimLatitude: 41 12 28.07N; verbatimLongitude: 9 17 47.62E; geodeticDatum: WGS84
- **Type status:** Other material. **Occurrence:** occurrenceID: 1ECFA020-9E1D-515D-ADB9-1B763AF7B90D; **Location:** country: Italy; countryCode: IT; stateProvince: Sassari; locality: Island of Culuccia; verbatimLatitude: 41 12 51.58N; verbatimLongitude: 9 17 31.46E; geodeticDatum: WGS84
- **Type status:** Other material. **Occurrence:** occurrenceID: EF11FC30-9FD4-5A85-938C-DB9BD94AC97A; **Location:** country: Italy; countryCode: IT; stateProvince: Sassari; locality: Island of Culuccia; verbatimLatitude: 41 13 17.70N; verbatimLongitude: 9 17 21.41E; geodeticDatum: WGS84

##### Notes

Alive, Fig. 10.

#### Patella rustica

Linnaeus, 1758

838222B1-20DC-578A-BEB9-4C095D4854EF

##### Materials

- **Type status:** Other material. **Occurrence:** occurrenceID: 4CCC358A-B1DD-53BC-90CE-D649EBC71E49; **Location:** country: Italy; countryCode: IT; stateProvince: Sassari; locality: Island of Culuccia; verbatimLatitude: 41 11 46.79N; verbatimLongitude: 9 17 20.34E; geodeticDatum: WGS84
- **Type status:** Other material. **Occurrence:** occurrenceID: 13077043-2A0C-5DED-81B2-F65F0EBFD61E; **Location:** country: Italy; countryCode: IT; stateProvince: Sassari; locality: Island of Culuccia; verbatimLatitude: 41 12 51.58N; verbatimLongitude: 9 17 31.46E; geodeticDatum: WGS84
- **Type status:** Other material. **Occurrence:** occurrenceID: A24FA4FA-8315-55A4-BCDE-6492B384604E; **Location:** country: Italy; countryCode: IT; stateProvince: Sassari; locality: Island of Culuccia; verbatimLatitude: 41 12 13.88N; verbatimLongitude: 9 17 56.14E; geodeticDatum: WGS84
- **Type status:** Other material. **Occurrence:** occurrenceID: 892D82A6-79F9-561E-B8AF-B934F6270F37; **Location:** country: Italy; countryCode: IT; stateProvince: Sassari; locality: Island of Culuccia; verbatimLatitude: 41 12 28.07N; verbatimLongitude: 9 17 47.62E; geodeticDatum: WGS84
- **Type status:** Other material. **Occurrence:** occurrenceID: F30CCC81-2F52-5A3F-8EE2-FE21AC9285F9; **Location:** country: Italy; countryCode: IT; stateProvince: Sassari; locality: Island of Culuccia; verbatimLatitude: 41 12 51.58N; verbatimLongitude: 9 17 31.46E; geodeticDatum: WGS84
- **Type status:** Other material. **Occurrence:** occurrenceID: 8EF2B546-2B57-58E8-819C-71543D0B686E; **Location:** country: Italy; countryCode: IT; stateProvince: Sassari; locality: Island of Culuccia; verbatimLatitude: 41 12 58.68N; verbatimLongitude: 9 17 26.51E; geodeticDatum: WGS84
- **Type status:** Other material. **Occurrence:** occurrenceID: 79128D5F-D7D5-5493-A697-D41C7A8263AC; **Location:** country: Italy; countryCode: IT; stateProvince: Sassari; locality: Island of Culuccia; verbatimLatitude: 41 12 58.16N; verbatimLongitude: 9 17 09.66E; geodeticDatum: WGS84

##### Notes

Alive, Fig. 11.

#### Patella ulyssiponensis

Gmelin, 1791

A9F3DABF-C6B0-5006-81B5-BB3FC2C565C0

##### Materials

- **Type status:** Other material. **Occurrence:** occurrenceID: 273CC16C-ECA9-516E-9DF8-E4B5A2BAF1C9; **Location:** country: Italy; countryCode: IT; stateProvince: Sassari; locality: Island of Culuccia; verbatimLatitude: 41 11 46.79N; verbatimLongitude: 9 17 20.34E; geodeticDatum: WGS84
- **Type status:** Other material. **Occurrence:** occurrenceID: 0B9BC4BB-8A02-52DA-8E23-29D1BECA3690; **Location:** country: Italy; countryCode: IT; stateProvince: Sassari; locality: Island of Culuccia; verbatimLatitude: 41 12 51.58N; verbatimLongitude: 9 17 31.46E; geodeticDatum: WGS84
- **Type status:** Other material. **Occurrence:** occurrenceID: 541A7598-7150-58E4-A575-2A94DD3F97A1; **Location:** country: Italy; countryCode: IT; stateProvince: Sassari; locality: Island of Culuccia; verbatimLatitude: 41 12 13.88N; verbatimLongitude: 9 17 56.14E; geodeticDatum: WGS84
- **Type status:** Other material. **Occurrence:** occurrenceID: F0C1C0F9-99A6-57C0-A8AE-7705BE804BFA; **Location:** country: Italy; countryCode: IT; stateProvince: Sassari; locality: Island of Culuccia; verbatimLatitude: 41 12 28.07N; verbatimLongitude: 9 17 47.62E; geodeticDatum: WGS84
- **Type status:** Other material. **Occurrence:** occurrenceID: 61BBDD56-C49D-5936-804C-6648AF9AC63F; **Location:** country: Italy; countryCode: IT; stateProvince: Sassari; locality: Island of Culuccia; verbatimLatitude: 41 12 51.58N; verbatimLongitude: 9 17 31.46E; geodeticDatum: WGS84
- **Type status:** Other material. **Occurrence:** occurrenceID: A8434292-583D-5CB3-9908-B9C3BC8E9C15; **Location:** country: Italy; countryCode: IT; stateProvince: Sassari; locality: Island of Culuccia; verbatimLatitude: 41 12 58.68N; verbatimLongitude: 9 17 26.51E; geodeticDatum: WGS84
- **Type status:** Other material. **Occurrence:** occurrenceID: D6BB67E2-0B63-5D20-882F-ECC50050EB32; **Location:** country: Italy; countryCode: IT; stateProvince: Sassari; locality: Island of Culuccia; verbatimLatitude: 41 12 58.16N; verbatimLongitude: 9 17 09.66E; geodeticDatum: WGS84

##### Notes

Alive, Fig. 12.

#### Lottiidae

FA920F84-669C-5AA4-B949-45DB25A6AE75

#### Tectura virginea

(Mueller O.F., 1776)

07D6E90E-E4EB-542C-9436-5C069EDD8F76

##### Materials

- **Type status:** Other material. **Occurrence:** occurrenceID: B691C5C2-8796-5770-B100-B8CABEEAFFD5; **Location:** country: Italy; countryCode: IT; stateProvince: Sassari; locality: Island of Culuccia; verbatimLatitude: 41 13 17.70N; verbatimLongitude: 9 17 21.41E; geodeticDatum: WGS84

##### Notes

Shell.

#### Lepetellida

683B060C-67A7-5A54-A9D1-58FBC1D28E07

#### Scissurellidae

4C1DF25B-DB86-5E38-86C5-C4F01E44E4EC

#### Scissurella costata

d'Orbigny, 1824

681198DC-9BF7-5C31-A2F9-08A49E825BB3

##### Materials

- **Type status:** Other material. **Occurrence:** occurrenceID: 28423D17-A2C2-5959-B4E6-E00C62CA603F; **Location:** country: Italy; countryCode: IT; stateProvince: Sassari; locality: Island of Culuccia; verbatimLatitude: 41 13 17.70N; verbatimLongitude: 9 17 21.41E; geodeticDatum: WGS84

##### Notes

Shell.

#### Haliotidae

EB2E6E5B-C5DA-5EC1-837A-3DAAB8E6A189

#### Haliotis tuberculata

Linnaeus, 1758

07BBB9AF-4478-5B09-B8C6-F2052B0EF2EE

##### Materials

- **Type status:** Other material. **Occurrence:** occurrenceID: 48D02B05-9C2B-5409-95BC-612437AABD82; **Location:** country: Italy; countryCode: IT; stateProvince: Sassari; locality: Island of Culuccia; verbatimLatitude: 41 12 51.58N; verbatimLongitude: 9 17 31.46E; geodeticDatum: WGS84
- **Type status:** Other material. **Occurrence:** occurrenceID: 89801DAB-3F87-5F0E-B6C4-4BA0712B517A; **Location:** country: Italy; countryCode: IT; stateProvince: Sassari; locality: Island of Culuccia; verbatimLatitude: 41 12 28.07N; verbatimLongitude: 9 17 47.62E; geodeticDatum: WGS84
- **Type status:** Other material. **Occurrence:** occurrenceID: 3CDA66B2-C3BE-5484-9333-2CC3787B1FDE; **Location:** country: Italy; countryCode: IT; stateProvince: Sassari; locality: Island of Culuccia; verbatimLatitude: 41 12 47.63N; verbatimLongitude: 9 17 08.68E; geodeticDatum: WGS84

##### Notes

Alive, Fig. 13, Suppl. material 1.

#### Fissurellidae

EEDA4007-9928-531D-AB55-79768CFBE2C6

#### Emarginula adriatica

OG Costa, 1830

39B93464-0B0A-5CDC-8535-31DE0EA54A38

##### Materials

- **Type status:** Other material. **Occurrence:** occurrenceID: 40AF59ED-C9E8-5596-9A67-DD2E6F684E60; **Location:** country: Italy; countryCode: IT; stateProvince: Sassari; locality: Island of Culuccia; verbatimLatitude: 41 13 17.70N; verbatimLongitude: 9 17 21.41E; geodeticDatum: WGS84

##### Notes

Shell.

#### Emarginula huzardii

Payraudeau, 1826

D265BF3F-A0DC-5E27-AD19-9E90AADB3463

##### Materials

- **Type status:** Other material. **Occurrence:** occurrenceID: 7B0969F8-7CF6-5974-B523-BE03C6D11801; **Location:** country: Italy; countryCode: IT; stateProvince: Sassari; locality: Island of Culuccia; verbatimLatitude: 41 11 28.32N; verbatimLongitude: 9 16 44.50E; geodeticDatum: WGS84

##### Notes

Alive, Fig. 14.

#### Emarginula pustula

Thiele in Küster, 1913

DFCBE0FE-1057-5C7F-BDBB-6F246D24FFB8

##### Materials

- **Type status:** Other material. **Occurrence:** occurrenceID: E2AC86C9-86BB-5734-8C82-478B00CD5CAC; **Location:** country: Italy; countryCode: IT; stateProvince: Sassari; locality: Island of Culuccia; verbatimLatitude: 41 13 17.70N; verbatimLongitude: 9 17 21.41E; geodeticDatum: WGS84

##### Notes

Shell.

#### Emarginula sicula

JE Gray, 1825

C52C7CBE-3A5C-5AB3-8BA2-1C890E2AB079

##### Materials

- **Type status:** Other material. **Occurrence:** occurrenceID: 59E1F24F-A85D-5590-996F-46D7BC314C65; **Location:** country: Italy; countryCode: IT; stateProvince: Sassari; locality: Island of Culuccia; verbatimLatitude: 41 13 17.70N; verbatimLongitude: 9 17 21.41E; geodeticDatum: WGS84

##### Notes

Shell.

#### Diodora gibberula

(Lamarck, 1822)

2C7E7165-F41F-5DD9-8587-4EDA9F4B2131

##### Materials

- **Type status:** Other material. **Occurrence:** occurrenceID: C0A5E9E5-34FA-59F9-8418-338246B51682; **Location:** country: Italy; countryCode: IT; stateProvince: Sassari; locality: Island of Culuccia; verbatimLatitude: 41 13 17.70N; verbatimLongitude: 9 17 21.41E; geodeticDatum: WGS84

##### Notes

Shell.

#### Diodora graeca

(Linnaeus, 1758)

7183B797-2EF4-5BFA-9BB1-0D9FE5AEF447

##### Materials

- **Type status:** Other material. **Occurrence:** occurrenceID: A636B918-A321-57C8-8AD6-5920EFF8E812; **Location:** country: Italy; countryCode: IT; stateProvince: Sassari; locality: Island of Culuccia; verbatimLatitude: 41 11 46.79N; verbatimLongitude: 9 17 20.34E; geodeticDatum: WGS84

##### Notes

Ali ve, Fig. 15.

#### Trochida

BF0C910B-A830-5CB3-8E8C-DFBA9ACABB6D

#### Trochidae

7FF0807C-8596-5B62-905F-5AB2E5911850

#### Phorcus articulatus

(Lamarck, 1822)

E6F573DC-9FD6-5180-B8FE-08358754585D

##### Materials

- **Type status:** Other material. **Occurrence:** occurrenceID: CDA1CE24-9744-5701-A11B-E6A88AB96564; **Location:** country: Italy; countryCode: IT; stateProvince: Sassari; locality: Island of Culuccia; verbatimLatitude: 41 13 17.70N; verbatimLongitude: 9 17 21.41E; geodeticDatum: WGS84

##### Notes

Alive.

#### Phorcus turbinatus

(Born, 1778)

472E03B4-D38B-5AC2-A183-42CB9B23BA49

##### Materials

- **Type status:** Other material. **Occurrence:** occurrenceID: 1B256C8F-3A08-576D-8089-70E8B29CEDA3; **Location:** country: Italy; countryCode: IT; stateProvince: Sassari; locality: Island of Culuccia; verbatimLatitude: 41 12 13.88N; verbatimLongitude: 9 17 56.14E; geodeticDatum: WGS84
- **Type status:** Other material. **Occurrence:** occurrenceID: 498ECD2B-45EE-5B42-A98D-839317EC43CB; **Location:** country: Italy; countryCode: IT; stateProvince: Sassari; locality: Island of Culuccia; verbatimLatitude: 41 12 28.07N; verbatimLongitude: 9 17 47.62E; geodeticDatum: WGS84
- **Type status:** Other material. **Occurrence:** occurrenceID: C99EC1D7-AC10-5E37-A002-9232A30075A3; **Location:** country: Italy; countryCode: IT; stateProvince: Sassari; locality: Island of Culuccia; verbatimLatitude: 41 12 42.40N; verbatimLongitude: 9 17 45.35E; geodeticDatum: WGS84
- **Type status:** Other material. **Occurrence:** occurrenceID: B2C0FA2F-C03C-5B6A-9C8C-FB016F4B410C; **Location:** country: Italy; countryCode: IT; stateProvince: Sassari; locality: Island of Culuccia; verbatimLatitude: 41 12 58.16N; verbatimLongitude: 9 17 09.66E; geodeticDatum: WGS84

##### Notes

Alive, Fig. 16.

#### Gibbula ardens

(Salis Marschlins, 1793)

C02E1652-E0FE-54ED-A069-1F2FC14057BC

##### Materials

- **Type status:** Other material. **Occurrence:** occurrenceID: 6CD18459-CD5A-5174-9FFC-3D01B791083A; **Location:** country: Italy; countryCode: IT; stateProvince: Sassari; locality: Island of Culuccia; verbatimLatitude: 41 13 17.70N; verbatimLongitude: 9 17 21.41E; geodeticDatum: WGS84

##### Notes

Shell.

#### Gibbula fanulum

(Gmelin, 1791)

124ED034-0087-51AB-A2B5-250279879744

##### Materials

- **Type status:** Other material. **Occurrence:** occurrenceID: A8C4E7A7-6390-54BB-8753-A1F79A9B800D; **Location:** country: Italy; countryCode: IT; stateProvince: Sassari; locality: Island of Culuccia; verbatimLatitude: 41 13 17.70N; verbatimLongitude: 9 17 21.41E; geodeticDatum: WGS84

##### Notes

Alive, Fig. 17.

#### Gibbula guttadauri

(Philippi, 1836)

AC468413-013C-5940-B93E-16ABBA4E84AE

##### Materials

- **Type status:** Other material. **Occurrence:** occurrenceID: B2B83D7A-4152-5FD7-9BC0-2774BDA1B33E; **Location:** country: Italy; countryCode: IT; stateProvince: Sassari; locality: Island of Culuccia; verbatimLatitude: 41 13 17.70N; verbatimLongitude: 9 17 21.41E; geodeticDatum: WGS84

##### Notes

Shell.

#### Gibbula turbinoides

(Deshayes, 1835)

8584A040-54F2-5477-9ED3-22BB01AEB5A6

##### Materials

- **Type status:** Other material. **Occurrence:** occurrenceID: 113C7973-39A1-5E22-8617-8C91189BD140; **Location:** country: Italy; countryCode: IT; stateProvince: Sassari; locality: Island of Culuccia; verbatimLatitude: 41 13 17.70N; verbatimLongitude: 9 17 21.41E; geodeticDatum: WGS84

##### Notes

Shell.

#### Gibbula umbilicaris

(Linnaeus, 1758)

36DF93B1-FD98-5DE3-97BD-58AA7EAA5297

##### Materials

- **Type status:** Other material. **Occurrence:** occurrenceID: AF7C9E69-1623-5D06-A73C-DFA0B9FB17C5; **Location:** country: Italy; countryCode: IT; stateProvince: Sassari; locality: Island of Culuccia; verbatimLatitude: 41 12 13.88N; verbatimLongitude: 9 17 56.14E; geodeticDatum: WGS84
- **Type status:** Other material. **Occurrence:** occurrenceID: 67E1EAD0-7680-5F77-AD71-656B6E777714; **Location:** country: Italy; countryCode: IT; stateProvince: Sassari; locality: Island of Culuccia; verbatimLatitude: 41 12 28.07N; verbatimLongitude: 9 17 47.62E; geodeticDatum: WGS84
- **Type status:** Other material. **Occurrence:** occurrenceID: 6A68A9C5-CC3F-54BB-B43F-AF99527AA896; **Location:** country: Italy; countryCode: IT; stateProvince: Sassari; locality: Island of Culuccia; verbatimLatitude: 41 12 42.40N; verbatimLongitude: 9 17 45.35E; geodeticDatum: WGS84
- **Type status:** Other material. **Occurrence:** occurrenceID: 45C6942A-1089-57AC-84C0-64FBD8CE1B2B; **Location:** country: Italy; countryCode: IT; stateProvince: Sassari; locality: Island of Culuccia; verbatimLatitude: 41 13 17.70N; verbatimLongitude: 9 17 21.41E; geodeticDatum: WGS84

##### Notes

Alive, Fig. 18.

#### Jujubinus baudoni

(Monterosato, 1891)

F39BE805-05AE-5658-93D3-06C386380F95

##### Materials

- **Type status:** Other material. **Occurrence:** occurrenceID: DA6867DF-624B-5472-843E-74AA113753DC; **Location:** country: Italy; countryCode: IT; stateProvince: Sassari; locality: Island of Culuccia; verbatimLatitude: 41 13 17.70N; verbatimLongitude: 9 17 21.41E; geodeticDatum: WGS84

##### Notes

Shell.

#### Jujubinus depictus

(Deshayes, 1835)

488E3ABE-B3AA-5C9F-8981-2485348B10C5

##### Materials

- **Type status:** Other material. **Occurrence:** occurrenceID: 4563CE2A-E293-59DA-B2C1-B8CDC367CCF0; **Location:** country: Italy; countryCode: IT; stateProvince: Sassari; locality: Island of Culuccia; verbatimLatitude: 41 13 17.70N; verbatimLongitude: 9 17 21.41E; geodeticDatum: WGS84

##### Notes

Shell.

#### Jujubinus corallinus

(Monterosato, 1884)

954439AF-90D5-5174-88E3-5BBEE83D6021

##### Materials

- **Type status:** Other material. **Occurrence:** occurrenceID: ADCE7E2B-040B-5291-AC57-267B17590093; **Location:** country: Italy; countryCode: IT; stateProvince: Sassari; locality: Island of Culuccia; verbatimLatitude: 41 13 17.70N; verbatimLongitude: 9 17 21.41E; geodeticDatum: WGS84

##### Notes

Shell.

#### Jujubinus striatus

(Linnaeus, 1758)

3F821F0B-B28B-59A1-8E52-6B6B5D26D609

##### Materials

- **Type status:** Other material. **Occurrence:** occurrenceID: 1DDE695A-65E3-5E21-AB29-1ED7D7B3405C; **Location:** country: Italy; countryCode: IT; stateProvince: Sassari; locality: Island of Culuccia; verbatimLatitude: 41 13 17.70N; verbatimLongitude: 9 17 21.41E; geodeticDatum: WGS84

##### Notes

Shell.

#### Jujubinus exasperatus

(Pennant, 1777)

C54A3A95-17B9-52AC-8BEF-3AAC92296D3F

##### Materials

- **Type status:** Other material. **Occurrence:** occurrenceID: 6ABA169B-6BDE-56D2-BBBC-5FC1B977C5C4; **Location:** country: Italy; countryCode: IT; stateProvince: Sassari; locality: Island of Culuccia; verbatimLatitude: 41 13 17.70N; verbatimLongitude: 9 17 21.41E; geodeticDatum: WGS84

##### Notes

Alive, Fig. 19.

#### Clanculus corallinus

(Gmelin, 1791)

37BB1949-CD7E-5374-97FC-2860C0803AAA

##### Materials

- **Type status:** Other material. **Occurrence:** occurrenceID: 755CB4CD-55F9-5E57-AD4B-99BA30F61896; **Location:** country: Italy; countryCode: IT; stateProvince: Sassari; locality: Island of Culuccia; verbatimLatitude: 41 13 17.70N; verbatimLongitude: 9 17 21.41E; geodeticDatum: WGS84

##### Notes

Shell.

#### Clanculus cruciatus

(Linnaeus, 1758)

754FA446-A516-57BD-8CA4-AABBB0223CFB

##### Materials

- **Type status:** Other material. **Occurrence:** occurrenceID: A97C22A1-71CD-52D4-8BB1-34C1497D6986; **Location:** country: Italy; countryCode: IT; stateProvince: Sassari; locality: Island of Culuccia; verbatimLatitude: 41 12 28.07N; verbatimLongitude: 9 17 47.62E; geodeticDatum: WGS84

##### Notes

Alive, Fig. 20.

#### Clanculus jussieui

(Payraudeau, 1826)

6A041973-93B4-505D-8DAA-7E3F0E3B63BB

##### Materials

- **Type status:** Other material. **Occurrence:** occurrenceID: 7B076390-BFEB-576A-B83A-0665C1D96A29; **Location:** country: Italy; countryCode: IT; stateProvince: Sassari; locality: Island of Culuccia; verbatimLatitude: 41 12 28.07N; verbatimLongitude: 9 17 47.62E; geodeticDatum: WGS84

##### Notes

Alive.

#### Calliostomatidae

DD63F9CE-6545-584A-8144-1E78FC348FA7

#### Calliostoma laugieri

(Payraudeau, 1826)

6E69AE50-9CBD-5B36-8154-FC7A71481CA6

##### Materials

- **Type status:** Other material. **Occurrence:** occurrenceID: 2A1D8E9B-16C9-5F3A-B0F1-BBC5DB8A0027; **Location:** country: Italy; countryCode: IT; stateProvince: Sassari; locality: Island of Culuccia; verbatimLatitude: 41 13 17.70N; verbatimLongitude: 9 17 21.41E; geodeticDatum: WGS84

##### Notes

Alive, Fig. 21.

#### Skeneidae

63DE277A-88DF-5902-A567-0E82DDE5EF2C

#### Skenea serpuloides

(Montagu, 1808)

3F6A3C4F-8F97-5310-B569-23243AA7E34C

##### Materials

- **Type status:** Other material. **Occurrence:** occurrenceID: 39262B2D-4681-5D26-BD26-FEE1CBCDDE76; **Location:** country: Italy; countryCode: IT; stateProvince: Sassari; locality: Island of Culuccia; verbatimLatitude: 41 13 17.70N; verbatimLongitude: 9 17 21.41E; geodeticDatum: WGS84

##### Notes

Shell.

#### Turbinidae

0972B0CC-5D11-57EB-B97A-9F92A29D2822

#### Bolma rugosa

(Linnaeus 1767)

AB30F5AF-4544-5722-B94F-C6220500D706

##### Materials

- **Type status:** Other material. **Occurrence:** occurrenceID: 232513C8-B0DE-5FD2-B38A-5816A3A9FE1C; **Location:** country: Italy; countryCode: IT; stateProvince: Sassari; locality: Island of Culuccia; verbatimLatitude: 41 12 13.88N; verbatimLongitude: 9 17 56.14E; geodeticDatum: WGS84
- **Type status:** Other material. **Occurrence:** occurrenceID: 6F453133-DE4F-50F7-875B-79D953841A10; **Location:** country: Italy; countryCode: IT; stateProvince: Sassari; locality: Island of Culuccia; verbatimLatitude: 41 12 28.07N; verbatimLongitude: 9 17 47.62E; geodeticDatum: WGS84

##### Notes

Alive, Fig. 22.

#### Homalopoma sanguineum

(Linnaeus, 1758)

3F22DB05-C4E3-5110-BC04-ECA4B9906FD3

##### Materials

- **Type status:** Other material. **Occurrence:** occurrenceID: 788C7F83-FA45-5CD0-BAAD-EC14F01DB3F7; **Location:** country: Italy; countryCode: IT; stateProvince: Sassari; locality: Island of Culuccia; verbatimLatitude: 41 13 17.70N; verbatimLongitude: 9 17 21.41E; geodeticDatum: WGS84

##### Notes

Shell.

#### Phasianellidae

CF9306AA-F36E-5899-86B1-E6672A0FFF72

#### Tricolia pullus

(Linnaeus, 1758)

7DE0BB4B-A6C6-5E3B-B52D-9910F8D0FF2D

##### Materials

- **Type status:** Other material. **Occurrence:** occurrenceID: 941BD127-FFAD-5A93-8D03-36E39F5A9BA1; **Location:** country: Italy; countryCode: IT; stateProvince: Sassari; locality: Island of Culuccia; verbatimLatitude: 41 13 17.70N; verbatimLongitude: 9 17 21.41E; geodeticDatum: WGS84

##### Notes

Shell.

#### Tricolia speciosa

(Von Muhelfeldt, 1824)

8B864BF0-B342-5C40-B877-BA42416C7CEB

##### Materials

- **Type status:** Other material. **Occurrence:** occurrenceID: B26B3726-9A25-51F9-A662-25B3C2F3306B; **Location:** country: Italy; countryCode: IT; stateProvince: Sassari; locality: Island of Culuccia; verbatimLatitude: 41 13 17.70N; verbatimLongitude: 9 17 21.41E; geodeticDatum: WGS84

##### Notes

Alive.

#### Cycloneritida

009B9B61-5DA0-5A79-AD00-4E8E38733F6E

#### Neritidae

372637C9-D23A-50AE-9883-776802EC9CFF

#### Smaragdia viridis

(Linnaeus, 1758)

B425A81D-CDFE-5C52-B11C-79C9E8B7E63B

##### Materials

- **Type status:** Other material. **Occurrence:** occurrenceID: A6253ABF-7D3B-50E3-B3C0-B20BA2B36ACB; **Location:** country: Italy; countryCode: IT; stateProvince: Sassari; locality: Island of Culuccia; verbatimLatitude: 41 12 28.07N; verbatimLongitude: 9 17 47.62E; geodeticDatum: WGS84

##### Notes

Alive.

#### Caenogastropoda *

55780F76-9449-5B75-824C-F18B5CF4E1F7

#### Cerithiidae

DE438463-84F7-5EEC-A440-76C903BC6478

#### Bittium reticulatum

(da Costa, 1778)

EF729E74-E35A-5F04-8E41-75D98C225703

##### Materials

- **Type status:** Other material. **Occurrence:** occurrenceID: F3C5EC89-2A33-577C-B6A0-217C23D11BC1; **Location:** country: Italy; countryCode: IT; stateProvince: Sassari; locality: Island of Culuccia; verbatimLatitude: 41 12 51.58N; verbatimLongitude: 9 17 31.46E; geodeticDatum: WGS84
- **Type status:** Other material. **Occurrence:** occurrenceID: 0E8E3303-FAB8-5B0F-80FE-08C77E5EC238; **Location:** country: Italy; countryCode: IT; stateProvince: Sassari; locality: Island of Culuccia; verbatimLatitude: 41 12 28.07N; verbatimLongitude: 9 17 47.62E; geodeticDatum: WGS84
- **Type status:** Other material. **Occurrence:** occurrenceID: ECD22F81-5F65-5FAF-BCD2-57F3DA15C7FE; **Location:** country: Italy; countryCode: IT; stateProvince: Sassari; locality: Island of Culuccia; verbatimLatitude: 41 13 17.70N; verbatimLongitude: 9 17 21.41E; geodeticDatum: WGS84

##### Notes

Alive.

#### Cerithium renovatum

Monterosato, 1884

251D29F9-37E7-5F4A-8FAE-43C1DF7C8544

##### Materials

- **Type status:** Other material. **Occurrence:** occurrenceID: E05E1A58-CCFC-525F-B7AB-143B5E67209A; **Location:** country: Italy; countryCode: IT; stateProvince: Sassari; locality: Island of Culuccia; verbatimLatitude: 41 12 51.58N; verbatimLongitude: 9 17 31.46E; geodeticDatum: WGS84
- **Type status:** Other material. **Occurrence:** occurrenceID: 23455B65-B58B-578A-8228-190B69675816; **Location:** country: Italy; countryCode: IT; stateProvince: Sassari; locality: Island of Culuccia; verbatimLatitude: 41 12 28.07N; verbatimLongitude: 9 17 47.62E; geodeticDatum: WGS84
- **Type status:** Other material. **Occurrence:** occurrenceID: DEAA48DC-96E8-5C6F-9AFA-1A73F55B182D; **Location:** country: Italy; countryCode: IT; stateProvince: Sassari; locality: Island of Culuccia; verbatimLatitude: 41 13 17.70N; verbatimLongitude: 9 17 21.41E; geodeticDatum: WGS84

##### Notes

Alive, Fig. 23.

#### Cerithium vulgatum

Bruguiere, 1792

33CF7DA2-11E9-555A-9DFC-77E4A7151579

##### Materials

- **Type status:** Other material. **Occurrence:** occurrenceID: 4AD3AF18-CA81-5F1C-BCDD-D803E4898173; **Location:** country: Italy; countryCode: IT; stateProvince: Sassari; locality: Island of Culuccia; verbatimLatitude: 41 12 51.58N; verbatimLongitude: 9 17 31.46E; geodeticDatum: WGS84
- **Type status:** Other material. **Occurrence:** occurrenceID: D2577C4E-87C9-59EA-82FF-0739AB8CAA74; **Location:** country: Italy; countryCode: IT; stateProvince: Sassari; locality: Island of Culuccia; verbatimLatitude: 41 12 28.07N; verbatimLongitude: 9 17 47.62E; geodeticDatum: WGS84
- **Type status:** Other material. **Occurrence:** occurrenceID: 239FC8DB-F4A0-504C-994C-C1A733733273; **Location:** country: Italy; countryCode: IT; stateProvince: Sassari; locality: Island of Culuccia; verbatimLatitude: 41 13 17.70N; verbatimLongitude: 9 17 21.41E; geodeticDatum: WGS84

##### Notes

Alive, Fig. 24.

#### Potamididae

5F3A0A4B-FF86-5F7F-A75D-C109913E19A7

#### Pirenella conica

(Blainville, 1826)

B016B836-C861-5C1C-AA67-C86229D8752D

##### Materials

- **Type status:** Other material. **Occurrence:** occurrenceID: 53F9AAFE-5BF5-5C7C-A45E-22CF97FB5FC3; **Location:** country: Italy; countryCode: IT; stateProvince: Sassari; locality: Island of Culuccia; verbatimLatitude: 41 11 22.14N; verbatimLongitude: 9 16 59.05E; geodeticDatum: WGS84

##### Notes

Alive.

#### Turritellidae

E145AD76-8A6D-52EF-838C-0B14150E116B

#### Turritella turbona

Monterosato, 1887

7D5BF311-71E3-5BDD-AA52-AED58453C9FA

##### Materials

- **Type status:** Other material. **Occurrence:** occurrenceID: CB0CA8B0-2686-52D5-822A-F4EAFE429437; **Location:** country: Italy; countryCode: IT; stateProvince: Sassari; locality: Island of Culuccia; verbatimLatitude: 41 13 17.70N; verbatimLongitude: 9 17 21.41E; geodeticDatum: WGS84

##### Notes

Shell.

#### Triphoridae

B5D13F43-E78C-5C72-94F8-99966FF8260B

#### Metaxia metaxa

(Delle Chiaje, 1828)

8D7BEEC6-D155-55C8-9CB9-E0449C6B217D

##### Materials

- **Type status:** Other material. **Occurrence:** occurrenceID: B455DD48-E160-546E-A146-01176992A758; **Location:** country: Italy; countryCode: IT; stateProvince: Sassari; locality: Island of Culuccia; verbatimLatitude: 41 13 17.70N; verbatimLongitude: 9 17 21.41E; geodeticDatum: WGS84

##### Notes

Shell.

#### Monophorus perversus

(Linnaeus, 1758)

29AF7C03-3015-50BB-8313-BB037B16BD72

##### Materials

- **Type status:** Other material. **Occurrence:** occurrenceID: 18EA033A-22C9-55E1-B454-36D4B7444217; **Location:** country: Italy; countryCode: IT; stateProvince: Sassari; locality: Island of Culuccia; verbatimLatitude: 41 13 17.70N; verbatimLongitude: 9 17 21.41E; geodeticDatum: WGS84

##### Notes

Shell.

#### Marshallora adversa

(Montagu, 1803)

3282AF9C-C3FE-5469-9579-2B0BF6F65218

##### Materials

- **Type status:** Other material. **Occurrence:** occurrenceID: FE9E4FED-1FAA-5B91-B095-F2FCFD07D274; **Location:** country: Italy; countryCode: IT; stateProvince: Sassari; locality: Island of Culuccia; verbatimLatitude: 41 13 17.70N; verbatimLongitude: 9 17 21.41E; geodeticDatum: WGS84

##### Notes

Shell.

#### Cerithiopsidae

45A86A83-C598-5D22-A698-4431BC00370A

#### Cerithiopsis tubercularis

(Montagu, 1803)

3F437804-6980-5D3A-AD33-8CE37AE0FFAF

##### Materials

- **Type status:** Other material. **Occurrence:** occurrenceID: D3F5ED9C-94EE-56C5-9ED0-56CE0AEF353E; **Location:** country: Italy; countryCode: IT; stateProvince: Sassari; locality: Island of Culuccia; verbatimLatitude: 41 13 17.70N; verbatimLongitude: 9 17 21.41E; geodeticDatum: WGS84

##### Notes

Shell.

#### Epitoniidae

351EB7F3-7C46-5502-AD8F-C40FEE29FB63

#### Epitonium clathrus

(Linnaeus, 1758)

7CDD8C23-40F1-53A1-B1AC-F7CFE3183063

##### Materials

- **Type status:** Other material. **Occurrence:** occurrenceID: 7E463BB1-80B6-5FCE-A079-78CD9C6A54EE; **Location:** country: Italy; countryCode: IT; stateProvince: Sassari; locality: Island of Culuccia; verbatimLatitude: 41 12 42.40N; verbatimLongitude: 9 17 45.35E; geodeticDatum: WGS84

##### Notes

Shell.

#### Littorinimorpha

67E668D9-16EA-5136-8358-549C70ABCAED

#### Eulimidae

F817425C-C890-50C3-A017-2453778993E8

#### Eulima glabra

(da Costa, 1778)

991FA655-4434-59C4-BFE5-DD969894039F

##### Materials

- **Type status:** Other material. **Occurrence:** occurrenceID: F8A9561A-7EDC-5527-BF35-B9FDD23D6F82; **Location:** country: Italy; countryCode: IT; stateProvince: Sassari; locality: Island of Culuccia; verbatimLatitude: 41 13 17.70N; verbatimLongitude: 9 17 21.41E; geodeticDatum: WGS84

##### Notes

Shell.

#### Eulima sp.

670AA7D9-7855-567A-9B1D-D9BC51A2DB56

##### Materials

- **Type status:** Other material. **Occurrence:** occurrenceID: 865263BF-3A0C-5EAB-8C68-2394D185A178; **Location:** country: Italy; countryCode: IT; stateProvince: Sassari; locality: Island of Culuccia; verbatimLatitude: 41 13 17.70N; verbatimLongitude: 9 17 21.41E; geodeticDatum: WGS84

##### Notes

Shell.

#### Melanella polita

(Linnaeus, 1758)

FEE278A0-DBB0-53CE-8D0D-C6C868E1E8EA

##### Materials

- **Type status:** Other material. **Occurrence:** occurrenceID: B8557482-350D-51B8-AAD8-34156E0A0413; **Location:** country: Italy; countryCode: IT; stateProvince: Sassari; locality: Island of Culuccia; verbatimLatitude: 41 13 17.70N; verbatimLongitude: 9 17 21.41E; geodeticDatum: WGS84

##### Notes

Shell.

#### Parvioris ibizenca

(F. Nordsieck, 1968)

B957D048-D1F3-521D-B7F2-472BD99E7FBD

##### Materials

- **Type status:** Other material. **Occurrence:** occurrenceID: 29FEFC07-3C2A-5BF6-9D2F-7C6284877927; **Location:** country: Italy; countryCode: IT; stateProvince: Sassari; locality: Island of Culuccia; verbatimLatitude: 41 13 17.70N; verbatimLongitude: 9 17 21.41E; geodeticDatum: WGS84

##### Notes

Shell.

#### Vanikoridae

B9705228-70E4-50E9-AA2F-56EBD6028F24

#### Megalomphalus azonus

(Brusina, 1865)

4C94C44F-3212-57F1-9220-01D856A81ABB

##### Materials

- **Type status:** Other material. **Occurrence:** occurrenceID: 15359214-B528-540C-94BB-52B7BF63F877; **Location:** country: Italy; countryCode: IT; stateProvince: Sassari; locality: Island of Culuccia; verbatimLatitude: 41 13 17.70N; verbatimLongitude: 9 17 21.41E; geodeticDatum: WGS84

##### Notes

Shell.

#### Rissoidae

031620AC-3A81-57FA-9397-8A742DDB7DD8

#### Pusillina inconspicua

(Alder, 1844)

5113A98D-7D6F-5986-923A-02841B5DCD24

##### Materials

- **Type status:** Other material. **Occurrence:** occurrenceID: 13A23877-2AD0-5BD9-8AB8-50C01CC5E2AF; **Location:** country: Italy; countryCode: IT; stateProvince: Sassari; locality: Island of Culuccia; verbatimLatitude: 41 13 17.70N; verbatimLongitude: 9 17 21.41E; geodeticDatum: WGS84

##### Notes

Shell.

#### Pusillina marginata

(Michaud, 1830)

A917B2BB-5D75-560E-BE0C-8F771C3CFD87

##### Materials

- **Type status:** Other material. **Occurrence:** occurrenceID: 27BB286A-9F81-5D6A-A0C1-22E8CC112C73; **Location:** country: Italy; countryCode: IT; stateProvince: Sassari; locality: Island of Culuccia; verbatimLatitude: 41 13 17.70N; verbatimLongitude: 9 17 21.41E; geodeticDatum: WGS84

##### Notes

Shell.

#### Pusillina radiata

(Philippi, 1836)

40A591AD-0700-534E-8473-742FE4BB6854

##### Materials

- **Type status:** Other material. **Occurrence:** occurrenceID: 559673D2-16B3-5A26-9D84-E3F93A46AECA; **Location:** country: Italy; countryCode: IT; stateProvince: Sassari; locality: Island of Culuccia; verbatimLatitude: 41 13 17.70N; verbatimLongitude: 9 17 21.41E; geodeticDatum: WGS84

##### Notes

Shell.

#### Rissoa aartseni

Verduin, 1985

C1EEC37C-9CF2-5C7C-A859-AE778879CD55

##### Materials

- **Type status:** Other material. **Occurrence:** occurrenceID: 2347D1FD-FA48-5CA3-BA7A-2239441303D3; **Location:** country: Italy; countryCode: IT; stateProvince: Sassari; locality: Island of Culuccia; verbatimLatitude: 41 13 17.70N; verbatimLongitude: 9 17 21.41E; geodeticDatum: WGS84

##### Notes

Shell; new record.

#### Rissoa auriscalpium

(Linnaeus, 1758)

FC314504-29F7-5F2C-8EC0-F5AD1AB88281

##### Materials

- **Type status:** Other material. **Occurrence:** occurrenceID: D64D816D-8751-5AB5-9D16-9F5049BFEFCB; **Location:** country: Italy; countryCode: IT; stateProvince: Sassari; locality: Island of Culuccia; verbatimLatitude: 41 13 17.70N; verbatimLongitude: 9 17 21.41E; geodeticDatum: WGS84

##### Notes

Alive.

#### Rissoa lia

(Monterosato, 1884)

3228B844-34B6-55B4-A50E-99CDF717EF33

##### Materials

- **Type status:** Other material. **Occurrence:** occurrenceID: 2392023A-D8A5-5269-AE3D-52A2211B3FBB; **Location:** country: Italy; countryCode: IT; stateProvince: Sassari; locality: Island of Culuccia; verbatimLatitude: 41 13 17.70N; verbatimLongitude: 9 17 21.41E; geodeticDatum: WGS84

##### Notes

Shell.

#### Rissoa membranacea

(J. Adams, 1800)

BE971617-0203-5A98-A106-D4C305E1362A

##### Materials

- **Type status:** Other material. **Occurrence:** occurrenceID: B6CDE519-4121-565F-9FA6-F99E8AE826B4; **Location:** country: Italy; countryCode: IT; stateProvince: Sassari; locality: Island of Culuccia; verbatimLatitude: 41 13 17.70N; verbatimLongitude: 9 17 21.41E; geodeticDatum: WGS84
- **Type status:** Other material. **Occurrence:** occurrenceID: D0679C77-68CA-59D7-ADA3-F8E0B71AFBCB; **Location:** country: Italy; countryCode: IT; stateProvince: Sassari; locality: Island of Culuccia; verbatimLatitude: 41 12 58.16N; verbatimLongitude: 9 17 09.66E; geodeticDatum: WGS84

##### Notes

Shell.

#### Rissoa sp.

340B7C35-9F74-556C-95D3-D6B8DFF56725

##### Materials

- **Type status:** Other material. **Occurrence:** occurrenceID: 4F09EA95-DF48-5B41-8E6E-ADDDA1506DB7; **Location:** country: Italy; countryCode: IT; stateProvince: Sassari; locality: Island of Culuccia; verbatimLatitude: 41 13 17.70N; verbatimLongitude: 9 17 21.41E; geodeticDatum: WGS84

##### Notes

Shell.

#### Rissoa variabilis

(Megerle von Mühlfeld, 1824)

F4961B1F-E261-58B2-B915-E95DE07EF965

##### Materials

- **Type status:** Other material. **Occurrence:** occurrenceID: E70CC4E6-486A-5BAE-AA01-3A1715E57760; **Location:** country: Italy; countryCode: IT; stateProvince: Sassari; locality: Island of Culuccia; verbatimLatitude: 41 13 17.70N; verbatimLongitude: 9 17 21.41E; geodeticDatum: WGS84

##### Notes

Shell.

#### Rissoa ventricosa

Desmarest, 1814

2EE519A3-46B3-53F3-9F5C-633F881837F6

##### Materials

- **Type status:** Other material. **Occurrence:** occurrenceID: A1B8C923-2710-5990-9EAC-CC4010C392AA; **Location:** country: Italy; countryCode: IT; stateProvince: Sassari; locality: Island of Culuccia; verbatimLatitude: 41 13 17.70N; verbatimLongitude: 9 17 21.41E; geodeticDatum: WGS84

##### Notes

Shell.

#### Rissoa violacea

Desmarest, 1814

ECF3DA80-0732-5797-A653-55046CE3862A

##### Materials

- **Type status:** Other material. **Occurrence:** occurrenceID: 655A929F-B1E0-59AF-B749-5A05E436F46E; **Location:** country: Italy; countryCode: IT; stateProvince: Sassari; locality: Island of Culuccia; verbatimLatitude: 41 13 17.70N; verbatimLongitude: 9 17 21.41E; geodeticDatum: WGS84

##### Notes

Shell.

#### Alvania beanii

(Hanley, 1844)

DC98697F-E56A-5223-9001-966C9F0655D8

##### Materials

- **Type status:** Other material. **Occurrence:** occurrenceID: D262DFA3-9783-53E1-B5A4-FD7EAB8E054D; **Location:** country: Italy; countryCode: IT; stateProvince: Sassari; locality: Island of Culuccia; verbatimLatitude: 41 13 17.70N; verbatimLongitude: 9 17 21.41E; geodeticDatum: WGS84

##### Notes

Shell, Fig. 25.

#### Alvania carinata

(da Costa, 1778)

7FD6E710-40B2-5022-B68C-0E82449A2214

##### Materials

- **Type status:** Other material. **Occurrence:** occurrenceID: D0993C33-DBAB-50C1-9F58-7EDD89BCA3DC; **Location:** country: Italy; countryCode: IT; stateProvince: Sassari; locality: Island of Culuccia; verbatimLatitude: 41 13 17.70N; verbatimLongitude: 9 17 21.41E; geodeticDatum: WGS84

##### Notes

Shell.

#### Alvania lactea

(Michaud, 1830)

51DF66A0-D5D6-553F-B7D6-CF6D12256E19

##### Materials

- **Type status:** Other material. **Occurrence:** occurrenceID: 2D439D13-3F2B-50CC-9D00-C40728E6DB15; **Location:** country: Italy; countryCode: IT; stateProvince: Sassari; locality: Island of Culuccia; verbatimLatitude: 41 13 17.70N; verbatimLongitude: 9 17 21.41E; geodeticDatum: WGS84

##### Notes

Shell.

#### Alvania lineata

Risso, 1826

1CD5E2E4-DB18-511E-BC36-F35AD67489B0

##### Materials

- **Type status:** Other material. **Occurrence:** occurrenceID: 4A9E0933-A974-5B6F-B083-A8D1C08FDD68; **Location:** country: Italy; countryCode: IT; stateProvince: Sassari; locality: Island of Culuccia; verbatimLatitude: 41 13 17.70N; verbatimLongitude: 9 17 21.41E; geodeticDatum: WGS84

##### Notes

Shell.

#### Alvania mamillata

Risso, 1826

4CC3A54B-15AD-577C-87DC-EF7262E03D43

##### Materials

- **Type status:** Other material. **Occurrence:** occurrenceID: BF21DF0C-52DD-5B48-B797-534C91F95872; **Location:** country: Italy; countryCode: IT; stateProvince: Sassari; locality: Island of Culuccia; verbatimLatitude: 41 13 17.70N; verbatimLongitude: 9 17 21.41E; geodeticDatum: WGS84

##### Notes

Shell.

#### Alvania scabra

(Philippi, 1844)

F6EA5E3E-C1B8-5617-8443-62B87675F88D

##### Materials

- **Type status:** Other material. **Occurrence:** occurrenceID: 8081AAAC-EB13-579D-8D3B-F39D054C566F; **Location:** country: Italy; countryCode: IT; stateProvince: Sassari; locality: Island of Culuccia; verbatimLatitude: 41 13 17.70N; verbatimLongitude: 9 17 21.41E; geodeticDatum: WGS84

##### Notes

Shell.

#### Rissoina bruguieri

(Payraudeau, 1826)

4304E56F-6213-5BDD-9DCA-50B1EC692BCF

##### Materials

- **Type status:** Other material. **Occurrence:** occurrenceID: 4719A908-B1A0-5AC4-8169-B320FDE4D6E2; **Location:** country: Italy; countryCode: IT; stateProvince: Sassari; locality: Island of Culuccia; verbatimLatitude: 41 13 17.70N; verbatimLongitude: 9 17 21.41E; geodeticDatum: WGS84

##### Notes

Shell.

#### Crisilla semistriata

(Montagu, 1808)

6DEA752B-CD55-5D97-814E-B06D077A4813

##### Materials

- **Type status:** Other material. **Occurrence:** occurrenceID: C1CC44A2-C159-5AAE-A14D-77CF965D4F32; **Location:** country: Italy; countryCode: IT; stateProvince: Sassari; locality: Island of Culuccia; verbatimLatitude: 41 13 17.70N; verbatimLongitude: 9 17 21.41E; geodeticDatum: WGS84

##### Notes

Shell; new record.

#### Manzonia crassa

(Kanmacher, 1798)

28AAA31F-2581-53AF-9AE0-13BF7F124C8E

##### Materials

- **Type status:** Other material. **Occurrence:** occurrenceID: F53E6B40-231A-5D22-B6D3-515559EC04DA; **Location:** country: Italy; countryCode: IT; stateProvince: Sassari; locality: Island of Culuccia; verbatimLatitude: 41 13 17.70N; verbatimLongitude: 9 17 21.41E; geodeticDatum: WGS84

##### Notes

Shell; new record.

#### Caecidae

C0A48707-1E4A-5162-AFBC-FD66F0085DFB

#### Caecum subannulatum

de Folin, 1870

DF90F671-E80D-5EDF-A283-9107B668D3D9

##### Materials

- **Type status:** Other material. **Occurrence:** occurrenceID: D53FD71F-78DE-53E6-984A-8D000D4E39D2; **Location:** country: Italy; countryCode: IT; stateProvince: Sassari; locality: Island of Culuccia; verbatimLatitude: 41 13 17.70N; verbatimLongitude: 9 17 21.41E; geodeticDatum: WGS84

##### Notes

Shell, Fig. 26.

#### Caecum trachea

(Montagu, 1803)

7F7C2BCB-A111-55C7-A87F-E2D84904A0D7

##### Materials

- **Type status:** Other material. **Occurrence:** occurrenceID: 22DF3666-A051-52F8-9664-A4593F5E19D6; **Location:** country: Italy; countryCode: IT; stateProvince: Sassari; locality: Island of Culuccia; verbatimLatitude: 41 13 17.70N; verbatimLongitude: 9 17 21.41E; geodeticDatum: WGS84

##### Notes

Shell.

#### Truncartellidae

24D53888-4D05-56C1-9F1F-A26C98D2358D

#### Truncatella subcylindrica

(Linnaeus, 1767)

75263C5E-81CE-563C-A9E4-94129F21370A

##### Materials

- **Type status:** Other material. **Occurrence:** occurrenceID: B7BE9276-2C2C-5F52-99DF-85D1C311F083; **Location:** country: Italy; countryCode: IT; stateProvince: Sassari; locality: Island of Culuccia; verbatimLatitude: 41 13 17.70N; verbatimLongitude: 9 17 21.41E; geodeticDatum: WGS84

##### Notes

Shell.

#### Calyptreidae

EFE6E2AC-ACAF-5307-886C-337CA4ED0FDA

#### Calyptraea chinensis

(Linnaeus, 1758)

ADA8EE88-C227-50CA-8392-85186A4328C7

##### Materials

- **Type status:** Other material. **Occurrence:** occurrenceID: E6549B97-46C5-5640-917A-0B0CFD8813AE; **Location:** country: Italy; countryCode: IT; stateProvince: Sassari; locality: Island of Culuccia; verbatimLatitude: 41 13 17.70N; verbatimLongitude: 9 17 21.41E; geodeticDatum: WGS84

##### Notes

Shell.

#### Crepidula unguiformis

Lamarck, 1822

880B3394-6C76-5848-A624-4AE17B4357C9

##### Materials

- **Type status:** Other material. **Occurrence:** occurrenceID: 6611CD81-DF3B-5575-A0BE-1DAB89CFBA93; **Location:** country: Italy; countryCode: IT; stateProvince: Sassari; locality: Island of Culuccia; verbatimLatitude: 41 13 17.70N; verbatimLongitude: 9 17 21.41E; geodeticDatum: WGS84

##### Notes

Shell.

#### Triviidae

1B9AD978-FF90-5BD9-945E-BA46DA6A2F83

#### Trivia arctica

(Pulteney, 1799)

A019892B-134B-54C7-B165-0D826E237146

##### Materials

- **Type status:** Other material. **Occurrence:** occurrenceID: EE7E528B-E744-577D-AC13-DAA4673C4C22; **Location:** country: Italy; countryCode: IT; stateProvince: Sassari; locality: Island of Culuccia; verbatimLatitude: 41 13 17.70N; verbatimLongitude: 9 17 21.41E; geodeticDatum: WGS84

##### Notes

Shell.

#### Trivia pulex

(Pulteney, 1799)

C972C40E-D122-5F70-B6B2-48C6F62ADF9F

##### Materials

- **Type status:** Other material. **Occurrence:** occurrenceID: 8A6C1933-FB5A-5A7A-ACF3-55306E5C6A0D; **Location:** country: Italy; countryCode: IT; stateProvince: Sassari; locality: Island of Culuccia; verbatimLatitude: 41 13 17.70N; verbatimLongitude: 9 17 21.41E; geodeticDatum: WGS84

##### Notes

Alive, Fig. 27.

#### Vermetidae

233080A5-6B03-5962-88D6-E25A9F379954

#### Vermetus granulatus

(Gravenhorst, 1831)

C3A320C2-520E-573D-9A09-6A37E214B9D2

##### Materials

- **Type status:** Other material. **Occurrence:** occurrenceID: FDC25DD6-A7F8-5E99-BFE9-074F22D5802A; **Location:** country: Italy; countryCode: IT; stateProvince: Sassari; locality: Island of Culuccia; verbatimLatitude: 41 12 51.58N; verbatimLongitude: 9 17 31.46E; geodeticDatum: WGS84
- **Type status:** Other material. **Occurrence:** occurrenceID: 7DA5436D-6DE9-55E1-A61E-E664905B60BE; **Location:** country: Italy; countryCode: IT; stateProvince: Sassari; locality: Island of Culuccia; verbatimLatitude: 41 12 13.88N; verbatimLongitude: 9 17 56.14E; geodeticDatum: WGS84
- **Type status:** Other material. **Occurrence:** occurrenceID: 98A13A30-BD3A-5E7C-BC4E-48D15AFBEB95; **Location:** country: Italy; countryCode: IT; stateProvince: Sassari; locality: Island of Culuccia; verbatimLatitude: 41 12 28.07N; verbatimLongitude: 9 17 47.62E; geodeticDatum: WGS84
- **Type status:** Other material. **Occurrence:** occurrenceID: D1C21F8A-A5C1-5AB7-B629-65EF7EA56F21; **Location:** country: Italy; countryCode: IT; stateProvince: Sassari; locality: Island of Culuccia; verbatimLatitude: 41 12 42.40N; verbatimLongitude: 9 17 45.35E; geodeticDatum: WGS84
- **Type status:** Other material. **Occurrence:** occurrenceID: C2650E29-6138-5C00-99C8-8D15BC9D232A; **Location:** country: Italy; countryCode: IT; stateProvince: Sassari; locality: Island of Culuccia; verbatimLatitude: 41 13 17.70N; verbatimLongitude: 9 17 21.41E; geodeticDatum: WGS84

##### Notes

Alive, Fig. 28.

#### Cypreaidae

29CDA3E5-0117-5AE0-B7D2-7515B3CEF5C4

#### Luria lurida

(Linnaeus, 1758)

10F1EA95-565D-5A9D-8060-C4BD0B048282

##### Materials

- **Type status:** Other material. **Occurrence:** occurrenceID: 25BC7385-2AA3-5FCC-98E0-507D8ACC997B; **Location:** country: Italy; countryCode: IT; stateProvince: Sassari; locality: Island of Culuccia; verbatimLatitude: 41 12 28.07N; verbatimLongitude: 9 17 47.62E; geodeticDatum: WGS84
- **Type status:** Other material. **Occurrence:** occurrenceID: 4CDA040B-15A6-5252-A0EA-08A146F26C7F; **Location:** country: Italy; countryCode: IT; stateProvince: Sassari; locality: Island of Culuccia; verbatimLatitude: 41 13 17.70N; verbatimLongitude: 9 17 21.41E; geodeticDatum: WGS84

##### Notes

Alive, Fig. 29.

#### Naria spurca

(Linnaeus, 1758)

A4312037-C427-5FD1-A4DB-2BB948408C87

##### Materials

- **Type status:** Other material. **Occurrence:** occurrenceID: 46708C9F-EDFB-57D5-8D54-5358951EDA56; **Location:** country: Italy; countryCode: IT; stateProvince: Sassari; locality: Island of Culuccia; verbatimLatitude: 41 12 28.07N; verbatimLongitude: 9 17 47.62E; geodeticDatum: WGS84
- **Type status:** Other material. **Occurrence:** occurrenceID: F6AF4EEE-F860-57B2-838D-86EDB9131560; **Location:** country: Italy; countryCode: IT; stateProvince: Sassari; locality: Island of Culuccia; verbatimLatitude: 41 13 17.70N; verbatimLongitude: 9 17 21.41E; geodeticDatum: WGS84

##### Notes

Alive, Fig. 30.

#### Naticidae

A2713776-B346-5C72-A0B0-6077EAEFDEB5

#### Euspira nitida

(Donovan, 1803)

5A62D26F-CC75-5A4D-817C-57EAC05B0991

##### Materials

- **Type status:** Other material. **Occurrence:** occurrenceID: 9665B106-D8E4-543B-A677-E9A0C78BD854; **Location:** country: Italy; countryCode: IT; stateProvince: Sassari; locality: Island of Culuccia; verbatimLatitude: 41 13 17.70N; verbatimLongitude: 9 17 21.41E; geodeticDatum: WGS84

##### Notes

Shell.

#### Payraudeautia intricata

(Donovan, 1804)

050E256A-2F64-5038-AC56-4D44ED6D2B1C

##### Materials

- **Type status:** Other material. **Occurrence:** occurrenceID: 231A2095-2872-5F48-ABDF-95BA42B51A22; **Location:** country: Italy; countryCode: IT; stateProvince: Sassari; locality: Island of Culuccia; verbatimLatitude: 41 13 17.70N; verbatimLongitude: 9 17 21.41E; geodeticDatum: WGS84

##### Notes

Shell.

#### Naticarius hebraeus

(Martyn, 1786)

7317D339-42C3-59E4-9F9A-9EEF1FD99442

##### Materials

- **Type status:** Other material. **Occurrence:** occurrenceID: C619E69D-4B74-5B5C-A418-2A829F9DB6F6; **Location:** country: Italy; countryCode: IT; stateProvince: Sassari; locality: Island of Culuccia; verbatimLatitude: 41 11 46.79N; verbatimLongitude: 9 17 20.34E; geodeticDatum: WGS84

##### Notes

Shell.

#### Neverita josephinia

Risso, 1826

E152BC10-86E6-51FD-875B-5F942A8D101B

##### Materials

- **Type status:** Other material. **Occurrence:** occurrenceID: 62BDF85F-5842-5A00-9D3C-93606B6167B3; **Location:** country: Italy; countryCode: IT; stateProvince: Sassari; locality: Island of Culuccia; verbatimLatitude: 41 11 46.79N; verbatimLongitude: 9 17 20.34E; geodeticDatum: WGS84
- **Type status:** Other material. **Occurrence:** occurrenceID: B91D8D20-637F-5E0C-A35F-2A11CE8CF3A4; **Location:** country: Italy; countryCode: IT; stateProvince: Sassari; locality: Island of Culuccia; verbatimLatitude: 41 11 56.04N; verbatimLongitude: 9 17 33.63E; geodeticDatum: WGS84

##### Notes

Shell.

#### Cassidae

F5238E3C-DC10-518D-BF80-F3F8FB3F04BD

#### Phalium granulatum

(Born, 1778)

4EA47F9F-391F-5C3D-89F0-AD9D5E55BFFA

##### Materials

- **Type status:** Other material. **Occurrence:** occurrenceID: 909BA5C0-EFCC-5BFA-B731-89DE5C25F1D2; **Location:** country: Italy; countryCode: IT; stateProvince: Sassari; locality: Island of Culuccia; verbatimLatitude: 41 12 28.07N; verbatimLongitude: 9 17 47.62E; geodeticDatum: WGS84

##### Notes

Alive, Fig. 31.

#### Bursidae

2F54A365-E84F-5F6A-AE6F-7A9EFC3FFA7D

#### Talisman scrobilator

(Linnaeus, 1758)

B2892ACB-3348-5847-A703-DBD53C5AAB9C

##### Materials

- **Type status:** Other material. **Occurrence:** occurrenceID: 83D49A74-F4CE-5516-862A-2D1AA816E4A3; **Location:** country: Italy; countryCode: IT; stateProvince: Sassari; locality: Island of Culuccia; verbatimLatitude: 41 13 17.70N; verbatimLongitude: 9 17 21.41E; geodeticDatum: WGS84

##### Notes

Shell.

#### Neogastropoda

EBF1CCA0-2FEF-57DE-A9BB-951D88C7779F

#### Muricidae

B48A14BD-D89C-5251-BEF7-7D83275968E5

#### Hexaplex trunculus

(Linnaeus, 1758)

5576A734-5BC4-5EDA-B987-419932CD2815

##### Materials

- **Type status:** Other material. **Occurrence:** occurrenceID: A690240D-407E-570B-B021-97A10AAE9546; **Location:** country: Italy; countryCode: IT; stateProvince: Sassari; locality: Island of Culuccia; verbatimLatitude: 41 12 28.07N; verbatimLongitude: 9 17 47.62E; geodeticDatum: WGS84
- **Type status:** Other material. **Occurrence:** occurrenceID: 38E7F6C5-73B0-5AE4-B0CA-25EF126AEB5F; **Location:** country: Italy; countryCode: IT; stateProvince: Sassari; locality: Island of Culuccia; verbatimLatitude: 41 11 28.32N; verbatimLongitude: 9 16 44.50E; geodeticDatum: WGS84
- **Type status:** Other material. **Occurrence:** occurrenceID: A9B7AC66-4D5A-5A73-B10C-754BF84FEDAD; **Location:** country: Italy; countryCode: IT; stateProvince: Sassari; locality: Island of Culuccia; verbatimLatitude: 41 11 22.14N; verbatimLongitude: 9 16 59.05E; geodeticDatum: WGS84

##### Notes

Alive, Fig. 32.

#### Bolinus brandaris

(Linnaeus, 1758)

AF3EF385-1246-51B2-86DD-ABACF2BB4A3F

##### Materials

- **Type status:** Other material. **Occurrence:** occurrenceID: EE9AB88B-2B4E-528D-ACED-70F6589B9B81; **Location:** country: Italy; countryCode: IT; stateProvince: Sassari; locality: Island of Culuccia; verbatimLatitude: 41 11 28.32N; verbatimLongitude: 9 16 44.50E; geodeticDatum: WGS84

##### Notes

Alive.

#### Dermomurex scalaroides

(de Blainville, 1829)

EDAEA6BF-8337-5B6B-A972-1343DB3FDDC8

##### Materials

- **Type status:** Other material. **Occurrence:** occurrenceID: E8B8281C-336F-52DC-A6A7-98B498B3B90D; **Location:** country: Italy; countryCode: IT; stateProvince: Sassari; locality: Island of Culuccia; verbatimLatitude: 41 13 17.70N; verbatimLongitude: 9 17 21.41E; geodeticDatum: WGS84

##### Notes

Shell.

#### Typhinellus labiatus

(de Cristofori & Jan, 1832)

538BC623-F770-5084-8CE6-114CCB7092FD

##### Materials

- **Type status:** Other material. **Occurrence:** occurrenceID: 33A17A5F-CC8F-59EB-9FCA-7C3BC0F099D4; **Location:** country: Italy; countryCode: IT; stateProvince: Sassari; locality: Island of Culuccia; verbatimLatitude: 41 13 17.70N; verbatimLongitude: 9 17 21.41E; geodeticDatum: WGS84

##### Notes

Shell.

#### Ocenebra edwardsii

(Payraudeau, 1826)

63021154-EACE-5607-B3DB-E42D648B6C71

##### Materials

- **Type status:** Other material. **Occurrence:** occurrenceID: 3763CD0A-26FB-5A10-874B-77B4B0C4C212; **Location:** country: Italy; countryCode: IT; stateProvince: Sassari; locality: Island of Culuccia; verbatimLatitude: 41 12 51.58N; verbatimLongitude: 9 17 31.46E; geodeticDatum: WGS84
- **Type status:** Other material. **Occurrence:** occurrenceID: 2C194CBF-7FB8-542E-B36C-C3387DAC0B9E; **Location:** country: Italy; countryCode: IT; stateProvince: Sassari; locality: Island of Culuccia; verbatimLatitude: 41 12 13.88N; verbatimLongitude: 9 17 56.14E; geodeticDatum: WGS84

##### Notes

Shell.

#### Ocinebrina corallina

Scacchi, 1836

E28AB474-CFA9-566B-B772-B591435953BF

##### Materials

- **Type status:** Other material. **Occurrence:** occurrenceID: 68F3918D-2022-5FED-988E-2635956EF303; **Location:** country: Italy; countryCode: IT; stateProvince: Sassari; locality: Island of Culuccia; verbatimLatitude: 41 13 17.70N; verbatimLongitude: 9 17 21.41E; geodeticDatum: WGS84

##### Notes

Shell.

#### Muricopsis cristata

(Brocchi, 1814)

EC0966D1-9F73-557B-93ED-FEED4FFF623F

##### Materials

- **Type status:** Other material. **Occurrence:** occurrenceID: 3CF77047-4FD1-5D13-BFB8-B07FC37F141D; **Location:** country: Italy; countryCode: IT; stateProvince: Sassari; locality: Island of Culuccia; verbatimLatitude: 41 11 46.79N; verbatimLongitude: 9 17 20.34E; geodeticDatum: WGS84
- **Type status:** Other material. **Occurrence:** occurrenceID: AD0D3B11-3096-55C4-B7C4-AECD622D1B38; **Location:** country: Italy; countryCode: IT; stateProvince: Sassari; locality: Island of Culuccia; verbatimLatitude: 41 11 46.14N; verbatimLongitude: 9 16 40.60E; geodeticDatum: WGS84
- **Type status:** Other material. **Occurrence:** occurrenceID: BBCBA8E4-39CD-5108-BEB8-1AB738003CCE; **Location:** country: Italy; countryCode: IT; stateProvince: Sassari; locality: Island of Culuccia; verbatimLatitude: 41 12 51.58N; verbatimLongitude: 9 17 31.46E; geodeticDatum: WGS84
- **Type status:** Other material. **Occurrence:** occurrenceID: D1B7486B-4A9C-55D9-B80E-D060A4B41960; **Location:** country: Italy; countryCode: IT; stateProvince: Sassari; locality: Island of Culuccia; verbatimLatitude: 41 13 17.70N; verbatimLongitude: 9 17 21.41E; geodeticDatum: WGS84

##### Notes

Alive, Fig. 33.

#### Stramonita haemastoma

(Linnaeus, 1767)

8B5179C4-1101-59C3-AF44-6F0D4F1B82EB

##### Materials

- **Type status:** Other material. **Occurrence:** occurrenceID: 15B98263-4A45-5FE0-8930-6A1CC9A4264C; **Location:** country: Italy; countryCode: IT; stateProvince: Sassari; locality: Island of Culuccia; verbatimLatitude: 41 12 28.07N; verbatimLongitude: 9 17 47.62E; geodeticDatum: WGS84
- **Type status:** Other material. **Occurrence:** occurrenceID: 1CA4EDCF-0925-55F0-B484-3AA20FE1DEF2; **Location:** country: Italy; countryCode: IT; stateProvince: Sassari; locality: Island of Culuccia; verbatimLatitude: 41 12 42.40N; verbatimLongitude: 9 17 45.35E; geodeticDatum: WGS84
- **Type status:** Other material. **Occurrence:** occurrenceID: BA06F2DA-3819-50F6-BBC5-56E474A43BB8; **Location:** country: Italy; countryCode: IT; stateProvince: Sassari; locality: Island of Culuccia; verbatimLatitude: 41 13 17.70N; verbatimLongitude: 9 17 21.41E; geodeticDatum: WGS84

##### Notes

Alive, Fig. 34.

#### Coralliophila meyendorffii

(Calcara, 1845)

ED445F6F-2D9E-5954-82E8-1D13143D51FC

##### Materials

- **Type status:** Other material. **Occurrence:** occurrenceID: 181BAAAA-C3D7-57AB-9DCE-0888FEB7A465; **Location:** country: Italy; countryCode: IT; stateProvince: Sassari; locality: Island of Culuccia; verbatimLatitude: 41 12 42.40N; verbatimLongitude: 9 17 45.35E; geodeticDatum: WGS84
- **Type status:** Other material. **Occurrence:** occurrenceID: 769593F3-8D00-5459-A1F9-9C05F94AC08F; **Location:** country: Italy; countryCode: IT; stateProvince: Sassari; locality: Island of Culuccia; verbatimLatitude: 41 12 58.16N; verbatimLongitude: 9 17 09.66E; geodeticDatum: WGS84

##### Notes

Alive, Fig. 35.

#### Cystiscidae

EE75C3BC-E935-53D6-9AD0-BF8F19FAB29E

#### Granulina marginata

(Bivona, 1832)

028743CE-1C49-5263-AA43-8A2AC600D562

##### Materials

- **Type status:** Other material. **Occurrence:** occurrenceID: B920D843-1C7E-5C18-9DCA-E7EB33407237; **Location:** country: Italy; countryCode: IT; stateProvince: Sassari; locality: Island of Culuccia; verbatimLatitude: 41 13 17.70N; verbatimLongitude: 9 17 21.41E; geodeticDatum: WGS84

##### Notes

Shell.

#### Marginellidae

0BFD1D16-985D-59FA-91FC-96E296BFBBD8

#### Gibberula caelata

(Monterosato, 1877)

5973F1CE-DD9E-589D-B2E6-677B15159739

##### Materials

- **Type status:** Other material. **Occurrence:** occurrenceID: D37A71A5-3DD8-54E4-B627-D716A8DBAB50; **Location:** country: Italy; countryCode: IT; stateProvince: Sassari; locality: Island of Culuccia; verbatimLatitude: 41 13 17.70N; verbatimLongitude: 9 17 21.41E; geodeticDatum: WGS84

##### Notes

Shell; new record.

#### Gibberula miliaria

(Linnaeus, 1758)

F2EE2955-F362-5FE1-B7C8-03440EC50224

##### Materials

- **Type status:** Other material. **Occurrence:** occurrenceID: 2E70238A-804C-5621-A412-5061CB622253; **Location:** country: Italy; countryCode: IT; stateProvince: Sassari; locality: Island of Culuccia; verbatimLatitude: 41 13 17.70N; verbatimLongitude: 9 17 21.41E; geodeticDatum: WGS84

##### Notes

Shell.

#### Gibberula philippii

(Monterosato, 1878)

37E0B0CC-761E-55B5-A9D5-19DEEBB5759C

##### Materials

- **Type status:** Other material. **Occurrence:** occurrenceID: 2F139A7B-735F-5DF7-A65B-4A61C83972C1; **Location:** country: Italy; countryCode: IT; stateProvince: Sassari; locality: Island of Culuccia; verbatimLatitude: 41 13 17.70N; verbatimLongitude: 9 17 21.41E; geodeticDatum: WGS84

##### Notes

Shell.

#### Costellaridae

8E8F0958-3B79-591C-BC64-9C590DF83E6D

#### Pusia ebenus

(Lamark, 1811)

B75C4CEE-ECEA-5169-9965-B8ABB036B94C

##### Materials

- **Type status:** Other material. **Occurrence:** occurrenceID: 200ADA67-E6BC-5A65-940E-E657F7D86F0E; **Location:** country: Italy; countryCode: IT; stateProvince: Sassari; locality: Island of Culuccia; verbatimLatitude: 41 12 58.16N; verbatimLongitude: 9 17 09.66E; geodeticDatum: WGS84
- **Type status:** Other material. **Occurrence:** occurrenceID: AC353E8E-C584-5D4E-937F-4520C35C8995; **Location:** country: Italy; countryCode: IT; stateProvince: Sassari; locality: Island of Culuccia; verbatimLatitude: 41 13 17.70N; verbatimLongitude: 9 17 21.41E; geodeticDatum: WGS84

##### Notes

Alive, Fig. 36.

#### Pusia granum

(Forbes, 1844)

35B5160E-858E-5450-ABED-81CB4C2BB43A

##### Materials

- **Type status:** Other material. **Occurrence:** occurrenceID: 3D5632AD-75CD-558D-970A-0C66B3C91E0B; **Location:** country: Italy; countryCode: IT; stateProvince: Sassari; locality: Island of Culuccia; verbatimLatitude: 41 13 17.70N; verbatimLongitude: 9 17 21.41E; geodeticDatum: WGS84

##### Notes

Shell, new record.

#### Pusia tricolor

(Gmelin, 1790)

7609A417-8C76-5289-984B-F8213AC33B58

##### Materials

- **Type status:** Other material. **Occurrence:** occurrenceID: 3754D987-6AB4-5EE3-89C6-AF3AF6309A8C; **Location:** country: Italy; countryCode: IT; stateProvince: Sassari; locality: Island of Culuccia; verbatimLatitude: 41 13 17.70N; verbatimLongitude: 9 17 21.41E; geodeticDatum: WGS84

##### Notes

Shell.

#### Chauvetiidae

529ED08A-4239-5632-A3D3-EAC824706C82

#### Chauvetia mamillata

(Risso, 1826)

9F5D3080-2261-5177-B1DF-FB3E205EB27E

##### Materials

- **Type status:** Other material. **Occurrence:** occurrenceID: 0F683C6A-C2B5-5B1C-824D-4D7434A1AD9B; **Location:** country: Italy; countryCode: IT; stateProvince: Sassari; locality: Island of Culuccia; verbatimLatitude: 41 13 17.70N; verbatimLongitude: 9 17 21.41E; geodeticDatum: WGS84

##### Notes

Shell.

#### Chauvetia turritellata

(Deshayes, 1835)

D21121E4-9989-5C4F-B1E5-EBD9A40083C5

##### Materials

- **Type status:** Other material. **Occurrence:** occurrenceID: 3953AB13-8194-5040-8AEE-DD28DBDD2F50; **Location:** country: Italy; countryCode: IT; stateProvince: Sassari; locality: Island of Culuccia; verbatimLatitude: 41 13 17.70N; verbatimLongitude: 9 17 21.41E; geodeticDatum: WGS84

##### Notes

Shell, Fig. 37.

#### Pisaniidae

FB1A26D8-FB17-56DF-870C-28BCC504D741

#### Pisania striata

(Gmelin, 1791)

9864CAFC-48D6-5FF0-8AB2-3A4D3EE7CF1F

##### Materials

- **Type status:** Other material. **Occurrence:** occurrenceID: C5580C8A-16F9-5481-8A3F-550095346A78; **Location:** country: Italy; countryCode: IT; stateProvince: Sassari; locality: Island of Culuccia; verbatimLatitude: 41 12 51.58N; verbatimLongitude: 9 17 31.46E; geodeticDatum: WGS84
- **Type status:** Other material. **Occurrence:** occurrenceID: DBE04CF7-E085-550D-B5EF-A7030AC5C64C; **Location:** country: Italy; countryCode: IT; stateProvince: Sassari; locality: Island of Culuccia; verbatimLatitude: 41 12 13.88N; verbatimLongitude: 9 17 56.14E; geodeticDatum: WGS84
- **Type status:** Other material. **Occurrence:** occurrenceID: F6AAB745-98CC-5D62-8501-018AC3660FEA; **Location:** country: Italy; countryCode: IT; stateProvince: Sassari; locality: Island of Culuccia; verbatimLatitude: 41 12 28.07N; verbatimLongitude: 9 17 47.62E; geodeticDatum: WGS84
- **Type status:** Other material. **Occurrence:** occurrenceID: CF1966AD-512E-596C-83B7-F81F7FFDD407; **Location:** country: Italy; countryCode: IT; stateProvince: Sassari; locality: Island of Culuccia; verbatimLatitude: 41 12 42.40N; verbatimLongitude: 9 17 45.35E; geodeticDatum: WGS84
- **Type status:** Other material. **Occurrence:** occurrenceID: 7C28CF2C-DC84-51D0-B596-3975AEE9A217; **Location:** country: Italy; countryCode: IT; stateProvince: Sassari; locality: Island of Culuccia; verbatimLatitude: 41 12 47.63N; verbatimLongitude: 9 17 08.68E; geodeticDatum: WGS84
- **Type status:** Other material. **Occurrence:** occurrenceID: 7C0BA602-2F1D-59A9-B059-49E7AF4C396A; **Location:** country: Italy; countryCode: IT; stateProvince: Sassari; locality: Island of Culuccia; verbatimLatitude: 41 13 17.70N; verbatimLongitude: 9 17 21.41E; geodeticDatum: WGS84

##### Notes

Alive, Fig. 38.

#### Aplus dorbigny

(Payraudeau, 1826)

FDE8B66C-759D-5C77-A3E1-A001D127D6DC

##### Materials

- **Type status:** Other material. **Occurrence:** occurrenceID: 69CC39A4-2064-53B2-8603-79FB49292A17; **Location:** country: Italy; countryCode: IT; stateProvince: Sassari; locality: Island of Culuccia; verbatimLatitude: 41 12 51.58N; verbatimLongitude: 9 17 31.46E; geodeticDatum: WGS84
- **Type status:** Other material. **Occurrence:** occurrenceID: 482D045F-2E20-5B45-B8A2-CB82FF1C1347; **Location:** country: Italy; countryCode: IT; stateProvince: Sassari; locality: Island of Culuccia; verbatimLatitude: 41 12 13.88N; verbatimLongitude: 9 17 56.14E; geodeticDatum: WGS84
- **Type status:** Other material. **Occurrence:** occurrenceID: 942E5066-C27C-5FBB-AD3F-325F7B81EA48; **Location:** country: Italy; countryCode: IT; stateProvince: Sassari; locality: Island of Culuccia; verbatimLatitude: 41 12 28.07N; verbatimLongitude: 9 17 47.62E; geodeticDatum: WGS84
- **Type status:** Other material. **Occurrence:** occurrenceID: A0B49045-E53C-58BC-94FC-1422B9593274; **Location:** country: Italy; countryCode: IT; stateProvince: Sassari; locality: Island of Culuccia; verbatimLatitude: 41 12 42.40N; verbatimLongitude: 9 17 45.35E; geodeticDatum: WGS84
- **Type status:** Other material. **Occurrence:** occurrenceID: 141B0CAB-5C6F-5513-AC7A-5CBDA047DFBD; **Location:** country: Italy; countryCode: IT; stateProvince: Sassari; locality: Island of Culuccia; verbatimLatitude: 41 12 47.63N; verbatimLongitude: 9 17 08.68E; geodeticDatum: WGS84
- **Type status:** Other material. **Occurrence:** occurrenceID: 9F4FC7AF-5719-5899-9042-59C0E92D8843; **Location:** country: Italy; countryCode: IT; stateProvince: Sassari; locality: Island of Culuccia; verbatimLatitude: 41 13 17.70N; verbatimLongitude: 9 17 21.41E; geodeticDatum: WGS84

##### Notes

Alive, Fig. 39.

#### Aplus scaber

(Locard, 1891)

BB730FAB-1815-5C17-BCE3-F15C22E8DCFE

##### Materials

- **Type status:** Other material. **Occurrence:** occurrenceID: 61A412E1-EAAC-5815-8FCA-4C44D87EF7C5; **Location:** country: Italy; countryCode: IT; stateProvince: Sassari; locality: Island of Culuccia; verbatimLatitude: 41 13 17.70N; verbatimLongitude: 9 17 21.41E; geodeticDatum: WGS84

##### Notes

Shell.

#### Nassariidae

E438CFF6-F1ED-5768-ADDA-33C09077CC23

#### Tritia corniculum

(Olivi, 1792)

3AA3804E-A857-5AFC-A773-34EC0BF1A436

##### Materials

- **Type status:** Other material. **Occurrence:** occurrenceID: FD9B4A9B-2A8B-5492-9944-A45B80783C62; **Location:** country: Italy; countryCode: IT; stateProvince: Sassari; locality: Island of Culuccia; verbatimLatitude: 41 11 22.14N; verbatimLongitude: 9 16 59.05E; geodeticDatum: WGS84

##### Notes

Alive, Fig. 40.

#### Tritia incrassata

(Strøm, 1768)

07AECB78-1C5B-597E-A6CA-E3B3FE75767F

##### Materials

- **Type status:** Other material. **Occurrence:** occurrenceID: CE52BC6B-8766-58C9-B109-8A76711B2EDD; **Location:** country: Italy; countryCode: IT; stateProvince: Sassari; locality: Island of Culuccia; verbatimLatitude: 41 12 58.68N; verbatimLongitude: 9 17 26.51E; geodeticDatum: WGS84
- **Type status:** Other material. **Occurrence:** occurrenceID: F32B981C-6EE9-5214-90CD-7559C9616F78; **Location:** country: Italy; countryCode: IT; stateProvince: Sassari; locality: Island of Culuccia; verbatimLatitude: 41 13 17.70N; verbatimLongitude: 9 17 21.41E; geodeticDatum: WGS84

##### Notes

Alive.

#### Tritia mutabilis

(Linnaeus, 1758)

1E473C42-5956-53F3-8E84-E171847D36D3

##### Materials

- **Type status:** Other material. **Occurrence:** occurrenceID: 363AD524-CF94-512D-A848-B8A0FA90C1EE; **Location:** country: Italy; countryCode: IT; stateProvince: Sassari; locality: Island of Culuccia; verbatimLatitude: 41 11 46.79N; verbatimLongitude: 9 17 20.34E; geodeticDatum: WGS84
- **Type status:** Other material. **Occurrence:** occurrenceID: 79059231-3CE8-54BE-B903-768840FE39AC; **Location:** country: Italy; countryCode: IT; stateProvince: Sassari; locality: Island of Culuccia; verbatimLatitude: 41 12 51.58N; verbatimLongitude: 9 17 31.46E; geodeticDatum: WGS84

##### Notes

Shell.

#### Tritia neritea

(Linnaeus, 1758)

AF699ECC-5D11-5DC7-9CBF-4A4EC05AE023

##### Materials

- **Type status:** Other material. **Occurrence:** occurrenceID: 26611D99-EBAB-5A3F-A7C0-32CC81A886AB; **Location:** country: Italy; countryCode: IT; stateProvince: Sassari; locality: Island of Culuccia; verbatimLatitude: 41 11 22.14N; verbatimLongitude: 9 16 59.05E; geodeticDatum: WGS84

##### Notes

Alive.

#### Tritia pellucida

(Risso, 1827)

EE56BEB7-1B1F-5FC1-8AFF-C3997A251315

##### Materials

- **Type status:** Other material. **Occurrence:** occurrenceID: C188067C-278F-5E19-93A3-92AB2AD3E7BF; **Location:** country: Italy; countryCode: IT; stateProvince: Sassari; locality: Island of Culuccia; verbatimLatitude: 41 11 46.79N; verbatimLongitude: 9 17 20.34E; geodeticDatum: WGS84
- **Type status:** Other material. **Occurrence:** occurrenceID: 81AF5D06-18B1-542D-A416-50EB9795B981; **Location:** country: Italy; countryCode: IT; stateProvince: Sassari; locality: Island of Culuccia; verbatimLatitude: 41 12 51.58N; verbatimLongitude: 9 17 31.46E; geodeticDatum: WGS84

##### Notes

Shell.

#### Tudiclidae

8348005D-C6D8-5E41-BE82-9A48510CA8BF

#### Euthria cornea

(Linnaeus, 1758)

7F223A0A-6EBB-5232-9C00-BABF6C674249

##### Materials

- **Type status:** Other material. **Occurrence:** occurrenceID: 58F0D1DA-0104-570E-8CE7-89953315C882; **Location:** country: Italy; countryCode: IT; stateProvince: Sassari; locality: Island of Culuccia; verbatimLatitude: 41 11 56.04N; verbatimLongitude: 9 17 33.63E; geodeticDatum: WGS84
- **Type status:** Other material. **Occurrence:** occurrenceID: 34A585AA-9814-5B53-876B-DBEBBD1EA3FD; **Location:** country: Italy; countryCode: IT; stateProvince: Sassari; locality: Island of Culuccia; verbatimLatitude: 41 12 58.16N; verbatimLongitude: 9 17 09.66E; geodeticDatum: WGS84

##### Notes

Alive, Fig. 41.

#### Columbellidae

28D8105A-7AC8-5765-8A60-51CC83B48573

#### Columbella rustica

(Linnaeus, 1758)

2247C291-69D9-54FF-896B-F61FFB045D21

##### Materials

- **Type status:** Other material. **Occurrence:** occurrenceID: DE4A7610-6D9F-5E4F-BCF3-FAD40C75C164; **Location:** country: Italy; countryCode: IT; stateProvince: Sassari; locality: Island of Culuccia; verbatimLatitude: 41 11 46.79N; verbatimLongitude: 9 17 20.34E; geodeticDatum: WGS84
- **Type status:** Other material. **Occurrence:** occurrenceID: 8C57FCF3-A5A8-5584-AAA5-631C541D43AD; **Location:** country: Italy; countryCode: IT; stateProvince: Sassari; locality: Island of Culuccia; verbatimLatitude: 41 12 51.58N; verbatimLongitude: 9 17 31.46E; geodeticDatum: WGS84
- **Type status:** Other material. **Occurrence:** occurrenceID: 12F3A7C7-C991-537E-A9AF-C096C46D6A7C; **Location:** country: Italy; countryCode: IT; stateProvince: Sassari; locality: Island of Culuccia; verbatimLatitude: 41 12 13.88N; verbatimLongitude: 9 17 56.14E; geodeticDatum: WGS84
- **Type status:** Other material. **Occurrence:** occurrenceID: E0A06E41-D821-55C0-8CF9-2D9BD6A213F4; **Location:** country: Italy; countryCode: IT; stateProvince: Sassari; locality: Island of Culuccia; verbatimLatitude: 41 12 28.07N; verbatimLongitude: 9 17 47.62E; geodeticDatum: WGS84
- **Type status:** Other material. **Occurrence:** occurrenceID: CF970F02-F38A-5F9D-ADD3-7984748A18E6; **Location:** country: Italy; countryCode: IT; stateProvince: Sassari; locality: Island of Culuccia; verbatimLatitude: 41 13 17.70N; verbatimLongitude: 9 17 21.41E; geodeticDatum: WGS84

##### Notes

Alive, Fig. 42.

#### Mitridae

09789E2A-B1E6-54B1-88ED-AE746A12830F

#### Episcomitra cornicula

(Linnaeus, 1758)

80C8A16D-F655-585F-8405-C7E90533F1B7

##### Materials

- **Type status:** Other material. **Occurrence:** occurrenceID: 9B3CA709-0A3C-578A-B08C-8E0B2552A5C1; **Location:** country: Italy; countryCode: IT; stateProvince: Sassari; locality: Island of Culuccia; verbatimLatitude: 41 12 13.88N; verbatimLongitude: 9 17 56.14E; geodeticDatum: WGS84
- **Type status:** Other material. **Occurrence:** occurrenceID: 48CB5E14-8CD9-5ED1-B4A5-28BD69234453; **Location:** country: Italy; countryCode: IT; stateProvince: Sassari; locality: Island of Culuccia; verbatimLatitude: 41 12 28.07N; verbatimLongitude: 9 17 47.62E; geodeticDatum: WGS84

##### Notes

Alive, Fig. 43.

#### Fasciolariide

4766E131-5DBD-5BAB-AFC1-B85E3A05D207

#### Tarantinaea lignaria

(Linnaeus, 1758)

247986F4-799E-5531-82B8-A33BE686390C

##### Materials

- **Type status:** Other material. **Occurrence:** occurrenceID: BDABE2A6-23B3-5D08-8E13-0EE538DAF577; **Location:** country: Italy; countryCode: IT; stateProvince: Sassari; locality: Island of Culuccia; verbatimLatitude: 41 12 28.07N; verbatimLongitude: 9 17 47.62E; geodeticDatum: WGS84
- **Type status:** Other material. **Occurrence:** occurrenceID: 0B521C0F-E74F-54D4-A7B4-6CB020173024; **Location:** country: Italy; countryCode: IT; stateProvince: Sassari; locality: Island of Culuccia; verbatimLatitude: 41 12 47.63N; verbatimLongitude: 9 17 08.68E; geodeticDatum: WGS84
- **Type status:** Other material. **Occurrence:** occurrenceID: A917D22F-5C9F-524A-90B5-578E0961C075; **Location:** country: Italy; countryCode: IT; stateProvince: Sassari; locality: Island of Culuccia; verbatimLatitude: 41 12 09.61N; verbatimLongitude: 9 16 43.24E; geodeticDatum: WGS84

##### Notes

Alive, Fig. 44.

#### Pseudofusus pulchellus

(Philippi, 1844)

7BA7F0B0-1278-5B25-8BA3-083B3CE1E2D3

##### Materials

- **Type status:** Other material. **Occurrence:** occurrenceID: B54E0CC5-BF97-59BB-8263-93C58FF6A5D9; **Location:** country: Italy; countryCode: IT; stateProvince: Sassari; locality: Island of Culuccia; verbatimLatitude: 41 12 28.07N; verbatimLongitude: 9 17 47.62E; geodeticDatum: WGS84
- **Type status:** Other material. **Occurrence:** occurrenceID: EF6D6085-E202-5500-9235-27D667196A1F; **Location:** country: Italy; countryCode: IT; stateProvince: Sassari; locality: Island of Culuccia; verbatimLatitude: 41 13 17.70N; verbatimLongitude: 9 17 21.41E; geodeticDatum: WGS84

##### Notes

Shell.

#### Aptyxis syracusana

(Linnaeus, 1758)

2A24F613-9C23-5859-AD26-4E1EA4105EC8

##### Materials

- **Type status:** Other material. **Occurrence:** occurrenceID: 529D02CD-B748-5A86-953B-CFF44FB8FC5F; **Location:** country: Italy; countryCode: IT; stateProvince: Sassari; locality: Island of Culuccia; verbatimLatitude: 41 12 28.07N; verbatimLongitude: 9 17 47.62E; geodeticDatum: WGS84

##### Notes

Shell.

#### Columbellidae

72BBBE5C-9AAC-5E51-A11B-DB231712B5A9

#### Mitrella minor

(Scacchi, 1836)

23794D57-0CE7-5FEA-B3FA-B7BBDFFC77E2

##### Materials

- **Type status:** Other material. **Occurrence:** occurrenceID: C4AF053E-8BB6-5465-A666-EA10550E67C6; **Location:** country: Italy; countryCode: IT; stateProvince: Sassari; locality: Island of Culuccia; verbatimLatitude: 41 13 17.70N; verbatimLongitude: 9 17 21.41E; geodeticDatum: WGS84

##### Notes

Shell.

#### Mitrella scripta

(Linnaeus, 1758)

838FB270-A043-5F03-AE58-C318E64EF562

##### Materials

- **Type status:** Other material. **Occurrence:** occurrenceID: 70E9B502-DA39-5B05-B091-65900C6262CD; **Location:** country: Italy; countryCode: IT; stateProvince: Sassari; locality: Island of Culuccia; verbatimLatitude: 41 13 17.70N; verbatimLongitude: 9 17 21.41E; geodeticDatum: WGS84

##### Notes

Shell.

#### Horaiclavidae

28265985-5CAD-5F57-A7A9-66484FD9383A

#### Haedropleura septangularis

(Montagu, 1803)

7AB6AD8E-BA8E-510C-B78D-413373D3C1B9

##### Materials

- **Type status:** Other material. **Occurrence:** occurrenceID: 3F3F8CAD-F880-596F-8C59-940A730BAD11; **Location:** country: Italy; countryCode: IT; stateProvince: Sassari; locality: Island of Culuccia; verbatimLatitude: 41 13 17.70N; verbatimLongitude: 9 17 21.41E; geodeticDatum: WGS84

##### Notes

Shell.

#### Mitromorphidae

5F47E664-2178-5298-A710-BD5FB23AF221

#### Mitromorpha columbellaria

(Scacchi, 1836)

14190262-8181-5B77-90A7-D3A75F5F7942

##### Materials

- **Type status:** Other material. **Occurrence:** occurrenceID: BCE3E3F2-FB9D-53EB-A9F7-8EE429D3878C; **Location:** country: Italy; countryCode: IT; stateProvince: Sassari; locality: Island of Culuccia; verbatimLatitude: 41 13 17.70N; verbatimLongitude: 9 17 21.41E; geodeticDatum: WGS84

##### Notes

Shell.

#### Conidae

B619EDC9-7399-5EE4-8EEC-AD3641EBE575

#### Conus ventricosus

Gmelin, 1791

B56CA7CA-18BF-5981-AB84-2AFCFC71829D

##### Materials

- **Type status:** Other material. **Occurrence:** occurrenceID: 0F067DAE-12E0-56E0-A7BB-C87F1CD29F62; **Location:** country: Italy; countryCode: IT; stateProvince: Sassari; locality: Island of Culuccia; verbatimLatitude: 41 11 56.04N; verbatimLongitude: 9 17 33.63E; geodeticDatum: WGS84

##### Notes

Alive, Fig. 45.

#### Raphitomidae

AAC3B3BD-0E7C-5004-A2DA-A9C94E7CE143

#### Cyrillia linearis

(Montagu, 1803)

1846D05C-DD64-5511-98DE-EFED39ACA4F0

##### Materials

- **Type status:** Other material. **Occurrence:** occurrenceID: 7A62C62C-764B-5A14-BF73-416391570B4B; **Location:** country: Italy; countryCode: IT; stateProvince: Sassari; locality: Island of Culuccia; verbatimLatitude: 41 13 17.70N; verbatimLongitude: 9 17 21.41E; geodeticDatum: WGS84

##### Notes

Alive.

#### Leufroyia concinna

(Scacchi, 1836)

BE666D03-6C35-5F65-9F15-2EA7A62D9F5E

##### Materials

- **Type status:** Other material. **Occurrence:** occurrenceID: 50A0AEC0-F98F-5810-B86F-567A64E6D382; **Location:** country: Italy; countryCode: IT; stateProvince: Sassari; locality: Island of Culuccia; verbatimLatitude: 41 13 17.70N; verbatimLongitude: 9 17 21.41E; geodeticDatum: WGS84

##### Notes

Shell.

#### Leufroyia leufroyi

(Michaud, 1828)

012A9C89-C328-5349-B0DF-62F221A28C68

##### Materials

- **Type status:** Other material. **Occurrence:** occurrenceID: F87FAF6A-C0FC-5ACF-830E-75C32787A4A7; **Location:** country: Italy; countryCode: IT; stateProvince: Sassari; locality: Island of Culuccia; verbatimLatitude: 41 13 17.70N; verbatimLongitude: 9 17 21.41E; geodeticDatum: WGS84

##### Notes

Shell.

#### Raphitoma bicolor

(Risso, 1826)

55A5DA77-2FDE-5ACC-A738-CA3C480A25A3

##### Materials

- **Type status:** Other material. **Occurrence:** occurrenceID: 0027DBCF-2E8B-58A2-9C5E-A28E4C9FB9EC; **Location:** country: Italy; countryCode: IT; stateProvince: Sassari; locality: Island of Culuccia; verbatimLatitude: 41 13 17.70N; verbatimLongitude: 9 17 21.41E; geodeticDatum: WGS84

##### Notes

Shell.

#### Raphitoma densa

(Monterosato, 1884)

690EF756-B661-5AF3-9A09-4B0481832636

##### Materials

- **Type status:** Other material. **Occurrence:** occurrenceID: 9293F068-698A-5BC4-8C98-283E1BF0D5FB; **Location:** country: Italy; countryCode: IT; stateProvince: Sassari; locality: Island of Culuccia; verbatimLatitude: 41 13 17.70N; verbatimLongitude: 9 17 21.41E; geodeticDatum: WGS84

##### Notes

Shell.

#### Raphitoma horrida

(Monterosato, 1884)

29315FE4-3B1E-5496-9614-DF7F60CE0098

##### Materials

- **Type status:** Other material. **Occurrence:** occurrenceID: 43775F99-DA85-54C0-B043-0738375B82CF; **Location:** country: Italy; countryCode: IT; stateProvince: Sassari; locality: Island of Culuccia; verbatimLatitude: 41 13 17.70N; verbatimLongitude: 9 17 21.41E; geodeticDatum: WGS84

##### Notes

Shell.

#### Raphitoma lineolata

(Bucquoy, Dautzenberg & Dollfus, 1883)

EC4F443E-D9A5-5877-BB96-808F560D6BC1

##### Materials

- **Type status:** Other material. **Occurrence:** occurrenceID: 3D6A094C-94A0-5471-888F-78DF996212F2; **Location:** country: Italy; countryCode: IT; stateProvince: Sassari; locality: Island of Culuccia; verbatimLatitude: 41 13 17.70N; verbatimLongitude: 9 17 21.41E; geodeticDatum: WGS84

##### Notes

Shell.

#### Raphitoma locardi

Pusateri, Giannuzzi-Savelli & Oliverio, 2013

1B4660A5-7D2A-52CA-968B-16E08EC08A44

##### Materials

- **Type status:** Other material. **Occurrence:** occurrenceID: ED947A67-7D08-5CE9-9DD2-EEC1DBE2A4EB; **Location:** country: Italy; countryCode: IT; stateProvince: Sassari; locality: Island of Culuccia; verbatimLatitude: 41 13 17.70N; verbatimLongitude: 9 17 21.41E; geodeticDatum: WGS84

##### Notes

Shell.

#### Raphitoma philberti

(Michaud, 1829)

B6F5D5E6-BE62-5284-9400-3059C72360E6

##### Materials

- **Type status:** Other material. **Occurrence:** occurrenceID: 92313D41-6111-5983-AF35-1C7FA295BCE8; **Location:** country: Italy; countryCode: IT; stateProvince: Sassari; locality: Island of Culuccia; verbatimLatitude: 41 13 17.70N; verbatimLongitude: 9 17 21.41E; geodeticDatum: WGS84

##### Notes

Shell.

#### Mangeliidae

0449F24A-33E6-559F-9BF0-018F83E209F7

#### Mangelia multilineolata

(Deshayes, 1835)

EEEFED36-3F52-53C7-9BC9-60CD035B25AD

##### Materials

- **Type status:** Other material. **Occurrence:** occurrenceID: 82791F31-A6AC-5612-9ADE-501A2664792D; **Location:** country: Italy; countryCode: IT; stateProvince: Sassari; locality: Island of Culuccia; verbatimLatitude: 41 13 17.70N; verbatimLongitude: 9 17 21.41E; geodeticDatum: WGS84

##### Notes

Shell.

#### Mangelia stosiciana

Brusina, 1869

03935DBA-A63E-5D6D-8E35-5E66890870A4

##### Materials

- **Type status:** Other material. **Occurrence:** occurrenceID: 8F39F862-26B7-5EFE-AB9B-48F4FE03894F; **Location:** country: Italy; countryCode: IT; stateProvince: Sassari; locality: Island of Culuccia; verbatimLatitude: 41 13 17.70N; verbatimLongitude: 9 17 21.41E; geodeticDatum: WGS84

##### Notes

Shell.

#### Mangelia taeniata

(Deshayes, 1835)

F2046027-85E7-556A-AF0B-45C4B66D14B9

##### Materials

- **Type status:** Other material. **Occurrence:** occurrenceID: AAF2EA95-E840-5ABD-895D-91E5355E8EAC; **Location:** country: Italy; countryCode: IT; stateProvince: Sassari; locality: Island of Culuccia; verbatimLatitude: 41 13 17.70N; verbatimLongitude: 9 17 21.41E; geodeticDatum: WGS84

##### Notes

Shell.

#### Mangelia unifasciata

(Deshayes, 1835)

6370B396-0033-54CB-B353-896DD7072A51

##### Materials

- **Type status:** Other material. **Occurrence:** occurrenceID: E431620E-419F-54B5-A27B-A33F2C3C81CF; **Location:** country: Italy; countryCode: IT; stateProvince: Sassari; locality: Island of Culuccia; verbatimLatitude: 41 13 17.70N; verbatimLongitude: 9 17 21.41E; geodeticDatum: WGS84

##### Notes

Shell.

#### Mangelia vauquelini

(Payraudeau, 1826)

5E957C0B-A723-56B4-A848-995110967B80

##### Materials

- **Type status:** Other material. **Occurrence:** occurrenceID: BF47856C-CB94-5B7D-AF98-BE95624740E1; **Location:** country: Italy; countryCode: IT; stateProvince: Sassari; locality: Island of Culuccia; verbatimLatitude: 41 13 17.70N; verbatimLongitude: 9 17 21.41E; geodeticDatum: WGS84

##### Notes

Shell.

#### Pleurobranchida

5F5D027F-CABA-57DE-A062-6D2DB55E9DEA

#### Pleurobranchidae

243B9028-EEE1-5F24-B816-CF078AB52A63

#### Berthella plumula

(Montagu, 1803)

166694DB-B514-5363-9F59-E0173B6A8A19

##### Materials

- **Type status:** Other material. **Occurrence:** occurrenceID: 38692D0D-99C8-5E55-B535-FA85A595C237; **Location:** country: Italy; countryCode: IT; stateProvince: Sassari; locality: Island of Culuccia; verbatimLatitude: 41 11 46.14N; verbatimLongitude: 9 16 40.60E; geodeticDatum: WGS84

##### Notes

Alive, Fig. 46; new record.

#### Berthella aurantiaca

(Risso, 1818)

DDEA3984-DAC1-5AB9-8D0A-D4401F47D6CD

##### Materials

- **Type status:** Other material. **Occurrence:** occurrenceID: 4F340B13-6BCC-59BB-8497-A5D1475BC5E4; **Location:** country: Italy; countryCode: IT; stateProvince: Sassari; locality: Island of Culuccia; verbatimLatitude: 41 12 28.07N; verbatimLongitude: 9 17 47.62E; geodeticDatum: WGS84
- **Type status:** Other material. **Occurrence:** occurrenceID: 1981575E-790E-54C8-83D6-13463FE18071; **Location:** country: Italy; countryCode: IT; stateProvince: Sassari; locality: Island of Culuccia; verbatimLatitude: 41 12 58.16N; verbatimLongitude: 9 17 09.66E; geodeticDatum: WGS84
- **Type status:** Other material. **Occurrence:** occurrenceID: D865CCAD-BB21-5E30-B35F-4E9329B250CD; **Location:** country: Italy; countryCode: IT; stateProvince: Sassari; locality: Island of Culuccia; verbatimLatitude: 41 12 47.63N; verbatimLongitude: 9 17 08.68E; geodeticDatum: WGS84
- **Type status:** Other material. **Occurrence:** occurrenceID: FA7C1DB9-41EE-5E5B-816F-F56BFB5C9409; **Location:** country: Italy; countryCode: IT; stateProvince: Sassari; locality: Island of Culuccia; verbatimLatitude: 41 13 17.70N; verbatimLongitude: 9 17 21.41E; geodeticDatum: WGS84
- **Type status:** Other material. **Occurrence:** occurrenceID: E4469AD3-83B7-5675-8AA2-5309784F9EFA; **Location:** country: Italy; countryCode: IT; stateProvince: Sassari; locality: Island of Culuccia; verbatimLatitude: 41 12 09.61N; verbatimLongitude: 9 16 43.24E; geodeticDatum: WGS84

##### Notes

Alive, Fig. 47; new record.

#### Nudibranchia

98B5A617-5222-5BDD-99FC-8876B8A59A58

#### Chromodorididae

6CD52026-F0FE-5FC4-8FB9-370D84534410

#### Felimida elegantula

(R. A. Philippi, 1844)

119E53D3-4F8A-57DF-8ED4-EDC8930B3E9A

##### Materials

- **Type status:** Other material. **Occurrence:** occurrenceID: 8CB9B21D-D354-57BC-AB1A-68E8B38392C0; **Location:** country: Italy; countryCode: IT; stateProvince: Sassari; locality: Island of Culuccia; verbatimLatitude: 41 12 09.61N; verbatimLongitude: 9 16 43.24E; geodeticDatum: WGS84

##### Notes

Alive.

#### Felimida krohni

(Vérany, 1846)

E42F14C3-4A5A-59B7-8E09-CF3273EF8188

##### Materials

- **Type status:** Other material. **Occurrence:** occurrenceID: 79EE8F72-5FC3-5FE5-8755-35296BB2A323; **Location:** country: Italy; countryCode: IT; stateProvince: Sassari; locality: Island of Culuccia; verbatimLatitude: 41 11 28.32N; verbatimLongitude: 9 16 44.50E; geodeticDatum: WGS84

##### Notes

Alive, Fig. 48.

#### Felimida luteorosea

(Rapp, 1827)

9E4EAAF3-7175-559C-8804-B36CD8EF0373

##### Materials

- **Type status:** Other material. **Occurrence:** occurrenceID: 94A9E1FC-EB1A-5FB4-A2CC-4FA9D042CB69; **Location:** country: Italy; countryCode: IT; stateProvince: Sassari; locality: Island of Culuccia; verbatimLatitude: 41 11 46.79N; verbatimLongitude: 9 17 20.34E; geodeticDatum: WGS84

##### Notes

Alive, Fig. 49.

#### Felimida purpurea

(Risso, 1831)

E097970B-C49B-5892-B9B0-CB4C2EDD32E8

##### Materials

- **Type status:** Other material. **Occurrence:** occurrenceID: D41ACB64-8C1E-5AAB-8CEC-51707609DA01; **Location:** country: Italy; countryCode: IT; stateProvince: Sassari; locality: Island of Culuccia; verbatimLatitude: 41 12 42.40N; verbatimLongitude: 9 17 45.35E; geodeticDatum: WGS84

##### Notes

Alive, Fig. 50.

#### Felimare orsinii

(Vérany, 1846)

6340EC9D-D100-5FCE-9F13-263CAF9EA0A4

##### Materials

- **Type status:** Other material. **Occurrence:** occurrenceID: BFECFED0-3D7B-5A56-A9CB-A56D0A10A202; **Location:** country: Italy; countryCode: IT; stateProvince: Sassari; locality: Island of Culuccia; verbatimLatitude: 41 12 42.40N; verbatimLongitude: 9 17 45.35E; geodeticDatum: WGS84

##### Notes

Alive, Fig. 51.

#### Felimare picta

(Schultz in Philippi, 1836)

179EC414-5E6D-5040-B781-9B2DE014FCAF

##### Materials

- **Type status:** Other material. **Occurrence:** occurrenceID: 43052442-083B-5469-A70E-5E4B9A212ABF; **Location:** country: Italy; countryCode: IT; stateProvince: Sassari; locality: Island of Culuccia; verbatimLatitude: 41 11 46.79N; verbatimLongitude: 9 17 20.34E; geodeticDatum: WGS84

##### Notes

Alive, Fig. 52, Suppl. material 2.

#### Felimare villafranca

(Risso, 1818)

08C1ECE8-5235-53C0-A091-EF2DA42DC6DD

##### Materials

- **Type status:** Other material. **Occurrence:** occurrenceID: 44B38B1B-D65A-537D-AEB0-5AEEB26CFF85; **Location:** country: Italy; countryCode: IT; stateProvince: Sassari; locality: Island of Culuccia; verbatimLatitude: 41 12 42.40N; verbatimLongitude: 9 17 45.35E; geodeticDatum: WGS84

##### Notes

Alive, Fig. 53.

#### Phyllidiidae

55703C4F-6BF4-54F8-9F9E-EFB4F73B520D

#### Phyllidia flava

Aradas, 1847

ED3AB8A1-19D8-51CF-8D0A-6A27FB01D418

##### Materials

- **Type status:** Other material. **Occurrence:** occurrenceID: FE3959E9-4594-53A1-A12F-D98C20E64534; **Location:** country: Italy; countryCode: IT; stateProvince: Sassari; locality: Island of Culuccia; verbatimLatitude: 41 13 17.70N; verbatimLongitude: 9 17 21.41E; geodeticDatum: WGS84

##### Notes

Alive, Fig. 54.

#### Dendrodorididae

885580F0-E565-55D7-BA3C-6AA253C71CD2

#### Dendrodoris grandiflora

(Rapp, 1827)

F692E36A-8B5B-5FC1-9353-B7130345E982

##### Materials

- **Type status:** Other material. **Occurrence:** occurrenceID: C651957D-0F8B-5190-9FDC-740C609D335C; **Location:** country: Italy; countryCode: IT; stateProvince: Sassari; locality: Island of Culuccia; verbatimLatitude: 41 13 17.70N; verbatimLongitude: 9 17 21.41E; geodeticDatum: WGS84

##### Notes

Alive, Fig. 55.

#### Dendrodoris limbata

(Cuvier, 1804)

B8A5A45D-D644-5203-8C06-C93CA3B29689

##### Materials

- **Type status:** Other material. **Occurrence:** occurrenceID: 66914B6A-E7EA-50FE-ADA4-4F411080929A; **Location:** country: Italy; countryCode: IT; stateProvince: Sassari; locality: Island of Culuccia; verbatimLatitude: 41 13 17.70N; verbatimLongitude: 9 17 21.41E; geodeticDatum: WGS84
- **Type status:** Other material. **Occurrence:** occurrenceID: E2D82273-51FE-5914-BA9C-376101CA784E; **Location:** country: Italy; countryCode: IT; stateProvince: Sassari; locality: Island of Culuccia; verbatimLatitude: 41 12 47.63N; verbatimLongitude: 9 17 08.68E; geodeticDatum: WGS84

##### Notes

Alive, Fig. 56.

#### Discodorididae

EE9F51EB-5A42-5A0A-BED0-4A09F096A5A6

#### Taringa armata

Swennen, 1961

E3158A8D-A07F-5432-B702-781AAE24AB70

##### Materials

- **Type status:** Other material. **Occurrence:** occurrenceID: DD1E7A6A-986E-5A31-8117-754B5B491214; **Location:** country: Italy; countryCode: IT; stateProvince: Sassari; locality: Island of Culuccia; verbatimLatitude: 41 12 28.07N; verbatimLongitude: 9 17 47.62E; geodeticDatum: WGS84

##### Notes

Alive, Fig. 57.

#### Janolidae

05614FD9-73DD-507D-8ACE-48C002167E8B

#### Antiopella cristata

(Delle Chiaje, 1841)

67FCCD71-26F1-5782-8C2A-F7B7C7B3A143

##### Materials

- **Type status:** Other material. **Occurrence:** occurrenceID: 724F710D-1E5A-5F9C-BBCA-787B3A2DC4FC; **Location:** country: Italy; countryCode: IT; stateProvince: Sassari; locality: Island of Culuccia; verbatimLatitude: 41 13 17.70N; verbatimLongitude: 9 17 21.41E; geodeticDatum: WGS84

##### Notes

Alive, Fig. 58.

#### Aeolidiidae

3D53FE11-DF8A-52B6-9391-C849A6913FF4

#### Aeolidiella alderi

(Cocks, 1852)

F3663914-314B-5660-8786-011008BD3800

##### Materials

- **Type status:** Other material. **Occurrence:** occurrenceID: 17841E95-C2C6-5A23-ADA0-AC7896FAC7A4; **Location:** country: Italy; countryCode: IT; stateProvince: Sassari; locality: Island of Culuccia; verbatimLatitude: 41 12 09.61N; verbatimLongitude: 9 16 43.24E; geodeticDatum: WGS84
- **Type status:** Other material. **Occurrence:** occurrenceID: C54BACF7-CBA7-510A-B685-BBB2CA664DC3; **Location:** country: Italy; countryCode: IT; stateProvince: Sassari; locality: Island of Culuccia; verbatimLatitude: 41 11 28.32N; verbatimLongitude: 9 16 44.50E; geodeticDatum: WGS84

##### Notes

Alive, Fig. 59.

#### Spurilla neapolitana

(Delle Chiaje, 1841)

39964897-E577-51A5-8185-A8EB0A3FC47B

##### Materials

- **Type status:** Other material. **Occurrence:** occurrenceID: 36243429-895D-577D-A3BB-4E7512DA2A3D; **Location:** country: Italy; countryCode: IT; stateProvince: Sassari; locality: Island of Culuccia; verbatimLatitude: 41 11 46.79N; verbatimLongitude: 9 17 20.34E; geodeticDatum: WGS84
- **Type status:** Other material. **Occurrence:** occurrenceID: 4AD50BB0-63A1-514B-B2EC-C0C85019641D; **Location:** country: Italy; countryCode: IT; stateProvince: Sassari; locality: Island of Culuccia; verbatimLatitude: 41 12 58.16N; verbatimLongitude: 9 17 09.66E; geodeticDatum: WGS84
- **Type status:** Other material. **Occurrence:** occurrenceID: CA7CD2DE-90A1-537B-BEDA-35D61C8EA2B7; **Location:** country: Italy; countryCode: IT; stateProvince: Sassari; locality: Island of Culuccia; verbatimLatitude: 41 12 09.61N; verbatimLongitude: 9 16 43.24E; geodeticDatum: WGS84

##### Notes

Alive, Fig. 60.

#### Facelinidae

17D0EB95-7F79-534E-91B9-4EFA6BBBE7A0

#### Cratena peregrina

(Gmelin, 1791)

4F0BC19B-4C4C-5B41-9A89-ED8B8192CD5A

##### Materials

- **Type status:** Other material. **Occurrence:** occurrenceID: 44D399F6-85CC-508B-BBF6-523E93245C71; **Location:** country: Italy; countryCode: IT; stateProvince: Sassari; locality: Island of Culuccia; verbatimLatitude: 41 11 46.79N; verbatimLongitude: 9 17 20.34E; geodeticDatum: WGS84
- **Type status:** Other material. **Occurrence:** occurrenceID: 7DCE5ADF-0C87-5978-A6E7-3A5DCED38914; **Location:** country: Italy; countryCode: IT; stateProvince: Sassari; locality: Island of Culuccia; verbatimLatitude: 41 11 46.14N; verbatimLongitude: 9 16 40.60E; geodeticDatum: WGS84

##### Notes

Alive, Fig. 61, Suppl. material 3.

#### Umbraculida

EF4260C2-092F-524F-AB8E-71A354FE6DE8

#### Tylodinidae

160A5CA3-AE8D-58FD-A364-3838E2B3137C

#### Tylodina perversa

(Gmelin, 1791)

5F2915E6-6DF1-5C05-9AB2-A5BC5EACC573

##### Materials

- **Type status:** Other material. **Occurrence:** occurrenceID: D1807523-13FB-5DF2-9BD5-984F03B2739F; **Location:** country: Italy; countryCode: IT; stateProvince: Sassari; locality: Island of Culuccia; verbatimLatitude: 41 13 17.70N; verbatimLongitude: 9 17 21.41E; geodeticDatum: WGS84

##### Notes

Shell.

#### Umbraculidae

5B19B5EA-83ED-57D2-B363-CD2052300568

#### Umbraculum umbraculum

([Lighfoot], 1786)

E6F40E36-F398-5543-8485-39AAE1FA5934

##### Materials

- **Type status:** Other material. **Occurrence:** occurrenceID: AD4493E1-28BE-5E6C-A0F5-51BA01EF8F28; **Location:** country: Italy; countryCode: IT; stateProvince: Sassari; locality: Island of Culuccia; verbatimLatitude: 41 12 47.63N; verbatimLongitude: 9 17 08.68E; geodeticDatum: WGS84

##### Notes

Alive, Fig. 62.

#### Cephalaspidea

49659981-4463-530C-9F13-79EA290C256B

#### Cylichnidae

F53996E3-EC88-524F-9660-9C6BE9D2A521

#### Cylichna cylindracea

(Pennant, 1777)

8D578DED-13E4-56E7-89B1-B136979CF813

##### Materials

- **Type status:** Other material. **Occurrence:** occurrenceID: 34725ED8-3E5C-5F8E-A522-EFF1F98998E3; **Location:** country: Italy; countryCode: IT; stateProvince: Sassari; locality: Island of Culuccia; verbatimLatitude: 41 13 17.70N; verbatimLongitude: 9 17 21.41E; geodeticDatum: WGS84

##### Notes

Shell.

#### Haminoeidae

C4F5BA09-3C1F-52C9-868F-3AB9F3997453

#### Haminoea hydatis

(Linnaeus, 1758)

904A5F06-177F-5DE3-A736-4595D5FA107D

##### Materials

- **Type status:** Other material. **Occurrence:** occurrenceID: B2698777-AA15-54D7-AD76-F8A1B877E18E; **Location:** country: Italy; countryCode: IT; stateProvince: Sassari; locality: Island of Culuccia; verbatimLatitude: 41 12 58.16N; verbatimLongitude: 9 17 09.66E; geodeticDatum: WGS84

##### Notes

Shell.

#### Lamprohaminoea ovalis

C46EC64D-43CE-5E20-A2F3-BDFFE04C960C

##### Materials

- **Type status:** Other material. **Occurrence:** occurrenceID: DCC99636-82F2-57F3-9F29-FF8BB63161A2; **Location:** country: Italy; countryCode: IT; stateProvince: Sassari; locality: Island of Culuccia; verbatimLatitude: 41 12 58.16N; verbatimLongitude: 9 17 09.66E; geodeticDatum: WGS84

##### Notes

Alive, Fig. 63.

#### Philinidae

3543B6C8-5171-549F-ACF1-2DAE9E298018

#### Philine catena

(Montagu, 1803)

209C60E0-D491-512C-87CF-E68B8365A67C

##### Materials

- **Type status:** Other material. **Occurrence:** occurrenceID: D8121C0D-8C12-57FB-9411-8DDB1EAE6966; **Location:** country: Italy; countryCode: IT; stateProvince: Sassari; locality: Island of Culuccia; verbatimLatitude: 41 13 17.70N; verbatimLongitude: 9 17 21.41E; geodeticDatum: WGS84

##### Notes

Shell.

#### Aplysiida

D7E6007B-B0A4-5463-A944-09825DF446B6

#### Aplysidae

F7BE8F19-7F9F-560F-85B4-FBB93EDD37BF

#### Bursatella leachii

Blainville, 1817

F80C5AF3-0F3B-5FCD-82AD-395AC33C444A

##### Materials

- **Type status:** Other material. **Occurrence:** occurrenceID: 431F8C1B-6268-5097-9688-63A50311A12C; **Location:** country: Italy; countryCode: IT; stateProvince: Sassari; locality: Island of Culuccia; verbatimLatitude: 41 12 09.61N; verbatimLongitude: 9 16 43.24E; geodeticDatum: WGS84

##### Notes

Alive, Fig. 64, Suppl. material 4; Non-indigenous species.

#### Aplysia depilans

Gmelin, 1791

091CC573-922F-527D-9DF9-3BFB2A7287ED

##### Materials

- **Type status:** Other material. **Occurrence:** occurrenceID: 76CAF1DF-7737-5489-9B63-264D1DE6191F; **Location:** country: Italy; countryCode: IT; stateProvince: Sassari; locality: Island of Culuccia; verbatimLatitude: 41 12 09.61N; verbatimLongitude: 9 16 43.24E; geodeticDatum: WGS84

##### Notes

Alive, Fig. 65.

#### Petalifera petalifera

(Rang, 1828)

7B8EE005-83FB-55D3-905E-8D54E21DD2AF

##### Materials

- **Type status:** Other material. **Occurrence:** occurrenceID: B58A515A-FAA5-55F1-99B3-55EF8603D744; **Location:** country: Italy; countryCode: IT; stateProvince: Sassari; locality: Island of Culuccia; verbatimLatitude: 41 12 58.16N; verbatimLongitude: 9 17 09.66E; geodeticDatum: WGS84

##### Notes

Alive, Fig. 66, Suppl. material 5.

#### Sacoglossa

DAA725F8-64E7-528A-A80F-B9B53127F3EB

#### Plakobranchidae

99CDADD6-1177-5A84-8BD8-5FED332FAA8B

#### Elysia timida

(Risso, 1818)

28166246-2814-59FA-A47F-388BB1CC9AD9

##### Materials

- **Type status:** Other material. **Occurrence:** occurrenceID: 1A91C829-E277-56D6-BD6E-4CAAF351D244; **Location:** country: Italy; countryCode: IT; stateProvince: Sassari; locality: Island of Culuccia; verbatimLatitude: 41 12 51.58N; verbatimLongitude: 9 17 31.46E; geodeticDatum: WGS84
- **Type status:** Other material. **Occurrence:** occurrenceID: 5CF6A548-FA7A-57BF-8F31-EA7172C49591; **Location:** country: Italy; countryCode: IT; stateProvince: Sassari; locality: Island of Culuccia; verbatimLatitude: 41 12 28.07N; verbatimLongitude: 9 17 47.62E; geodeticDatum: WGS84
- **Type status:** Other material. **Occurrence:** occurrenceID: 5875A317-4537-5F51-BE86-01AE5D562363; **Location:** country: Italy; countryCode: IT; stateProvince: Sassari; locality: Island of Culuccia; verbatimLatitude: 41 12 09.61N; verbatimLongitude: 9 16 43.24E; geodeticDatum: WGS84
- **Type status:** Other material. **Occurrence:** occurrenceID: 1F9C6881-EBA9-5135-8D5B-978A7BF52B2B; **Location:** country: Italy; countryCode: IT; stateProvince: Sassari; locality: Island of Culuccia; verbatimLatitude: 41 11 46.14N; verbatimLongitude: 9 16 40.60E; geodeticDatum: WGS84

##### Notes

Alive, Fig. 67.

#### Thuridilla hopei

(Vérany, 1853)

55146DEC-4E4E-5787-BFDC-9D115F8D4521

##### Materials

- **Type status:** Other material. **Occurrence:** occurrenceID: 057DC48A-35DB-5AAA-8F48-BF49AAA4C646; **Location:** country: Italy; countryCode: IT; stateProvince: Sassari; locality: Island of Culuccia; verbatimLatitude: 41 12 51.58N; verbatimLongitude: 9 17 31.46E; geodeticDatum: WGS84
- **Type status:** Other material. **Occurrence:** occurrenceID: B2738112-53BA-5288-9D2C-55F29A45E93C; **Location:** country: Italy; countryCode: IT; stateProvince: Sassari; locality: Island of Culuccia; verbatimLatitude: 41 12 58.16N; verbatimLongitude: 9 17 09.66E; geodeticDatum: WGS84
- **Type status:** Other material. **Occurrence:** occurrenceID: 44A1D449-00ED-5A3E-AEAB-784728E28D99; **Location:** country: Italy; countryCode: IT; stateProvince: Sassari; locality: Island of Culuccia; verbatimLatitude: 41 12 09.61N; verbatimLongitude: 9 16 43.24E; geodeticDatum: WGS84

##### Notes

Alive, Fig. 68.

#### Siphonarida

929C13EF-3F55-5389-942A-8708C6C27D73

#### Siphonariidae

FDA6CA73-5E40-5AE8-86D1-A42A0CC7D4CA

##### Materials

- **Type status:** Other material. **Occurrence:** occurrenceID: 6402AF77-07FA-57A6-876D-D1B55F3302EF; **Location:** country: Italy; countryCode: IT; stateProvince: Sassari; locality: Island of Culuccia; verbatimLatitude: 41 13 17.70N; verbatimLongitude: 9 17 21.41E; geodeticDatum: WGS84

#### Williamia gussoni

(O. G. Costa, 1829)

48DAA188-8D37-56A7-A116-7A942255B28B

##### Notes

Shell.

#### Pylopulmonata

9816130E-07BC-5597-A1CE-1B57BF4A6CCF

#### Pyramidellidae

8CE066B6-4525-583F-A9E9-A062FDA45DFA

#### Euparthenia humboldti

(Risso, 1826)

6B4B5131-590C-5056-B7B6-F027F0C49891

##### Materials

- **Type status:** Other material. **Occurrence:** occurrenceID: D34D69A7-615D-59AA-8D8E-F40079612E97; **Location:** country: Italy; countryCode: IT; stateProvince: Sassari; locality: Island of Culuccia; verbatimLatitude: 41 13 17.70N; verbatimLongitude: 9 17 21.41E; geodeticDatum: WGS84

##### Notes

Shell.

#### Folinella excavata

(Philippi, 1836)

4D64E953-84F4-519E-9424-C0E774D1FF9C

##### Materials

- **Type status:** Other material. **Occurrence:** occurrenceID: A537A975-F8AA-59CF-A098-EC0C61CCC151; **Location:** country: Italy; countryCode: IT; stateProvince: Sassari; locality: Island of Culuccia; verbatimLatitude: 41 13 17.70N; verbatimLongitude: 9 17 21.41E; geodeticDatum: WGS84

##### Notes

Shell.

#### Parthenina alesii

Micali, Nofroni & Perna, 2012

17DD1E90-45DA-582D-97BA-C5DE1B0B9E3A

##### Materials

- **Type status:** Other material. **Occurrence:** occurrenceID: D9F33A9B-AD5D-59FE-891C-4D7562F1B632; **Location:** country: Italy; countryCode: IT; stateProvince: Sassari; locality: Island of Culuccia; verbatimLatitude: 41 13 17.70N; verbatimLongitude: 9 17 21.41E; geodeticDatum: WGS84

##### Notes

Shell; new record.

#### Parthenina angulosa

(Monterosato, 1889)

1C27A18B-6A08-5C50-89DF-FE9EC817AAC4

##### Materials

- **Type status:** Other material. **Occurrence:** occurrenceID: 7A5BEB1A-8B64-5DCD-A5EB-E8D35F9C26EF; **Location:** country: Italy; countryCode: IT; stateProvince: Sassari; locality: Island of Culuccia; verbatimLatitude: 41 13 17.70N; verbatimLongitude: 9 17 21.41E; geodeticDatum: WGS84

##### Notes

Shell.

#### Parthenina monozona

(Brusina, 1869)

0F0B8778-5E7A-5539-9243-EC0E450465C6

##### Materials

- **Type status:** Other material. **Occurrence:** occurrenceID: EF94EF40-B09B-5C58-929F-CD431418239D; **Location:** country: Italy; countryCode: IT; stateProvince: Sassari; locality: Island of Culuccia; verbatimLatitude: 41 13 17.70N; verbatimLongitude: 9 17 21.41E; geodeticDatum: WGS84

##### Notes

Shell.

#### Megastomia conoidea

(Brocchi, 1814)

9BA4D5F0-FF93-509F-9483-6BD20B1AB428

##### Materials

- **Type status:** Other material. **Occurrence:** occurrenceID: 2D0FEE63-8521-56B2-8D2B-AF64F7A1DFFA; **Location:** country: Italy; countryCode: IT; stateProvince: Sassari; locality: Island of Culuccia; verbatimLatitude: 41 13 17.70N; verbatimLongitude: 9 17 21.41E; geodeticDatum: WGS84

##### Notes

Shell, new record.

#### Turbonilla gradata

Bucquoy, Dautzenberg & Dollfus, 1883

6813587B-41CF-5F5E-8031-01EDDF78A56A

##### Materials

- **Type status:** Other material. **Occurrence:** occurrenceID: D8F0B235-EB39-5AA9-BC03-1C357A43E25A; **Location:** country: Italy; countryCode: IT; stateProvince: Sassari; locality: Island of Culuccia; verbatimLatitude: 41 13 17.70N; verbatimLongitude: 9 17 21.41E; geodeticDatum: WGS84

##### Notes

Shell.

#### Turbonilla jeffreysii

(Forbes & Hanley, 1850)

E8E41C9E-345F-5CB0-8DA4-E6D1CB35C9CC

##### Materials

- **Type status:** Other material. **Occurrence:** occurrenceID: 854985A2-6FFF-5D51-B998-8CAD4F62DFC1; **Location:** country: Italy; countryCode: IT; stateProvince: Sassari; locality: Island of Culuccia; verbatimLatitude: 41 13 17.70N; verbatimLongitude: 9 17 21.41E; geodeticDatum: WGS84

##### Notes

Shell.

### Class Scaphopoda

#### Dentaliida

1FAA7C62-7B5B-5ADC-89A5-D0D7CE95DBAA

#### Dentaliidae

71CD67FE-1FE6-51A2-9106-0F511A2B60B7

#### Antalis vulgaris

(da Costa, 1778)

E4D52B2C-1B9C-5B79-B25A-BD81D0439C78

##### Materials

- **Type status:** Other material. **Occurrence:** occurrenceID: 3F3301A8-E7F0-58AB-91DD-23915507C679; **Location:** country: Italy; countryCode: IT; stateProvince: Sassari; locality: Island of Culuccia; verbatimLatitude: 41 13 17.70N; verbatimLongitude: 9 17 21.41E; geodeticDatum: WGS84

##### Notes

Alive, Fig. 69.

### Class Bivalvia

#### Arcida

D29859D9-B310-5D6E-BBEF-E60E003C0E7C

#### Arcidae

B7C97C62-B600-51F6-B6BC-4B2135F2C8EB

#### Arca noae

Linnaeus, 1758

B3A88123-9AE1-5771-AE4F-B27AC5DBA97A

##### Materials

- **Type status:** Other material. **Occurrence:** occurrenceID: 8F7CA50A-2A0D-5ABD-B209-17FDABD09A25; **Location:** country: Italy; countryCode: IT; stateProvince: Sassari; locality: Island of Culuccia; verbatimLatitude: 41 11 46.79N; verbatimLongitude: 9 17 20.34E; geodeticDatum: WGS84
- **Type status:** Other material. **Occurrence:** occurrenceID: 6BA5E27B-96E4-5C68-BB26-861CA467EBD2; **Location:** country: Italy; countryCode: IT; stateProvince: Sassari; locality: Island of Culuccia; verbatimLatitude: 41 11 56.04N; verbatimLongitude: 9 17 33.63E; geodeticDatum: WGS84
- **Type status:** Other material. **Occurrence:** occurrenceID: 5C0F4151-8CB2-57A9-A0E4-CCACA1F1A65C; **Location:** country: Italy; countryCode: IT; stateProvince: Sassari; locality: Island of Culuccia; verbatimLatitude: 41 13 17.70N; verbatimLongitude: 9 17 21.41E; geodeticDatum: WGS84
- **Type status:** Other material. **Occurrence:** occurrenceID: DAEFE6E8-2370-5B86-B2D1-933D21A3A8D8; **Location:** country: Italy; countryCode: IT; stateProvince: Sassari; locality: Island of Culuccia; verbatimLatitude: 41 12 09.61N; verbatimLongitude: 9 16 43.24E; geodeticDatum: WGS84

##### Notes

Alive, Fig. 70.

#### Barbatia barbata

(Linnaeus, 1758)

0D5D913C-E367-5566-91DE-8DD457B7C51C

##### Materials

- **Type status:** Other material. **Occurrence:** occurrenceID: 36F189F0-1395-5236-B870-190834BABCEC; **Location:** country: Italy; countryCode: IT; stateProvince: Sassari; locality: Island of Culuccia; verbatimLatitude: 41 11 46.79N; verbatimLongitude: 9 17 20.34E; geodeticDatum: WGS84
- **Type status:** Other material. **Occurrence:** occurrenceID: B17033BD-8DE3-52EE-9BFE-CB60A0D28217; **Location:** country: Italy; countryCode: IT; stateProvince: Sassari; locality: Island of Culuccia; verbatimLatitude: 41 11 56.04N; verbatimLongitude: 9 17 33.63E; geodeticDatum: WGS84
- **Type status:** Other material. **Occurrence:** occurrenceID: 1292A9FD-372A-5C44-9C9D-DD110F9CE861; **Location:** country: Italy; countryCode: IT; stateProvince: Sassari; locality: Island of Culuccia; verbatimLatitude: 41 12 28.07N; verbatimLongitude: 9 17 47.62E; geodeticDatum: WGS84

##### Notes

Alive, Fig. 71.

#### Noetiidae

6D690B24-A9DD-5E72-A54B-73FE6978A3B8

#### Striarca lactea

(Linnaeus, 1758)

DB1C7D93-F06E-5E3B-9FB9-85155C23CE58

##### Materials

- **Type status:** Other material. **Occurrence:** occurrenceID: 90AEA74C-C435-5E38-82D5-ED5D2EF9B548; **Location:** country: Italy; countryCode: IT; stateProvince: Sassari; locality: Island of Culuccia; verbatimLatitude: 41 12 32.26N; verbatimLongitude: 9 16 52.46E; geodeticDatum: WGS84
- **Type status:** Other material. **Occurrence:** occurrenceID: 262F6CD4-A2CD-572A-AD0E-BE01E731518F; **Location:** country: Italy; countryCode: IT; stateProvince: Sassari; locality: Island of Culuccia; verbatimLatitude: 41 11 56.04N; verbatimLongitude: 9 17 33.63E; geodeticDatum: WGS84
- **Type status:** Other material. **Occurrence:** occurrenceID: BB89A243-8BDD-5EFB-B6EF-8B84CEE8CC75; **Location:** country: Italy; countryCode: IT; stateProvince: Sassari; locality: Island of Culuccia; verbatimLatitude: 41 11 28.32N; verbatimLongitude: 9 16 44.50E; geodeticDatum: WGS84

##### Notes

Alive, Fig. 72.

#### Mytilida

CDA4A245-634E-5309-A4FC-603783E29F34

#### Mytilidae

77A0C0FC-519B-58D5-A50A-DEA8C11741D2

#### Litophaga lithophaga

(Linnaeus, 1758)

F002E19E-1A60-53A1-BD16-AB5BCD4EBD72

##### Materials

- **Type status:** Other material. **Occurrence:** occurrenceID: 563D985B-D31E-5C3F-9266-765CD3A8E6F8; **Location:** country: Italy; countryCode: IT; stateProvince: Sassari; locality: Island of Culuccia; verbatimLatitude: 41 11 56.04N; verbatimLongitude: 9 17 33.63E; geodeticDatum: WGS84

##### Notes

Alive.

#### Mytilaster marioni

(Locard, 1889)

5A411A7D-8A9F-5D9A-957A-8F0EBF16CBDB

##### Materials

- **Type status:** Other material. **Occurrence:** occurrenceID: EB7784B9-6F00-5EB4-9DE3-0F865ECBE5BC; **Location:** country: Italy; countryCode: IT; stateProvince: Sassari; locality: Island of Culuccia; verbatimLatitude: 41 11 46.14N; verbatimLongitude: 9 16 40.60E; geodeticDatum: WGS84

##### Notes

Alive, Fig. 73.

#### Mytilus galloprovincialis

Lamarck, 1819

C791D4F4-8389-59CD-A3CD-CBCC0ABE9672

##### Materials

- **Type status:** Other material. **Occurrence:** occurrenceID: B2D2FD74-AA3D-5F00-A460-1221EE34E72D; **Location:** country: Italy; countryCode: IT; stateProvince: Sassari; locality: Island of Culuccia; verbatimLatitude: 41 11 46.14N; verbatimLongitude: 9 16 40.60E; geodeticDatum: WGS84

##### Notes

Alive.

#### Musculus subpictus

(Cantraine 1835)

E95CDE71-C257-56C7-A6BB-FAB96F729300

##### Materials

- **Type status:** Other material. **Occurrence:** occurrenceID: 0B0142FA-F243-512A-B421-3C19CD42E9A5; **Location:** country: Italy; countryCode: IT; stateProvince: Sassari; locality: Island of Culuccia; verbatimLatitude: 41 13 17.70N; verbatimLongitude: 9 17 21.41E; geodeticDatum: WGS84

##### Notes

Shell.

#### Gregariella semigranata

(Reeve, 1858)

86BF57D3-2B1D-5812-98FA-E447797DFF91

##### Materials

- **Type status:** Other material. **Occurrence:** occurrenceID: 47CE54D4-AC3D-54DD-82A4-DC628CF2043F; **Location:** country: Italy; countryCode: IT; stateProvince: Sassari; locality: Island of Culuccia; verbatimLatitude: 41 13 17.70N; verbatimLongitude: 9 17 21.41E; geodeticDatum: WGS84

##### Notes

Alive; new record.

#### Pectinida

67908E4B-384E-5F4F-BA56-82C6726DE055

#### Spondylidae

C5CA1A6E-765C-5012-B0D9-32817CCE3323

#### Spondylus gaederopus

Linnaeus, 1758

5157C7C1-DA49-543F-870D-B5EC1E879C75

##### Materials

- **Type status:** Other material. **Occurrence:** occurrenceID: 54AFBBC5-3AB8-5CE4-9067-2D0B5321F127; **Location:** country: Italy; countryCode: IT; stateProvince: Sassari; locality: Island of Culuccia; verbatimLatitude: 41 12 42.40N; verbatimLongitude: 9 17 45.35E; geodeticDatum: WGS84
- **Type status:** Other material. **Occurrence:** occurrenceID: 01D358C1-EC4C-591E-8B32-0D02E6C6C1D3; **Location:** country: Italy; countryCode: IT; stateProvince: Sassari; locality: Island of Culuccia; verbatimLatitude: 41 13 17.70N; verbatimLongitude: 9 17 21.41E; geodeticDatum: WGS84

##### Notes

Alive, Fig. 74.

#### Pectinidae

71FDBF45-9754-52C5-9654-10951817C1EF

#### Mimachlamys varia

(Linnaeus, 1758)

0CAA2E7F-0D55-5C1A-976C-19FAEE3EACCF

##### Materials

- **Type status:** Other material. **Occurrence:** occurrenceID: D9D24F73-DFF1-50E4-9A00-C4F459A9C198; **Location:** country: Italy; countryCode: IT; stateProvince: Sassari; locality: Island of Culuccia; verbatimLatitude: 41 11 28.32N; verbatimLongitude: 9 16 44.50E; geodeticDatum: WGS84
- **Type status:** Other material. **Occurrence:** occurrenceID: 3C054522-5EC1-5940-AAC6-3892AAEDE665; **Location:** country: Italy; countryCode: IT; stateProvince: Sassari; locality: Island of Culuccia; verbatimLatitude: 41 11 22.14N; verbatimLongitude: 9 16 59.05E; geodeticDatum: WGS84
- **Type status:** Other material. **Occurrence:** occurrenceID: 9EECABD4-3FBD-5B6E-BA85-03896FF463C3; **Location:** country: Italy; countryCode: IT; stateProvince: Sassari; locality: Island of Culuccia; verbatimLatitude: 41 12 47.63N; verbatimLongitude: 9 17 08.68E; geodeticDatum: WGS84

##### Notes

Alive, Fig. 75.

#### Manupecten pesfelis

(Linnaeus, 1758)

43589166-68F3-5E23-9A20-D790932A4A44

##### Materials

- **Type status:** Other material. **Occurrence:** occurrenceID: 714105D3-8A5F-5F0F-9737-ACBB90847EB9; **Location:** country: Italy; countryCode: IT; stateProvince: Sassari; locality: Island of Culuccia; verbatimLatitude: 41 13 17.70N; verbatimLongitude: 9 17 21.41E; geodeticDatum: WGS84

##### Notes

Alive, Fig. 76.

#### Talochlamys multistriata

(Poli, 1795)

771F8DBE-8C13-5FD2-959B-F495EEB3CCFD

##### Materials

- **Type status:** Other material. **Occurrence:** occurrenceID: FC6B472B-1C26-5705-8556-1A77E2AD7CEF; **Location:** country: Italy; countryCode: IT; stateProvince: Sassari; locality: Island of Culuccia; verbatimLatitude: 41 11 28.32N; verbatimLongitude: 9 16 44.50E; geodeticDatum: WGS84

##### Notes

Shell, Fig. 77.

#### Flexopecten hyalinus

(Poli, 1795)

4053EBD4-2492-5CAE-AE88-B4F8CA5D8E78

##### Materials

- **Type status:** Other material. **Occurrence:** occurrenceID: CB7E458E-B90C-574B-B0BE-23D7526F5088; **Location:** country: Italy; countryCode: IT; stateProvince: Sassari; locality: Island of Culuccia; verbatimLatitude: 41 13 17.70N; verbatimLongitude: 9 17 21.41E; geodeticDatum: WGS84

##### Notes

Alive.

#### Palliolum incomparabile

(Risso, 1826)

9644859E-2959-55FD-8BDD-4D17AB626CB2

##### Materials

- **Type status:** Other material. **Occurrence:** occurrenceID: DDE27085-80FB-565D-ADFE-58FEC08C90DD; **Location:** country: Italy; countryCode: IT; stateProvince: Sassari; locality: Island of Culuccia; verbatimLatitude: 41 13 17.70N; verbatimLongitude: 9 17 21.41E; geodeticDatum: WGS84

##### Notes

Shell.

#### Anomiidae

2B6FDA81-7CC1-5DF5-AE3A-25C70CD6E640

#### Anomia ephippium

Linnaeus, 1758

EAFECBD7-B0B1-55D7-BE1A-279FEE5D2879

##### Materials

- **Type status:** Other material. **Occurrence:** occurrenceID: 595BEE47-52FF-502A-9E31-F0AE8AA22F17; **Location:** country: Italy; countryCode: IT; stateProvince: Sassari; locality: Island of Culuccia; verbatimLatitude: 41 12 09.61N; verbatimLongitude: 9 16 43.24E; geodeticDatum: WGS84
- **Type status:** Other material. **Occurrence:** occurrenceID: 06B859A6-4BB0-5C7A-A425-F4116C3D4775; **Location:** country: Italy; countryCode: IT; stateProvince: Sassari; locality: Island of Culuccia; verbatimLatitude: 41 11 46.14N; verbatimLongitude: 9 16 40.60E; geodeticDatum: WGS84
- **Type status:** Other material. **Occurrence:** occurrenceID: AEB0FEBE-31F3-5F74-93B3-AD35B1FA59D5; **Location:** country: Italy; countryCode: IT; stateProvince: Sassari; locality: Island of Culuccia; verbatimLatitude: 41 11 28.32N; verbatimLongitude: 9 16 44.50E; geodeticDatum: WGS84
- **Type status:** Other material. **Occurrence:** occurrenceID: 54D9670E-25B9-547A-A47F-B9C6A3BCBDCC; **Location:** country: Italy; countryCode: IT; stateProvince: Sassari; locality: Island of Culuccia; verbatimLatitude: 41 11 22.14N; verbatimLongitude: 9 16 59.05E; geodeticDatum: WGS84

##### Notes

Alive, Fig. 78.

#### Pododesmus squama

(Gmelin, 1791)

86233CA0-FABC-5EB1-9C5C-713A7FF62563

##### Materials

- **Type status:** Other material. **Occurrence:** occurrenceID: 3DC49077-90A0-5EA0-9865-AE6498D37561; **Location:** country: Italy; countryCode: IT; stateProvince: Sassari; locality: Island of Culuccia; verbatimLatitude: 41 12 47.63N; verbatimLongitude: 9 17 08.68E; geodeticDatum: WGS84

##### Notes

Alive.

#### Limida

109A8D21-AF38-5A42-B271-ED3415A1043A

#### Limidae

14D10A92-A5AC-56D8-97BA-58047805A2BF

#### Lima lima

(Linnaeus, 1758)

FCEEA00F-90E6-5F30-A286-3B3FA3C8A625

##### Materials

- **Type status:** Other material. **Occurrence:** occurrenceID: 15E606FA-B380-5E20-B4CE-14CAE4F2266E; **Location:** country: Italy; countryCode: IT; stateProvince: Sassari; locality: Island of Culuccia; verbatimLatitude: 41 12 28.07N; verbatimLongitude: 9 17 47.62E; geodeticDatum: WGS84
- **Type status:** Other material. **Occurrence:** occurrenceID: C5B377AB-109C-5734-8B02-C3DE9B944B1E; **Location:** country: Italy; countryCode: IT; stateProvince: Sassari; locality: Island of Culuccia; verbatimLatitude: 41 12 58.16N; verbatimLongitude: 9 17 09.66E; geodeticDatum: WGS84

##### Notes

Alive, Fig. 79.

#### Limaria hians

(Gmelin, 1791)

E4D799B5-C6B2-5FF7-8000-6549831268CE

##### Materials

- **Type status:** Other material. **Occurrence:** occurrenceID: 795E1637-6EA3-5A35-A871-EFDDD0CD8CFF; **Location:** country: Italy; countryCode: IT; stateProvince: Sassari; locality: Island of Culuccia; verbatimLatitude: 41 12 42.40N; verbatimLongitude: 9 17 45.35E; geodeticDatum: WGS84
- **Type status:** Other material. **Occurrence:** occurrenceID: D6C862E1-0B8D-5CFA-9188-C5C458B82DE8; **Location:** country: Italy; countryCode: IT; stateProvince: Sassari; locality: Island of Culuccia; verbatimLatitude: 41 12 28.07N; verbatimLongitude: 9 17 47.62E; geodeticDatum: WGS84
- **Type status:** Other material. **Occurrence:** occurrenceID: 2B94FDA3-74D0-5269-B624-76EB1F5E42CE; **Location:** country: Italy; countryCode: IT; stateProvince: Sassari; locality: Island of Culuccia; verbatimLatitude: 41 12 58.16N; verbatimLongitude: 9 17 09.66E; geodeticDatum: WGS84

##### Notes

Alive, Fig. 80, Suppl. material 6.

#### Limaria tuberculata

(Olivi, 1792)

226B2FB4-6D38-5274-B6F5-A87966B7E845

##### Materials

- **Type status:** Other material. **Occurrence:** occurrenceID: 9E28F910-12F6-5F1C-9E60-9A6CAC9C45E1; **Location:** country: Italy; countryCode: IT; stateProvince: Sassari; locality: Island of Culuccia; verbatimLatitude: 41 12 09.61N; verbatimLongitude: 9 16 43.24E; geodeticDatum: WGS84
- **Type status:** Other material. **Occurrence:** occurrenceID: 98BB4B1D-316A-5489-80A3-04A5DC3C023B; **Location:** country: Italy; countryCode: IT; stateProvince: Sassari; locality: Island of Culuccia; verbatimLatitude: 41 11 28.32N; verbatimLongitude: 9 16 44.50E; geodeticDatum: WGS84
- **Type status:** Other material. **Occurrence:** occurrenceID: 6AAB8549-E613-5B00-B50B-7BD0B271254F; **Location:** country: Italy; countryCode: IT; stateProvince: Sassari; locality: Island of Culuccia; verbatimLatitude: 41 11 22.14N; verbatimLongitude: 9 16 59.05E; geodeticDatum: WGS84

##### Notes

Alive, Fig. 81, Suppl. material 7.

#### Ostreida

E104949C-F35E-5084-865A-9A866878BE9D

#### Ostreidae

64359D42-21D7-5EB3-8DAA-E631ADF6E9E0

#### Ostrea edulis

Linnaeus, 1758

620D0F34-F390-564B-8581-3960190491BB

##### Materials

- **Type status:** Other material. **Occurrence:** occurrenceID: 2C2057D8-32C2-521F-BB83-43BD32339F86; **Location:** country: Italy; countryCode: IT; stateProvince: Sassari; locality: Island of Culuccia; verbatimLatitude: 41 11 46.79N; verbatimLongitude: 9 17 20.34E; geodeticDatum: WGS84
- **Type status:** Other material. **Occurrence:** occurrenceID: 8AA117F8-7315-5E43-B08F-F9EE3AD421EB; **Location:** country: Italy; countryCode: IT; stateProvince: Sassari; locality: Island of Culuccia; verbatimLatitude: 41 11 56.04N; verbatimLongitude: 9 17 33.63E; geodeticDatum: WGS84
- **Type status:** Other material. **Occurrence:** occurrenceID: 8A4C9FC5-AC56-5ABB-A9EC-BF6146F99534; **Location:** country: Italy; countryCode: IT; stateProvince: Sassari; locality: Island of Culuccia; verbatimLatitude: 41 12 47.63N; verbatimLongitude: 9 17 08.68E; geodeticDatum: WGS84
- **Type status:** Other material. **Occurrence:** occurrenceID: 66DBEABC-6298-5EF9-86EF-05320EC5B3B3; **Location:** country: Italy; countryCode: IT; stateProvince: Sassari; locality: Island of Culuccia; verbatimLatitude: 41 11 28.32N; verbatimLongitude: 9 16 44.50E; geodeticDatum: WGS84

##### Notes

Alive, Fig. 82.

#### Ostrea stentina

Payraudeau, 1826

2E4E1483-127A-5E72-B30A-B3700C02DF57

##### Materials

- **Type status:** Other material. **Occurrence:** occurrenceID: 844AE492-A214-5B9C-A2E1-D70B1572D7E3; **Location:** country: Italy; countryCode: IT; stateProvince: Sassari; locality: Island of Culuccia; verbatimLatitude: 41 11 46.14N; verbatimLongitude: 9 16 40.60E; geodeticDatum: WGS84
- **Type status:** Other material. **Occurrence:** occurrenceID: 3A689CF4-DD91-5C1B-BE92-2C2AC9C8CF1A; **Location:** country: Italy; countryCode: IT; stateProvince: Sassari; locality: Island of Culuccia; verbatimLatitude: 41 11 28.32N; verbatimLongitude: 9 16 44.50E; geodeticDatum: WGS84

##### Notes

Alive, Fig. 83.

#### Magallana gigas

(Thunberg, 1793)

FB1798D8-1B04-55A2-A3DB-D055C207E2F2

##### Materials

- **Type status:** Other material. **Occurrence:** occurrenceID: 0129F0F7-645C-5EB2-AFC8-57EAB894A84C; **Location:** country: Italy; countryCode: IT; stateProvince: Sassari; locality: Island of Culuccia; verbatimLatitude: 41 12 09.61N; verbatimLongitude: 9 16 43.24E; geodeticDatum: WGS84

##### Notes

Shell, Fig. 84; Non-indigenous species.

#### Pinnidae

81EF776D-D49D-56D1-A9F0-527729DE6A64

#### Pinna nobilis

Linnaeus, 1758

83110101-38AC-507B-94C0-59BF181D7D8B

##### Materials

- **Type status:** Other material. **Occurrence:** occurrenceID: 70F8D837-B77C-51C6-B652-D3BE862C68B1; **Location:** country: Italy; countryCode: IT; stateProvince: Sassari; locality: Island of Culuccia; verbatimLatitude: 41 12 47.63N; verbatimLongitude: 9 17 08.68E; geodeticDatum: WGS84
- **Type status:** Other material. **Occurrence:** occurrenceID: FA0D2168-DDAB-5882-9815-A10312E5C089; **Location:** country: Italy; countryCode: IT; stateProvince: Sassari; locality: Island of Culuccia; verbatimLatitude: 41 12 32.26N; verbatimLongitude: 9 16 52.46E; geodeticDatum: WGS84

##### Notes

Shell, Fig. 85.

#### Margaritidae

4FF7CDE6-BDFE-58D3-B928-6109E60BE933

#### Pinctada radiata

(Leach, 1814)

AE3BEFE3-27E6-53D3-AF82-2CFE41D500EA

##### Materials

- **Type status:** Other material. **Occurrence:** occurrenceID: F4AB8AF3-C794-55AB-968D-778E8CE59DA7; **Location:** country: Italy; countryCode: IT; stateProvince: Sassari; locality: Island of Culuccia; verbatimLatitude: 41 12 47.63N; verbatimLongitude: 9 17 08.68E; geodeticDatum: WGS84

##### Notes

Shell, Fig. 86, Non-Indigenous Species.

#### Lucinida

25B33B90-F110-51D9-ADAB-795E1F35FA1A

#### Lucinidae

EB84188A-84F3-5A75-9731-46F848CF5557

#### Ctena decussata

(O. G. Costa, 1829)

B5A3BA6F-BC07-59F4-B9FC-9DC560F9113E

##### Materials

- **Type status:** Other material. **Occurrence:** occurrenceID: F945A2E8-61E0-582D-BFEE-B91D42376C19; **Location:** country: Italy; countryCode: IT; stateProvince: Sassari; locality: Island of Culuccia; verbatimLatitude: 41 13 17.70N; verbatimLongitude: 9 17 21.41E; geodeticDatum: WGS84

##### Notes

Shell.

#### Loripes lacteus

(Linnaeus, 1758)

2C83EA3E-EDE2-52F7-8286-6C94283FCB35

##### Materials

- **Type status:** Other material. **Occurrence:** occurrenceID: D717317E-47BC-5544-8A63-09FEE8FD8CCC; **Location:** country: Italy; countryCode: IT; stateProvince: Sassari; locality: Island of Culuccia; verbatimLatitude: 41 12 28.07N; verbatimLongitude: 9 17 47.62E; geodeticDatum: WGS84

##### Notes

Alive, Fig. 87.

#### Galeommatida

59C0041F-2244-518F-AA11-B954B46BA75B

#### Galeommatidae

AF19E177-8559-5DBC-9313-92DABCC4FD4E

#### Galeomma turtoni

W. Turton, 1825

762DDB23-6937-5091-A366-D2070A259334

##### Materials

- **Type status:** Other material. **Occurrence:** occurrenceID: E8D1DDE9-3D48-5EC4-B6A1-CC9CEE89E234; **Location:** country: Italy; countryCode: IT; stateProvince: Sassari; locality: Island of Culuccia; verbatimLatitude: 41 12 58.16N; verbatimLongitude: 9 17 09.66E; geodeticDatum: WGS84

##### Notes

Alive, Fig. 88.

#### Carditida

D569CF90-7871-5138-BE9E-529CBA4031FA

#### Carditidae

7ABE44D7-562B-595A-A108-1496B5243914

#### Glans trapezia

(Linnaeus, 1767)

9C43ADEF-E0CD-515C-A7DF-91A849278956

##### Materials

- **Type status:** Other material. **Occurrence:** occurrenceID: FD29E6B8-E0A0-5E1B-862C-90EB59F8AFFB; **Location:** country: Italy; countryCode: IT; stateProvince: Sassari; locality: Island of Culuccia; verbatimLatitude: 41 13 17.70N; verbatimLongitude: 9 17 21.41E; geodeticDatum: WGS84

##### Notes

Shell.

#### Cardites antiquatus

(Linnaeus, 1758)

8AAFB470-6B5A-53D3-AFE6-7DD33E7211FB

##### Materials

- **Type status:** Other material. **Occurrence:** occurrenceID: F42AAD43-D8A0-5BDD-970A-D468B2F32D55; **Location:** country: Italy; countryCode: IT; stateProvince: Sassari; locality: Island of Culuccia; verbatimLatitude: 41 11 56.04N; verbatimLongitude: 9 17 33.63E; geodeticDatum: WGS84
- **Type status:** Other material. **Occurrence:** occurrenceID: F1A07784-3205-5875-B01E-9AA96410C24D; **Location:** country: Italy; countryCode: IT; stateProvince: Sassari; locality: Island of Culuccia; verbatimLatitude: 41 12 13.88N; verbatimLongitude: 9 17 56.14E; geodeticDatum: WGS84

##### Notes

Shell.

#### Cardita calyculata

(Linnaeus, 1758)

ED5D9CB9-2D3B-5CBC-AEC8-506C02D8ED64

##### Materials

- **Type status:** Other material. **Occurrence:** occurrenceID: 157B7DB0-DFAE-5F5B-9782-C91A8ADA8231; **Location:** country: Italy; countryCode: IT; stateProvince: Sassari; locality: Island of Culuccia; verbatimLatitude: 41 11 46.14N; verbatimLongitude: 9 16 40.60E; geodeticDatum: WGS84

##### Notes

Alive, Fig. 89.

#### Cardiidae

25E1188E-8C72-5145-B1AA-3BF3DD1E2D0F

#### Acanthocardia paucicostata

(G. B. Sowerby II, 1834)

4DA4D34B-BE62-5E40-86D2-127087BB6183

##### Materials

- **Type status:** Other material. **Occurrence:** occurrenceID: 3AB98DC1-2C4C-5725-A5A2-4EAA2F3F6C1E; **Location:** country: Italy; countryCode: IT; stateProvince: Sassari; locality: Island of Culuccia; verbatimLatitude: 41 11 46.79N; verbatimLongitude: 9 17 20.34E; geodeticDatum: WGS84
- **Type status:** Other material. **Occurrence:** occurrenceID: 12DC9F18-679F-54D9-9218-A8AFA72F5672; **Location:** country: Italy; countryCode: IT; stateProvince: Sassari; locality: Island of Culuccia; verbatimLatitude: 41 11 56.04N; verbatimLongitude: 9 17 33.63E; geodeticDatum: WGS84

##### Notes

Shell.

#### Acanthocardia tuberculata

(Linnaeus, 1758)

8123AF71-4172-5013-B37B-17C4BBE641BC

##### Materials

- **Type status:** Other material. **Occurrence:** occurrenceID: 48A8D8B1-F947-56CF-A247-9A5A9E219B0B; **Location:** country: Italy; countryCode: IT; stateProvince: Sassari; locality: Island of Culuccia; verbatimLatitude: 41 11 46.79N; verbatimLongitude: 9 17 20.34E; geodeticDatum: WGS84
- **Type status:** Other material. **Occurrence:** occurrenceID: 7AF58FDB-5FD0-5578-8898-62D681A5D6CE; **Location:** country: Italy; countryCode: IT; stateProvince: Sassari; locality: Island of Culuccia; verbatimLatitude: 41 11 56.04N; verbatimLongitude: 9 17 33.63E; geodeticDatum: WGS84

##### Notes

Shell.

#### Cerastoderma glaucum

(Bruguière, 1789)

5571867B-F14D-507F-8758-A9C9529EB34A

##### Materials

- **Type status:** Other material. **Occurrence:** occurrenceID: 661A17BC-515A-5617-94D8-E76E10E4F20F; **Location:** country: Italy; countryCode: IT; stateProvince: Sassari; locality: Island of Culuccia; verbatimLatitude: 41 11 22.14N; verbatimLongitude: 9 16 59.05E; geodeticDatum: WGS84

##### Notes

Alive, Fig. 90; new record.

#### Parvicardium exiguum

(Gmelin, 1791)

142D6939-4B8E-5CA9-9BEA-AE8833DC0D78

##### Materials

- **Type status:** Other material. **Occurrence:** occurrenceID: A2B28C0C-66DE-546B-AA69-B3431E1A2812; **Location:** country: Italy; countryCode: IT; stateProvince: Sassari; locality: Island of Culuccia; verbatimLatitude: 41 13 17.70N; verbatimLongitude: 9 17 21.41E; geodeticDatum: WGS84

##### Notes

Shell.

#### Papillicardium papillosum

(Poli, 1791)

D143C32E-A127-5109-82BD-787DA90D4FD5

##### Materials

- **Type status:** Other material. **Occurrence:** occurrenceID: 4BE039B9-2C88-5251-A9A1-D53439CC3EF1; **Location:** country: Italy; countryCode: IT; stateProvince: Sassari; locality: Island of Culuccia; verbatimLatitude: 41 13 17.70N; verbatimLongitude: 9 17 21.41E; geodeticDatum: WGS84

##### Notes

Shell.

#### Tellinidae

49673B47-A011-54A2-AA02-513313ED5087

#### Bosemprella incarnata

(Linnaeus, 1758)

42D961D8-A75F-5605-8DD2-EBD27B0E1F7C

##### Materials

- **Type status:** Other material. **Occurrence:** occurrenceID: BD56C427-D004-563F-887B-520CE461DAC4; **Location:** country: Italy; countryCode: IT; stateProvince: Sassari; locality: Island of Culuccia; verbatimLatitude: 41 11 22.14N; verbatimLongitude: 9 16 59.05E; geodeticDatum: WGS84

##### Notes

Shell, Fig. 91.

#### Fabulina fabula

(Gmelin, 1791)

E5EA21B7-AA33-5571-9B85-0535AA8B04CC

##### Materials

- **Type status:** Other material. **Occurrence:** occurrenceID: 064A050D-4000-5ECB-A976-674F561F247B; **Location:** country: Italy; countryCode: IT; stateProvince: Sassari; locality: Island of Culuccia; verbatimLatitude: 41 11 22.14N; verbatimLongitude: 9 16 59.05E; geodeticDatum: WGS84

##### Notes

Shell.

#### Moerella donacina

(Linnaues, 1758)

485EC20D-12C9-520C-8656-E13E3E7B0BCA

##### Materials

- **Type status:** Other material. **Occurrence:** occurrenceID: B15468FF-56EE-551A-8831-F9CC62FEC680; **Location:** country: Italy; countryCode: IT; stateProvince: Sassari; locality: Island of Culuccia; verbatimLatitude: 41 11 22.14N; verbatimLongitude: 9 16 59.05E; geodeticDatum: WGS84

##### Notes

Shell, Fig. 92.

#### Moerella pulchella

(Lamarck, 1818)

7BD58FEC-1576-5ECA-940A-6CAE2625AADC

##### Materials

- **Type status:** Other material. **Occurrence:** occurrenceID: 92F4D01D-6C86-52F3-B950-063709CDDB09; **Location:** country: Italy; countryCode: IT; stateProvince: Sassari; locality: Island of Culuccia; verbatimLatitude: 41 11 22.14N; verbatimLongitude: 9 16 59.05E; geodeticDatum: WGS84

##### Notes

Shell, Fig. 93.

#### Peronaea planata

(Linnaeus, 1758)

7AED92BC-4654-585E-9E25-3616B7C71371

##### Materials

- **Type status:** Other material. **Occurrence:** occurrenceID: 571371E8-DFF7-5FEE-B8A2-14258D0BB22A; **Location:** country: Italy; countryCode: IT; stateProvince: Sassari; locality: Island of Culuccia; verbatimLatitude: 41 11 22.14N; verbatimLongitude: 9 16 59.05E; geodeticDatum: WGS84

##### Notes

Alive, Fig. 94.

#### Peronidia albicans

(Gmelin, 1791)

A90FE830-0976-58F2-958E-E33BCE41C583

##### Materials

- **Type status:** Other material. **Occurrence:** occurrenceID: C3A999B2-D142-5EC2-A59A-F94AC1FD46E2; **Location:** country: Italy; countryCode: IT; stateProvince: Sassari; locality: Island of Culuccia; verbatimLatitude: 41 11 22.14N; verbatimLongitude: 9 16 59.05E; geodeticDatum: WGS84

##### Notes

Alive, Fig. 95.

#### Arcopella balaustina

(Linnaeus, 1758)

513A93E7-549A-5375-A98C-F22B87F4A9A5

##### Materials

- **Type status:** Other material. **Occurrence:** occurrenceID: AEA23BCF-9A08-59E7-A144-B301624FC721; **Location:** country: Italy; countryCode: IT; stateProvince: Sassari; locality: Island of Culuccia; verbatimLatitude: 41 13 17.70N; verbatimLongitude: 9 17 21.41E; geodeticDatum: WGS84

##### Notes

Alive.

#### Gastrana fragilis

(Linnaeus, 1758)

CBFD0CF8-453B-5AAD-B796-D8497E924AD8

##### Materials

- **Type status:** Other material. **Occurrence:** occurrenceID: C6855869-12F3-548B-AEBA-A1C25EF6DA4C; **Location:** country: Italy; countryCode: IT; stateProvince: Sassari; locality: Island of Culuccia; verbatimLatitude: 41 12 13.88N; verbatimLongitude: 9 17 56.14E; geodeticDatum: WGS84
- **Type status:** Other material. **Occurrence:** occurrenceID: 06D9F221-8274-58F9-934F-B69CE5FB8349; **Location:** country: Italy; countryCode: IT; stateProvince: Sassari; locality: Island of Culuccia; verbatimLatitude: 41 12 28.07N; verbatimLongitude: 9 17 47.62E; geodeticDatum: WGS84
- **Type status:** Other material. **Occurrence:** occurrenceID: 1643FA58-259B-5582-A135-966636DA45A2; **Location:** country: Italy; countryCode: IT; stateProvince: Sassari; locality: Island of Culuccia; verbatimLatitude: 41 11 22.14N; verbatimLongitude: 9 16 59.05E; geodeticDatum: WGS84

##### Notes

Alive.

#### Solecurtidae

6E4707B6-DEBC-54E3-AD5D-454D9D70FAC5

#### Solecurtus strigilatus

(Linnaeus, 1758)

22D46959-FB14-51CA-BB51-734C7C501561

##### Materials

- **Type status:** Other material. **Occurrence:** occurrenceID: 1328ACE8-9E15-5605-B78F-F48DD07E136C; **Location:** country: Italy; countryCode: IT; stateProvince: Sassari; locality: Island of Culuccia; verbatimLatitude: 41 12 28.07N; verbatimLongitude: 9 17 47.62E; geodeticDatum: WGS84

##### Notes

Shell, Fig. 96.

#### Donacidae

D3E2BD23-421A-5807-9961-ED668AD5A89B

#### Donax trunculus

Linnaeus, 1758

50B7FFF0-85AF-5D8B-B1EF-71FE8C18E409

##### Materials

- **Type status:** Other material. **Occurrence:** occurrenceID: 7D36E02C-321C-5180-8DEF-4F76BE3497AF; **Location:** country: Italy; countryCode: IT; stateProvince: Sassari; locality: Island of Culuccia; verbatimLatitude: 41 11 46.79N; verbatimLongitude: 9 17 20.34E; geodeticDatum: WGS84

##### Notes

Shell.

#### Donax variegatus

(Gmelin, 1791)

80C098C7-6BF0-586F-BC6F-D37EC62A3755

##### Materials

- **Type status:** Other material. **Occurrence:** occurrenceID: 4F98193B-D49D-59E7-A752-34E7356CBC47; **Location:** country: Italy; countryCode: IT; stateProvince: Sassari; locality: Island of Culuccia; verbatimLatitude: 41 13 17.70N; verbatimLongitude: 9 17 21.41E; geodeticDatum: WGS84

##### Notes

Alive, Fig. 97.

#### Psammobiidae

1703413E-B366-528D-8E02-F1F3970380EB

#### Gari costulata

(W. Turton, 1822)

431C3EDA-E8FA-5426-8FFC-C9A2D1B91984

##### Materials

- **Type status:** Other material. **Occurrence:** occurrenceID: 06D9D22F-033C-585D-B5C2-5FC66C2E0619; **Location:** country: Italy; countryCode: IT; stateProvince: Sassari; locality: Island of Culuccia; verbatimLatitude: 41 11 46.79N; verbatimLongitude: 9 17 20.34E; geodeticDatum: WGS84

##### Notes

Alive.

#### Gari depressa

(Pennant, 1777)

**Figure 6.**
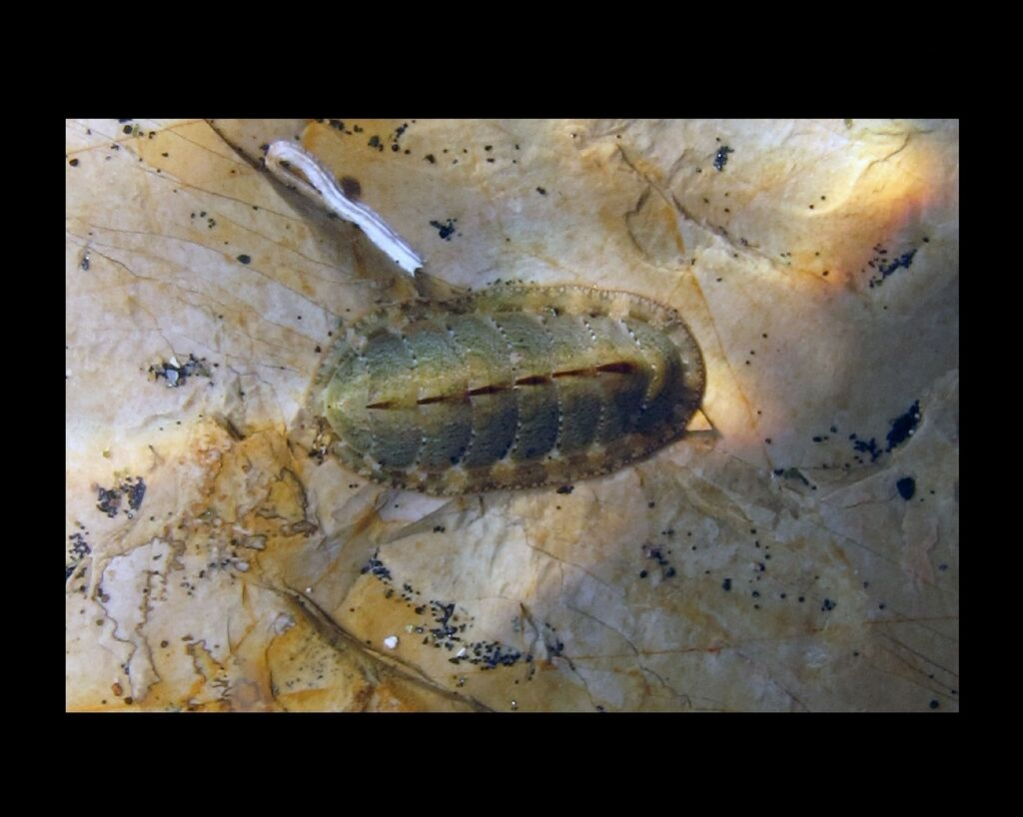
*Lepidochitonacinerea* (Linnaeus, 1767). *In situ* photograph.

**Figure 7.**
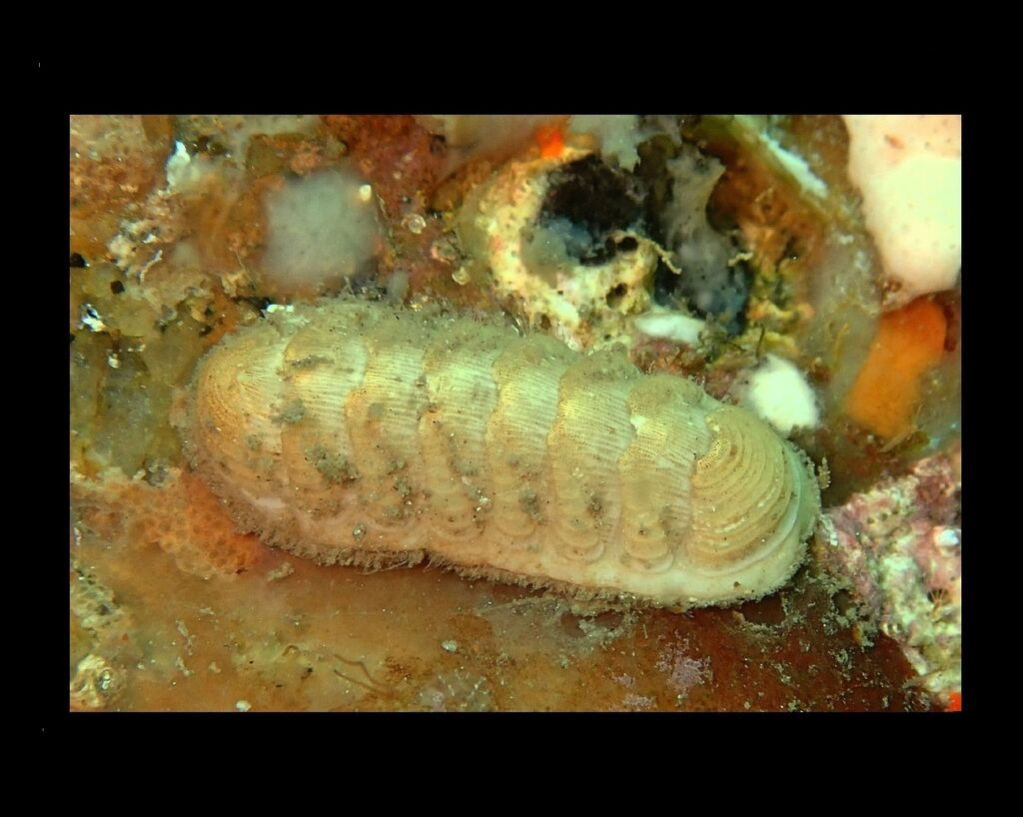
*Lepidopleuruscajetanus* (Poli, 1791). *In situ* photograph.

**Figure 13.**
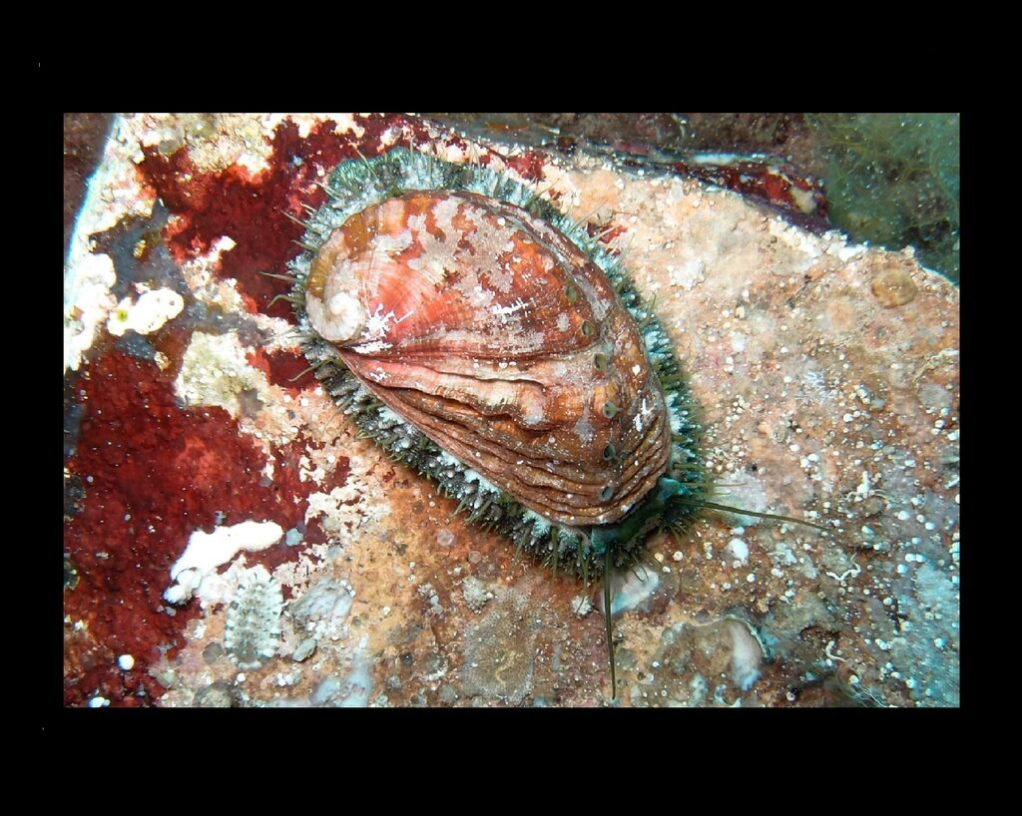
*Haliotistuberculata* Linnaeus, 1758. *In situ* photograph.

**Figure 46.**
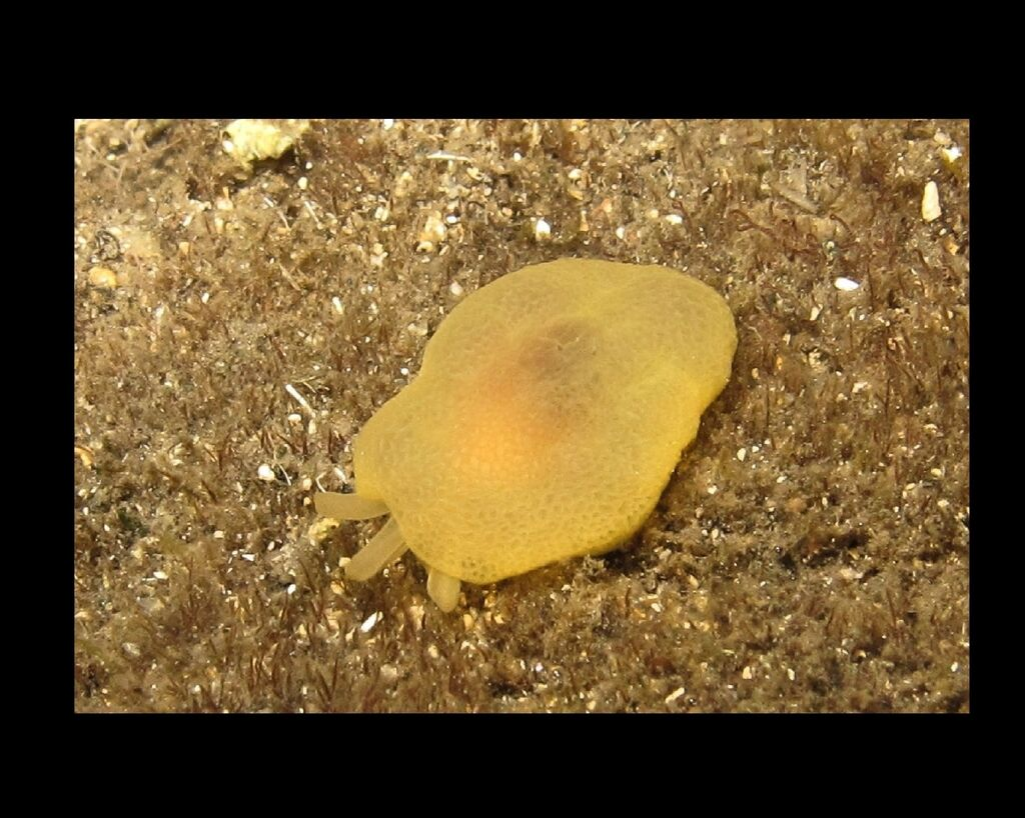
*Berthellaplumula* (Montagu, 1803). *In situ* photograph.

**Figure 47.**
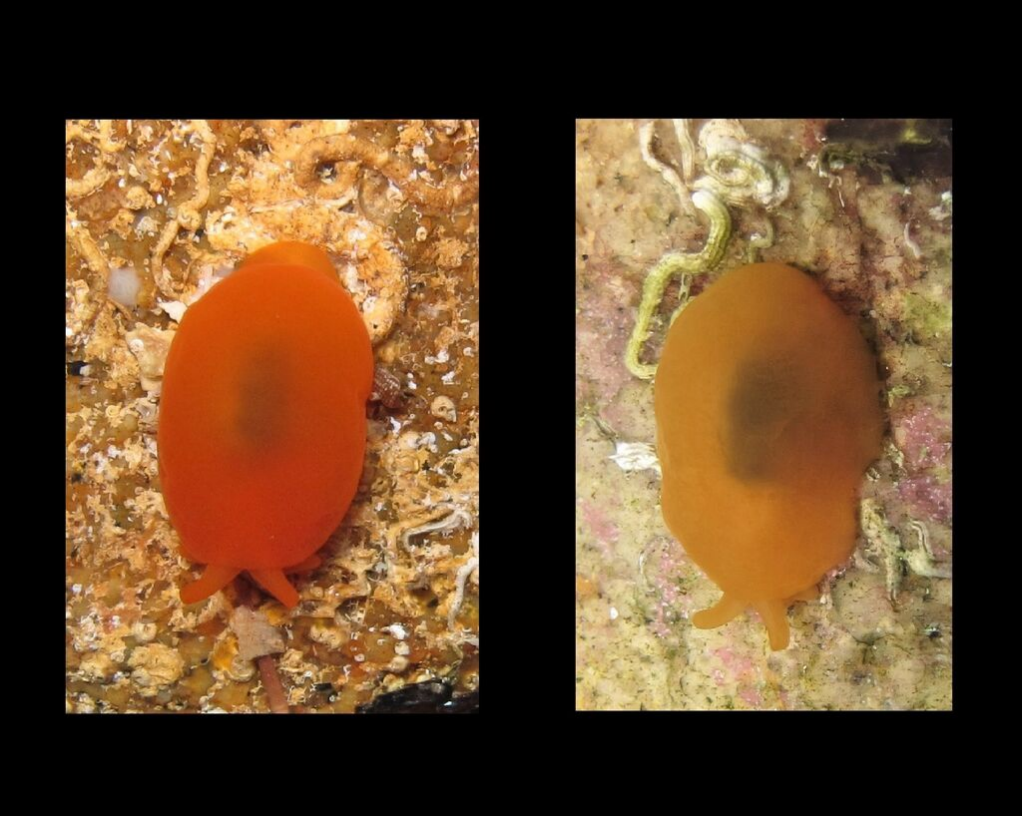
*Berthellinaaurantiaca* (Risso, 1818). Different colour morphotypes. *In situ* photographs.

**Figure 52.**
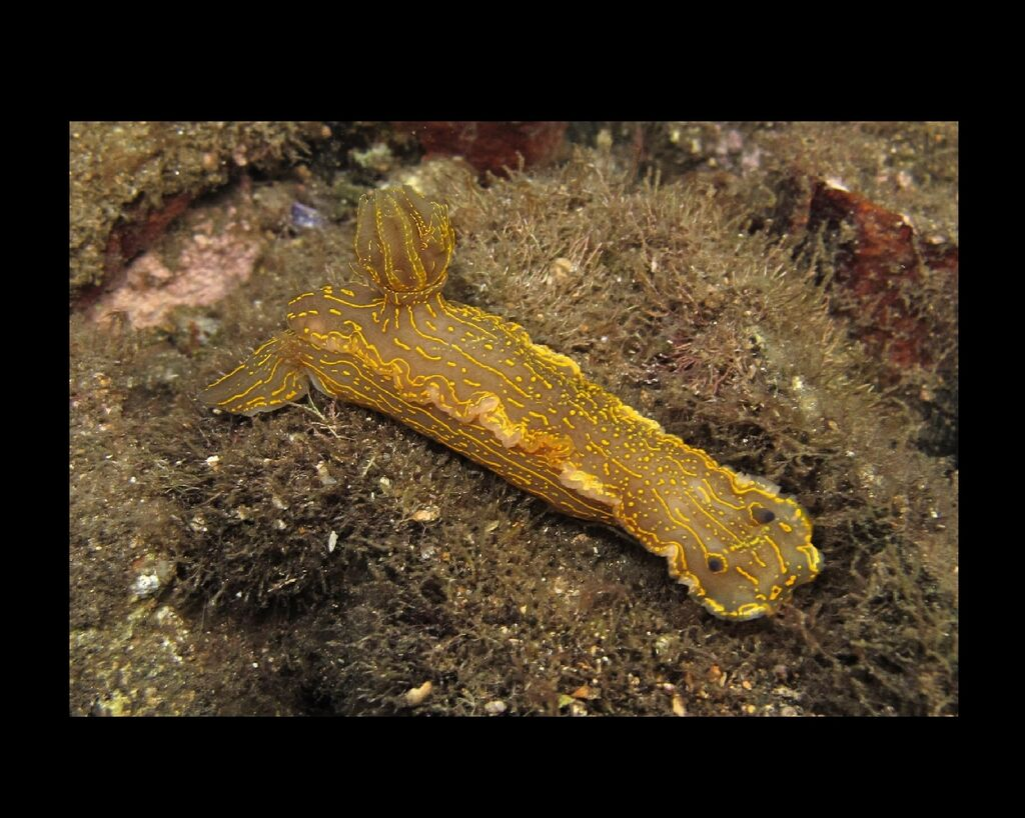
*Felimarepicta* (Schultz in Philippi, 1836). *In situ* photograph.

**Figure 61.**
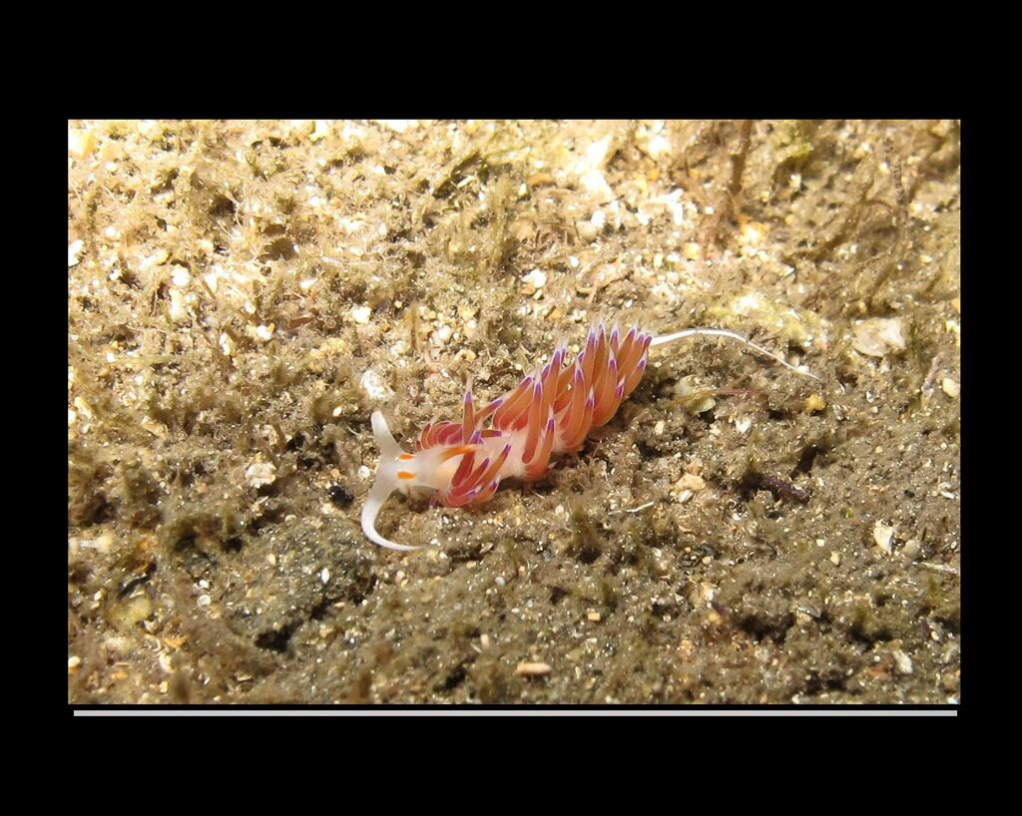
*Cratenaperegrina* (Gmelin, 1791). *In situ* photograph.

**Figure 66.**
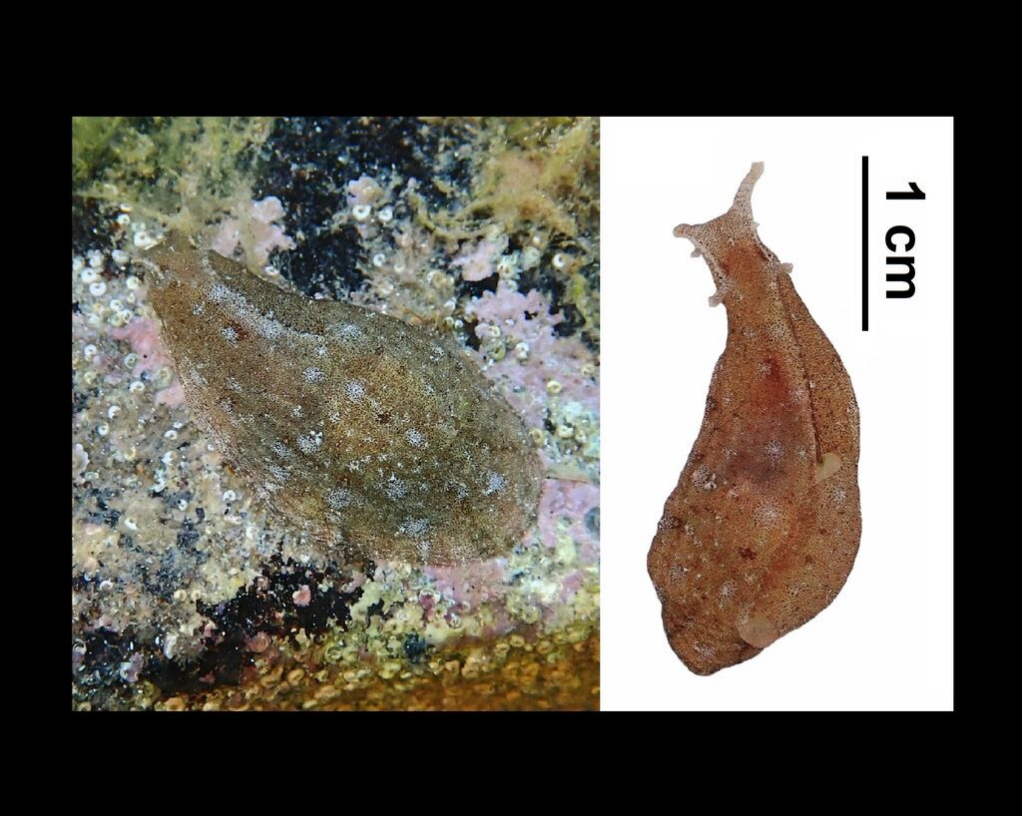
*Petaliferapetalifera* (Rang, 1828). On the left, *in situ* photograph; on the right, optical photograph.

**Figure 80.**
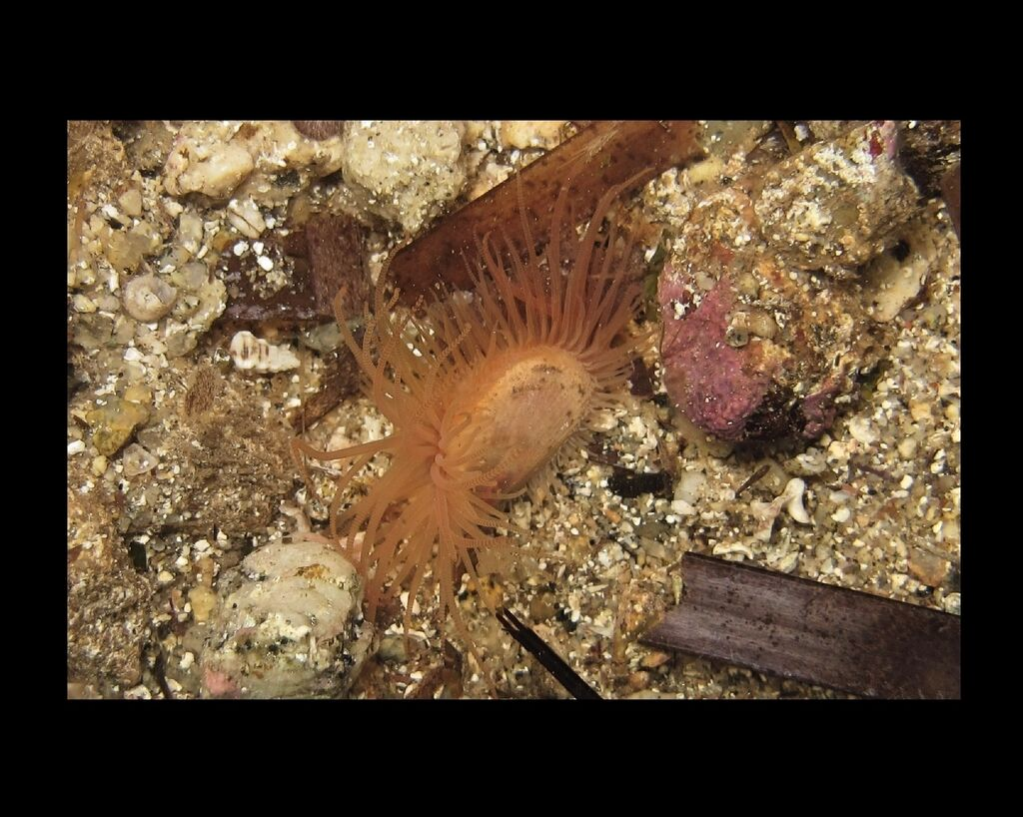
*Limariahians* (Gmelin, 1791). *In situ* photograph.

**Figure 81.**
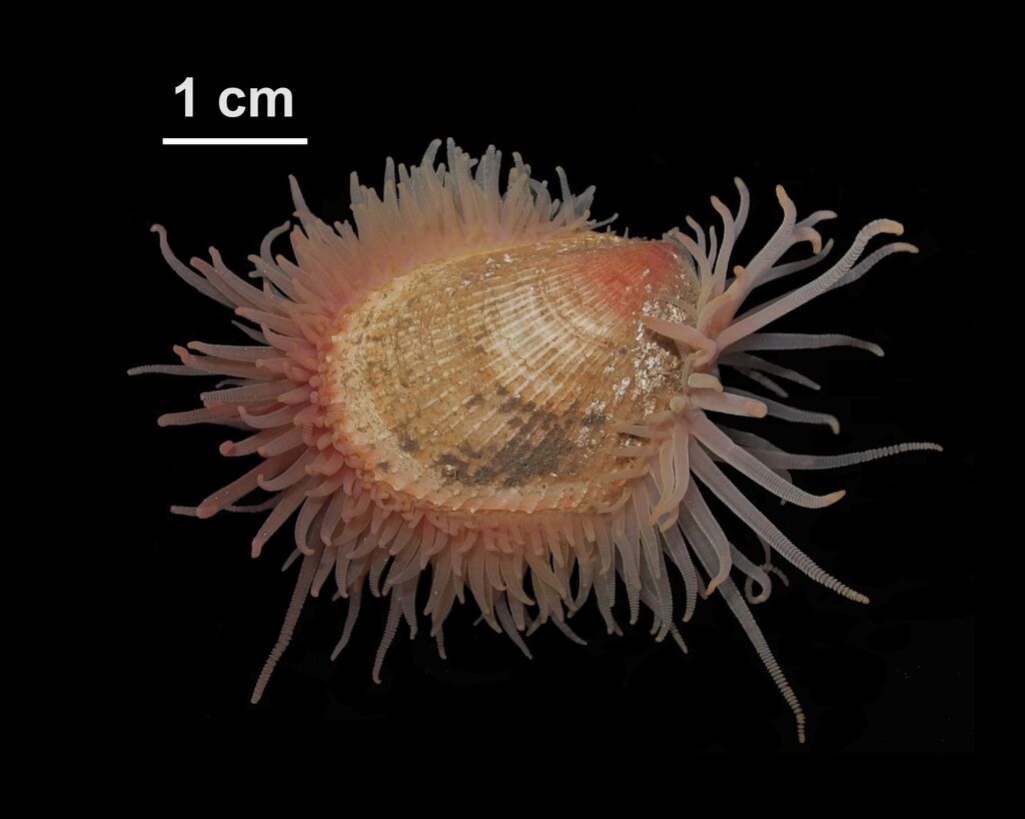
*Limariatuberculata* (Olivi, 1792). Optical photograph.

**Figure 84.**
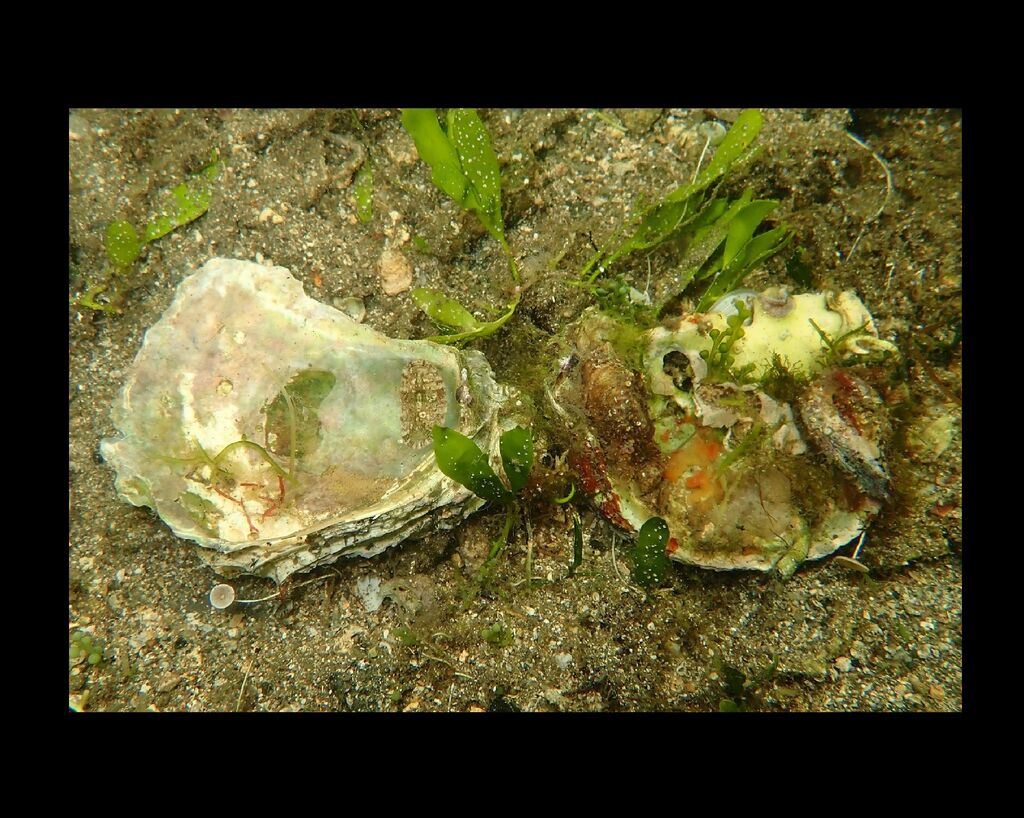
*Magallanagigas* (Thunberg, 1793). Wasted shell from the aquaculture plant. *In situ* photograph.

**Figure 86.**
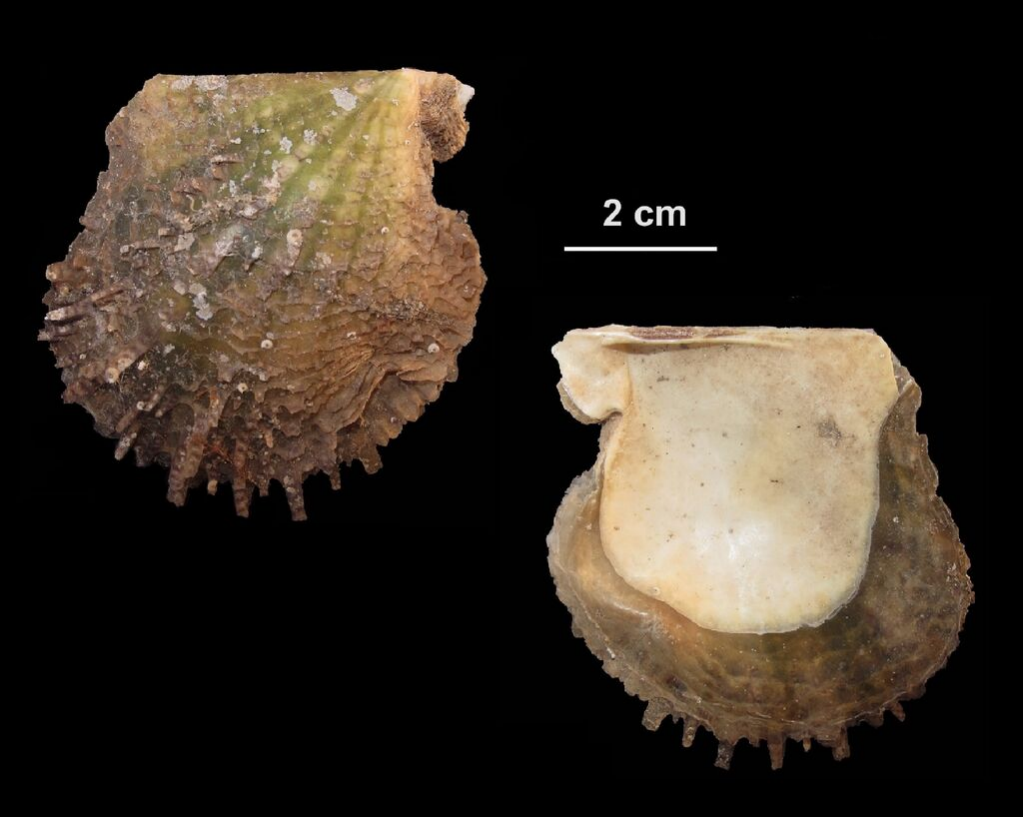
*Pinctadaradiata* (Leach, 1814). Left valve. Optical photograph.

**Figure 90.**
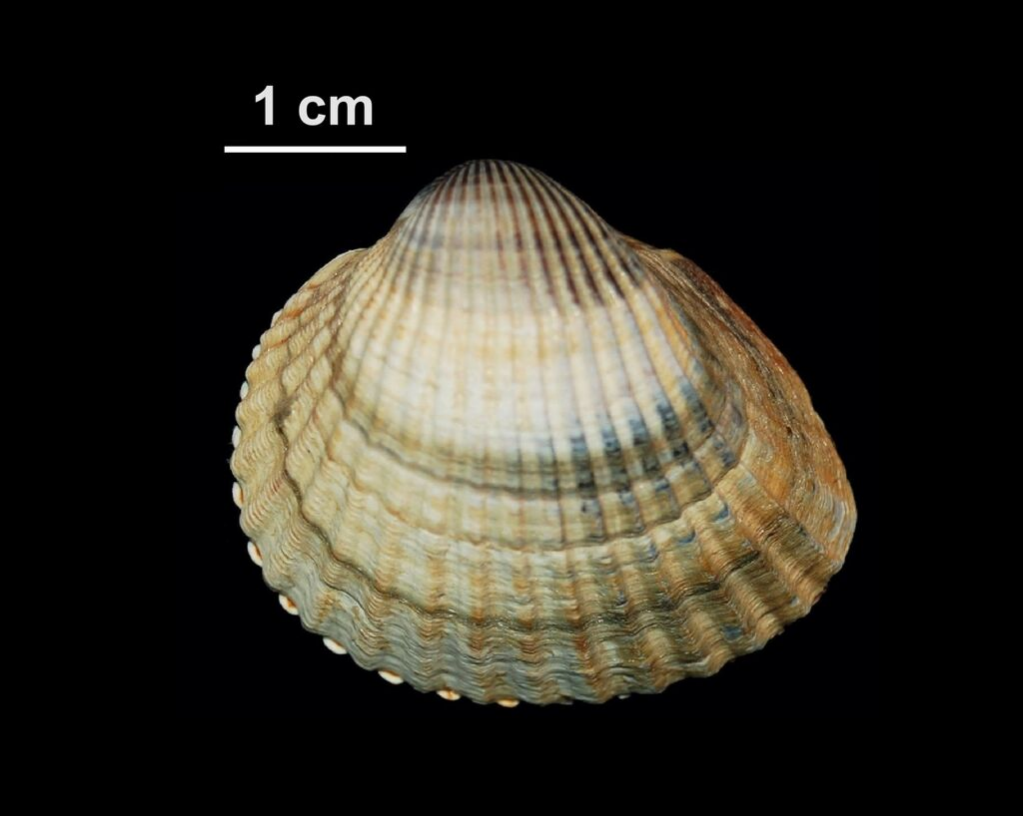
*Cerastodermaglaucum* (Bruguière, 1789). *In situ* photograph.

33A0ABB7-6CCB-5AD6-834A-3ACB5E933868

##### Materials

- **Type status:** Other material. **Occurrence:** occurrenceID: 205F26B3-A8AA-5E40-B510-BBAE70D57C26; **Location:** country: Italy; countryCode: IT; stateProvince: Sassari; locality: Island of Culuccia; verbatimLatitude: 41 11 46.79N; verbatimLongitude: 9 17 20.34E; geodeticDatum: WGS84
- **Type status:** Other material. **Occurrence:** occurrenceID: 8E72B2A3-DDBF-588E-B4E1-561BDBF79CC2; **Location:** country: Italy; countryCode: IT; stateProvince: Sassari; locality: Island of Culuccia; verbatimLatitude: 41 11 22.14N; verbatimLongitude: 9 16 59.05E; geodeticDatum: WGS84

##### Notes

Alive.

#### Venerida

7312DEA2-4F84-50CD-B536-2B24DA14761F

#### Chamidae

C8A47B37-3FDE-5EE2-B646-8BFC8CE6A716

#### Chama circinata

Monterosato, 1878

CD893CA4-1B9B-5F40-9621-954494198A86

##### Materials

- **Type status:** Other material. **Occurrence:** occurrenceID: C3BB6AA9-6BCF-531C-BCAF-44CC6997809D; **Location:** country: Italy; countryCode: IT; stateProvince: Sassari; locality: Island of Culuccia; verbatimLatitude: 41 12 28.07N; verbatimLongitude: 9 17 47.62E; geodeticDatum: WGS84

##### Notes

Alive, Fig. 98.

#### Chama gryphoides

(Linnaeus, 1758)

4AC3D63C-3FA6-519D-A3DA-E482ADF4200C

##### Materials

- **Type status:** Other material. **Occurrence:** occurrenceID: 5B390FEF-A662-525A-927E-8351EC03A872; **Location:** country: Italy; countryCode: IT; stateProvince: Sassari; locality: Island of Culuccia; verbatimLatitude: 41 11 56.04N; verbatimLongitude: 9 17 33.63E; geodeticDatum: WGS84
- **Type status:** Other material. **Occurrence:** occurrenceID: 67A9CBEE-C568-5959-982D-B4E167823294; **Location:** country: Italy; countryCode: IT; stateProvince: Sassari; locality: Island of Culuccia; verbatimLatitude: 41 12 28.07N; verbatimLongitude: 9 17 47.62E; geodeticDatum: WGS84

##### Notes

Alive, Fig. 99.

#### Pseudochama gryphina

(Lamarck, 1819)

D1FD5210-C130-5D54-AC51-46CF3535C1BD

##### Materials

- **Type status:** Other material. **Occurrence:** occurrenceID: 8306676E-9983-59CA-BAE4-088E7F0C3FBC; **Location:** country: Italy; countryCode: IT; stateProvince: Sassari; locality: Island of Culuccia; verbatimLatitude: 41 11 56.04N; verbatimLongitude: 9 17 33.63E; geodeticDatum: WGS84
- **Type status:** Other material. **Occurrence:** occurrenceID: 658D073A-55A3-50D5-A001-CBCD3245146E; **Location:** country: Italy; countryCode: IT; stateProvince: Sassari; locality: Island of Culuccia; verbatimLatitude: 41 12 42.40N; verbatimLongitude: 9 17 45.35E; geodeticDatum: WGS84

##### Notes

Alive, Fig. 100.

#### Neoleptonidae

C6724E25-1B94-5853-BAC7-215BE1AD5257

#### Neolepton sulcatulum

(Jeffreys, 1859)

1367C54B-DB1C-582F-80A2-F670C4CFE08D

##### Materials

- **Type status:** Other material. **Occurrence:** occurrenceID: 53C4BEC3-75C1-5A88-89B3-76F8BAC36C2D; **Location:** country: Italy; countryCode: IT; stateProvince: Sassari; locality: Island of Culuccia; verbatimLatitude: 41 13 17.70N; verbatimLongitude: 9 17 21.41E; geodeticDatum: WGS84

##### Notes

Shell; new record.

#### Mactridae

CB657308-77D0-5303-8D54-C39644DDDAB3

#### Mactra stultorum

(Linnaeus, 1758)

DFF31D0D-B82C-5476-9D14-EAD58CF754CB

##### Materials

- **Type status:** Other material. **Occurrence:** occurrenceID: 35B2C188-6BC4-5BEE-B7E7-0C0F903B9198; **Location:** country: Italy; countryCode: IT; stateProvince: Sassari; locality: Island of Culuccia; verbatimLatitude: 41 11 46.79N; verbatimLongitude: 9 17 20.34E; geodeticDatum: WGS84
- **Type status:** Other material. **Occurrence:** occurrenceID: 1262CF6C-CF5F-5836-A5D5-FEE7417071D8; **Location:** country: Italy; countryCode: IT; stateProvince: Sassari; locality: Island of Culuccia; verbatimLatitude: 41 11 56.04N; verbatimLongitude: 9 17 33.63E; geodeticDatum: WGS84

##### Notes

Alive.

#### Spisula subtruncata

(da Costa, 1778)

EE7D80C3-B447-5F0D-952C-8EE86F399979

##### Materials

- **Type status:** Other material. **Occurrence:** occurrenceID: 19E8D865-9AEF-5B29-874F-86A86F5ACC86; **Location:** country: Italy; countryCode: IT; stateProvince: Sassari; locality: Island of Culuccia; verbatimLatitude: 41 11 46.79N; verbatimLongitude: 9 17 20.34E; geodeticDatum: WGS84
- **Type status:** Other material. **Occurrence:** occurrenceID: 0EDBB847-CF80-524F-9B9E-5BDA8A49FC55; **Location:** country: Italy; countryCode: IT; stateProvince: Sassari; locality: Island of Culuccia; verbatimLatitude: 41 11 56.04N; verbatimLongitude: 9 17 33.63E; geodeticDatum: WGS84

##### Notes

Shell.

#### Lutraria oblonga

(Gmelin, 1791)

39834FBF-62B7-52AA-B76F-6B7E4F683CAC

##### Materials

- **Type status:** Other material. **Occurrence:** occurrenceID: 8F3E39B1-6C9D-559B-B209-C862B4DBACEE; **Location:** country: Italy; countryCode: IT; stateProvince: Sassari; locality: Island of Culuccia; verbatimLatitude: 41 11 46.79N; verbatimLongitude: 9 17 20.34E; geodeticDatum: WGS84

##### Notes

Shell.

#### Mesodesmatidae

CBDB4CAA-2183-5923-BFFE-C8F6B9775679

#### Donacilla cornea

(Poli, 1795)

8FEFBA68-9712-5839-B01F-9DE099137550

##### Materials

- **Type status:** Other material. **Occurrence:** occurrenceID: C0918744-E1D7-5176-8E6A-7A9CD1AE96CD; **Location:** country: Italy; countryCode: IT; stateProvince: Sassari; locality: Island of Culuccia; verbatimLatitude: 41 11 22.14N; verbatimLongitude: 9 16 59.05E; geodeticDatum: WGS84

##### Notes

Alive.

#### Veneridae

3A89651F-746F-5537-B9A7-840A21CFBEF3

#### Venus verrucosa

Linnaeus, 1758

D387033C-801F-5905-9592-DE3E4F40643B

##### Materials

- **Type status:** Other material. **Occurrence:** occurrenceID: D1B51D8C-8A6E-52D4-AEA1-3F9FEAE71584; **Location:** country: Italy; countryCode: IT; stateProvince: Sassari; locality: Island of Culuccia; verbatimLatitude: 41 11 46.79N; verbatimLongitude: 9 17 20.34E; geodeticDatum: WGS84
- **Type status:** Other material. **Occurrence:** occurrenceID: 0D979544-8DE3-5966-BBB8-4C866FA5E83F; **Location:** country: Italy; countryCode: IT; stateProvince: Sassari; locality: Island of Culuccia; verbatimLatitude: 41 11 22.14N; verbatimLongitude: 9 16 59.05E; geodeticDatum: WGS84

##### Notes

Alive, Fig. 101.

#### Dosinia exoleta

(Linnaeus, 1758)

B823BD3E-785C-5901-92B3-3C111EA61B66

##### Materials

- **Type status:** Other material. **Occurrence:** occurrenceID: 3AE2B5D3-90FC-5341-87D9-3CC14BCDCB86; **Location:** country: Italy; countryCode: IT; stateProvince: Sassari; locality: Island of Culuccia; verbatimLatitude: 41 13 17.70N; verbatimLongitude: 9 17 21.41E; geodeticDatum: WGS84

##### Notes

Alive.

#### Dosinia lupinus

(Linnaeus, 1758)

7CC7136B-F424-524C-80DF-DDB6941DFE8E

##### Materials

- **Type status:** Other material. **Occurrence:** occurrenceID: E7957C35-90AE-5774-A4AD-24003CBFFBC1; **Location:** country: Italy; countryCode: IT; stateProvince: Sassari; locality: Island of Culuccia; verbatimLatitude: 41 12 28.07N; verbatimLongitude: 9 17 47.62E; geodeticDatum: WGS84
- **Type status:** Other material. **Occurrence:** occurrenceID: 48D5F633-8BDE-5350-AA81-7F2114FD6DCD; **Location:** country: Italy; countryCode: IT; stateProvince: Sassari; locality: Island of Culuccia; verbatimLatitude: 41 11 22.14N; verbatimLongitude: 9 16 59.05E; geodeticDatum: WGS84

##### Notes

Alive, Fig. 102.

#### Irus irus

(Linnaeus, 1758)

457F98C9-1564-5DDD-BA5A-1C18D9FDD81E

##### Materials

- **Type status:** Other material. **Occurrence:** occurrenceID: FAFDB4C1-4083-5F32-B6BF-645F9CFCEC91; **Location:** country: Italy; countryCode: IT; stateProvince: Sassari; locality: Island of Culuccia; verbatimLatitude: 41 13 17.70N; verbatimLongitude: 9 17 21.41E; geodeticDatum: WGS84

##### Notes

Shell.

#### Polititapes aureus

(Gmelin, 1791)

0EC03814-C664-5ECE-BB68-D53981243907

##### Materials

- **Type status:** Other material. **Occurrence:** occurrenceID: 86B5BD6C-0A36-502B-864D-321911D9806A; **Location:** country: Italy; countryCode: IT; stateProvince: Sassari; locality: Island of Culuccia; verbatimLatitude: 41 11 46.79N; verbatimLongitude: 9 17 20.34E; geodeticDatum: WGS84
- **Type status:** Other material. **Occurrence:** occurrenceID: 14FD896F-5820-537F-848E-FF5FFEDE1EE2; **Location:** country: Italy; countryCode: IT; stateProvince: Sassari; locality: Island of Culuccia; verbatimLatitude: 41 11 28.32N; verbatimLongitude: 9 16 44.50E; geodeticDatum: WGS84
- **Type status:** Other material. **Occurrence:** occurrenceID: CFB2BFA2-20BB-5F20-9322-5A0E12D9D366; **Location:** country: Italy; countryCode: IT; stateProvince: Sassari; locality: Island of Culuccia; verbatimLatitude: 41 11 22.14N; verbatimLongitude: 9 16 59.05E; geodeticDatum: WGS84

##### Notes

Alive, Fig. 103.

#### Polititapes lucens

(Locard, 1886)

C1C76619-393A-5672-85F9-6D66A6CF0EC0

##### Materials

- **Type status:** Other material. **Occurrence:** occurrenceID: EFE046EF-0A2B-531C-9C82-C0C3942BC1F5; **Location:** country: Italy; countryCode: IT; stateProvince: Sassari; locality: Island of Culuccia; verbatimLatitude: 41 13 17.70N; verbatimLongitude: 9 17 21.41E; geodeticDatum: WGS84

##### Notes

Alive.

#### Ruditapes decussatus

(Linnaeus, 1758)

CF8D6471-6A99-52BB-91C9-988175D11B1D

##### Materials

- **Type status:** Other material. **Occurrence:** occurrenceID: F56E3108-CA5A-5D9C-8453-A47EC499B39B; **Location:** country: Italy; countryCode: IT; stateProvince: Sassari; locality: Island of Culuccia; verbatimLatitude: 41 11 46.79N; verbatimLongitude: 9 17 20.34E; geodeticDatum: WGS84
- **Type status:** Other material. **Occurrence:** occurrenceID: A9D512B2-3E5A-5C28-94CC-5F752E0BEC8D; **Location:** country: Italy; countryCode: IT; stateProvince: Sassari; locality: Island of Culuccia; verbatimLatitude: 41 11 22.14N; verbatimLongitude: 9 16 59.05E; geodeticDatum: WGS84

##### Notes

Alive.

#### Callista chione

(Linnaeus, 1758)

16B7B765-9669-5470-A455-6255A0E3B369

##### Materials

- **Type status:** Other material. **Occurrence:** occurrenceID: 6BBA1D87-092A-5607-A3BB-5F4EA8D82E0E; **Location:** country: Italy; countryCode: IT; stateProvince: Sassari; locality: Island of Culuccia; verbatimLatitude: 41 11 46.79N; verbatimLongitude: 9 17 20.34E; geodeticDatum: WGS84
- **Type status:** Other material. **Occurrence:** occurrenceID: 12035329-C7ED-5ABA-A66E-1FA1782D1FC8; **Location:** country: Italy; countryCode: IT; stateProvince: Sassari; locality: Island of Culuccia; verbatimLatitude: 41 11 56.04N; verbatimLongitude: 9 17 33.63E; geodeticDatum: WGS84

##### Notes

Shell.

#### Trapezidae

9E09FF1C-1084-569B-AF50-14EED6473D0E

#### Coralliophaga lithophagella

(Lamarck, 1819)

77DA8AE7-0E6D-511F-9E57-5344F018F8F8

##### Materials

- **Type status:** Other material. **Occurrence:** occurrenceID: 34D3AD86-64C4-5386-85D0-3F8E31711388; **Location:** country: Italy; countryCode: IT; stateProvince: Sassari; locality: Island of Culuccia; verbatimLatitude: 41 13 17.70N; verbatimLongitude: 9 17 21.41E; geodeticDatum: WGS84

##### Notes

Shell.

#### Ungulinidae

00FD2EB4-DD5B-584A-A839-EBBB38080778

#### Microstagon trigonum

(Scacchi, 1835)

7563FC28-A272-5CD9-8802-0BFC2B15A9C8

##### Materials

- **Type status:** Other material. **Occurrence:** occurrenceID: 88116113-3427-52DE-A45C-69E08B5AB949; **Location:** country: Italy; countryCode: IT; stateProvince: Sassari; locality: Island of Culuccia; verbatimLatitude: 41 13 17.70N; verbatimLongitude: 9 17 21.41E; geodeticDatum: WGS84

##### Notes

Shell.

#### Adapedonta

97CF6AE2-8463-5BFC-A796-5CDAFBD18F2C

#### Solenidae

60BB6ECF-2873-5531-97F5-E352A462CC79

#### Solen marginatus

Pulteney, 1799

07C360B1-AC76-5DFF-AB55-B23D11302163

##### Materials

- **Type status:** Other material. **Occurrence:** occurrenceID: 615BFB03-B6F9-58BA-8B59-7E730EE9F69C; **Location:** country: Italy; countryCode: IT; stateProvince: Sassari; locality: Island of Culuccia; verbatimLatitude: 41 11 46.79N; verbatimLongitude: 9 17 20.34E; geodeticDatum: WGS84
- **Type status:** Other material. **Occurrence:** occurrenceID: B5B63BCF-9A3E-5911-BE5D-98698CAD119D; **Location:** country: Italy; countryCode: IT; stateProvince: Sassari; locality: Island of Culuccia; verbatimLatitude: 41 12 28.07N; verbatimLongitude: 9 17 47.62E; geodeticDatum: WGS84

##### Notes

Shell.

#### Pharidae

29C62A8E-9A3A-570F-9CC9-A88B3AD4D3D9

#### Ensis minor

(Chenu, 1843)

B6F2465E-11CD-513B-9606-9ECA41ED8E60

##### Materials

- **Type status:** Other material. **Occurrence:** occurrenceID: A4F1C82D-AB0E-5D68-A932-7AC1C451A802; **Location:** country: Italy; countryCode: IT; stateProvince: Sassari; locality: Island of Culuccia; verbatimLatitude: 41 11 46.79N; verbatimLongitude: 9 17 20.34E; geodeticDatum: WGS84
- **Type status:** Other material. **Occurrence:** occurrenceID: DB90F455-1563-5992-820B-B6A7D8A2DA20; **Location:** country: Italy; countryCode: IT; stateProvince: Sassari; locality: Island of Culuccia; verbatimLatitude: 41 11 56.04N; verbatimLongitude: 9 17 33.63E; geodeticDatum: WGS84

##### Notes

Shell.

#### Myida

B484247D-C1B4-52BA-9502-0C94BC1BE829

#### Myidae

6FB14723-51BF-577F-B348-A75279938DCA

#### Sphenia binghami

W. Turton, 1822

A80D1B85-B442-504F-87CA-23BA3E9810EC

##### Materials

- **Type status:** Other material. **Occurrence:** occurrenceID: 6E344CCB-2BCC-528C-BEA9-38E396102810; **Location:** country: Italy; countryCode: IT; stateProvince: Sassari; locality: Island of Culuccia; verbatimLatitude: 41 13 17.70N; verbatimLongitude: 9 17 21.41E; geodeticDatum: WGS84

##### Notes

Shell.

#### Anomalodesmata

51062C7A-7ACF-54D7-9988-376DCA1489E8

#### Thraciidae

6034B449-8C76-5FD0-82A3-C7D5EB733F5D

#### Thracia convexa

(W. Wood, 1815)

07BA0031-14CB-5280-A55B-F667E903BB0B

##### Materials

- **Type status:** Other material. **Occurrence:** occurrenceID: 02B6C8CD-3EE3-5262-9874-2931848A3728; **Location:** country: Italy; countryCode: IT; stateProvince: Sassari; locality: Island of Culuccia; verbatimLatitude: 41 13 17.70N; verbatimLongitude: 9 17 21.41E; geodeticDatum: WGS84
- **Type status:** Other material. **Occurrence:** occurrenceID: 0CD9D27A-1A8C-5A9F-AD6E-4F7CFC6CBDF3; **Location:** country: Italy; countryCode: IT; stateProvince: Sassari; locality: Island of Culuccia; verbatimLatitude: 41 11 46.79N; verbatimLongitude: 9 17 20.34E; geodeticDatum: WGS84

##### Notes

Shell.

#### Thracia phaseolina

(Lamarck, 1818)

ADA979F4-F087-5B86-8A80-755A3E873895

##### Materials

- **Type status:** Other material. **Occurrence:** occurrenceID: EF4BD90A-100F-5E03-981A-5C4109FBD1AC; **Location:** country: Italy; countryCode: IT; stateProvince: Sassari; locality: Island of Culuccia; verbatimLatitude: 41 13 17.70N; verbatimLongitude: 9 17 21.41E; geodeticDatum: WGS84

##### Notes

Shell.

#### Thracia villosiuscula

(MacGillivray, 1827)

8289F2E7-07EF-5180-9A54-3C28BE0F469A

##### Materials

- **Type status:** Other material. **Occurrence:** occurrenceID: 8BA528E2-A7BB-5B70-BDBC-13B1983163EF; **Location:** country: Italy; countryCode: IT; stateProvince: Sassari; locality: Island of Culuccia; verbatimLatitude: 41 13 17.70N; verbatimLongitude: 9 17 21.41E; geodeticDatum: WGS84
- **Type status:** Other material. **Occurrence:** occurrenceID: 0F579E75-78D0-597B-894E-D2F2BDB7702C; **Location:** country: Italy; countryCode: IT; stateProvince: Sassari; locality: Island of Culuccia; verbatimLatitude: 41 11 46.79N; verbatimLongitude: 9 17 20.34E; geodeticDatum: WGS84

##### Notes

Alive.

## Analysis

**Figure 104.**
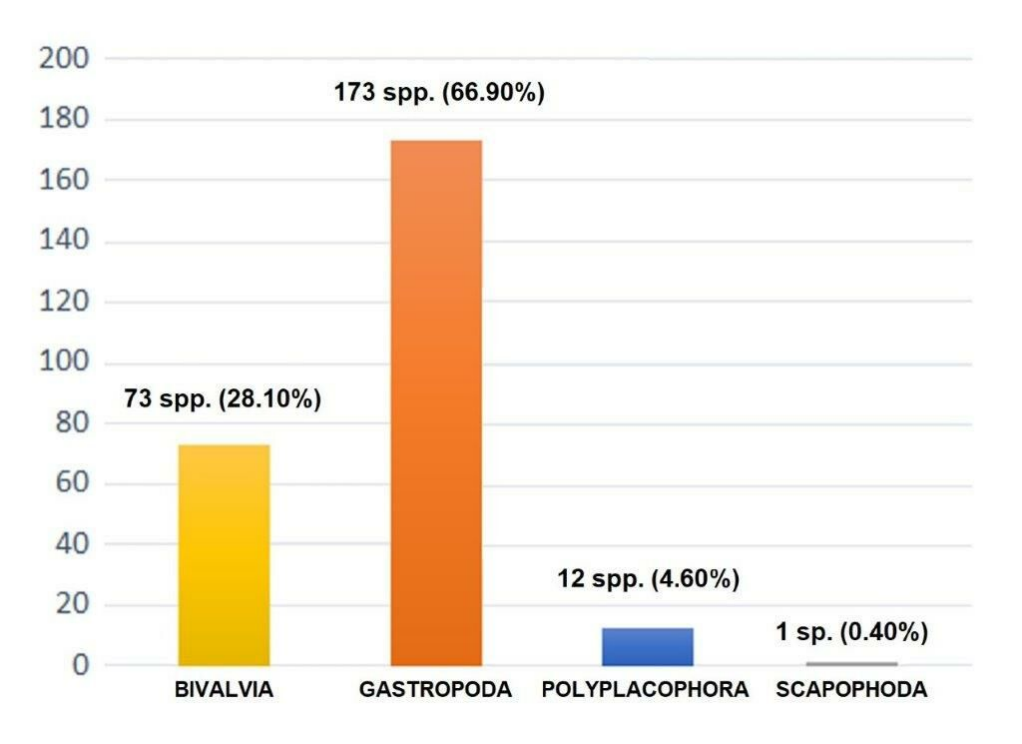
Composition of the marine molluscs of the Culuccia Peninsula.

Marine molluscs were collected in 15 sampling sites alongside Culuccia Peninsula (Fig. 1). In total, 114 live-collected specimens and 142 empty shells were sampled, belonging to 256 species, 176 genera, 99 families, 31 orders and four classes. Composition of the marine molluscs of the Culuccia Peninsula resulted as follows: 73 species of Bivalvia (61 genera, 28 families, 13 orders), 173 species of Gastropoda (107 genera, 63 families, 14 orders), 12 species of Polyplacophora (7 genera, 7 families, 3 orders), one species of Scapophoda. Amongst the four classes recorded, gastropods were the most represented (66.90% of the total number species), followed by bivalves (28.10%), polyplacophorans (4.60%) and scapophods (0.40%) (Fig. 104).

## Discussion

Amongst the species recorded, some taxa are worthy of mention being threatened or rarely collected/observed. This is the case of the fan mussel (or nobel pen shell) *Pinnanobilis* (Linnaeus, 1758), the largest bivalve of the Mediterranean Sea, an endemic species protected under the European Council since 1992 [Annex IV of EU Habitats Directive 92/43/EEC (EEC 1992)]. Currently, *P.nobilis* has become a threatened and vulnerable species and is legally protected under Annex II of the Barcelona Convention (SPA/BD Protocol 1995), Annex IV of the EU Habitats Directive (EU Habitats Directive 2007) and the Spanish Catalogue of Threatened Species (Category: Vulnerable, Royal Decree 139/2011). During the 20^th^ century, populations of this bivalve have greatly declined because of human activities, accidental killing by boat anchoring, fishing nets and trawlers and, in the last years, parasites' infection. In 2017-2018, a mass mortality event (MME) affecting this pen shell was detected along the Spanish coasts (Vázquez-Luis et al. 2017) and, later on, at many sites across the whole Mediterranean Sea. This MME was associated to a haplosporidian (*Haplosporidiumpinnae*) parasite (Darriba 2017, Catanese et al. 2018, Tiscar et al. 2022) and/or to mycobacterial (*Mycobacterium* sp.) infection (Carella et al. 2019, Lattos et al. 2020), in some cases with the addition of a concomitant vibriosis (*Vibrio* spp.) (Künili et al. 2021) and other opportunistic pathogens like *Perkinsus* sp. (Carella et al. 2019). All the *P.nobilis* observed at Culuccia were dead, confirming the observation that the MME also occurred in northern Sardinia (Scarpa et al. 2020). The limpet *P.ferruginea* is a Mediterranean endemic species listed amongst the most threatened marine macro-invertebrates occurring in this Basin (European Council Directive 92/43/EEC on the Conservation of Natural Habitat of Wild Fauna and Flora, 1992). Although having the 'strict protection’ status, this species is still collected by fishermen and unaware tourists. Cossu et al. (2022) have examined the genetic diversity of this limpet in northern Sardinian populations, as well as their spatial distribution. According to their study, the closest geographical spot where this endangered species was recorded is La Maddalena Island. We have observed *P.ferruginea* in two sampling sites at Culuccia (SS #4 and #8).

Four NIS, two gastropods and two bivalves have been recorded. The cephalaspidean *Lamprohaminoeacyanomarginata* (Heller & Thompson, 1983) is a lessepsian species and, in spite of the fact that it is missing in “The New Checklist of the Italian Fauna” (Renda et al. 2022, herein abbreviated as CIFmM) for the Sardinia waters, this non-indigenous seaslug has been reported since 2016 by Azzola et al. (2022) from the Tavolara-Punta Coda Cavallo Marine Protected Area. With this investigation, its presence has been confirmed and its range has been slightly enlarged to the north-western portion of Sardinia. The ragged sea hare *Bursatellaleachii* Blainville, 1817 is nowadays widespread in the whole Mediterranean Sea (Selfati et al. 2017) and it was reported from the Gulf of Olbia since 2010 (Doneddu 2010). We confirm the presence of this aplysiid in north-western Sardinia. The Pacific oyster *Magallanagigas* (Thunberg, 1793) is one of the most important cultured bivalves worldwide (Tran et al. 2022 and references therein). In a suspended aquaculture plant located in the southern part of the Coluccia Fjiord (SS#14), this oyster has been reared since 2019. Interestingly, in the coastline (SS#13-15) facing the oyster farm, abundant populations of both *Ostreaedulis* and *O.stentina* have been recorded on rocky substrates, while no individuals of *M.gigas* have been found. *Pinctadaradiata* (Leach, 1814) is an alien bivalve that is progressively expanding its Mediterranean distribution. According to several authors (Barbieri et al. 2015, Gavrilović et al. 2017, Petović and Mačić 2017, Ballesteros et al. 2020, Png-Gonzalez et al. 2021), the Mediterranean specimens of this alien Margaritidae belong to the subspecies *P.imbricataradiata*. In particular, Barbieri et al. (2015) have described in details its invasion history in the Mediterranean and its rapid spread. Another subspecies, *P.imbricatafucata*, has been recorded by Scuderi et al. (2019) and Cunningham Aparicio and Mulero Méndez (2021) in the Mediterranean Sea, claiming a second (independent) alien *Pinctada* colonisation. In “The New Checklist of the Italian Fauna” (Renda et al. 2022), both *P.imbricataradiata* and *P.imbricatafucata* are reported in Italian waters. A note reporting the first record for Sardinia of living specimens of this allochthonous species, denominated as *P.radiata*, have recently been submitted for publication (M. Garzia, personal communication). Interestingly, a valve of this species has been collected in SS#10, confirming the presence of this taxon in northern Sardinia. Amongst the four classes recorded, gastropods were the most represented (68.83% of the total number species), followed by bivalves (25.91%), polyplacophorans (4.86%) and scapophods (0.40%) (Fig. 104). This is in line with the prevalence of rocky substrates, including pebbles and gravel, over sandy-muddy shores, around the Peninsula of Culuccia (Table 1). Considering only the four classes Bivalvia, Gastropoda, Polyplacophora and Scapophoda, according to the CIFmM (Renda et al. 2022), a total of 1,283 molluscan species have been listed of the Marine Sector 2 (MS2, northern Tyrrhenian, including the sea around Corsica and Sardinia). Amongst the 26 species of Polyplacophora present in the MS2, 10 are confirmed, while *Callochitonseptemvalvis* (Montagu, 1803) (Fig. 2) and *Leptochitonasellus* (Gmelin, 1791) are new records for Sector 2. In this Mediterranean sector, out of 960 gastropod species, 160 have been confirmed and nine species must be added to the CIFmM-MS2; of these, seven are: *Rissoaaartseni* Verduin, 1985; *Crisillasemistriata* (Montagu, 1808); *Manzoniacrassa* (Kanmacher, 1798); *Pusiagranum* (Forbes, 1844); *Gibberulacaelata* (Monterosato, 1878); *Partheninaalesii* Micali, Nofroni & Perna, 2012 and *Megastomiaconoidea* (Brocchi, 1814). The two additional taxa to be added are the Pleurobranchidae *Berthellaplumula* (Montagu, 1803) and *Berthellinaaurantiaca* (Risso, 1818), albeit they have been previously reported by Trainito and Doneddu (2016) from the Marine Protected Area Tavolara-Punta Coda Cavallo (north-eastern Sardinia). In Marine Sector 2, 286 bivalves are listed, 68 have been confirmed, while three resulted in being new records: *Gregariellasemigranata* (Reeve, 1858), *Neoleptonsulcatulum* (Jeffreys, 1859) and *Cerastodermaglaucum* (Bruguière, 1789).

In conclusion, with this investigation, 16 molluscan species can be added to the CIFmM of the Marine Sector 2 as new records [2 polyplacophorans, 10 gastropods (including *L.cyanomarginata*), 4 bivalves (including *P.radiata*)]. As stressed by the Marine Strategy Framework Directive (2008/56/EC), molluscs are amongst the best descriptors of the effects of climate change and human impact on marine ecosystems, as well as to evaluate the status of protected areas. Therefore, long-term biodiversity monitoring and observation are needed to improve our knowledge of biodiversity changes that can occur in marine areas. This work is an important addition to local and regional biodiversity knowledge and a baseline for future biodiversity monitoring in northern Sardinia.

## Supplementary Material

8459E20A-6216-5032-A091-622E1CF8E41A 10.3897/BDJ.12.e115051.suppl1 Supplementary material 1 Haliotistuberculata
Data type Movie Brief description *Haliotistuberculata* crawling on rocky substrate. File: oo_917202.MOV https://binary.pensoft.net/file/917202 P. Mariottini

45408256-A3E7-5C95-A2ED-2E557387E62C 10.3897/BDJ.12.e115051.suppl2 Supplementary material 2 Felimarepicta
Data type Movie Brief description *Felimarepicta* crawling on a rock. File: oo_916100.MOV https://binary.pensoft.net/file/916100 P. Mariottini

494055DD-E214-5EA2-BEA0-FE3011792720 10.3897/BDJ.12.e115051.suppl3 Supplementary material 3 Cratenaperegrina
Data type Movie Brief description *Cratenaperegrina* feeding on a colony of *Eudendrium* sp. File: oo_916071.mov https://binary.pensoft.net/file/916071 P. Mariottini

A6D32520-EB1E-5936-900B-78DE7505E8DC 10.3897/BDJ.12.e115051.suppl4 Supplementary material 4 Bursatellaleachii
Data type Movie Brief description *Bursatellaleachii* crawling on muddy substrate covered by the seaweed *Caulerpaprolifera*. File: oo_916068.mov https://binary.pensoft.net/file/916068 G. Mariottini

F10FF0EB-1E79-54B1-97BA-673CF77AA738 10.3897/BDJ.12.e115051.suppl5 Supplementary material 5 Petaliferapetalifera
Data type Movie Brief description *Petaliferapetalifera* crawling on encrusted rock surface. File: oo_916064.MOV https://binary.pensoft.net/file/916064 P. Mariottini

9AB64BBA-A8E1-59A8-A0E8-376C7438907B 10.3897/BDJ.12.e115051.suppl6 Supplementary material 6 Limariahians
Data type Movie Brief description *Limariahians* on a sandy substrate covered by *Caulerpaprolifera*. File: oo_916073.mov https://binary.pensoft.net/file/916073 P. Mariottini

0A2A57CA-72D3-5A8E-9594-B8DCCDB171F9 10.3897/BDJ.12.e115051.suppl7 Supplementary material 7 Limariatuberculata
Data type Movie Brief description *Limariatuberculata* on a sandy substrate. File: oo_916081.mov https://binary.pensoft.net/file/916081 P. Mariottini

## Figures and Tables

**Figure 3.**
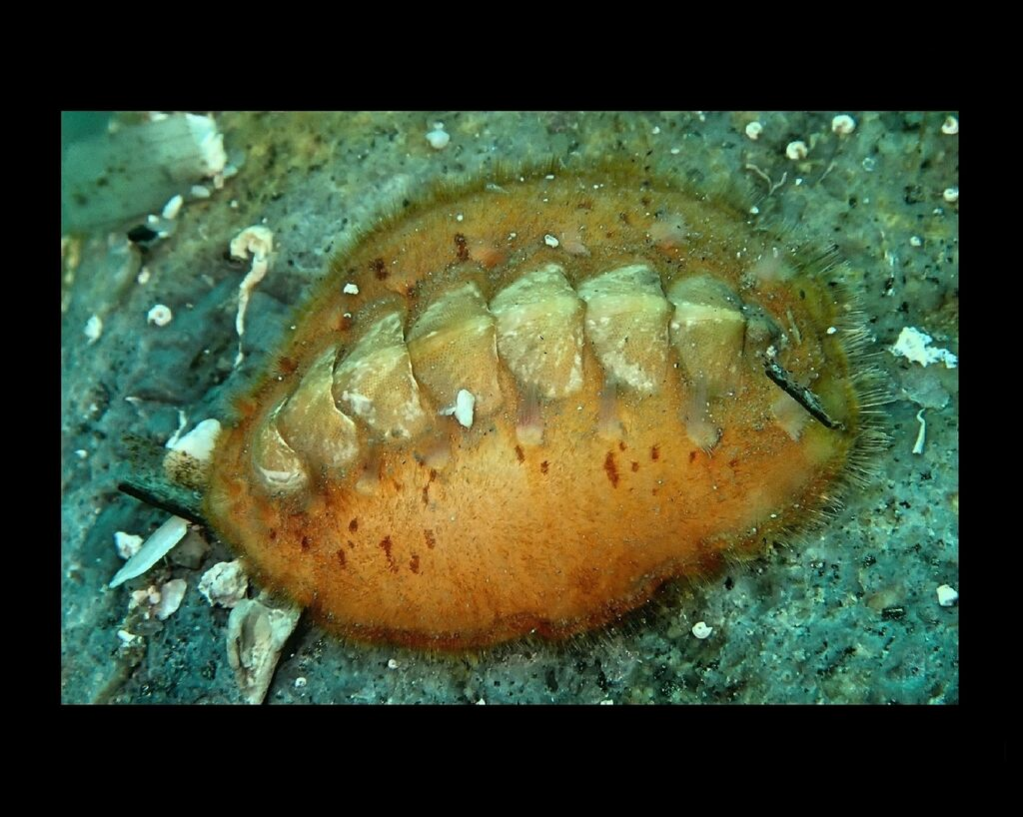
*Acanthochitonafascicularis* (Linnaeus, 1767). *In situ* photograph.

**Figure 4.**
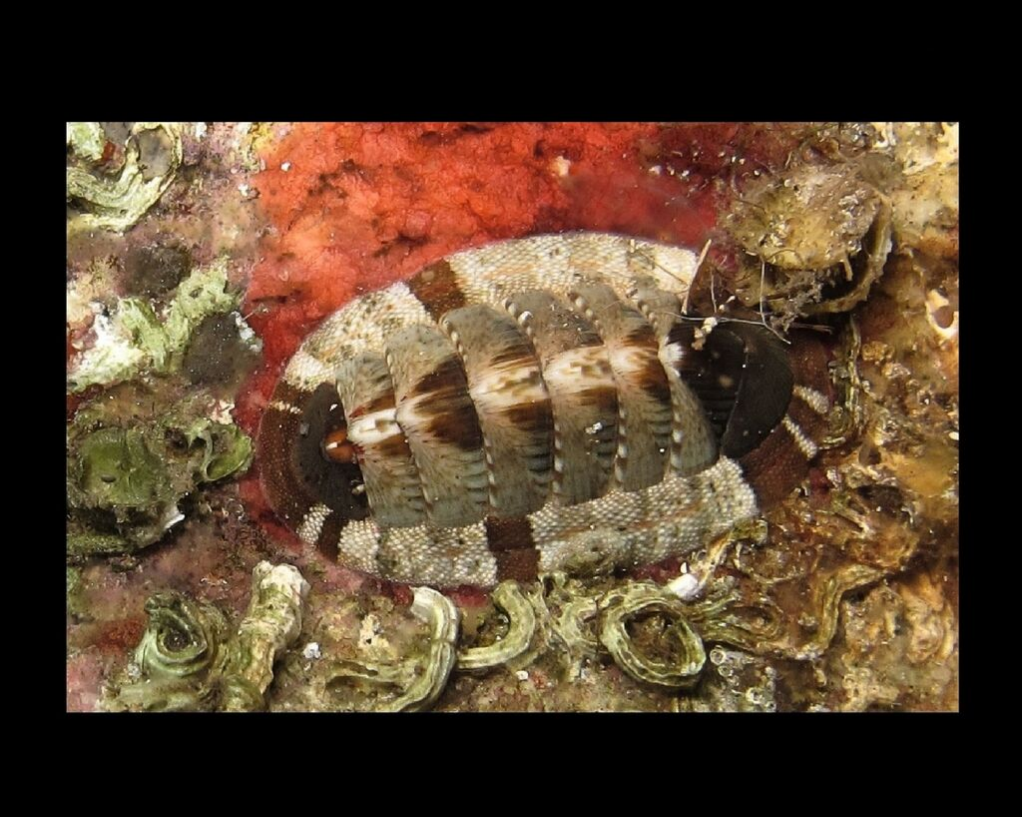
*Rhyssoplaxolivacea* Spengler, 1797. *In situ* photograph.

**Figure 5.**
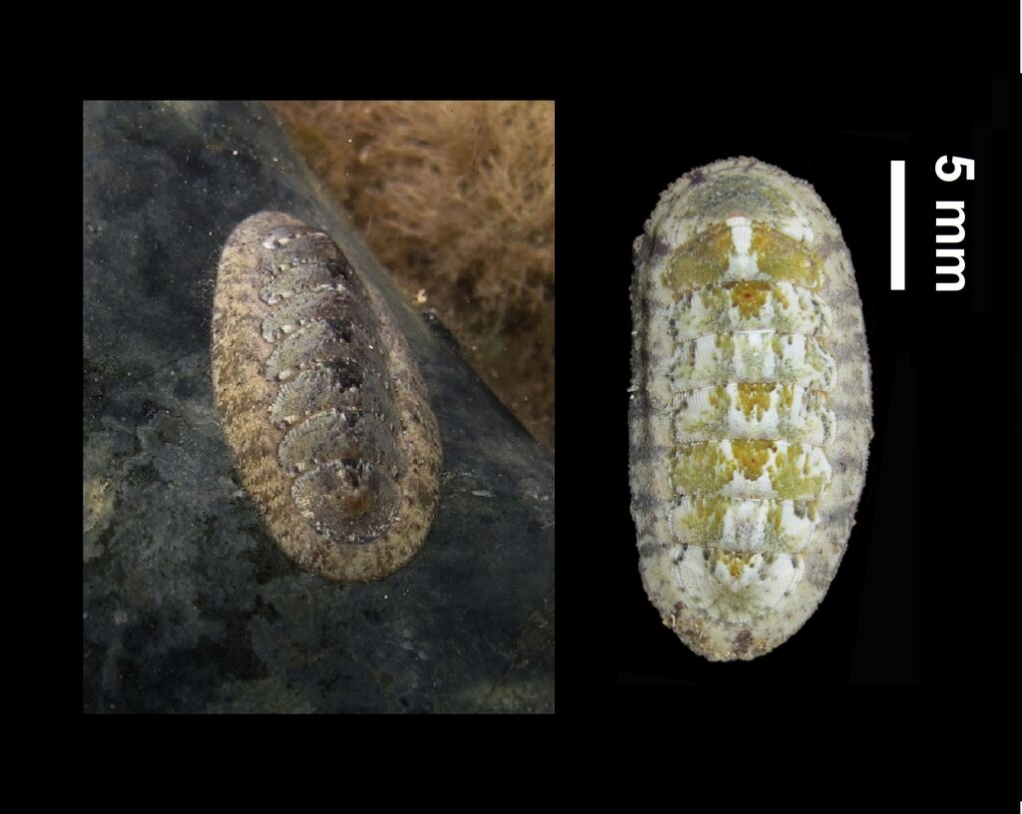
*Ischnochitonrissoi* (Payraudeau, 1826). On the left, *in situ* photograph; on the right, optical photograph.

**Figure 8.**
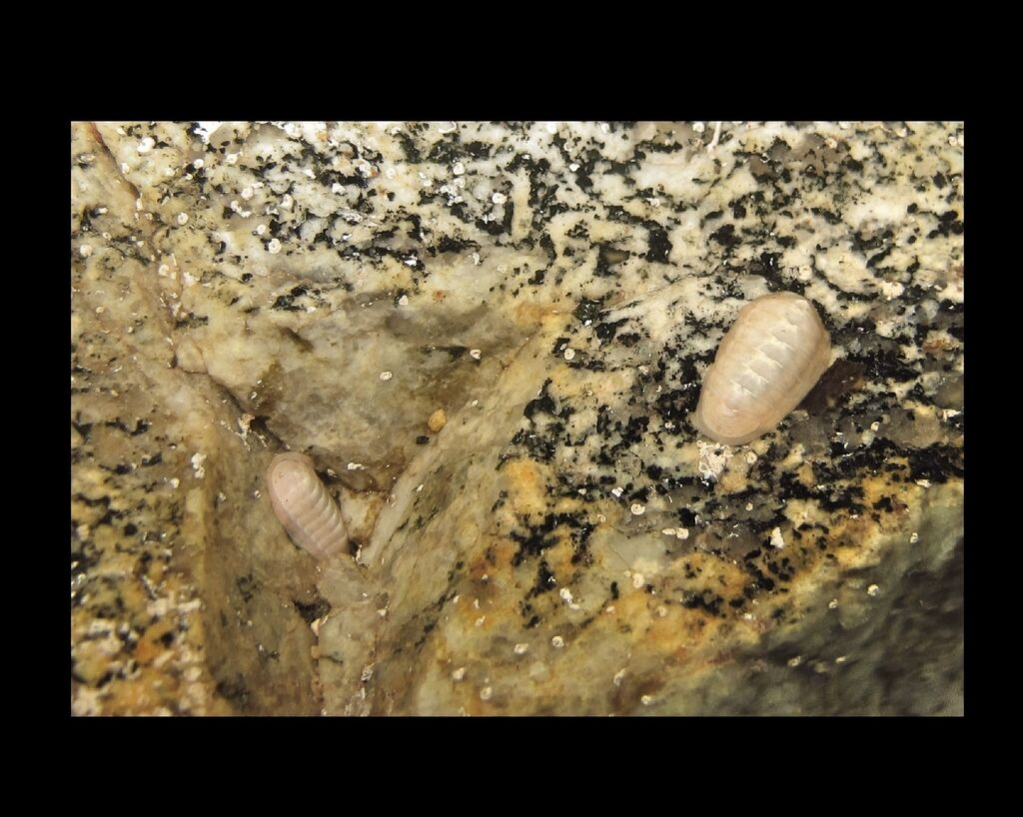
*Leptochitonalgesirensis* (Capellini, 1859). *In situ* photograph.

**Figure 9.**
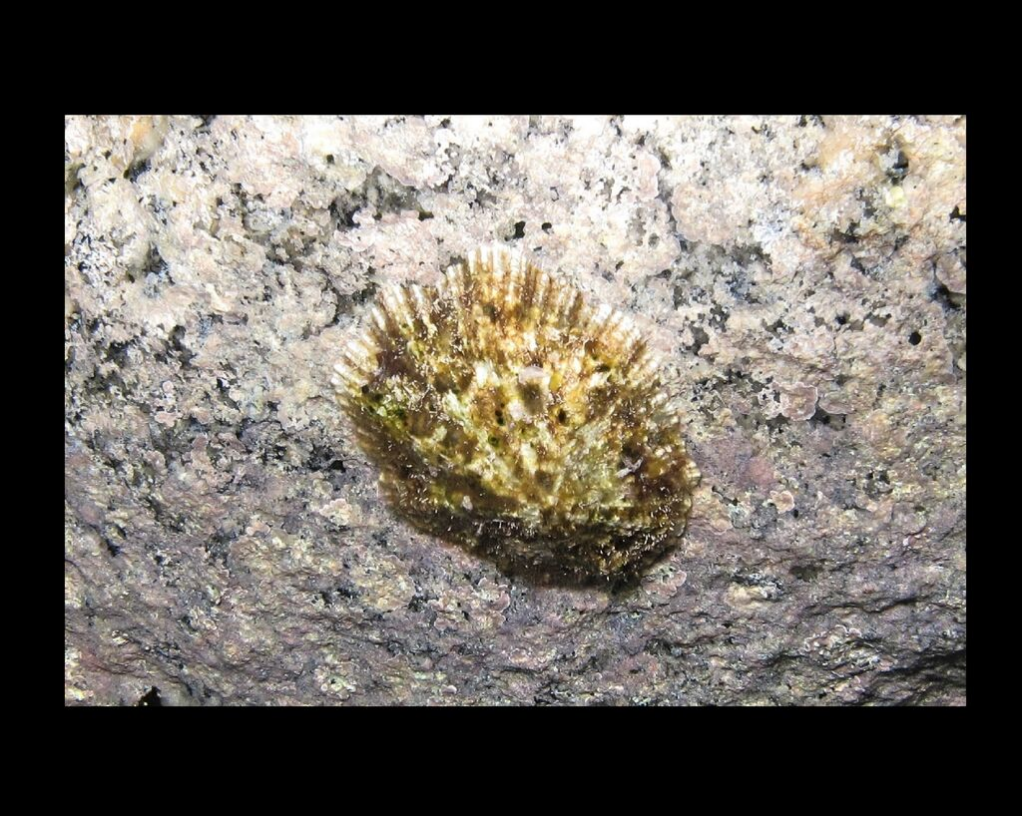
*Patellacaerulea* Linnaeus, 1758. *In situ* photograph.

**Figure 10.**
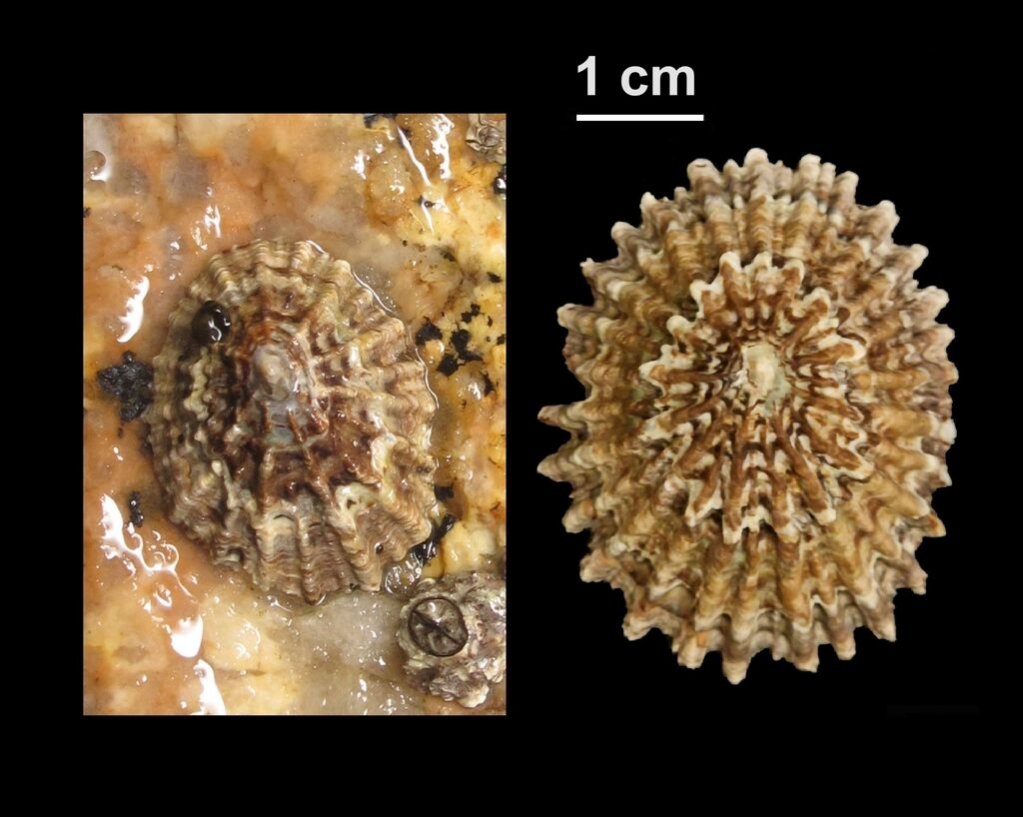
*Patellaferruginea* Gmelin, 1791. On the left, *in situ* photograph; on the right, optical photograph of a shell.

**Figure 11.**
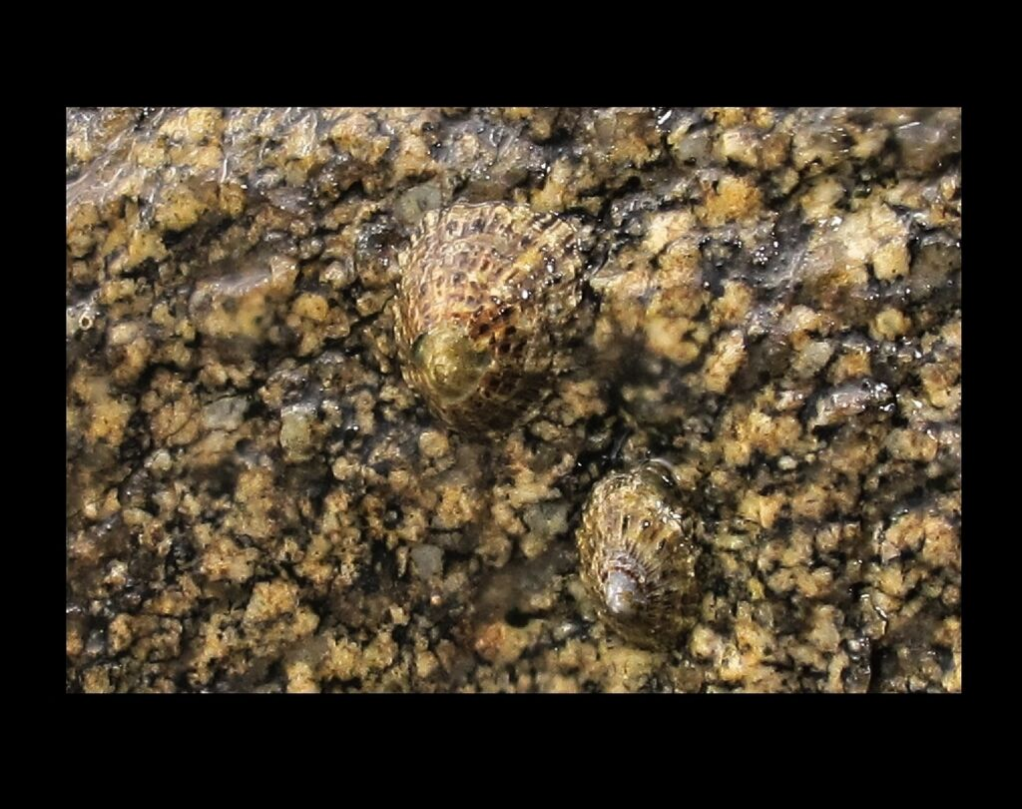
*Patellarustica* Linnaeus, 1758. *In situ* photograph.

**Figure 12.**
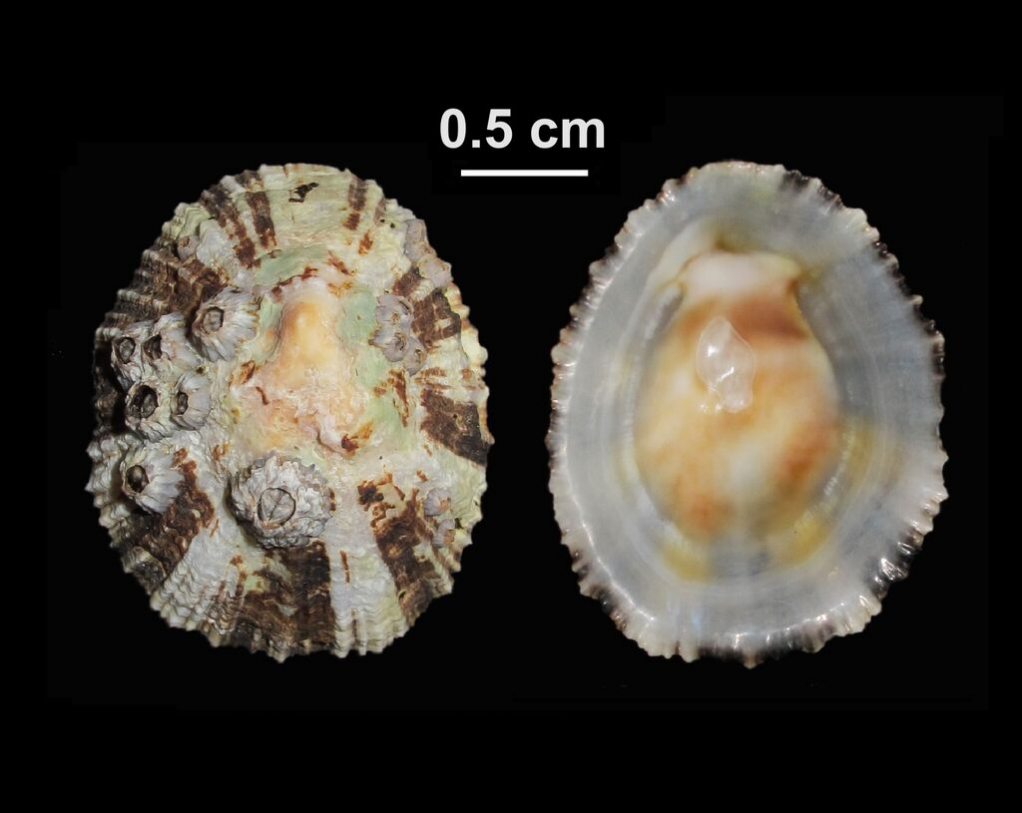
*Patellaulyssiponensis* Gmelin, 1791. Dorsal and internal views. Optical photograph.

**Figure 14.**
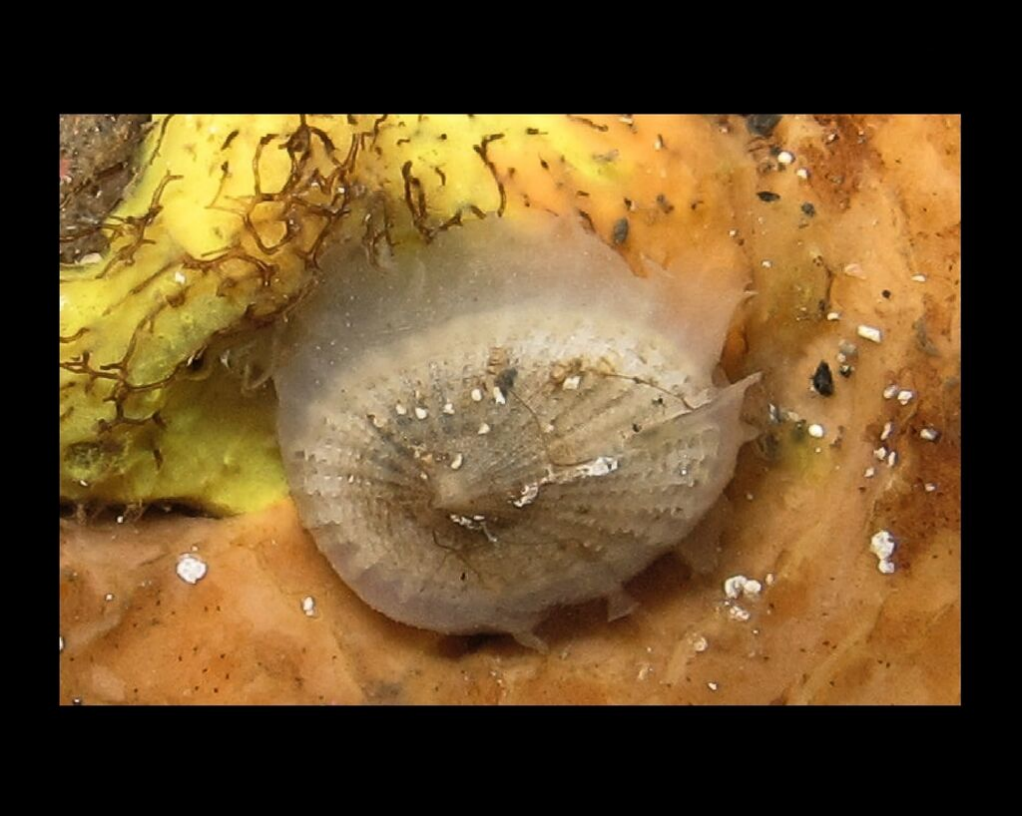
*Emarginulahuzardii* Payraudeau, 1826. *In situ* photograph.

**Figure 15.**
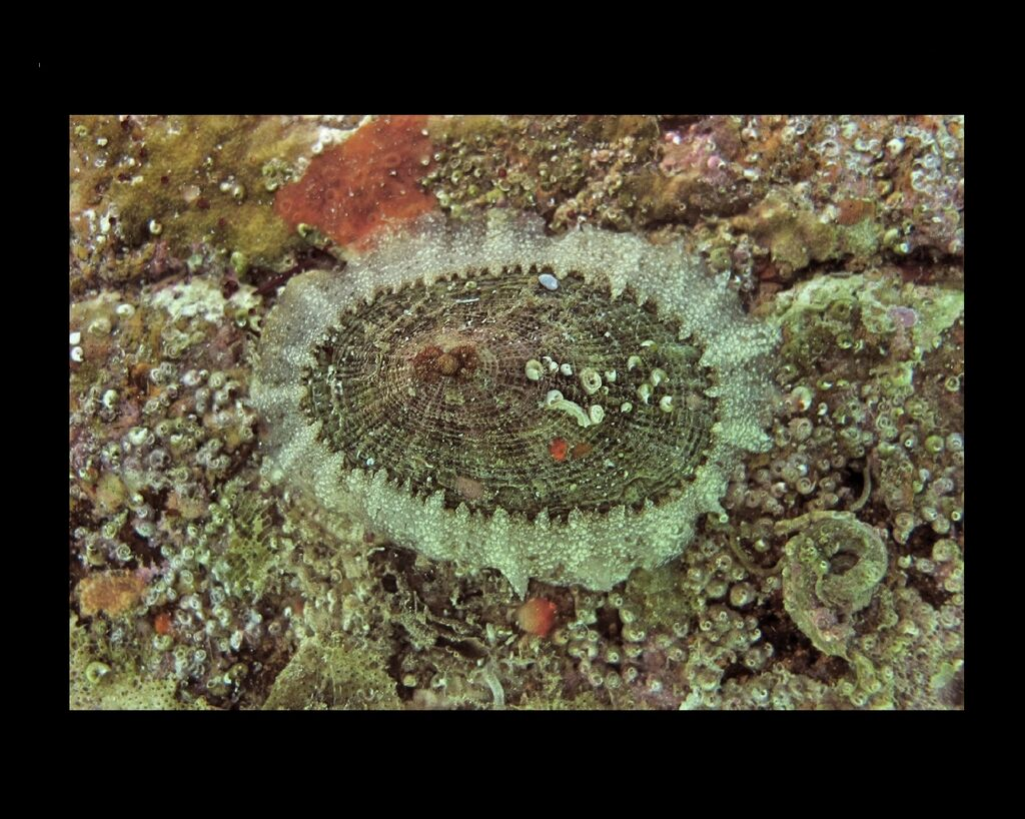
*Diodoragraeca* O.G. Costa 1830. *In situ* photograph.

**Figure 16.**
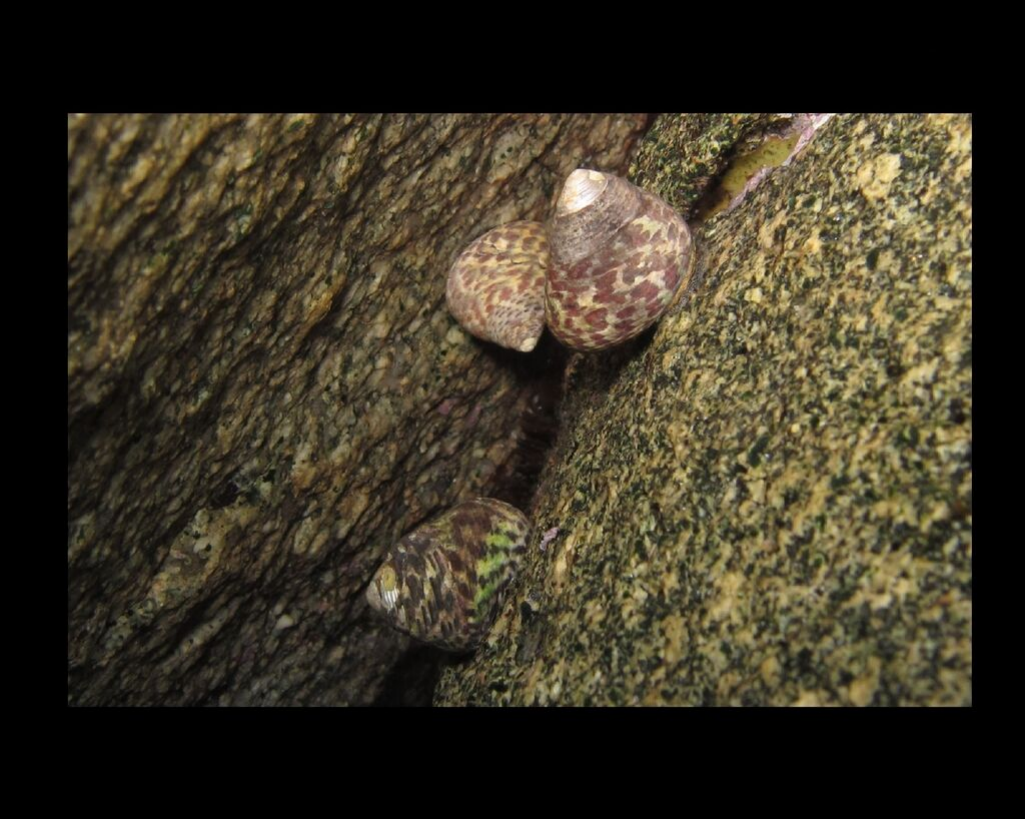
*Phorcusturbinatus* (Born, 1778). *In situ* photograph.

**Figure 17.**
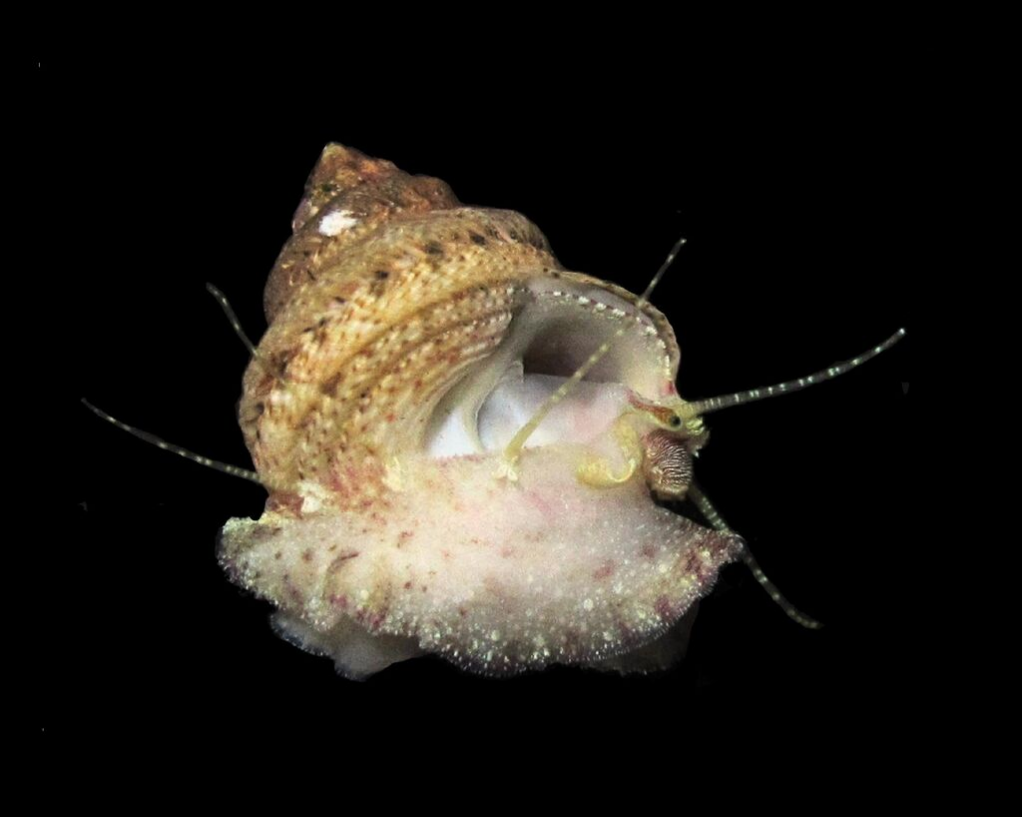
*Gibbulafanulum* (Gmelin, 1791). Optical photograph.

**Figure 18.**
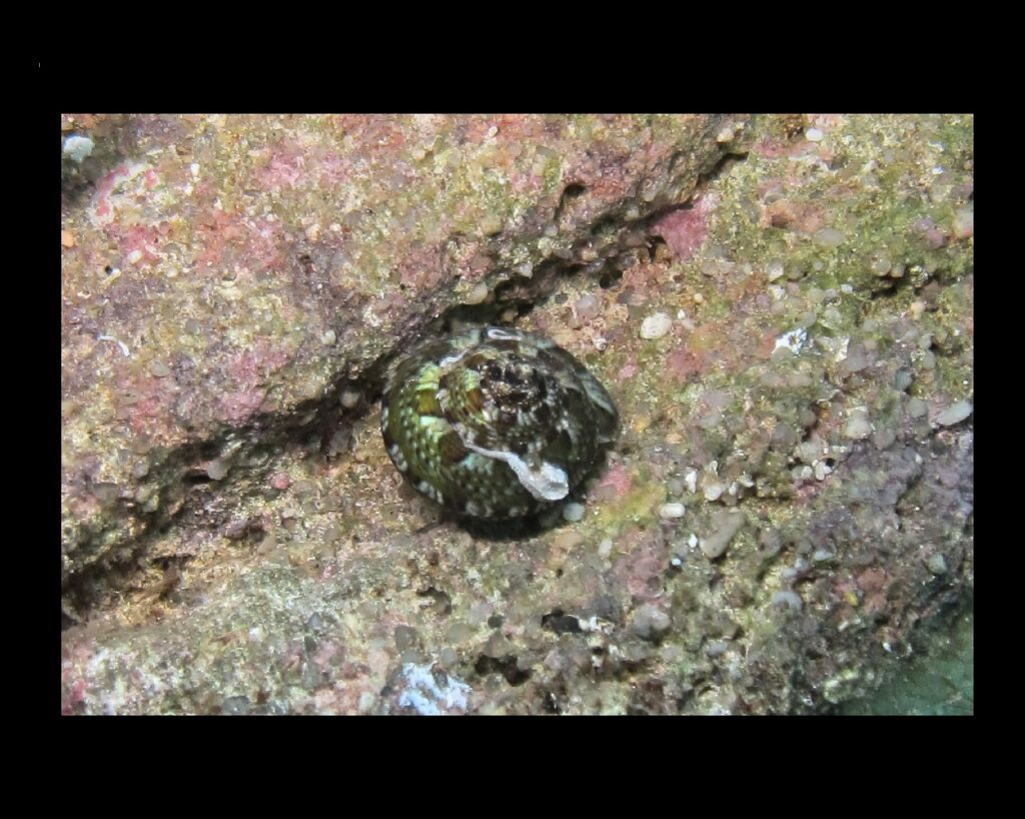
*Gibbulaumbilicaris* (Linnaeus, 1758). *In situ* photograph.

**Figure 19.**
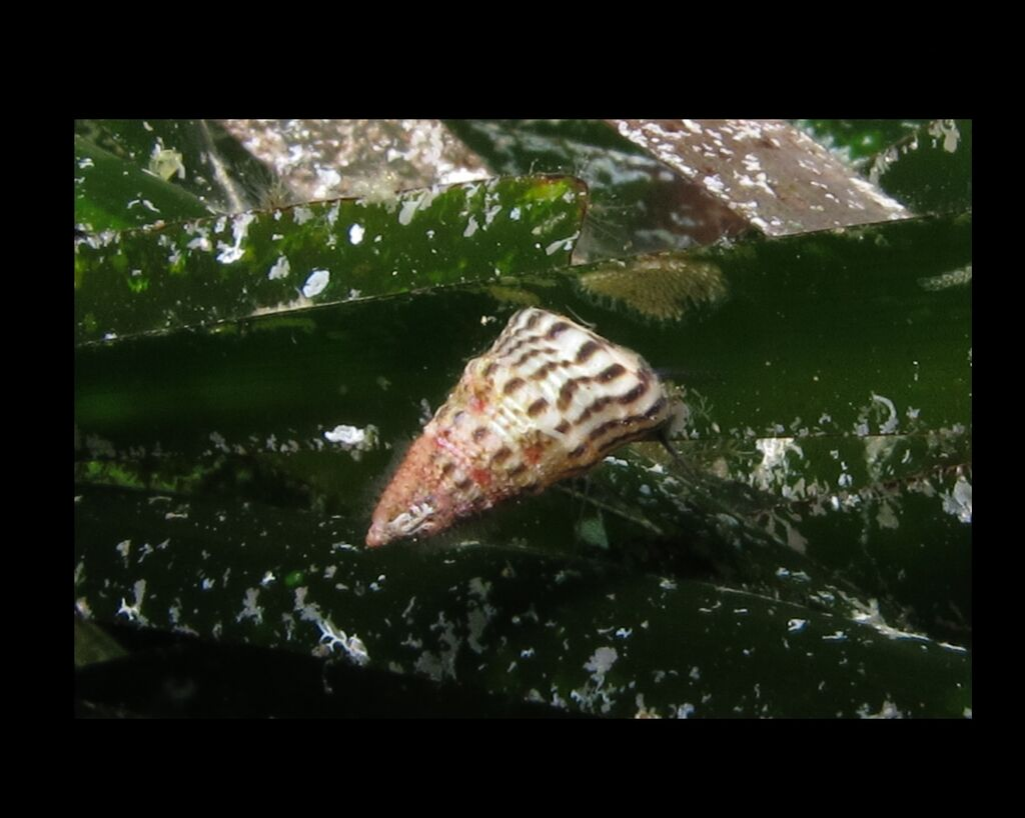
*Jujubinusexasperatus* (Pennant, 1777). *In situ* photograph.

**Figure 20.**
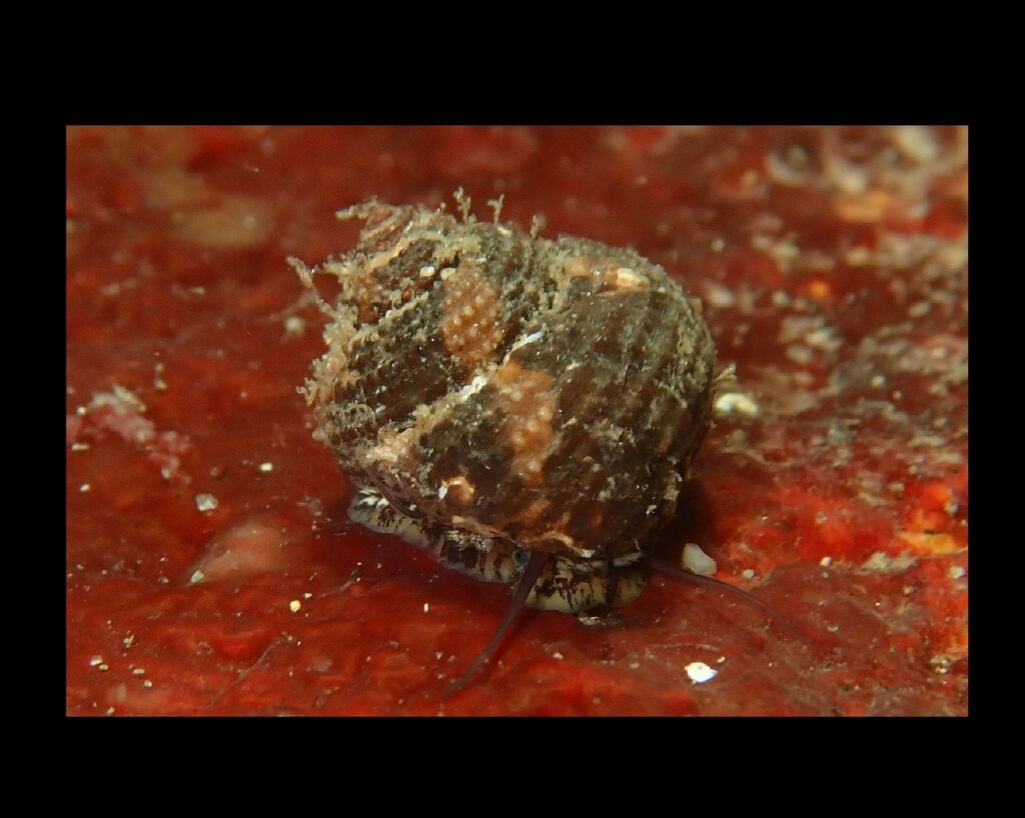
*Clanculuscruciatus* (Linnaeus, 1758). *In situ* photograph.

**Figure 21.**
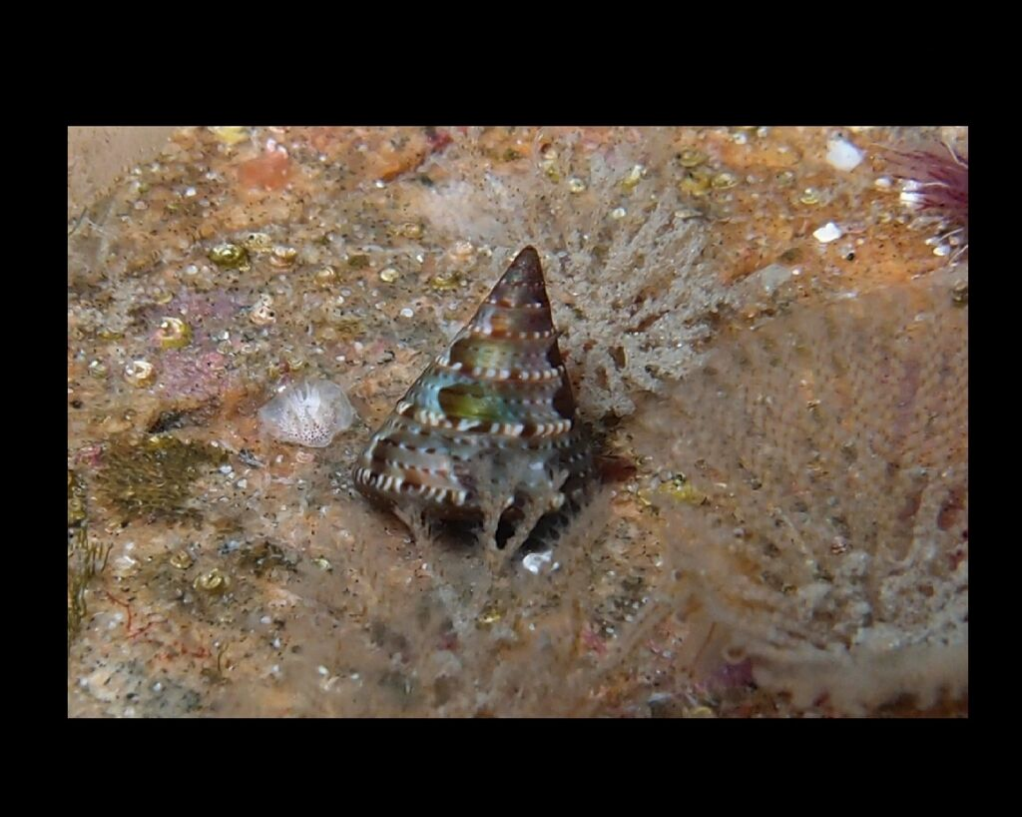
*Calliostomalaugieri* (Payraudeau, 1826). *In situ* photograph.

**Figure 22.**
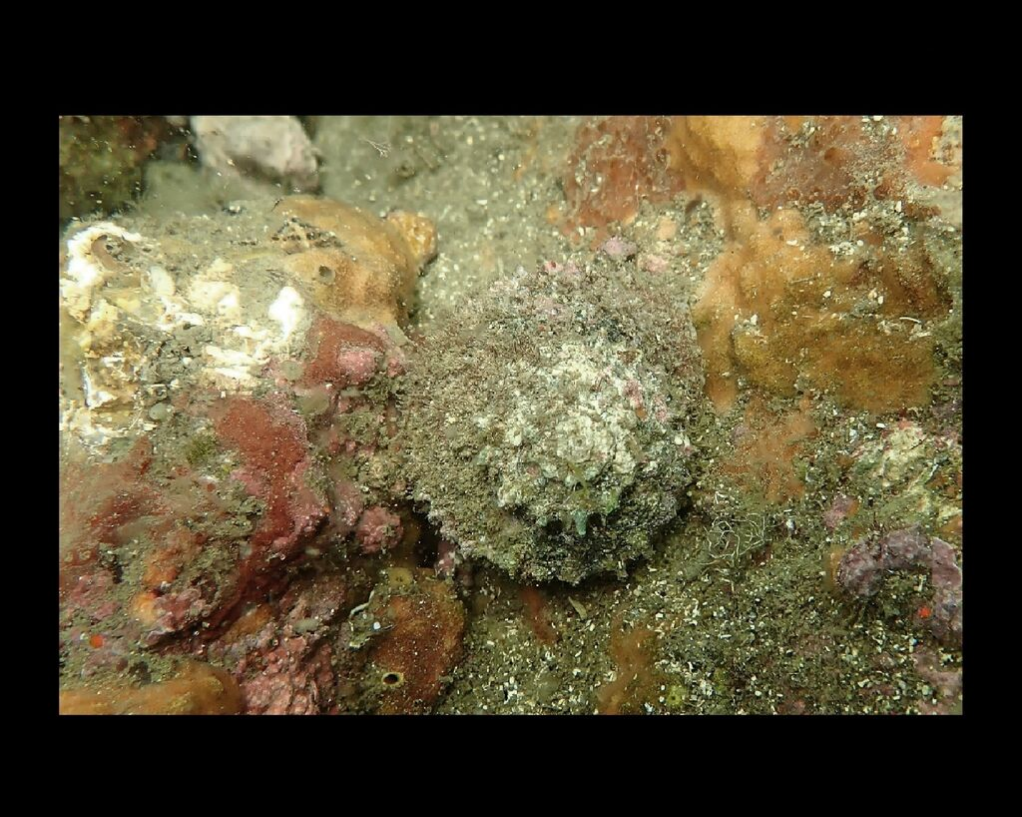
*Bolmarugosa* (Linnaeus 1767). *In situ* photograph.

**Figure 23.**
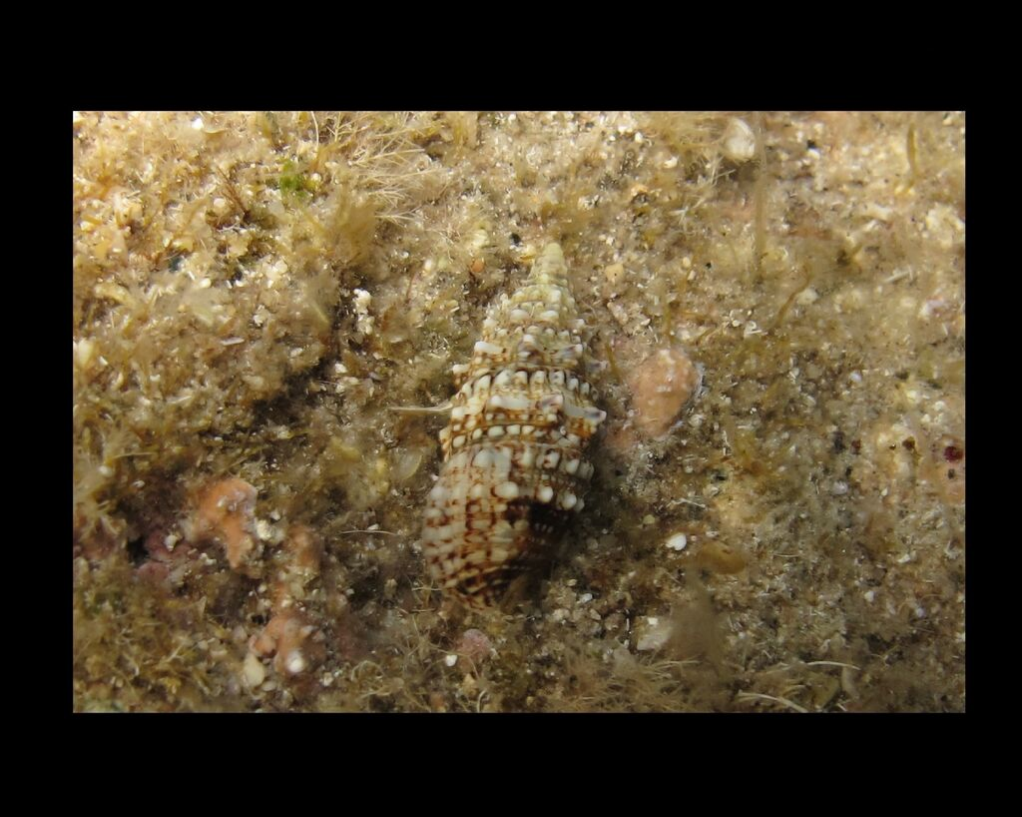
*Cerithiumrenovatum* Monterosato, 1884. *In situ* photograph.

**Figure 24.**
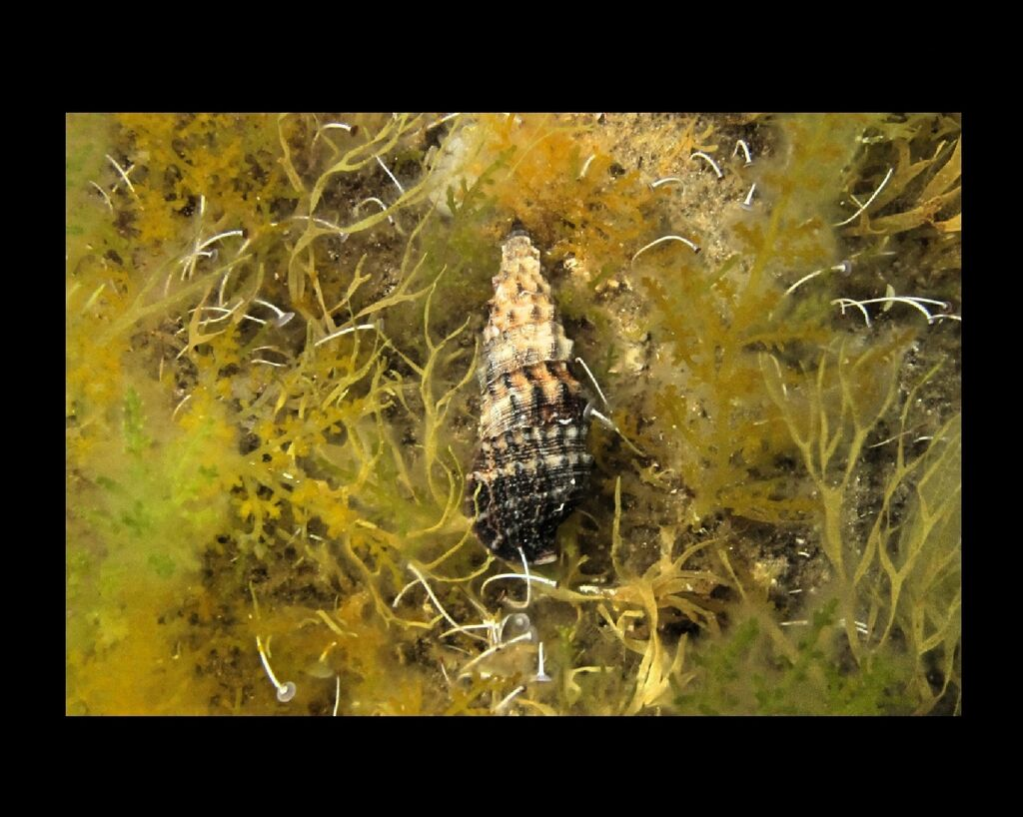
*Cerithiumvulgatum* Bruguiere, 1792. *In situ* photograph.

**Figure 25.**
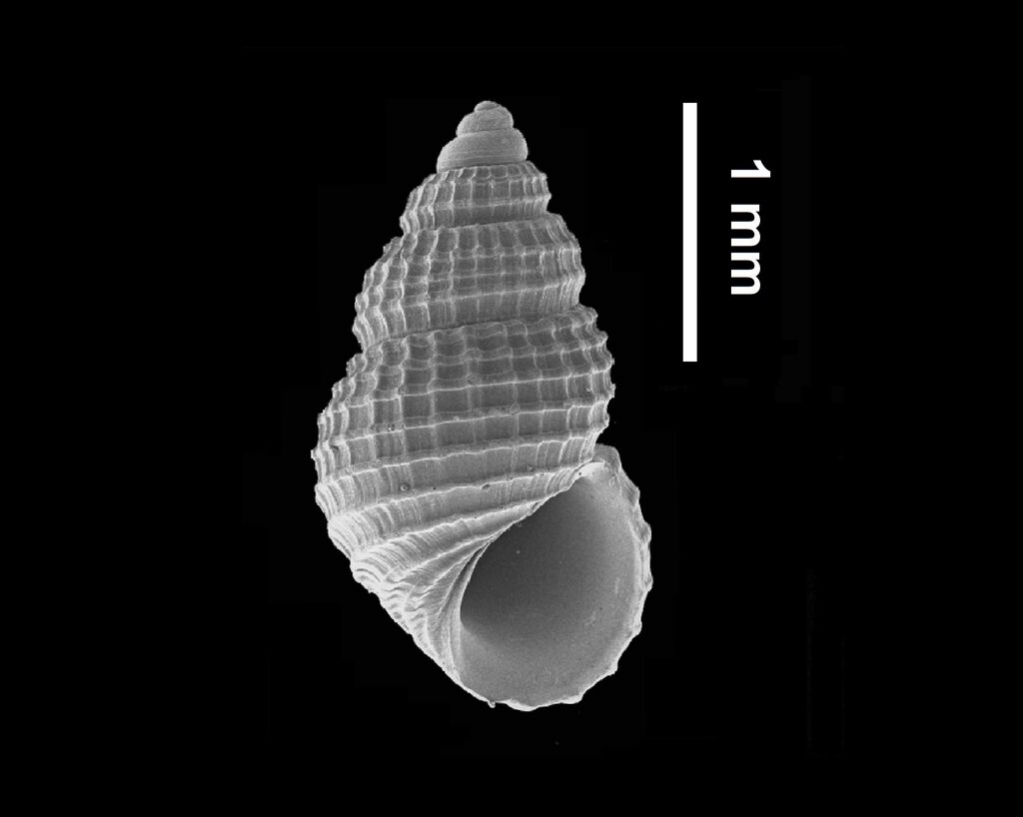
*Alvaniabeanii* (Hanley, 1844). SEM photograph.

**Figure 26.**
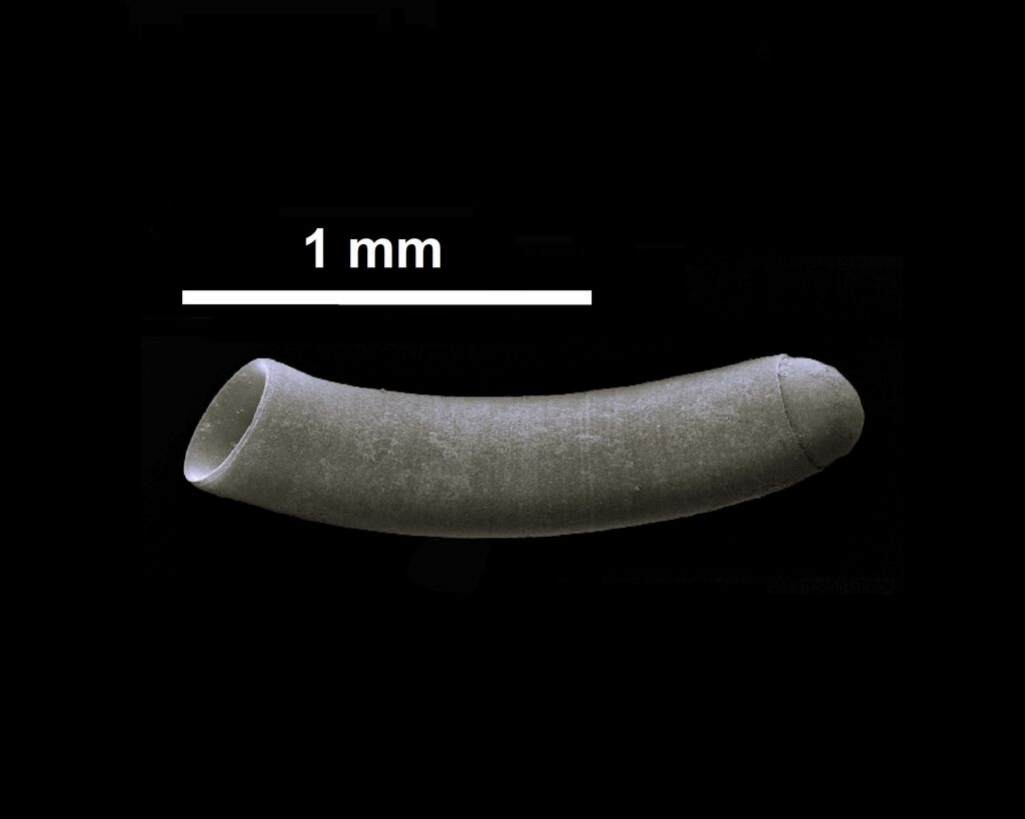
*Caecumsubannulatum* de Folin, 1870. SEM photograph.

**Figure 27.**
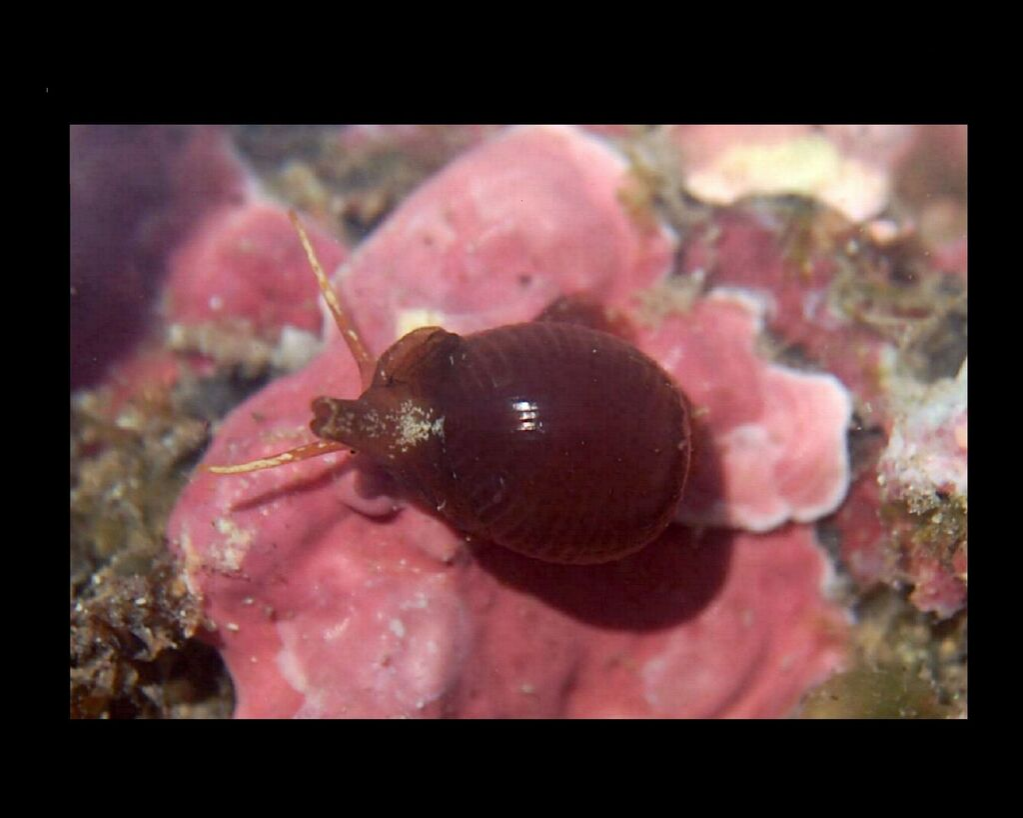
*Triviamediterranea* (Risso, 1826). *In situ* photograph.

**Figure 28.**
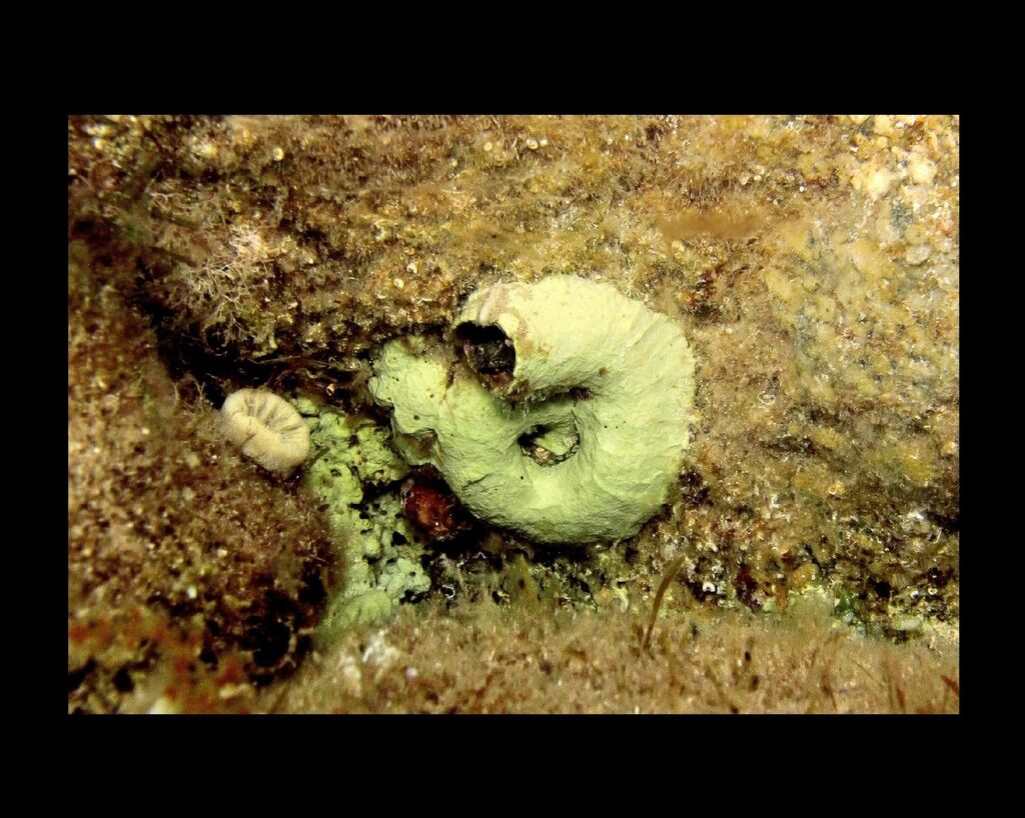
*Vermetusgranulatus* (Gravenhorst, 1831). *In situ* photograph.

**Figure 29.**
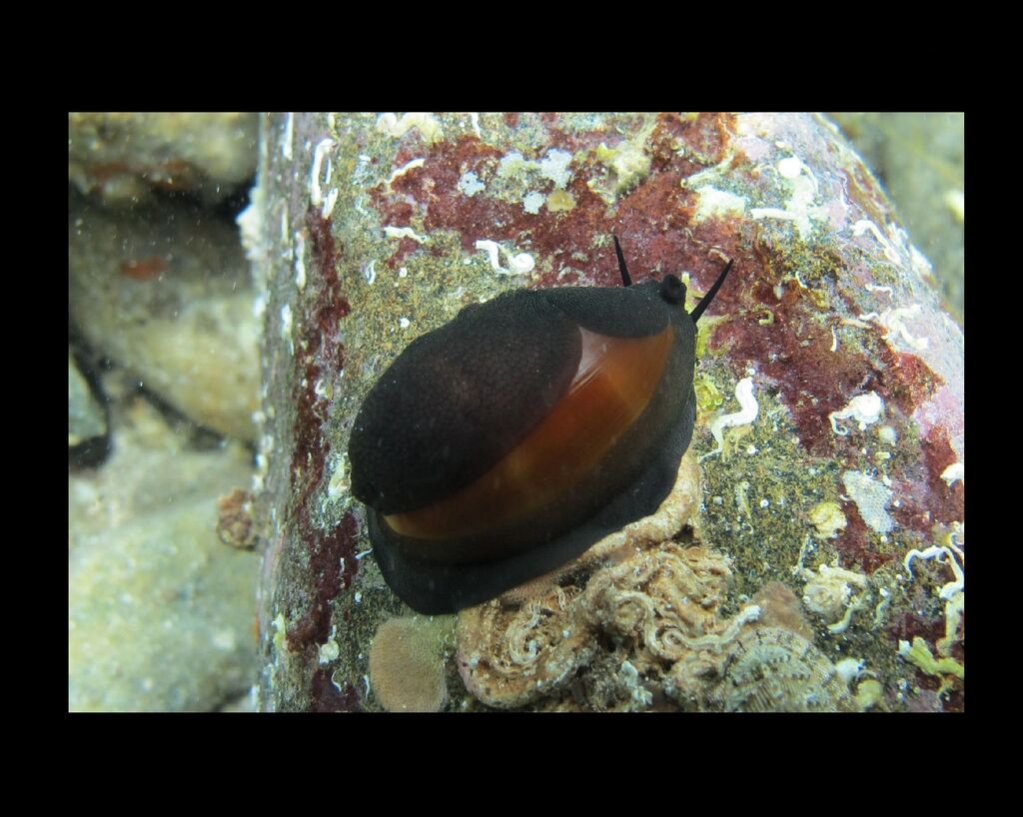
*Lurialurida* (Linnaeus, 1758). *In situ* photograph.

**Figure 30.**
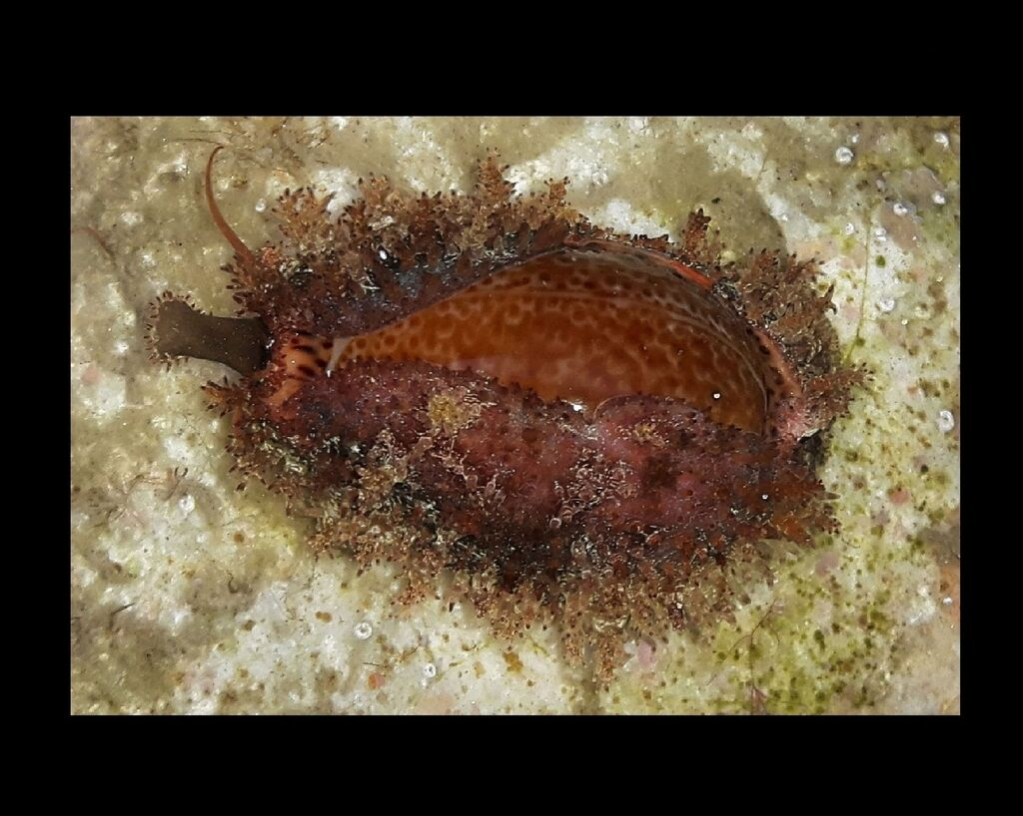
*Nariaspurca* (Linnaeus, 1758). *In situ* photograph.

**Figure 31.**
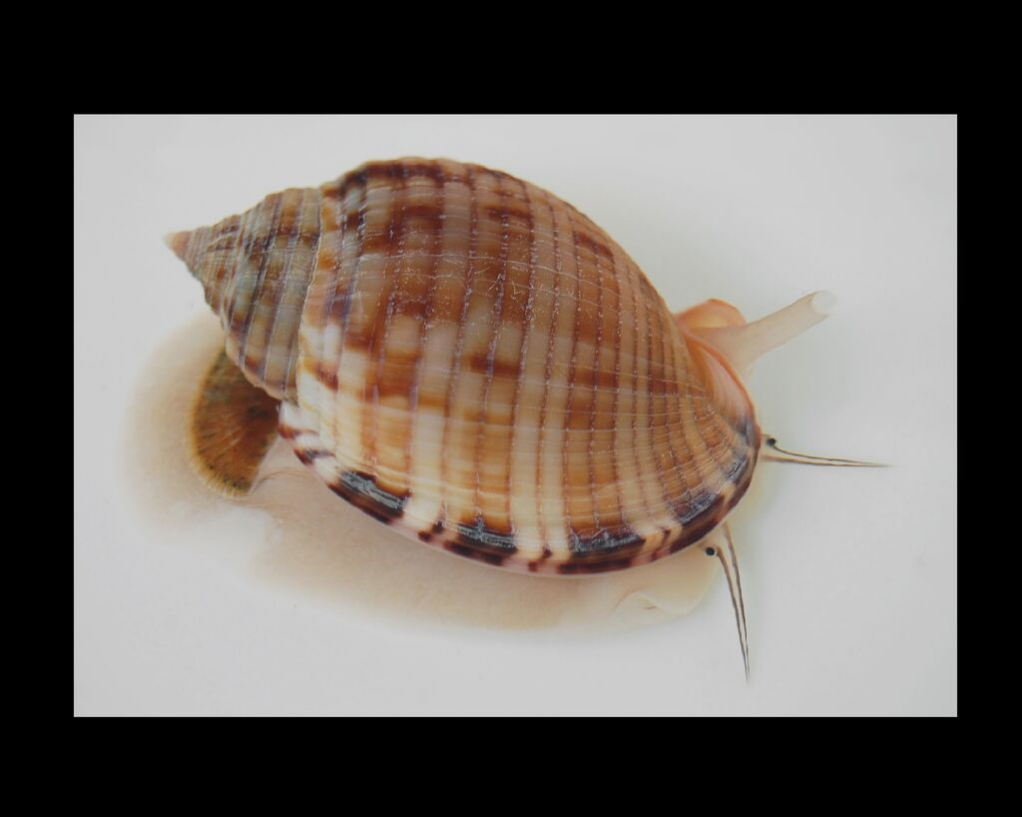
*Phaliumgranulatum* (Born, 1778). Optical photograph.

**Figure 32.**
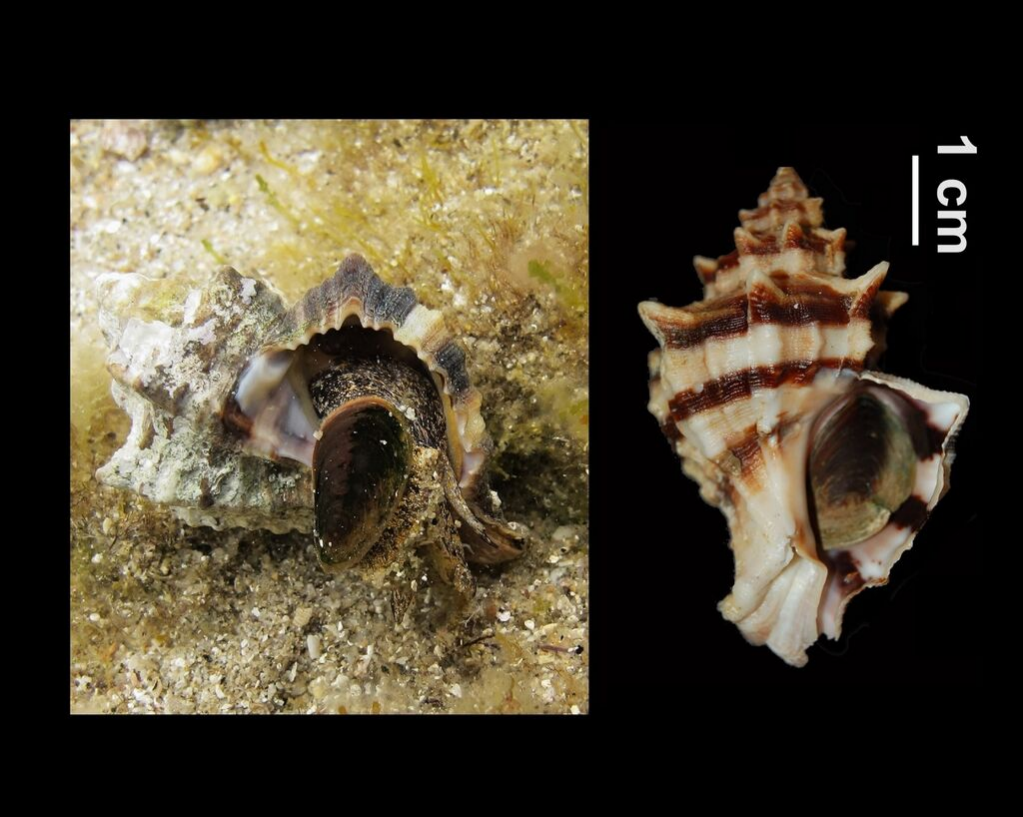
*Hexaplextrunculus* (Linnaeus, 1758). On the left, *in situ* photograph specimen A; on the right optical photograph specimen B.

**Figure 33.**
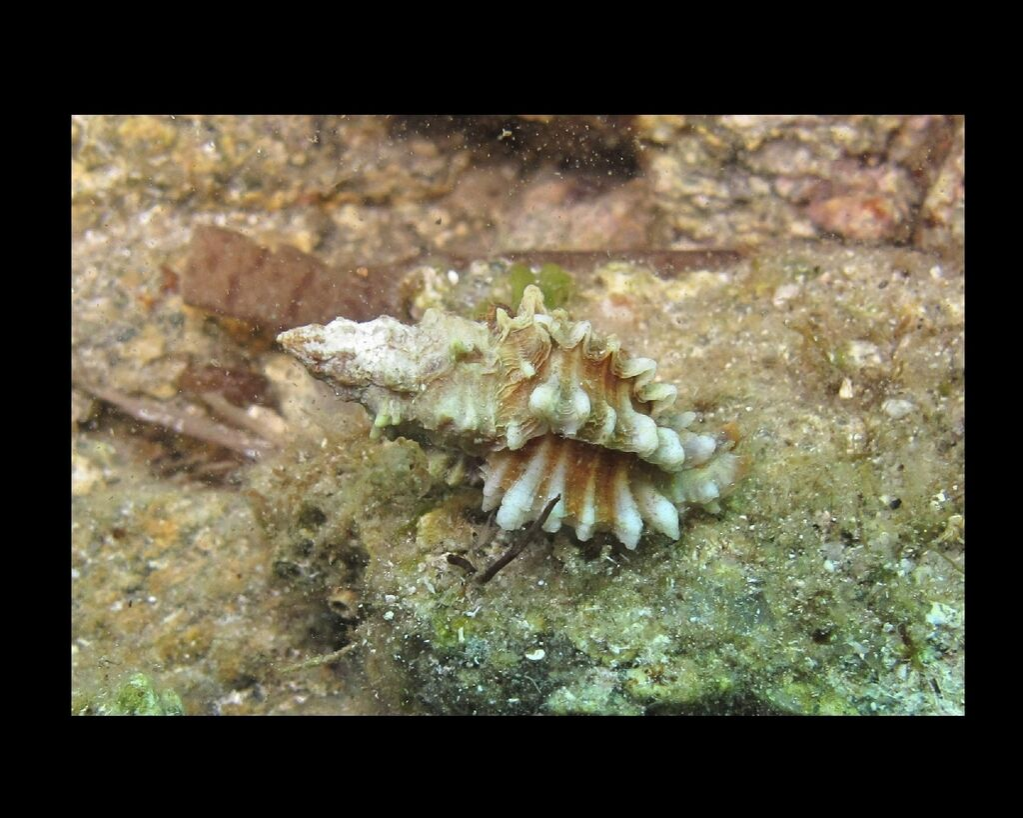
*Muricopsiscristata* (Brocchi, 1814). *In situ* photograph.

**Figure 34.**
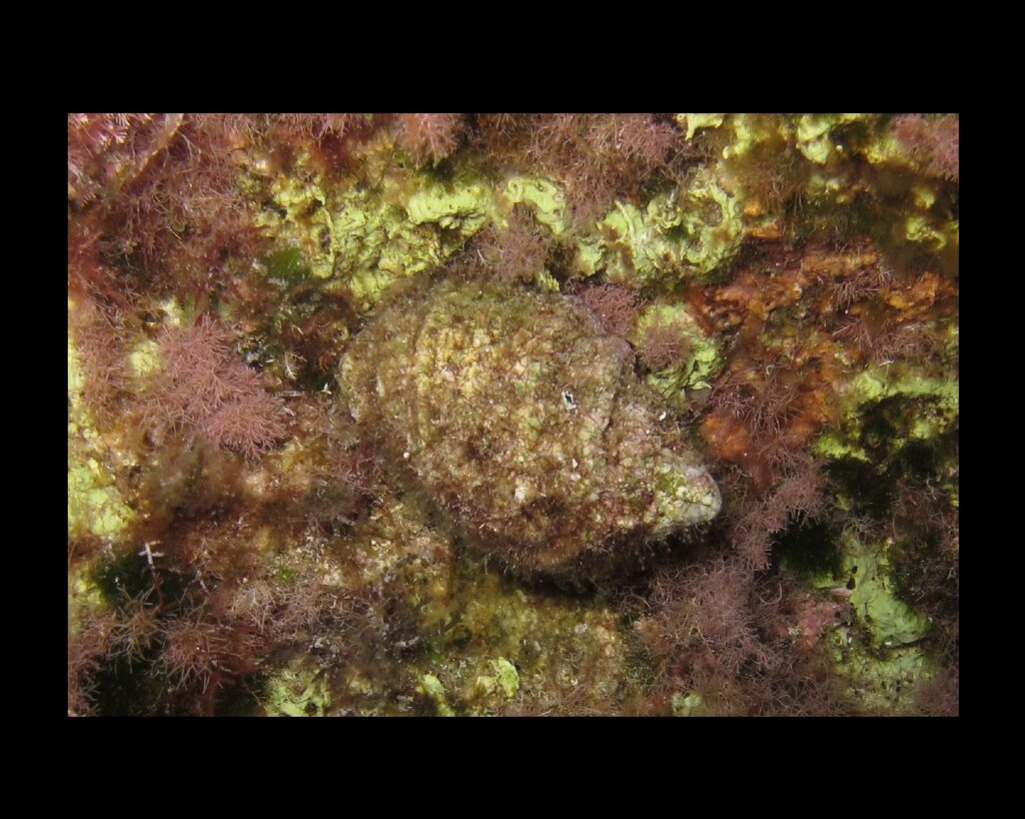
*Stramonitahaemastoma* (Linnaeus, 1767). *In situ* photograph.

**Figure 35.**
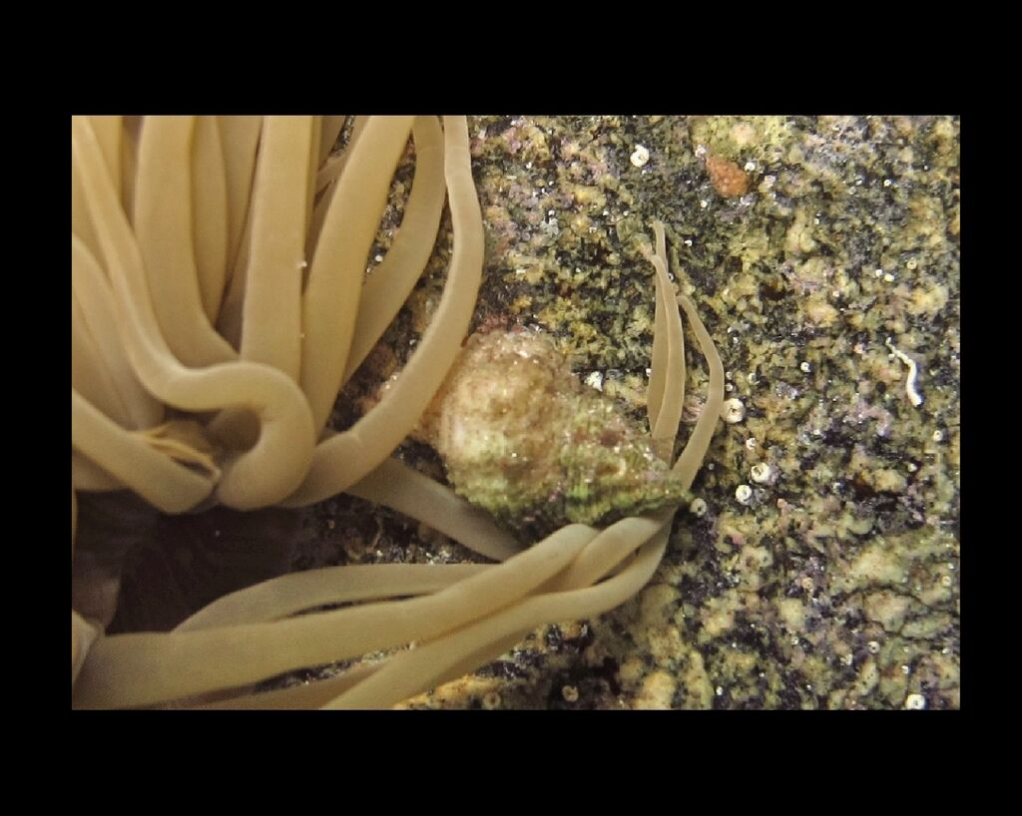
*Coralliophilameyendorffii* (Calcara, 1845). *In situ* photograph.

**Figure 36.**
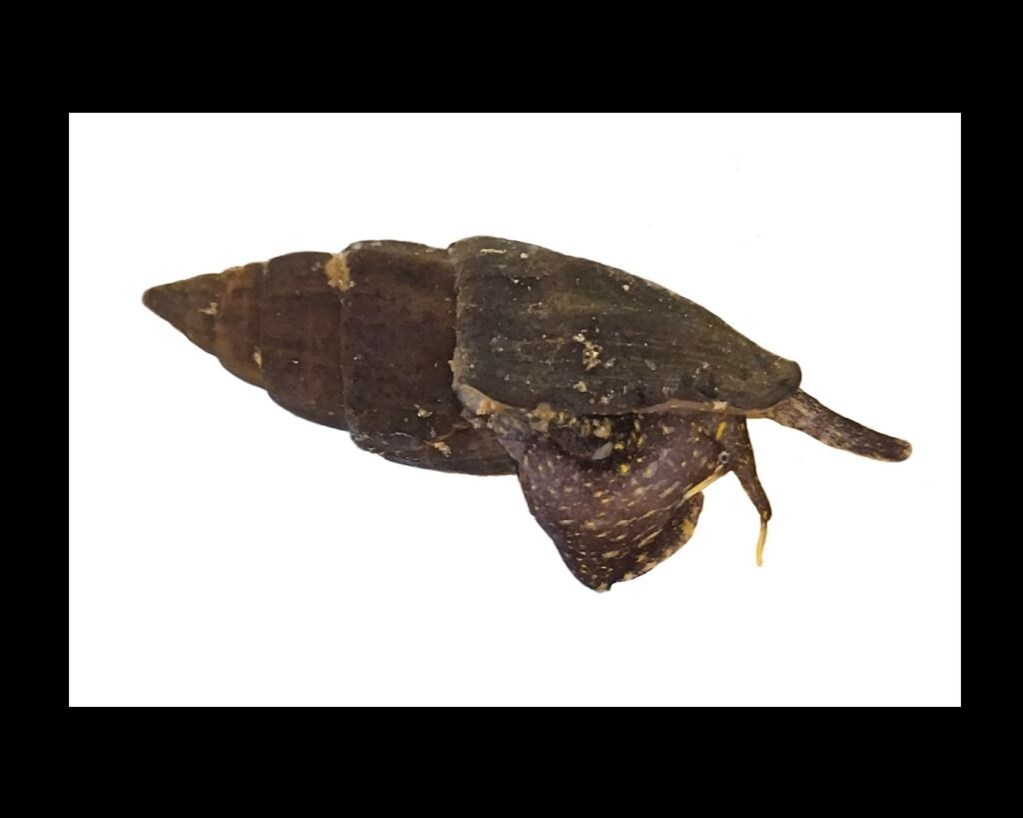
*Pusiaebenus* (Lamark, 1811). Optical photograph.

**Figure 37.**
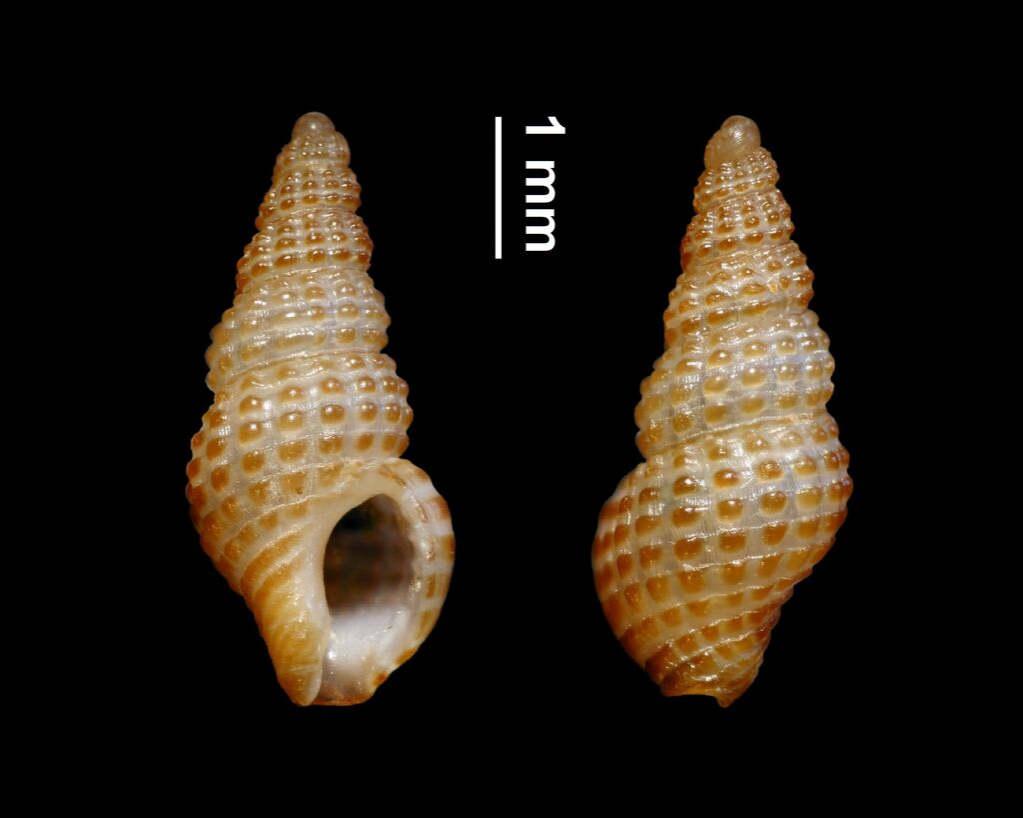
*Chauvetiaturritellata* (Deshayes, 1835). Optical photograph.

**Figure 38.**
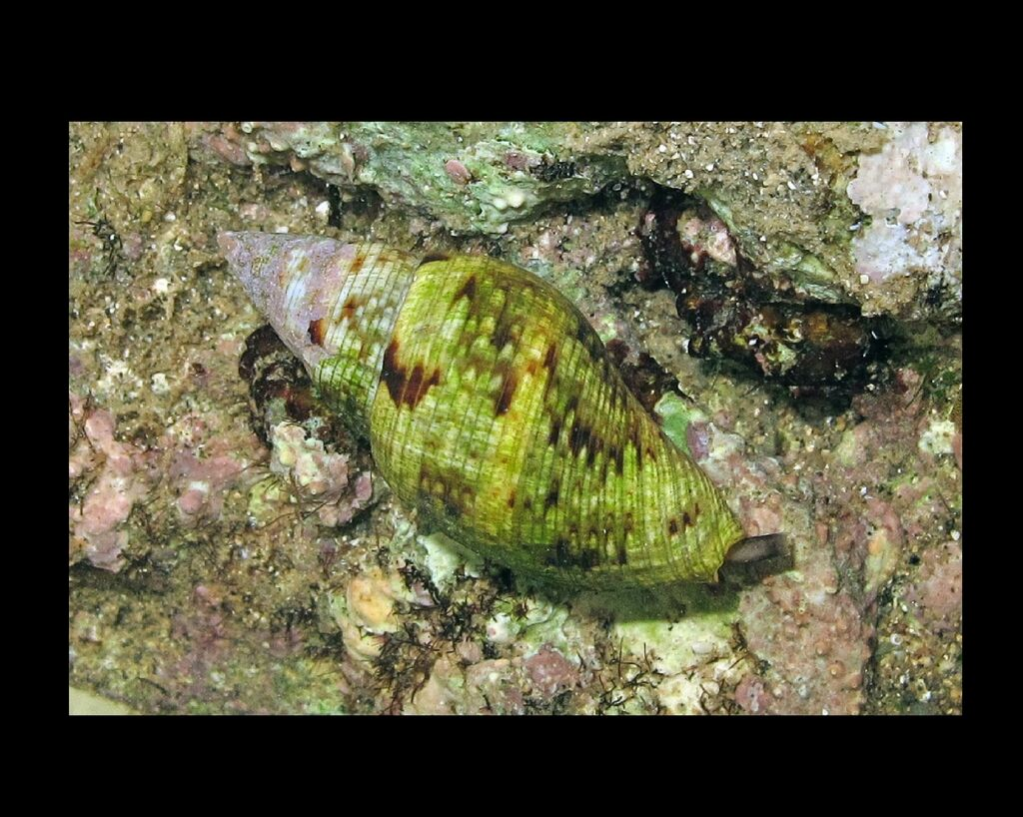
*Pisaniastriata* (Gmelin, 1791). *In situ* photograph.

**Figure 39.**
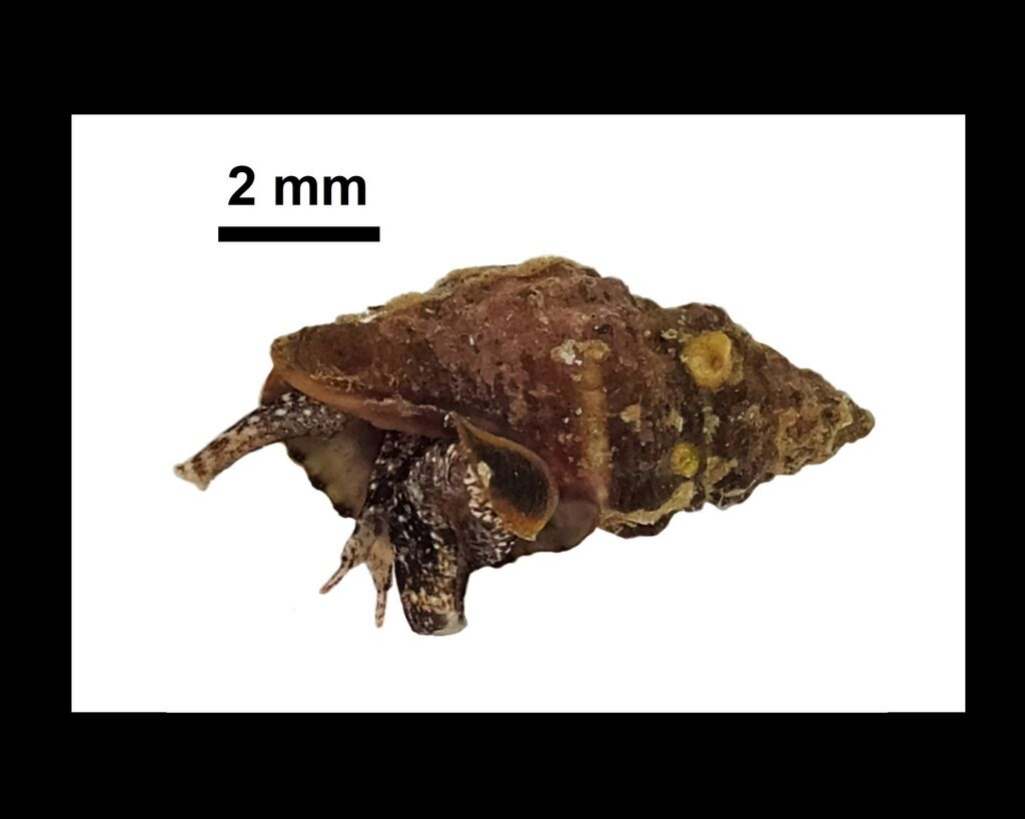
*Aplusdorbigny* (Payraudeau, 1826). Optical photograph.

**Figure 40.**
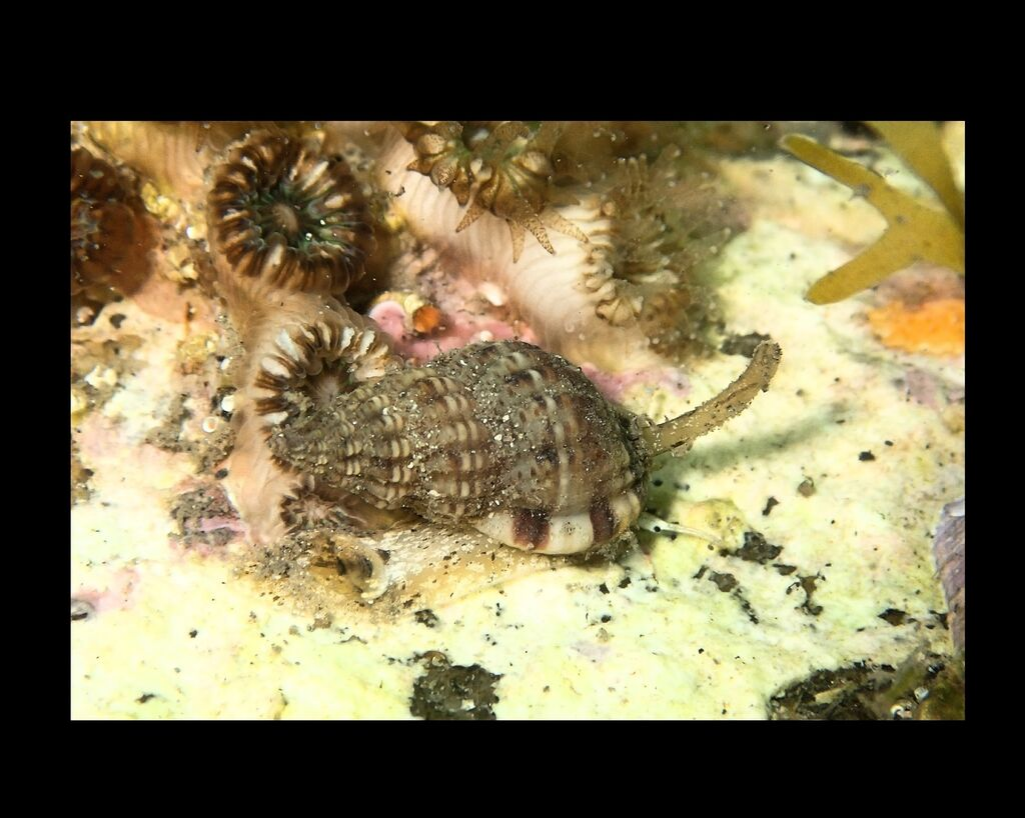
*Tritiaincrassata* (Strøm, 1768). *In situ* photograph.

**Figure 41.**
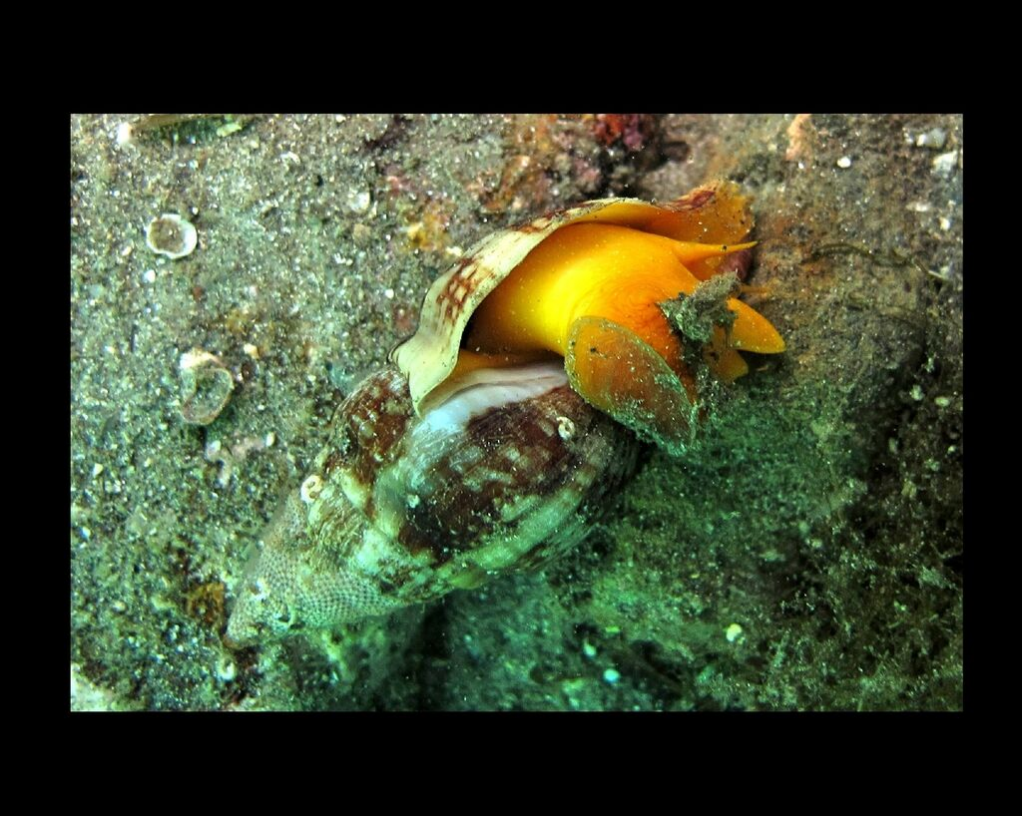
*Euthriacornea* (Linnaeus, 1758). *In situ* photograph.

**Figure 42.**
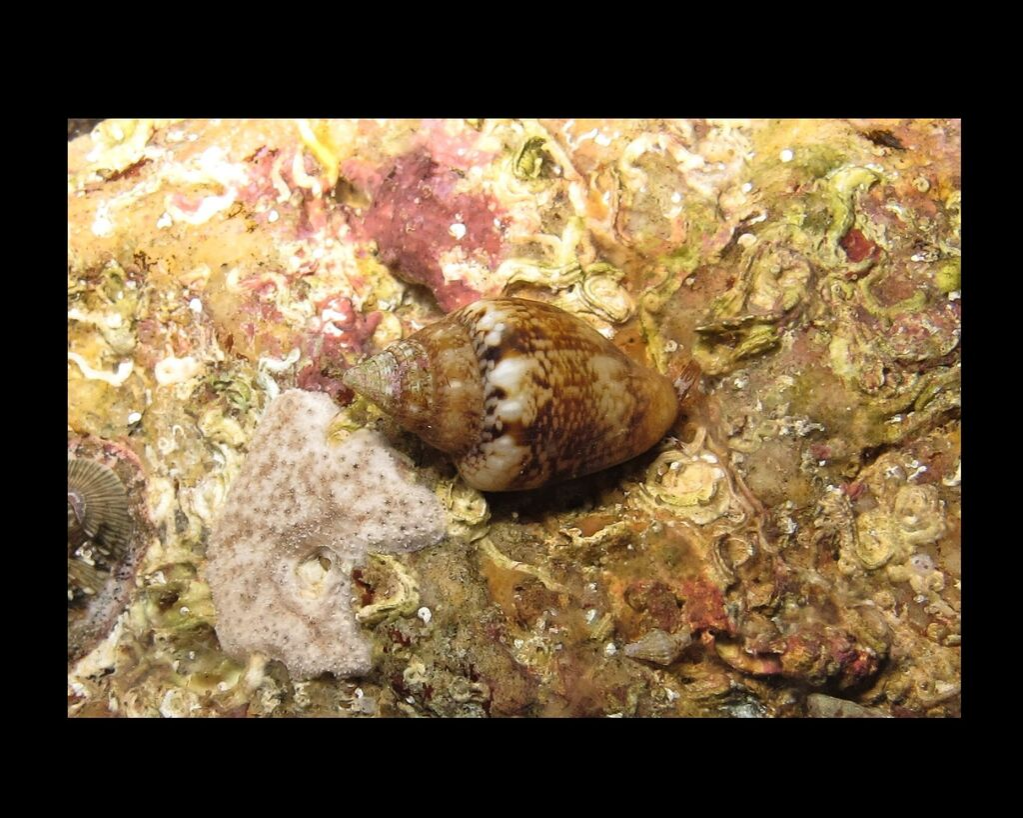
*Columbellarustica* (Linnaeus, 1758). *In situ* photograph.

**Figure 43.**
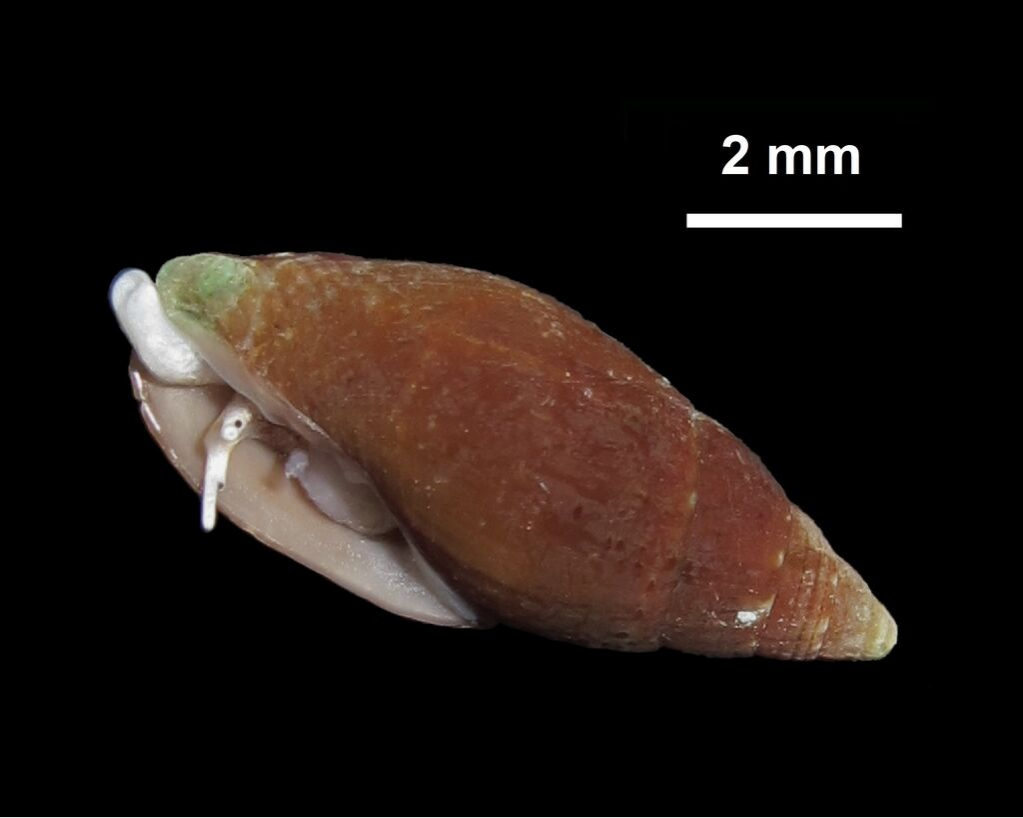
*Episcomitracornicula* (Linnaeus, 1758). Optical photograph.

**Figure 44.**
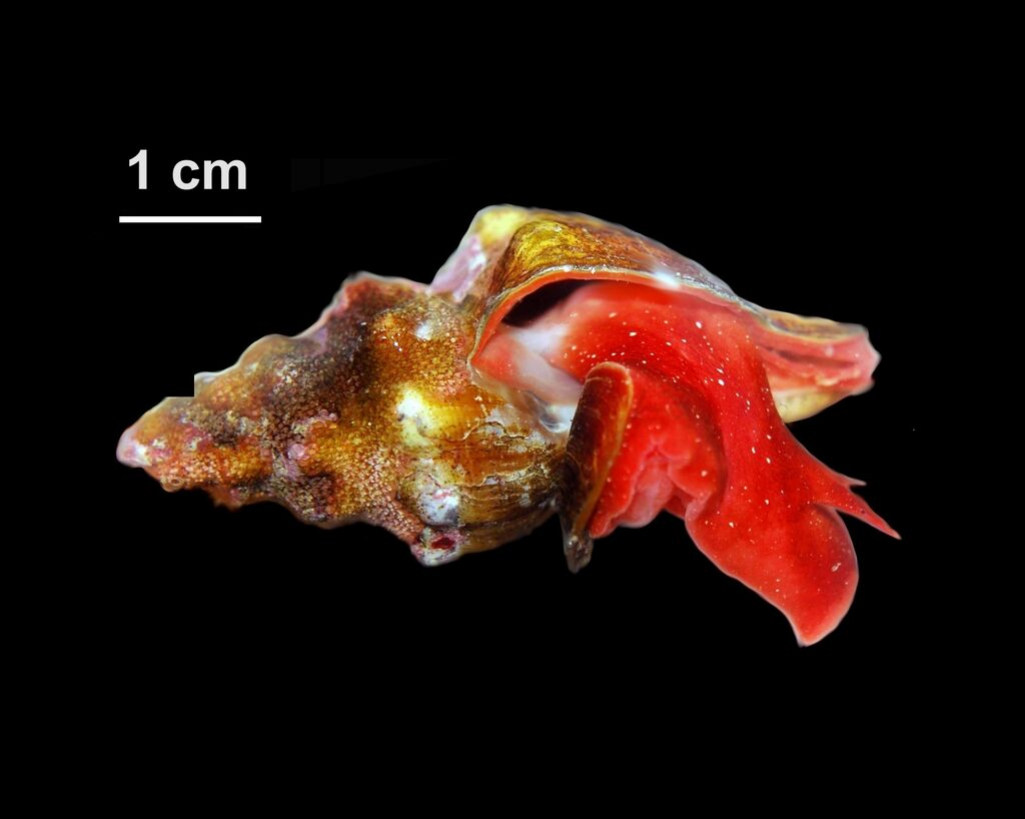
*Tarantinaealignaria* (Linnaeaus, 1758). Optical photograph.

**Figure 45.**
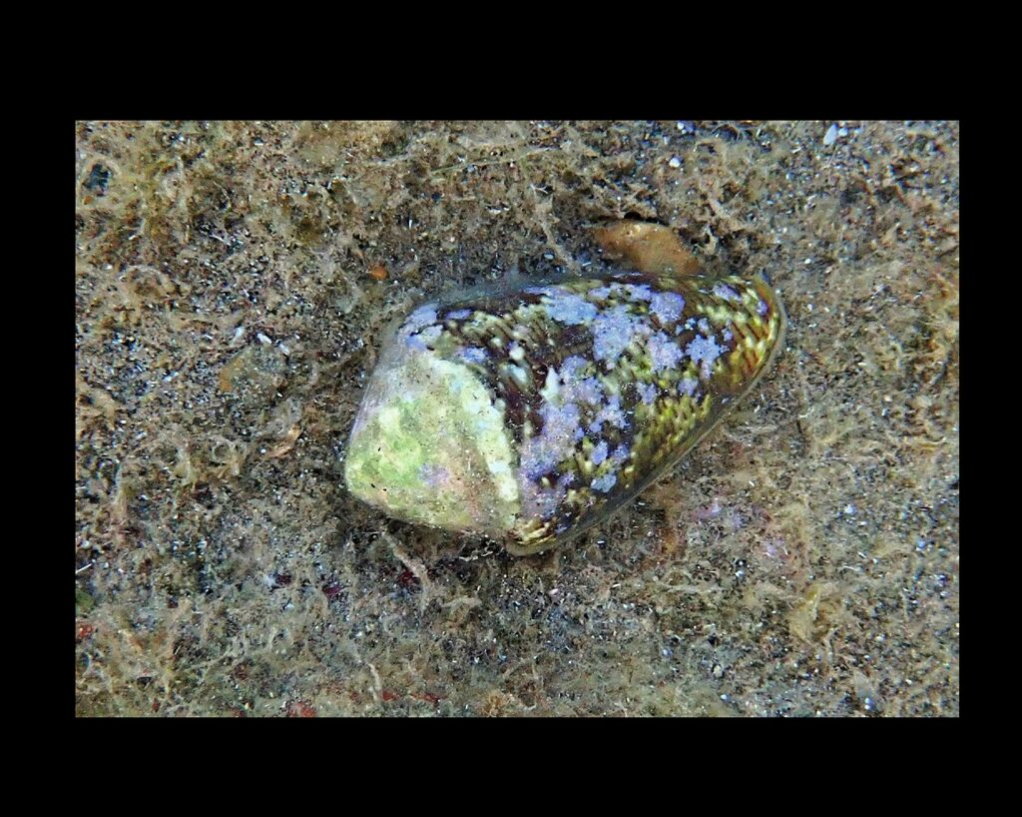
*Conusventricosus* Gmelin, 1791. *In situ* photograph.

**Figure 48.**
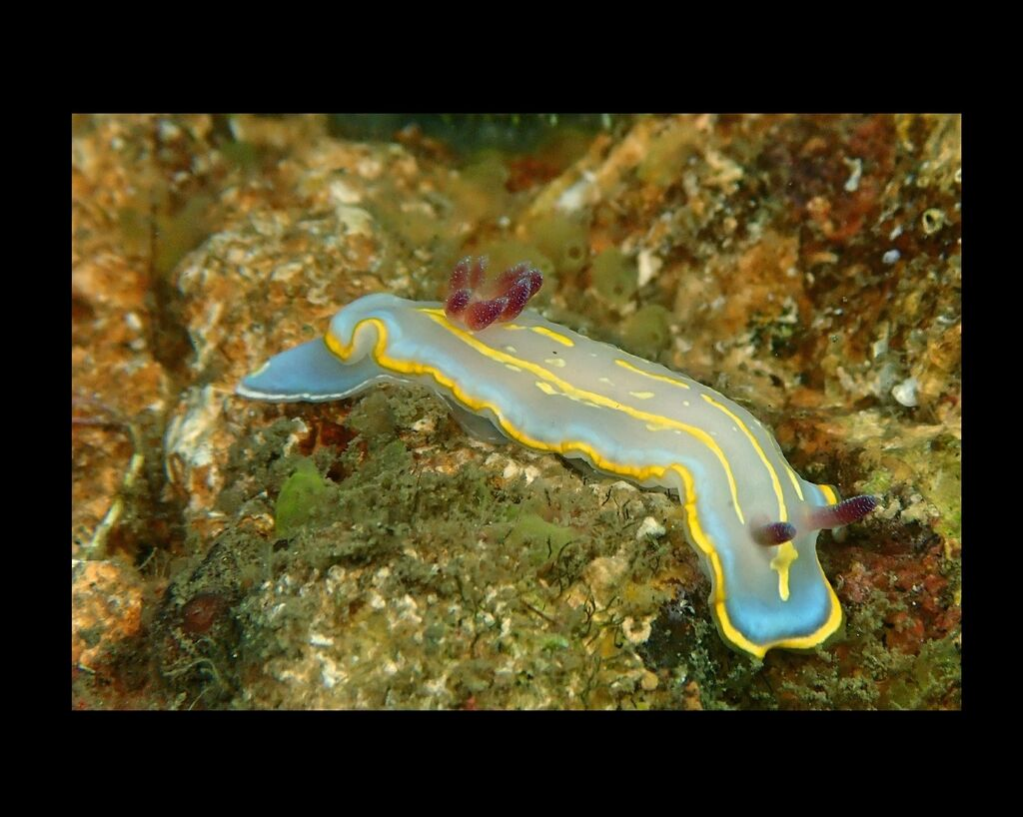
*Felimidakrohni* (Vérany, 1846). *In situ* photograph.

**Figure 49.**
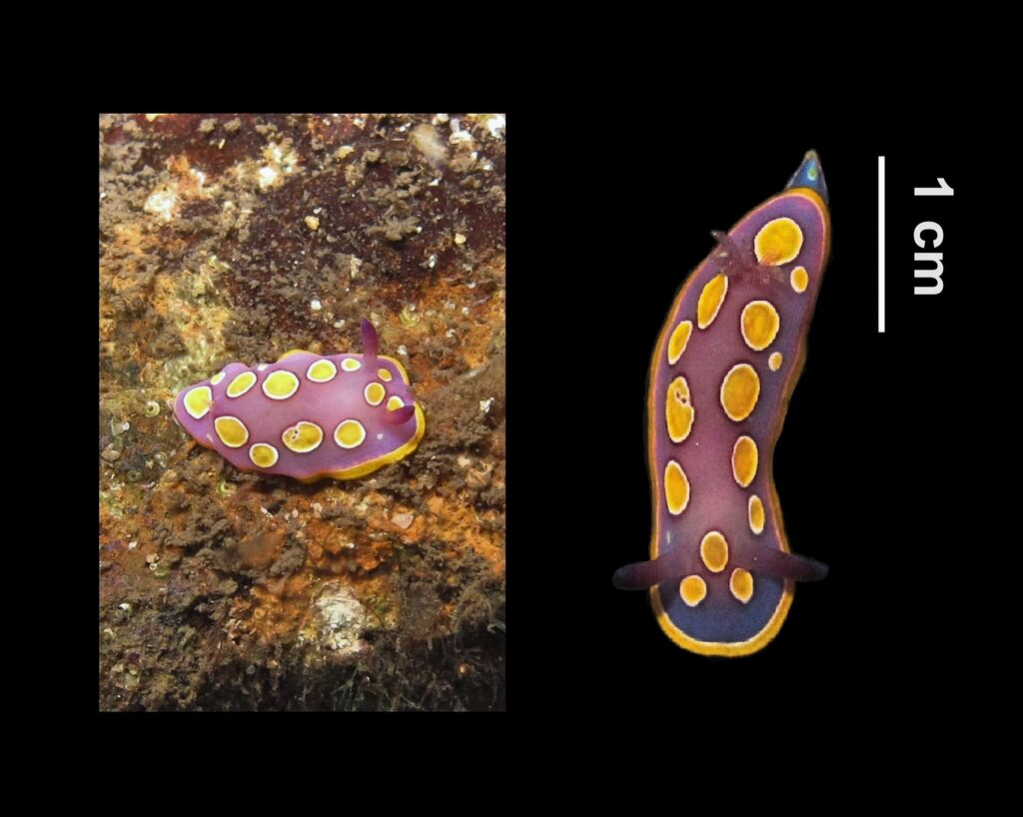
*Felimidaluteorosea* (Rapp, 1827). On the left, *in situ* photograph of specimen A; on the right, optical photograph of specimen B.

**Figure 50.**
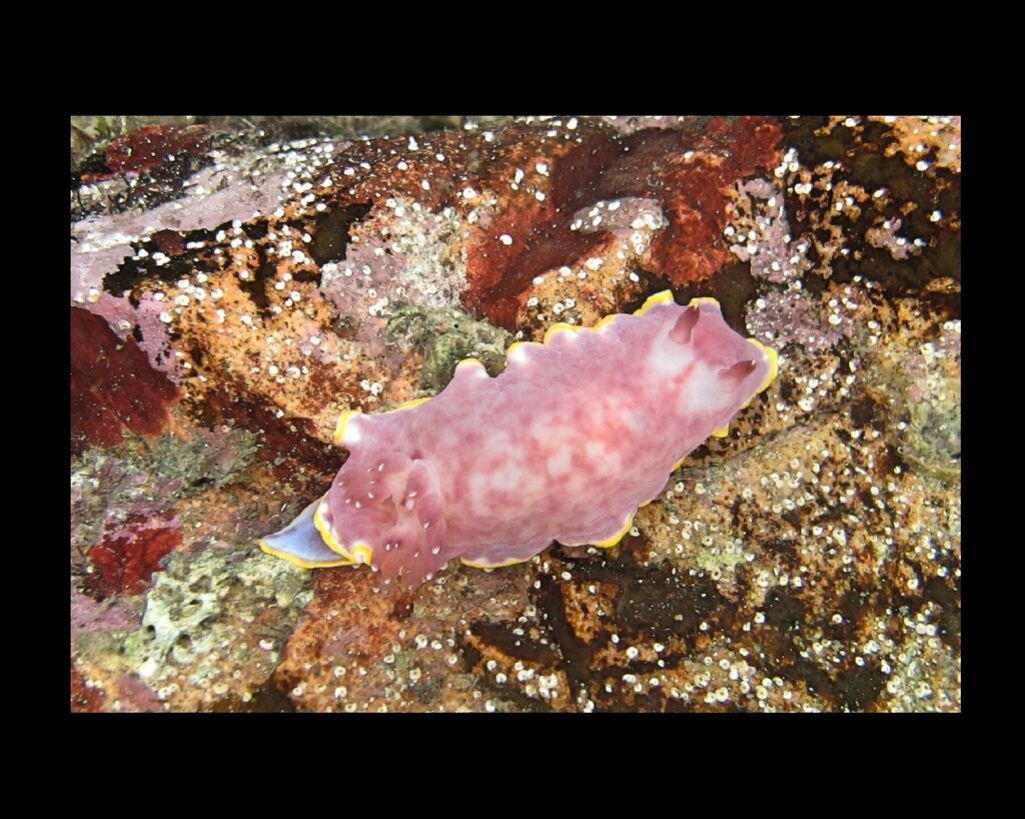
*Felimidapurpurea* (Risso, 1831). *In situ* photograph.

**Figure 51.**
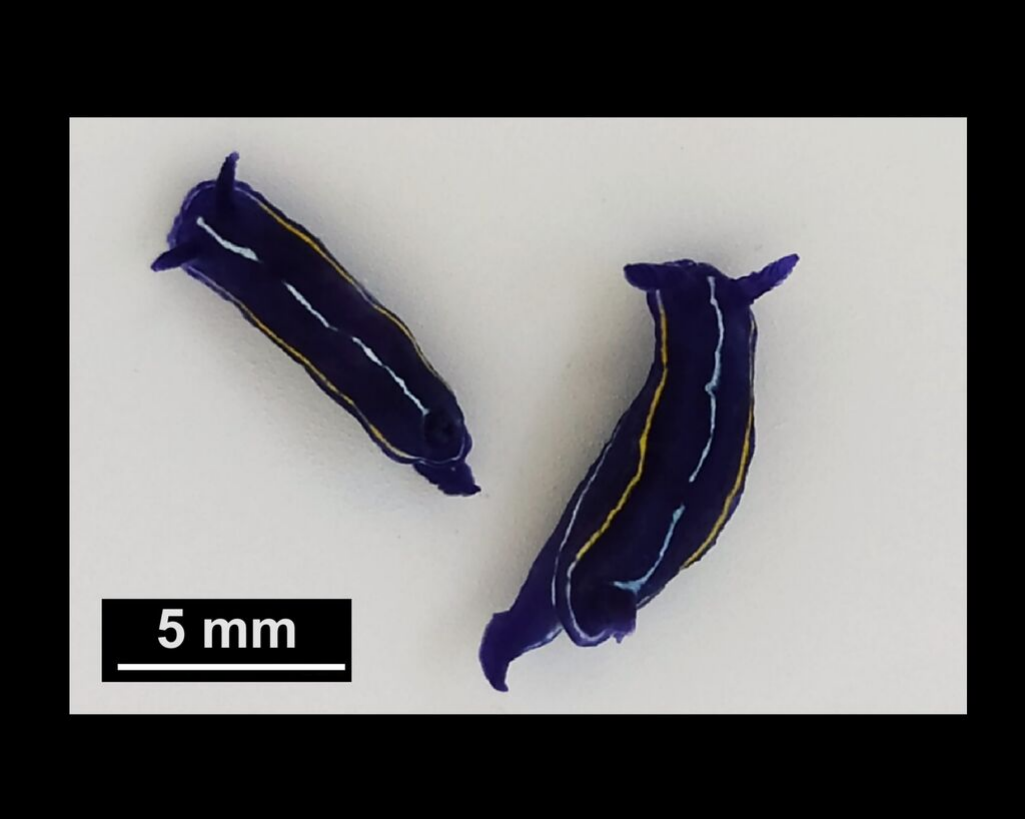
*Felimareorsinii* (Vérany, 1846). *In situ* photograph.

**Figure 53.**
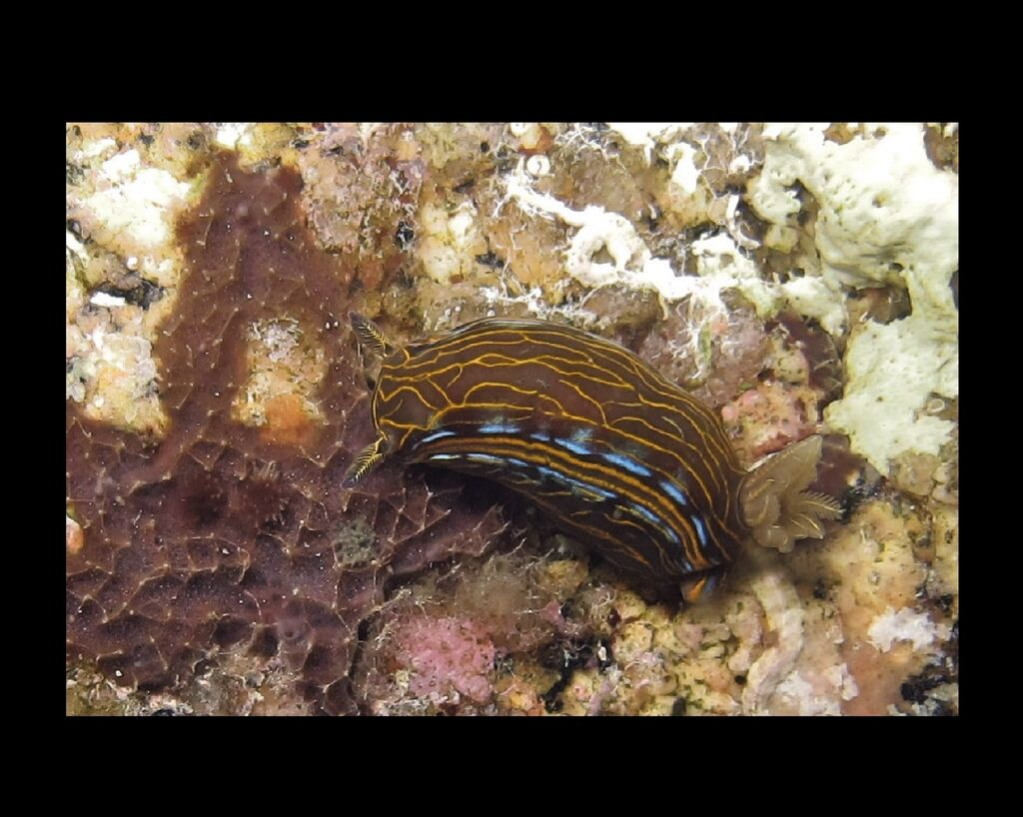
*Felimarevillafranca* (Risso, 1818). *In situ* photograph.

**Figure 54.**
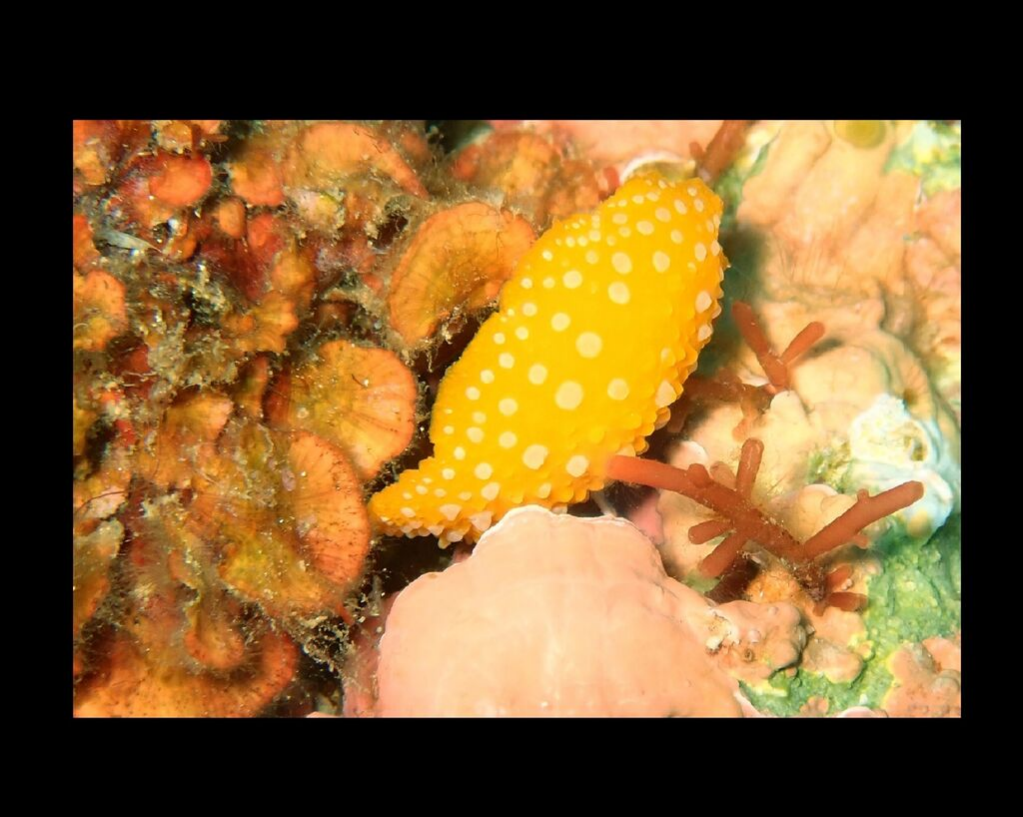
*Phyllidiaflava* Aradas, 1847. *In situ* photograph.

**Figure 55.**
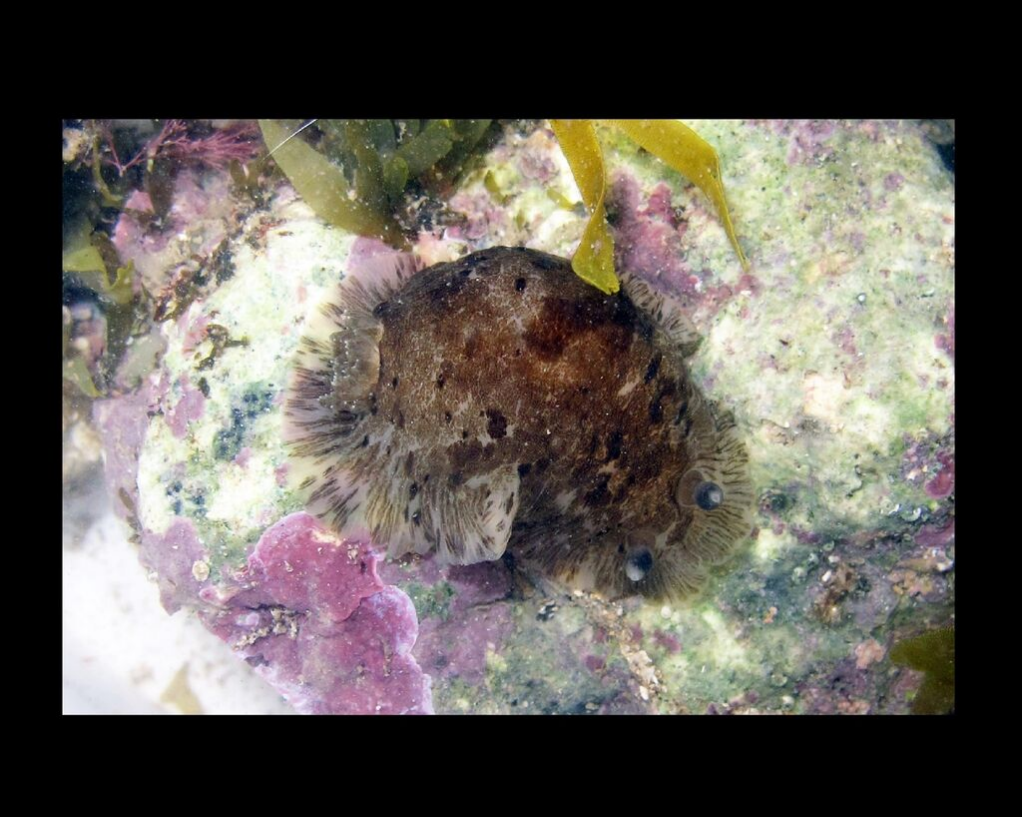
*Dendrodorisgrandiflora* (Rapp, 1827). *In situ* photograph.

**Figure 56.**
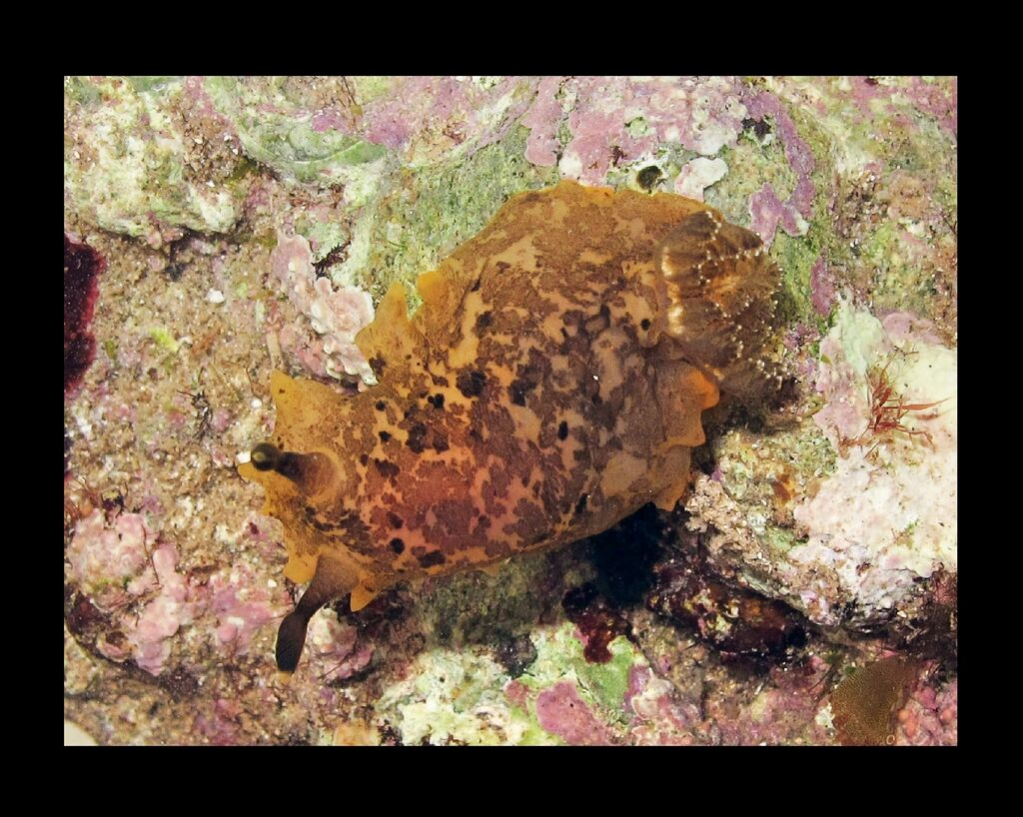
*Dendrodorislimbata* (Cuvier, 1804). *In situ* photograph.

**Figure 57.**
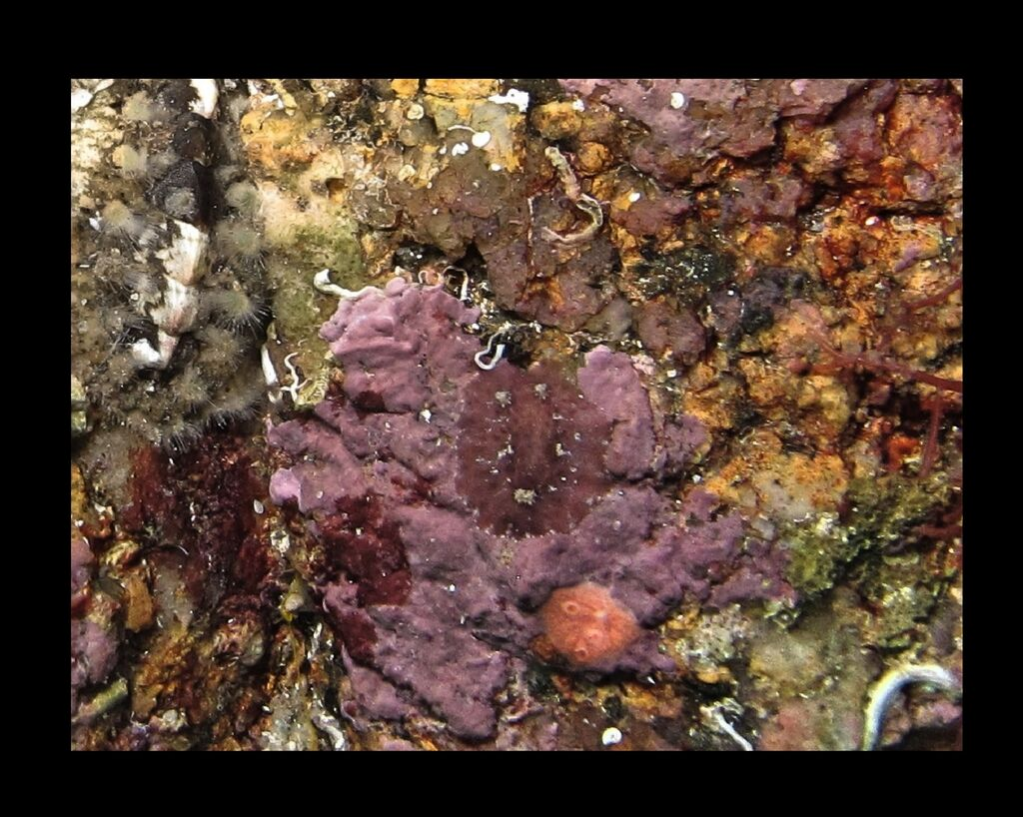
*Taringaarmata* Swennen, 1961. *In situ* photograph.

**Figure 58.**
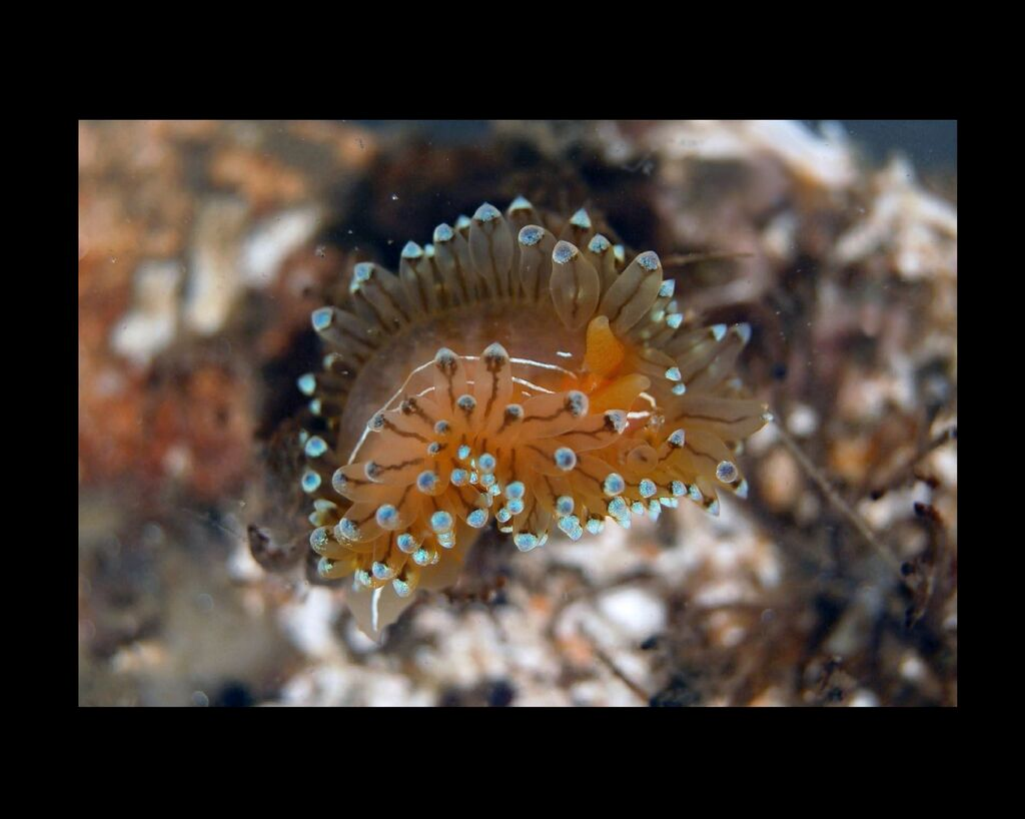
*Antiopellacristata* (Delle Chiaje, 1841). *In situ* photograph.

**Figure 59.**
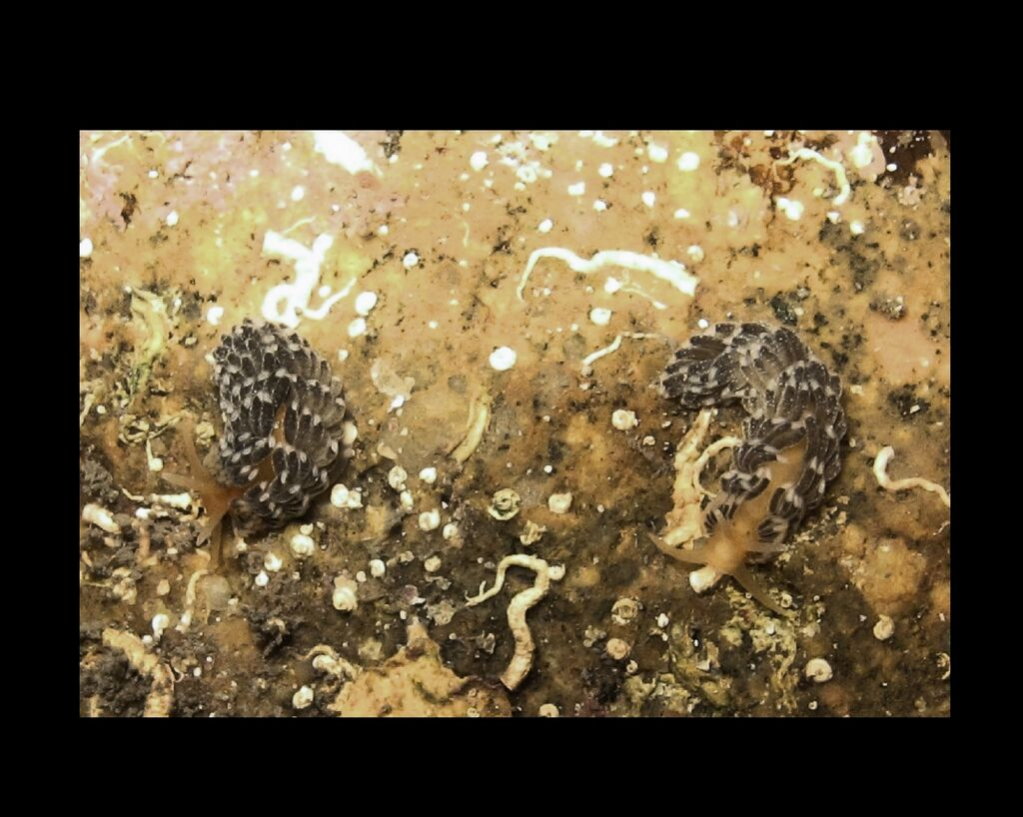
*Aeolidiellaalderi* (Cocks, 1852). *In situ* photograph.

**Figure 60.**
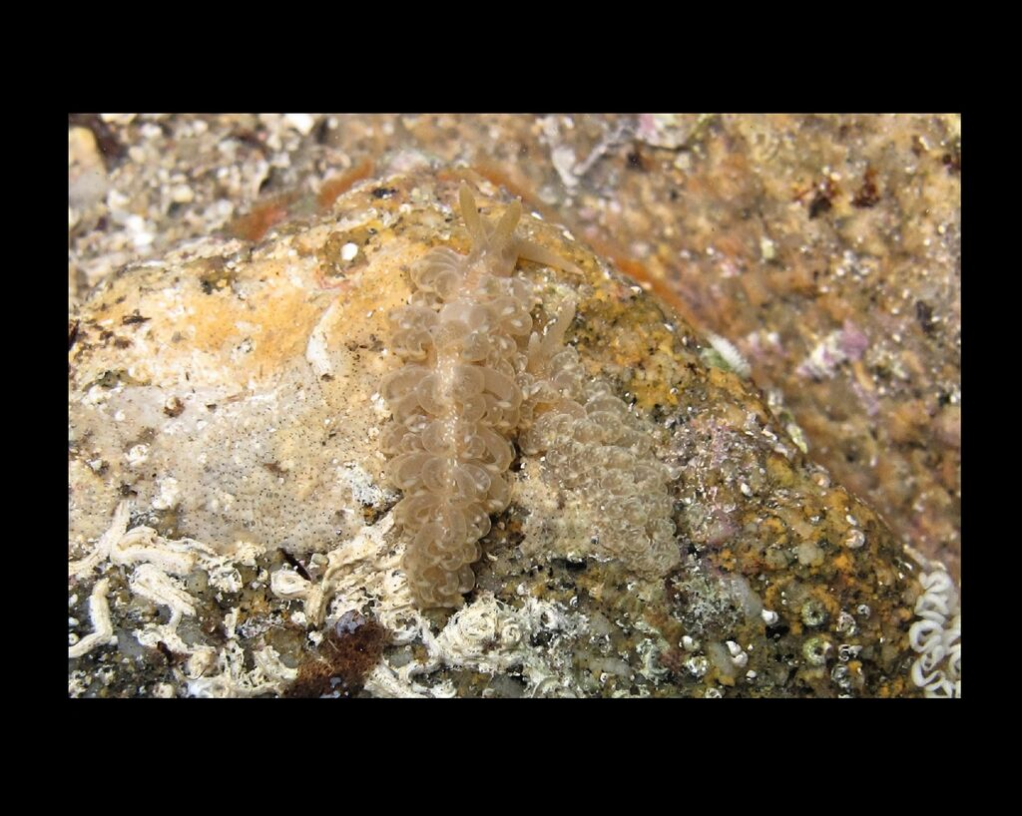
*Spurillaneapolitana* (Delle Chiaje, 1841). *In situ* photograph.

**Figure 62.**
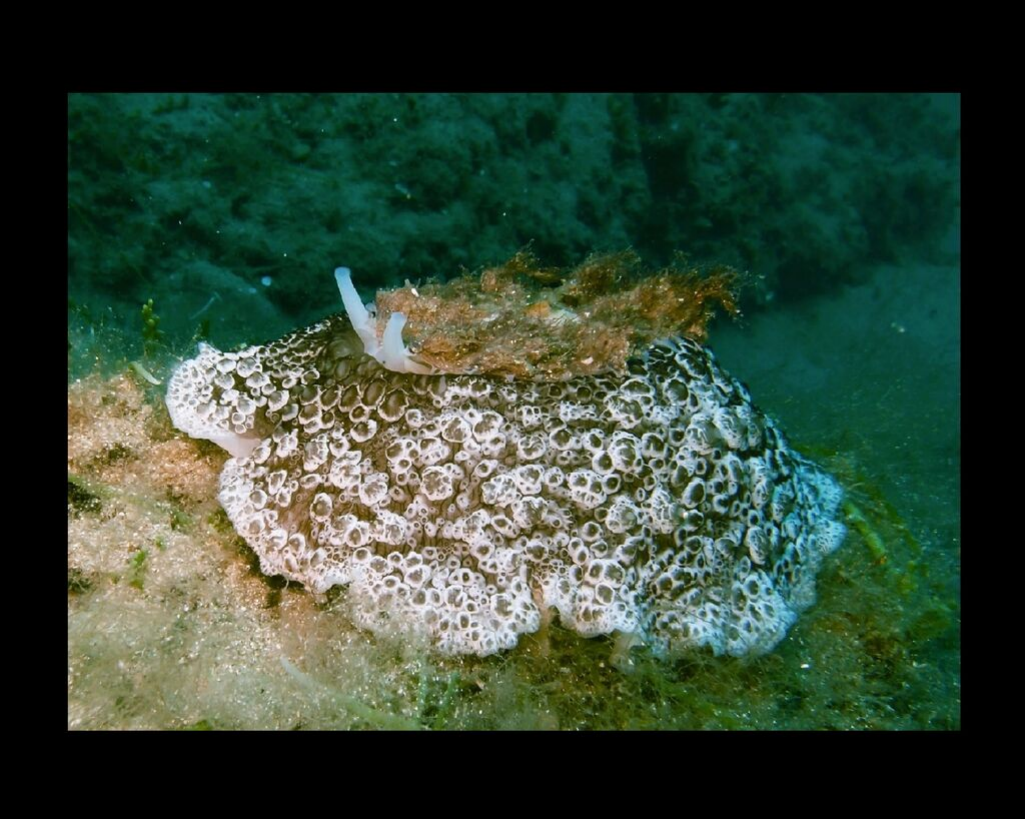
*Umbraculumumbraculum* ([Lighfoot], 1786). *In situ* photograph.

**Figure 63.**
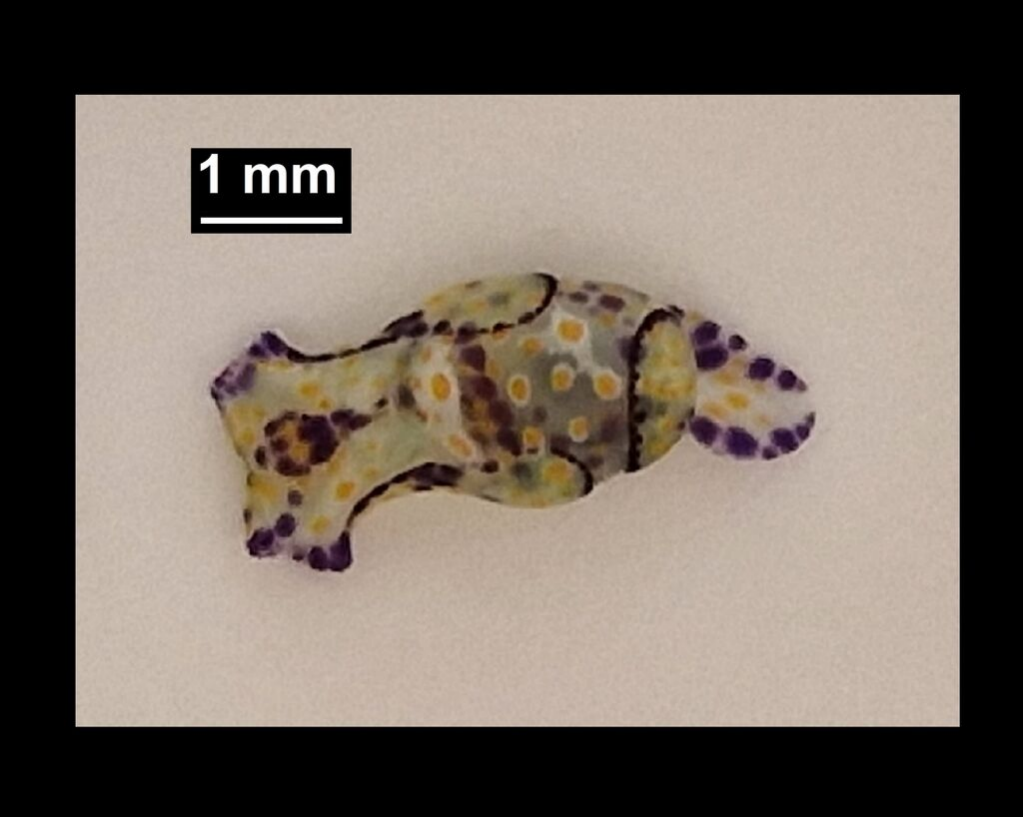
*Lamprohaminoeacyanomarginata* (Heller & T. E. Thompson, 1983). Optical photograph.

**Figure 64a.**
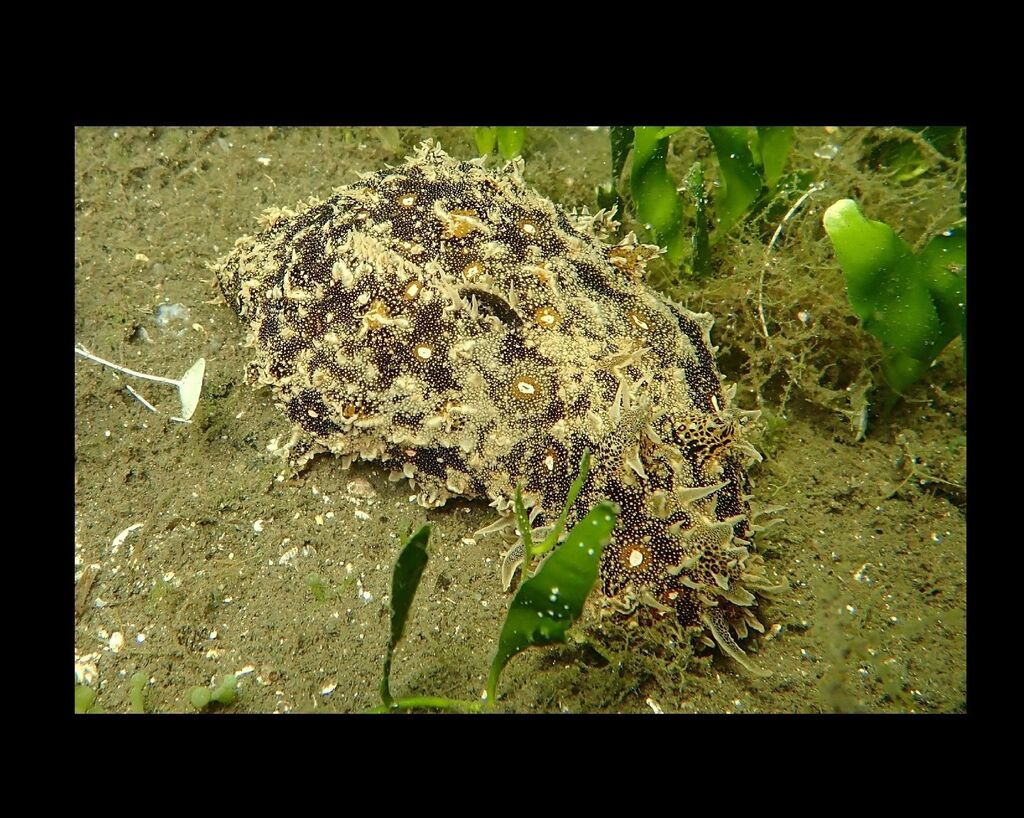
*In situ* photograph;

**Figure 64b.**
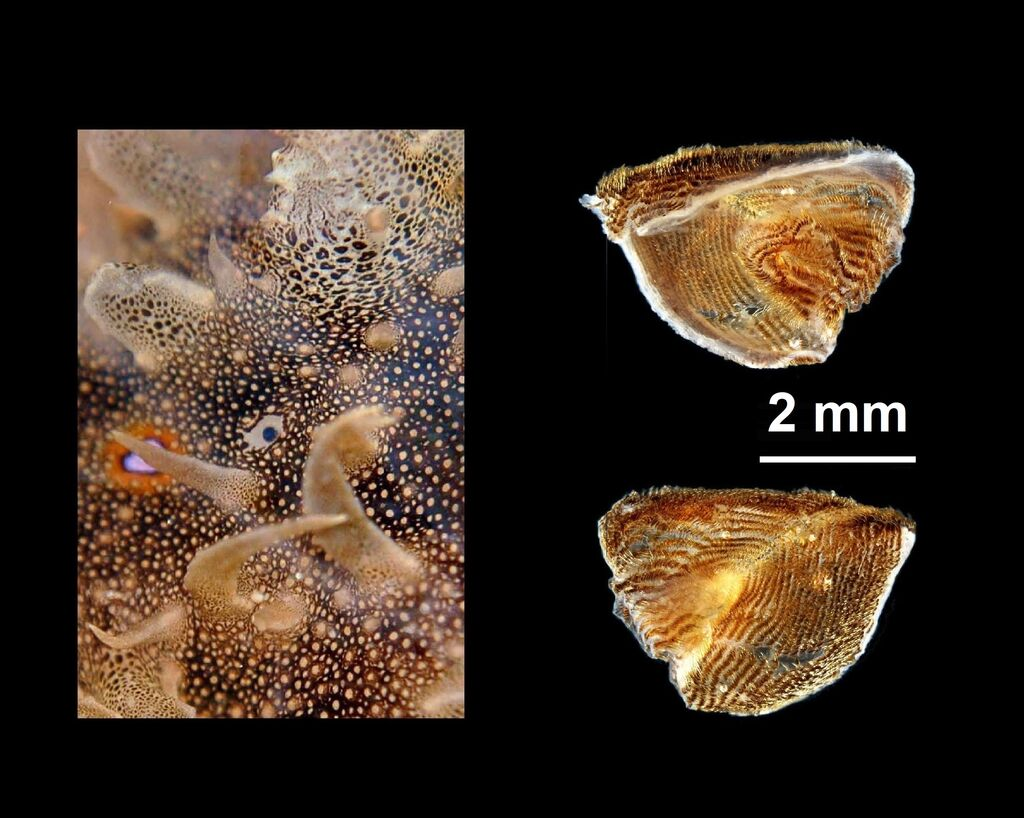
Details of the notum and radula.

**Figure 65a.**
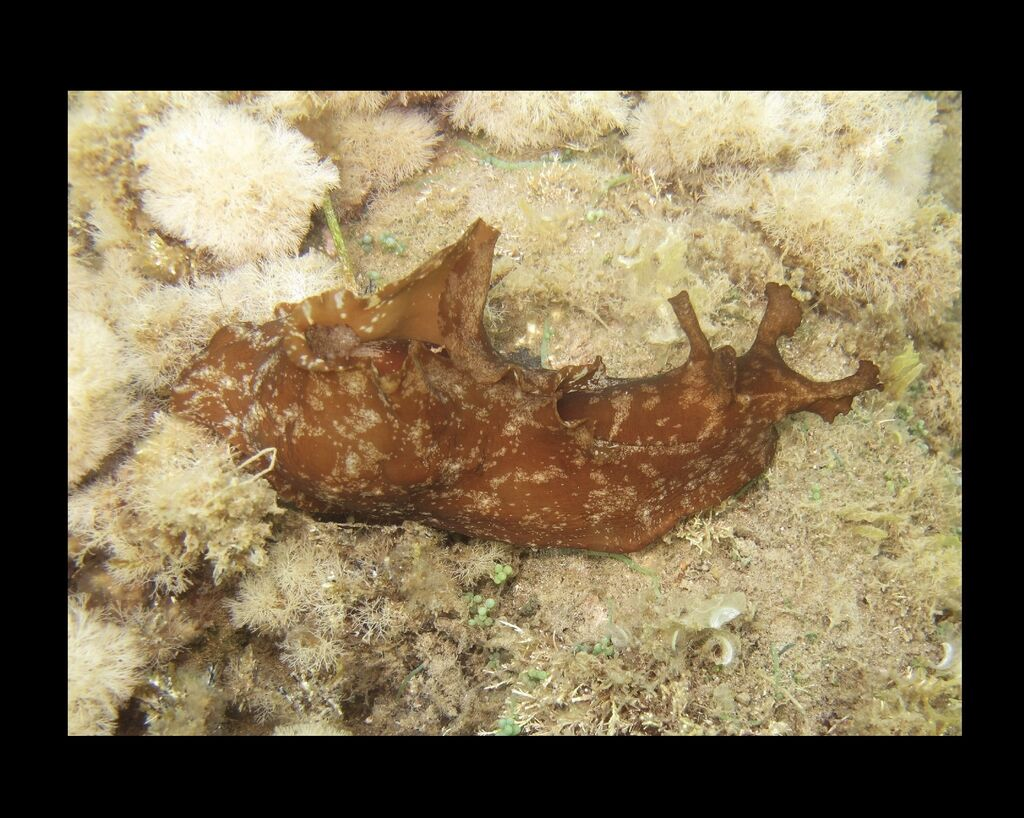
*In situ* photograph;

**Figure 65b.**
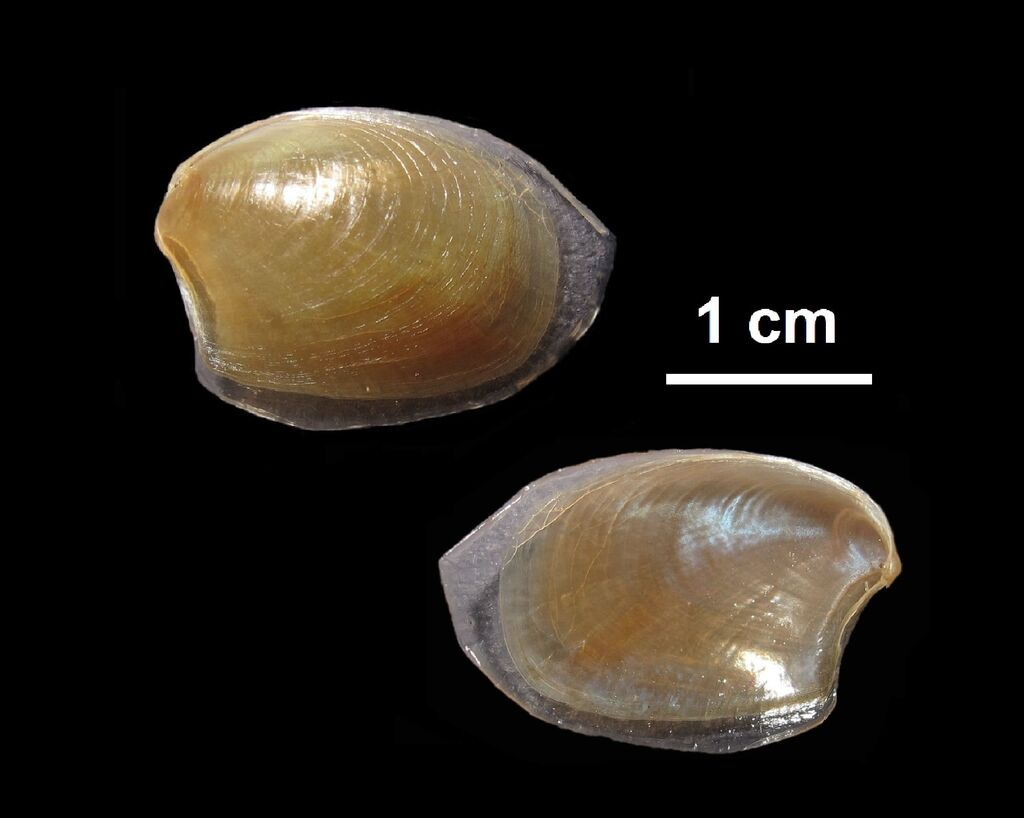
Internal shell.

**Figure 67.**
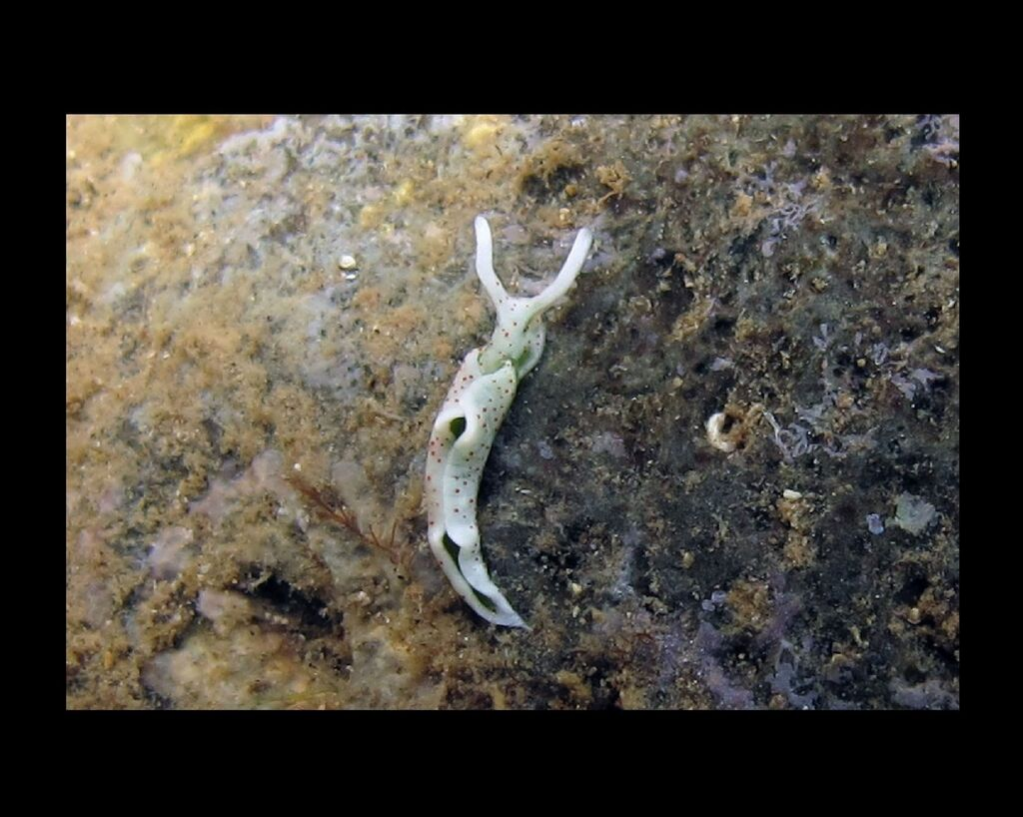
*Elysiatimida* (Risso, 1818). *In situ* photograph.

**Figure 68.**
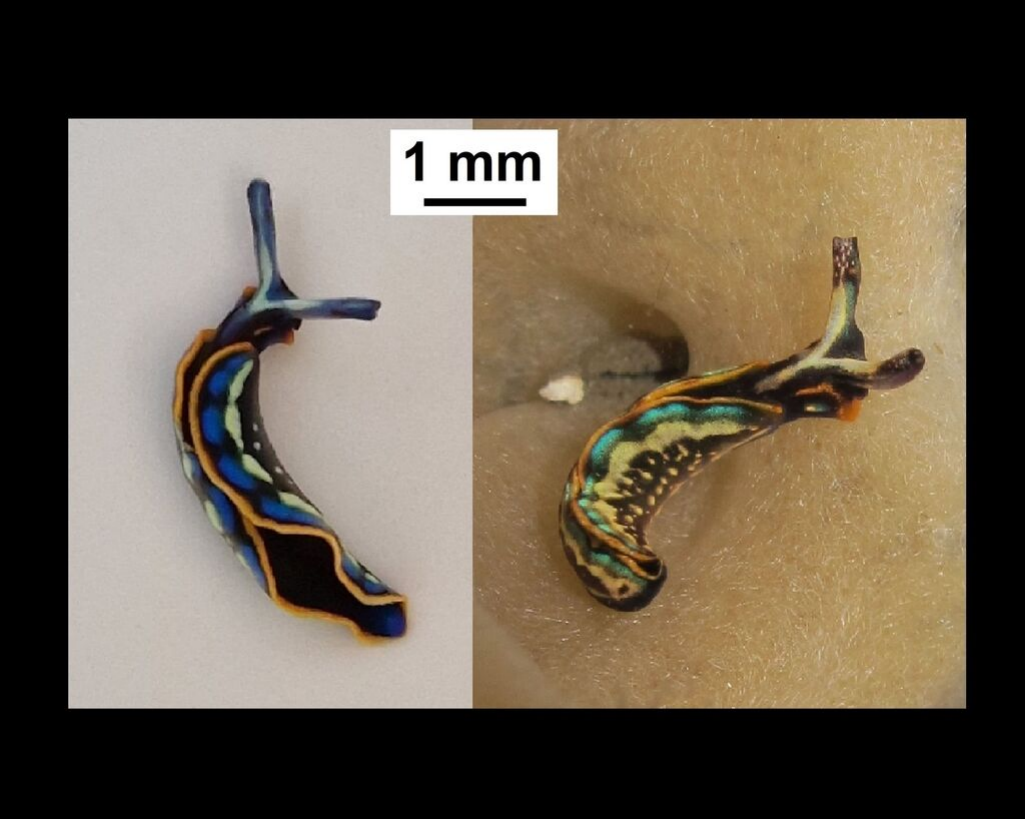
*Thuridillahopei* (Vérany, 1853). Different colour morphotypes. On the left, optical photograph; on the right, *in situ* photograph.

**Figure 69.**
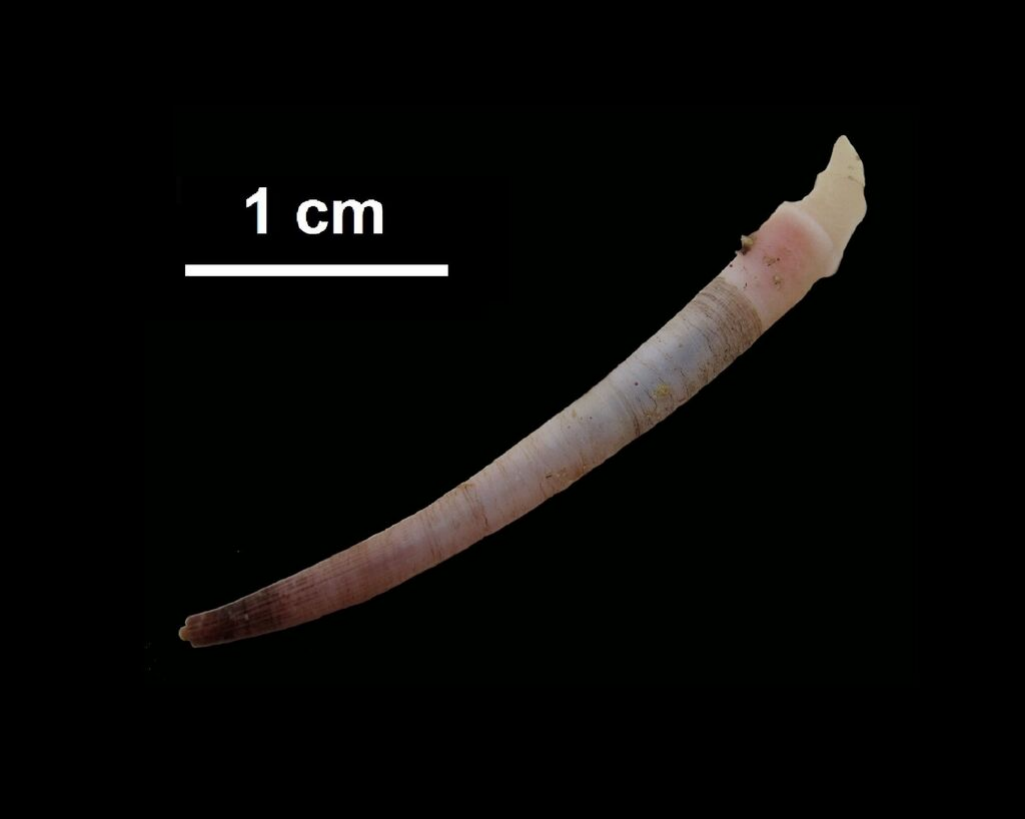
*Antalisvulgaris* (da Costa, 1778). Optical photograph.

**Figure 70.**
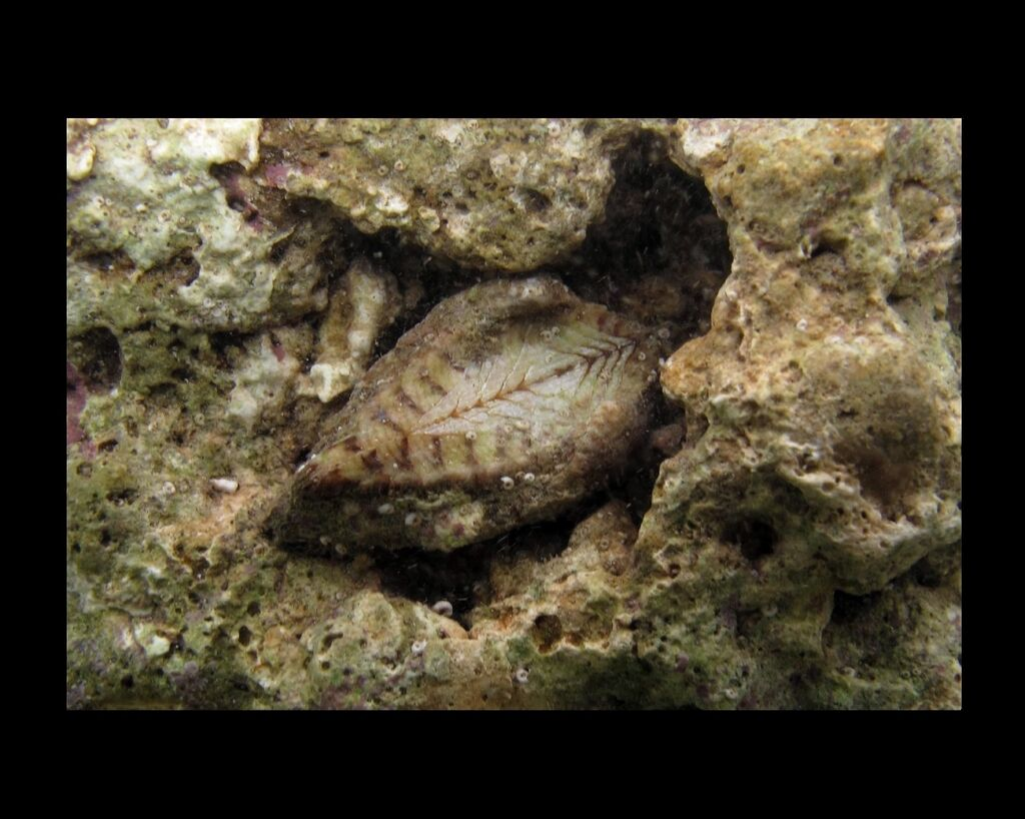
*Arcanoae* Linnaeus, 1758. *In situ* photograph.

**Figure 71.**
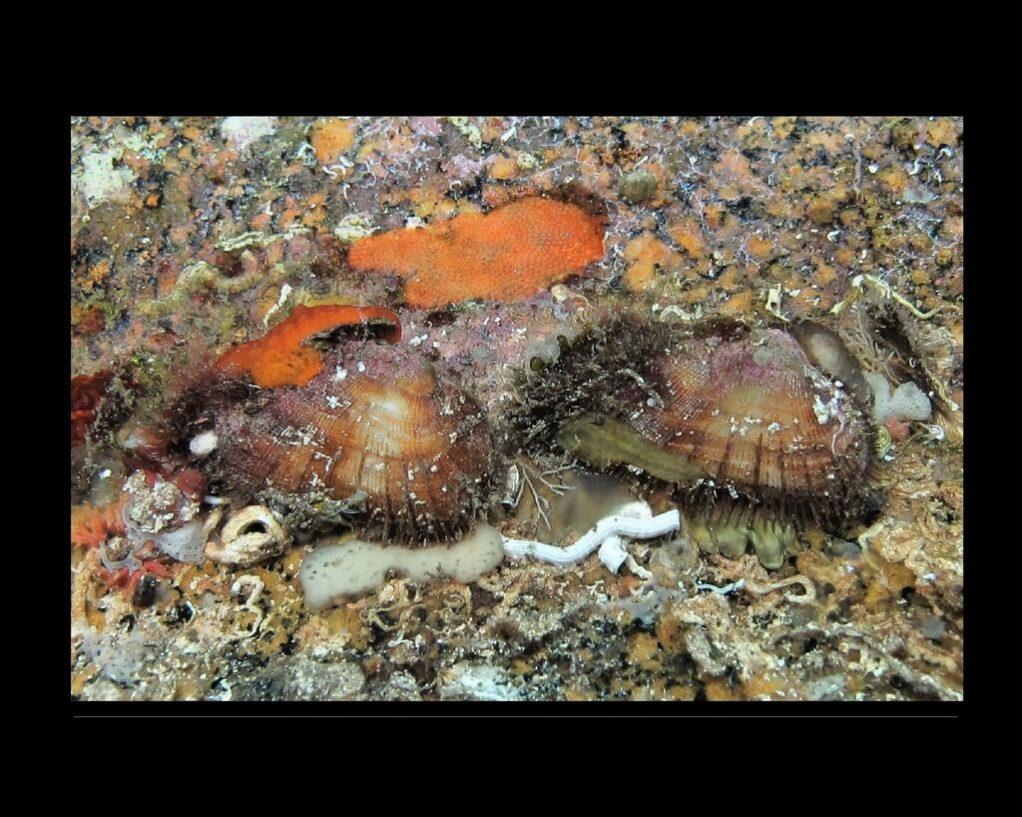
*Barbatiabarbata* (Linnaeus, 1758). *In situ* photograph.

**Figure 72.**
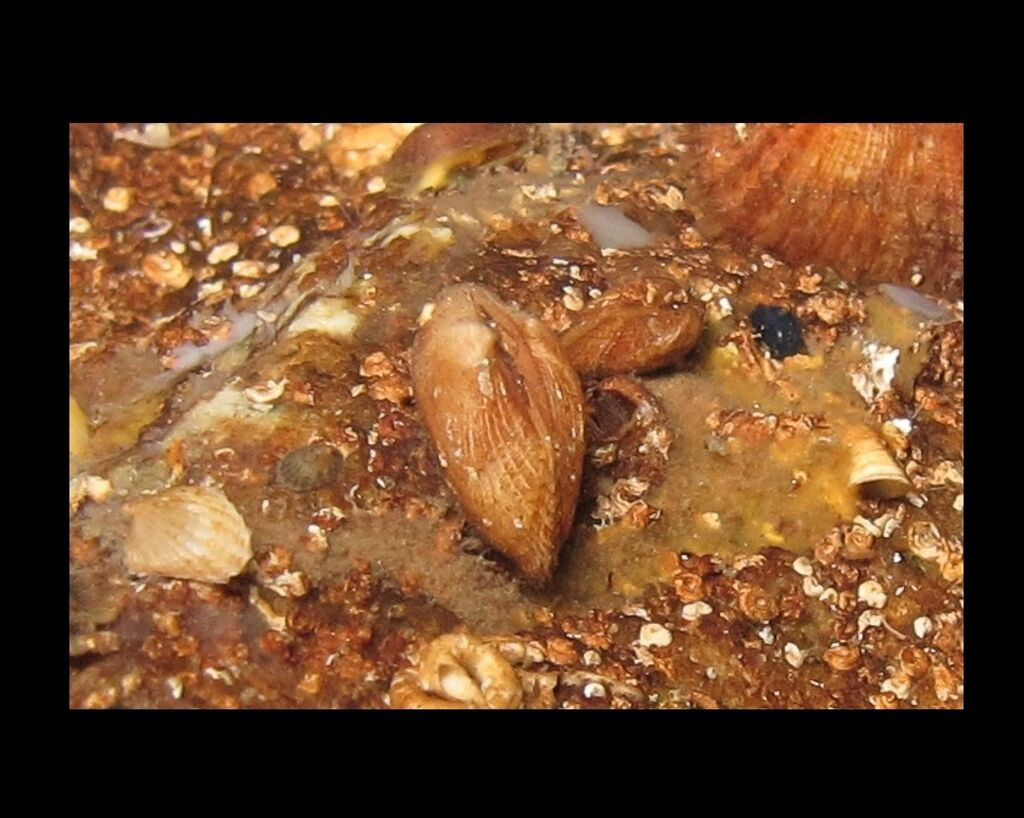
*Striarcalactea* (Linnaeus, 1758). *In situ* photograph.

**Figure 73.**
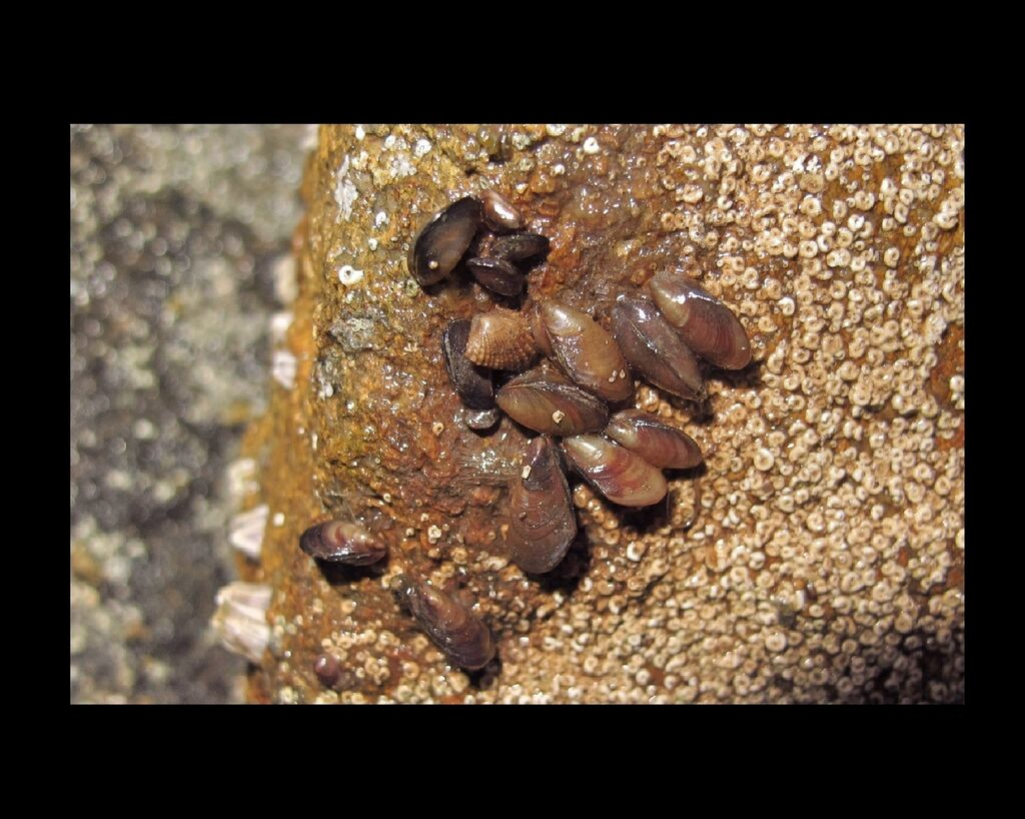
*Mytilastermarioni* (Locard, 1889). *In situ* photograph.

**Figure 74.**
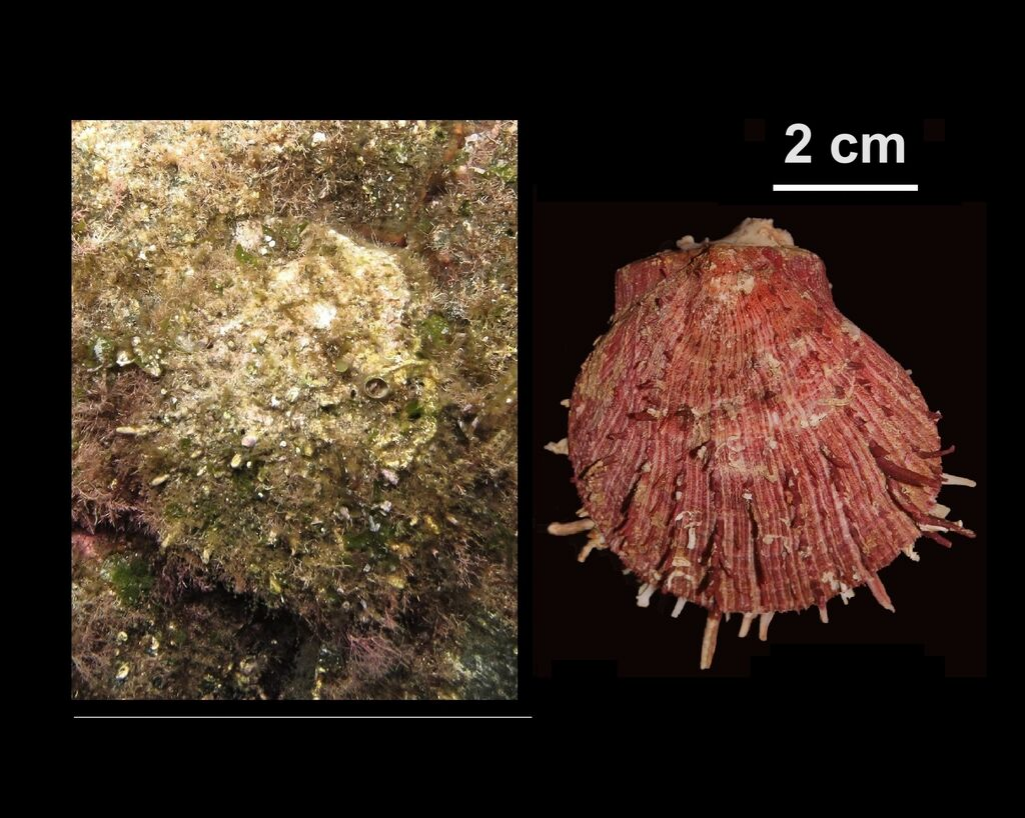
*Spondylusgaederopus* Linnaeus, 1758. On the left, *in situ* photograph; on the right, optical phototgraph.

**Figure 75.**
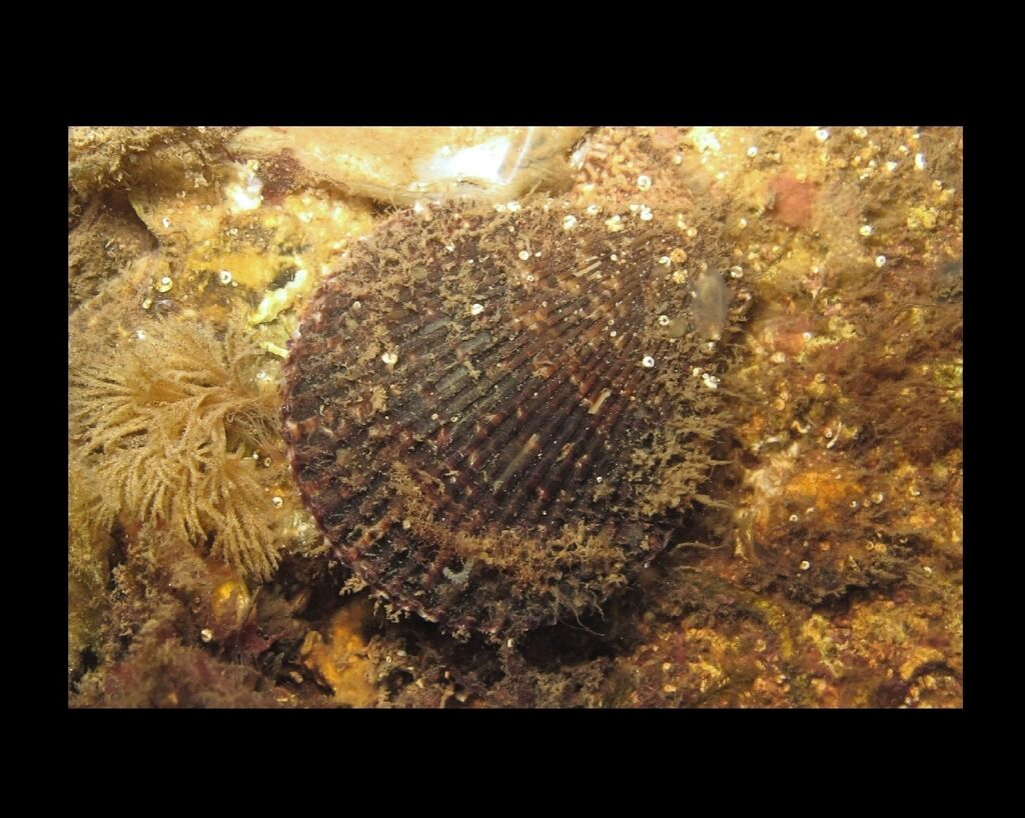
*Mimachlamysvaria* (Linnaeus, 1758). *In situ* photograph.

**Figure 76.**
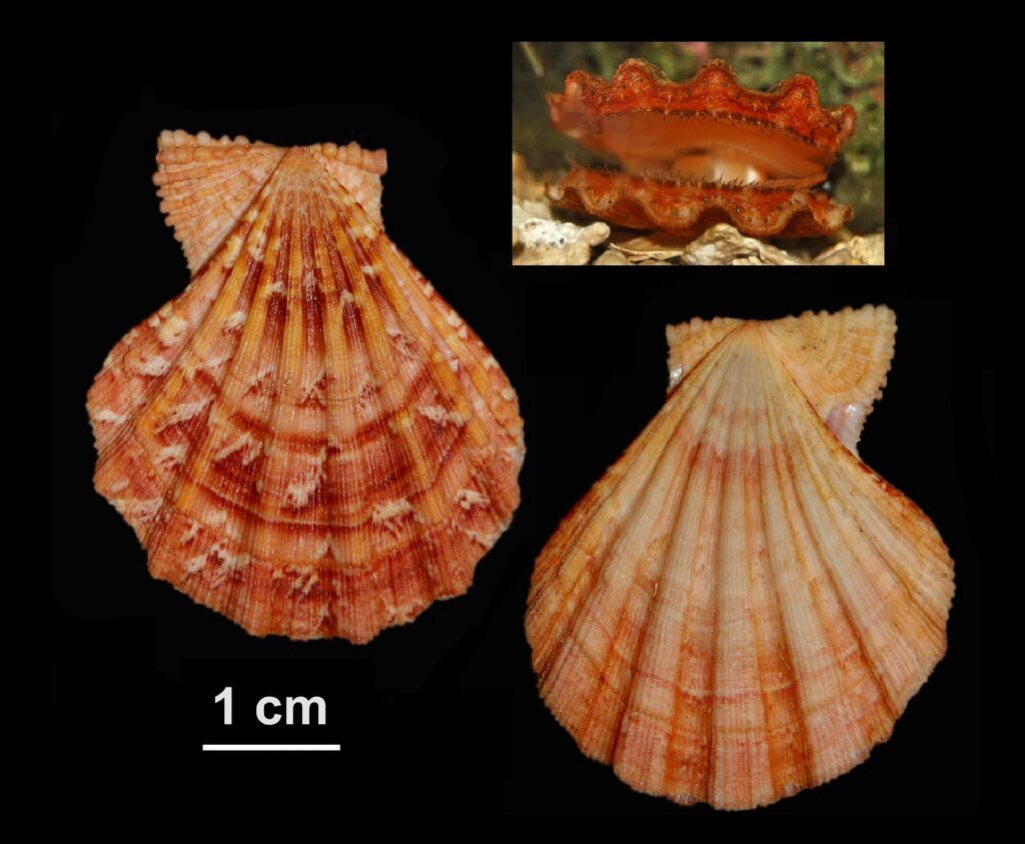
*Manupectenpesfelis* (Linnaeus, 1758). Lower, optical photograph; upper, *in situ* photograph.

**Figure 77.**
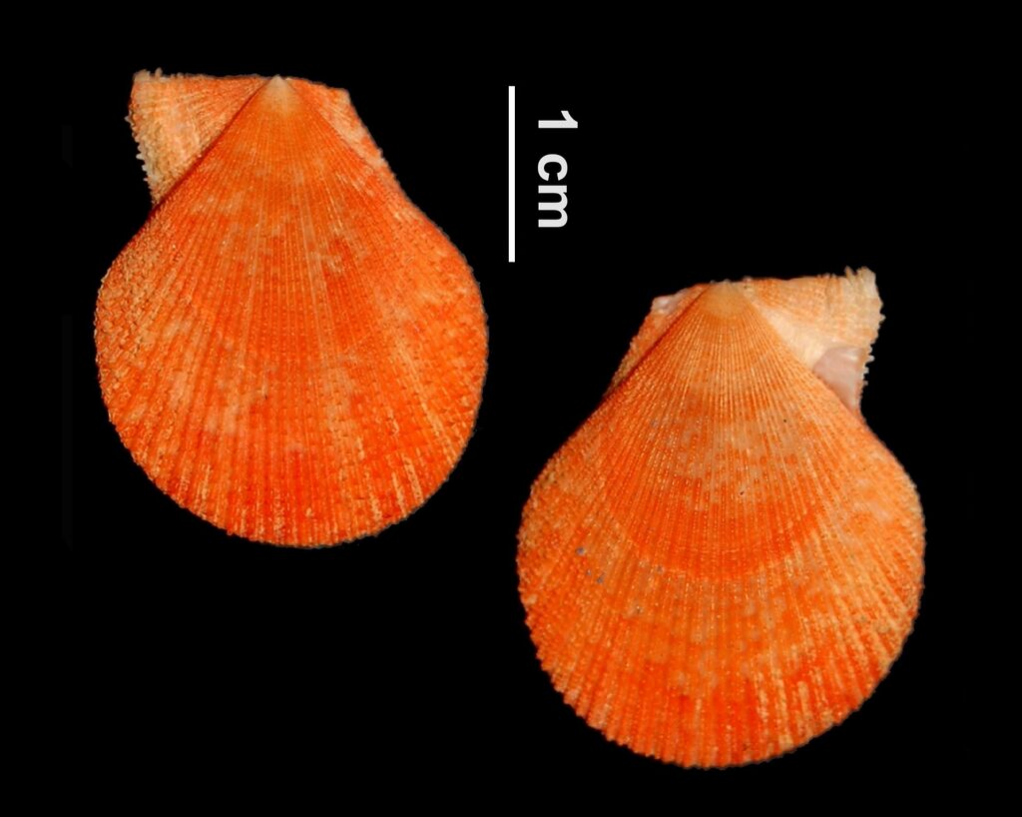
*Talochlamysmultistriata* (Poli, 1795). Optical photograph.

**Figure 78.**
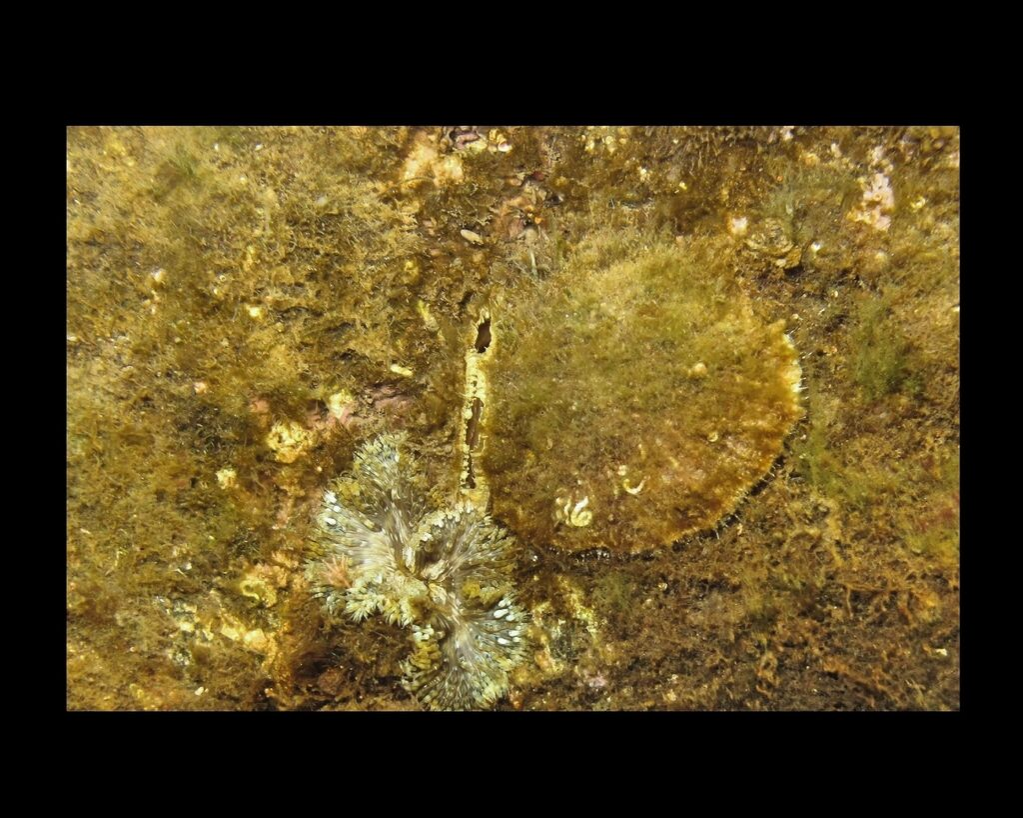
*Anomiaephippium* Linnaeus, 1758. *In situ* photograph.

**Figure 79.**
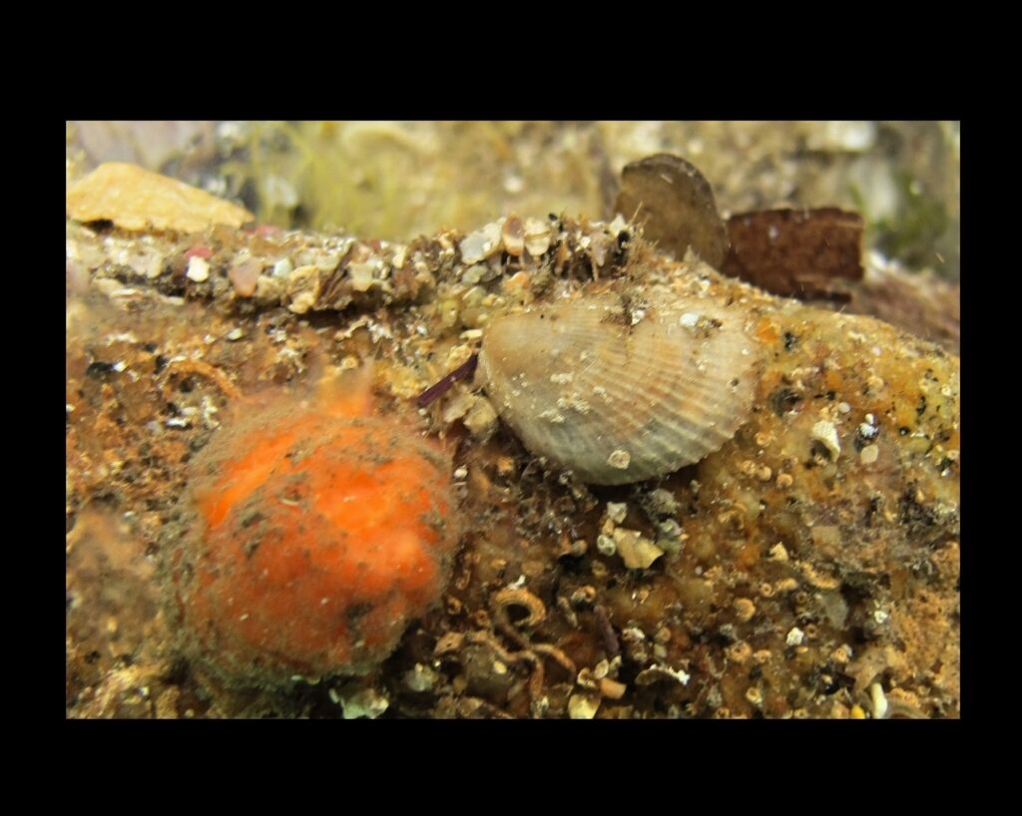
*Limalima* (Linnaeus, 1758). *In situ* photograph.

**Figure 82.**
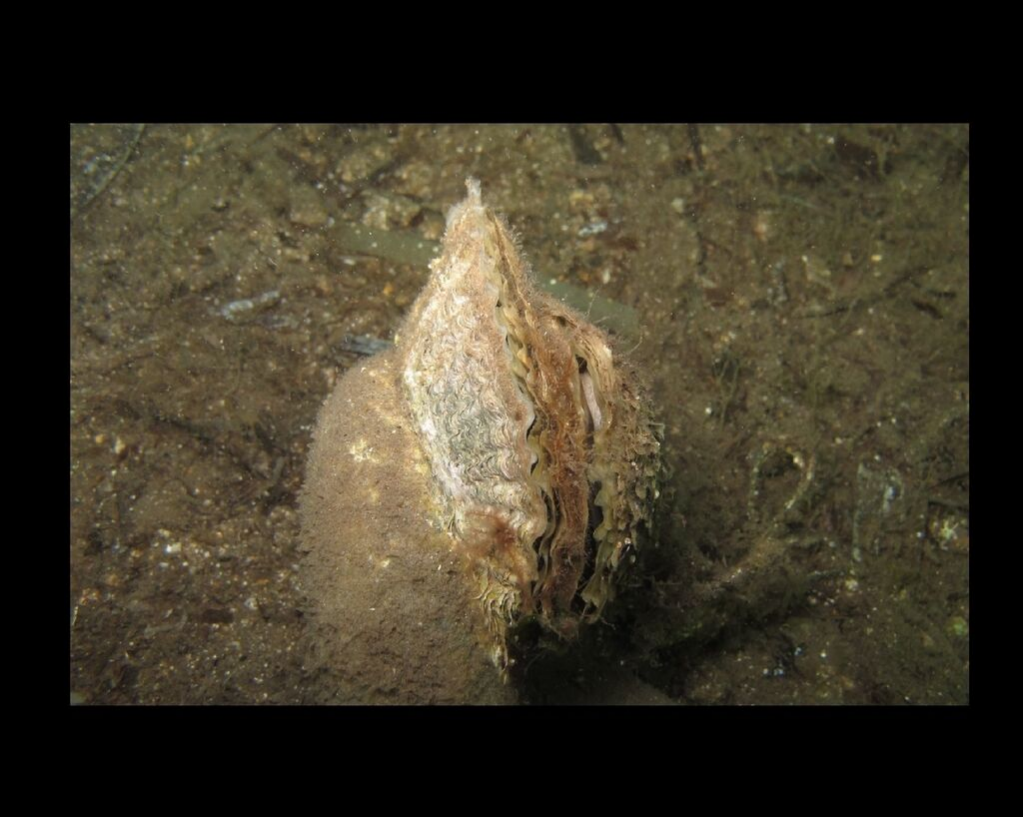
*Ostreaedulis* Linnaeus, 1758. *In situ* photograph.

**Figure 83.**
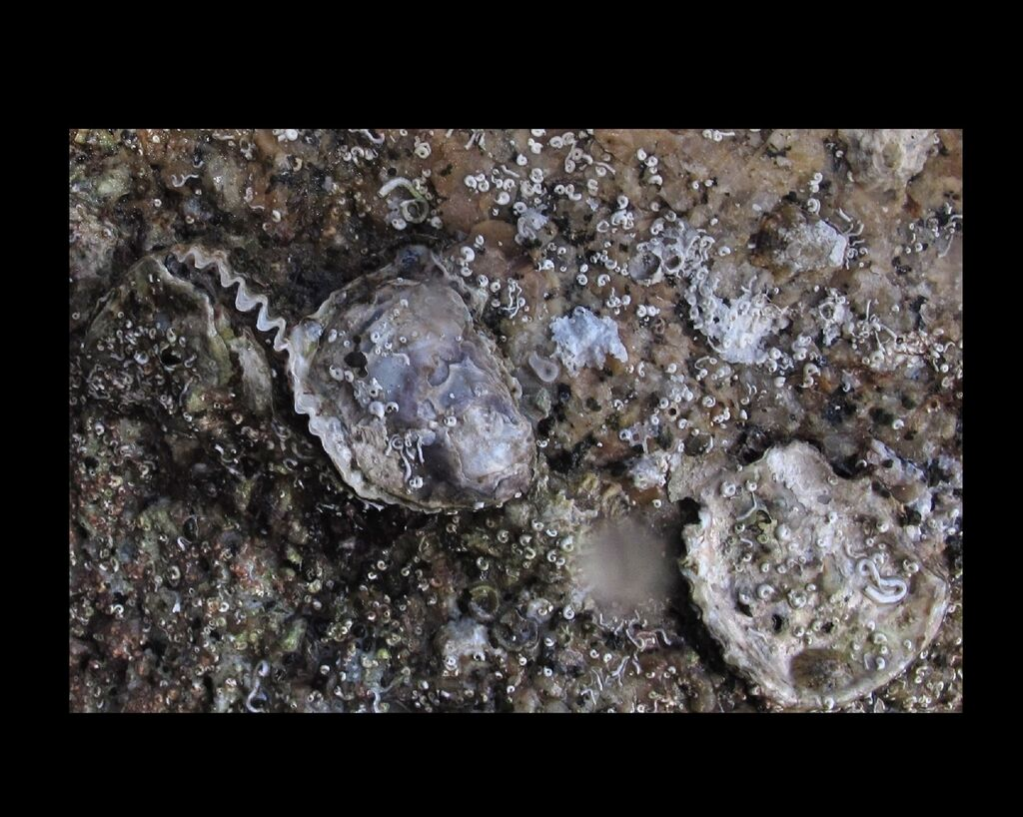
*Ostreastentina* Payraudeau, 1826. *In situ* photograph.

**Figure 85.**
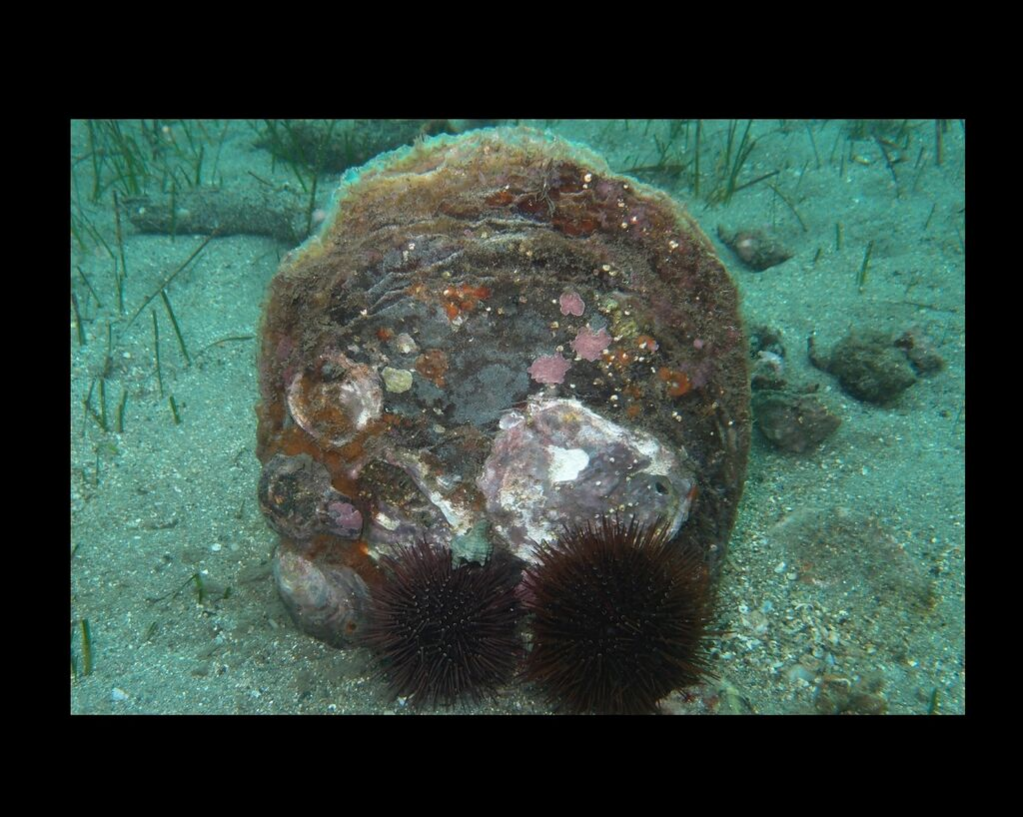
*Pinnanobilis* Linnaeus, 1758. Dead shell encrusted with epibionts.

**Figure 87.**
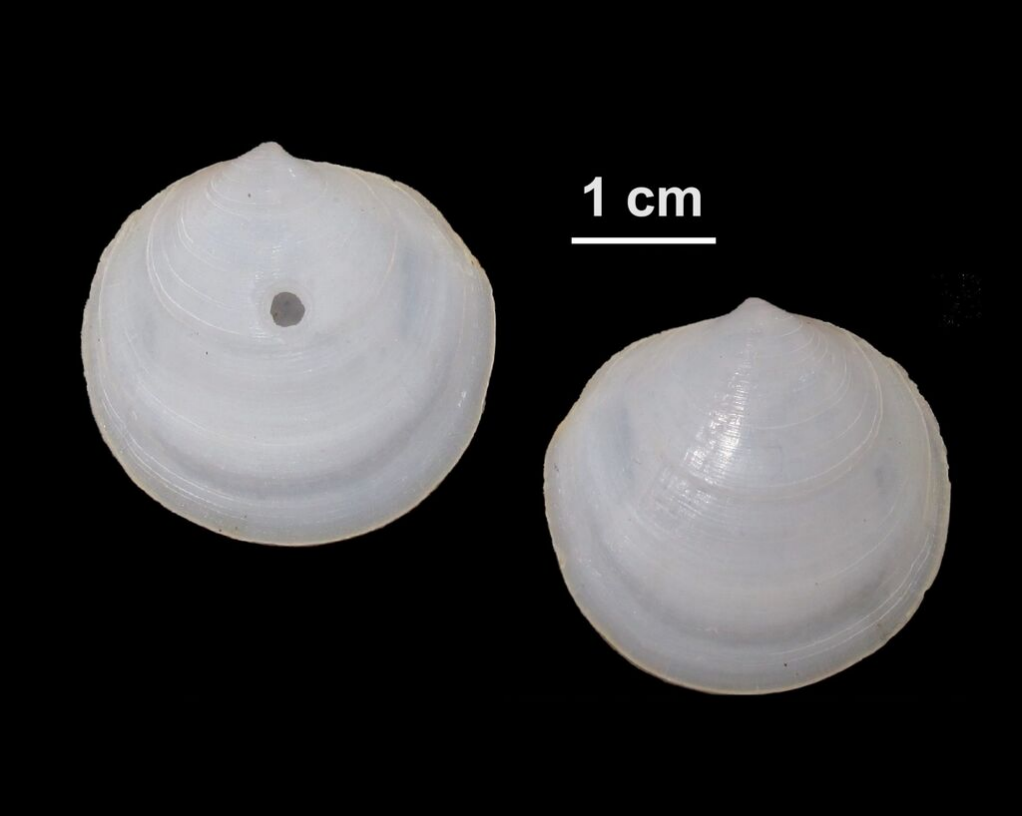
*Loripeslacteus* (Linnaeus, 1758). Preyed shell. Optical photograph.

**Figure 88.**
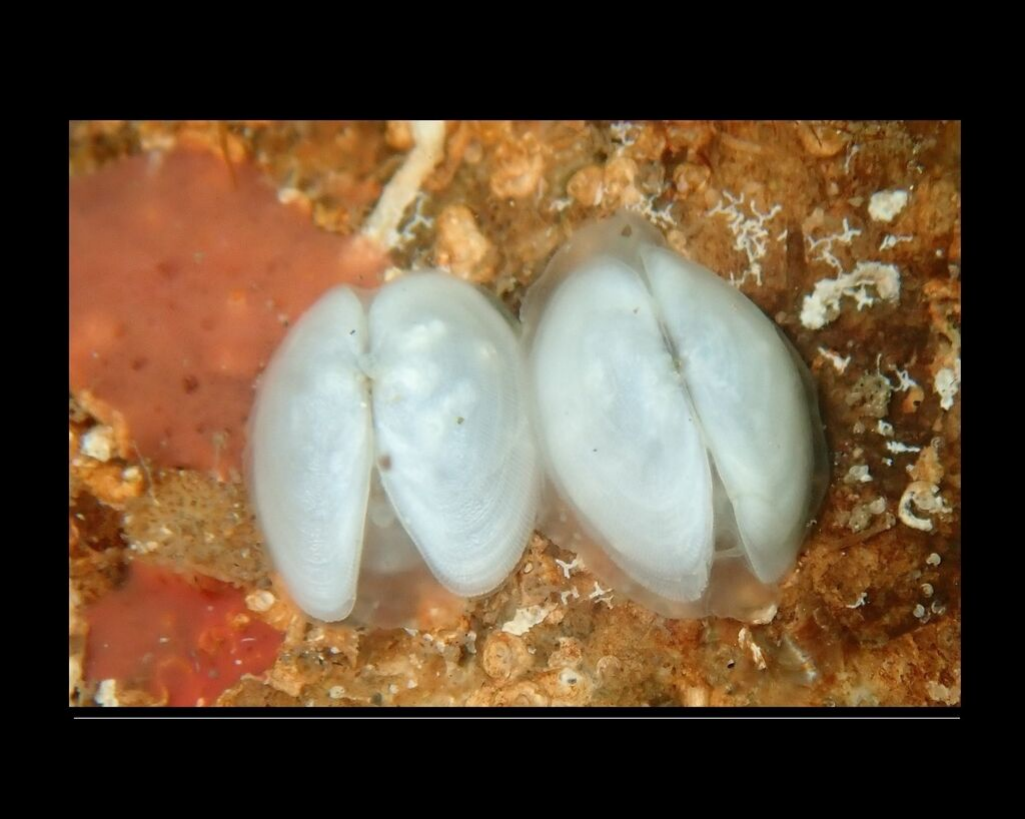
*Galeommaturtoni* W. Turton, 1825. *In situ* photograph.

**Figure 89.**
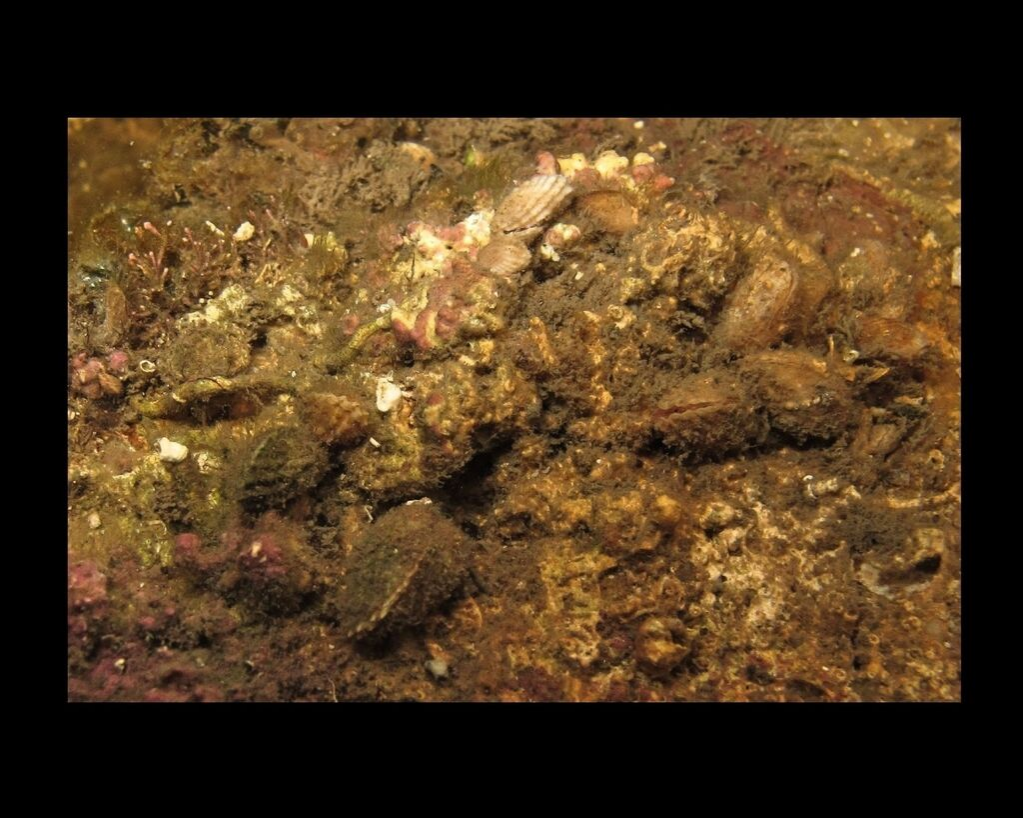
*Carditacalyculata* (Linnaeus, 1758). Optical photograph.

**Figure 91.**
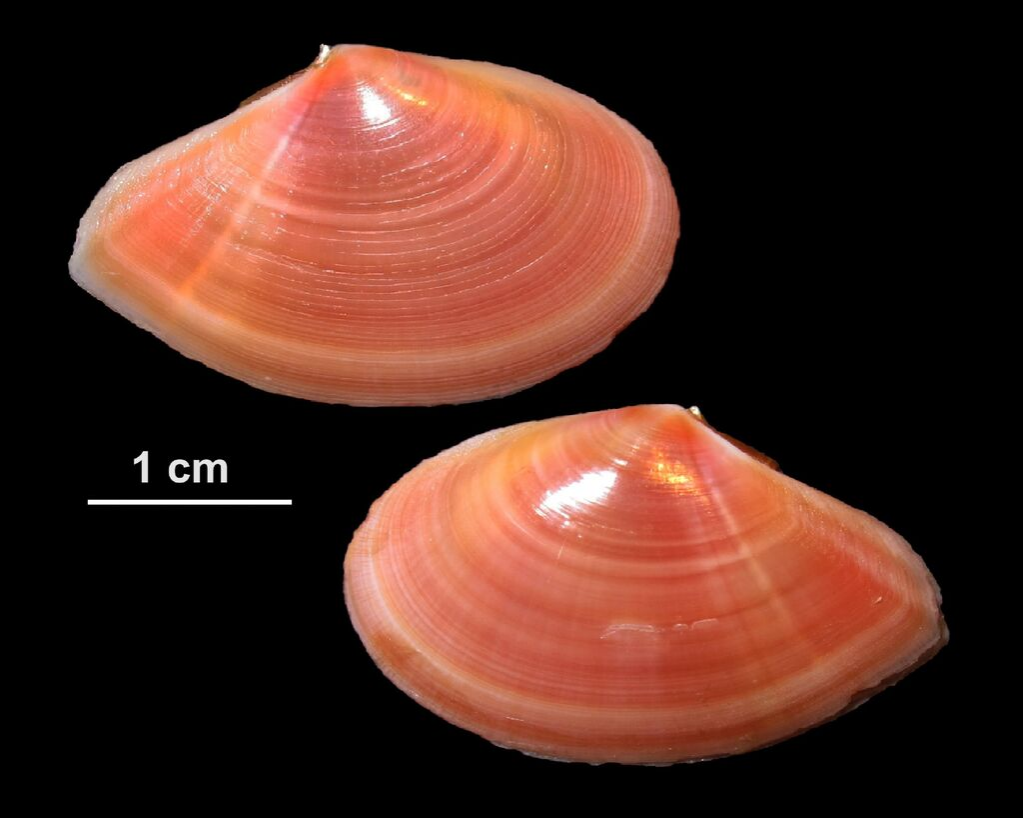
*Bosemprellaincarnata* (Linnaeus, 1758). Optical photograph.

**Figure 92.**
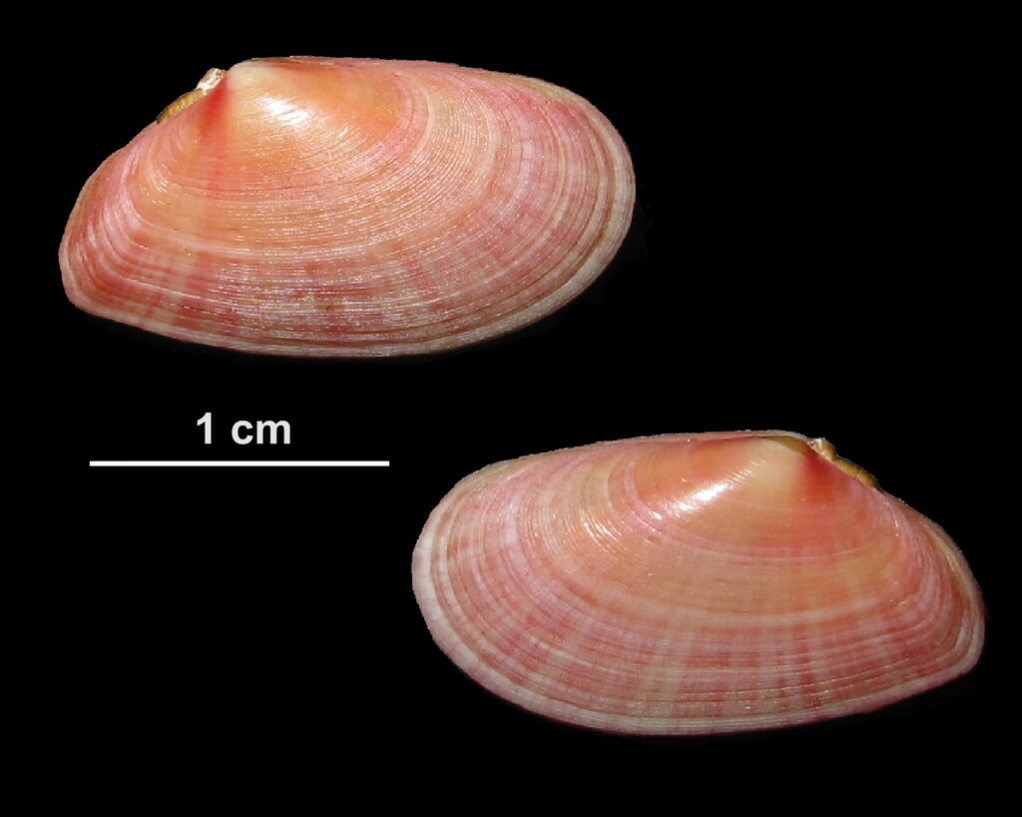
*Moerelladonacina* (Linnaues, 1758). Optical photograph.

**Figure 93.**
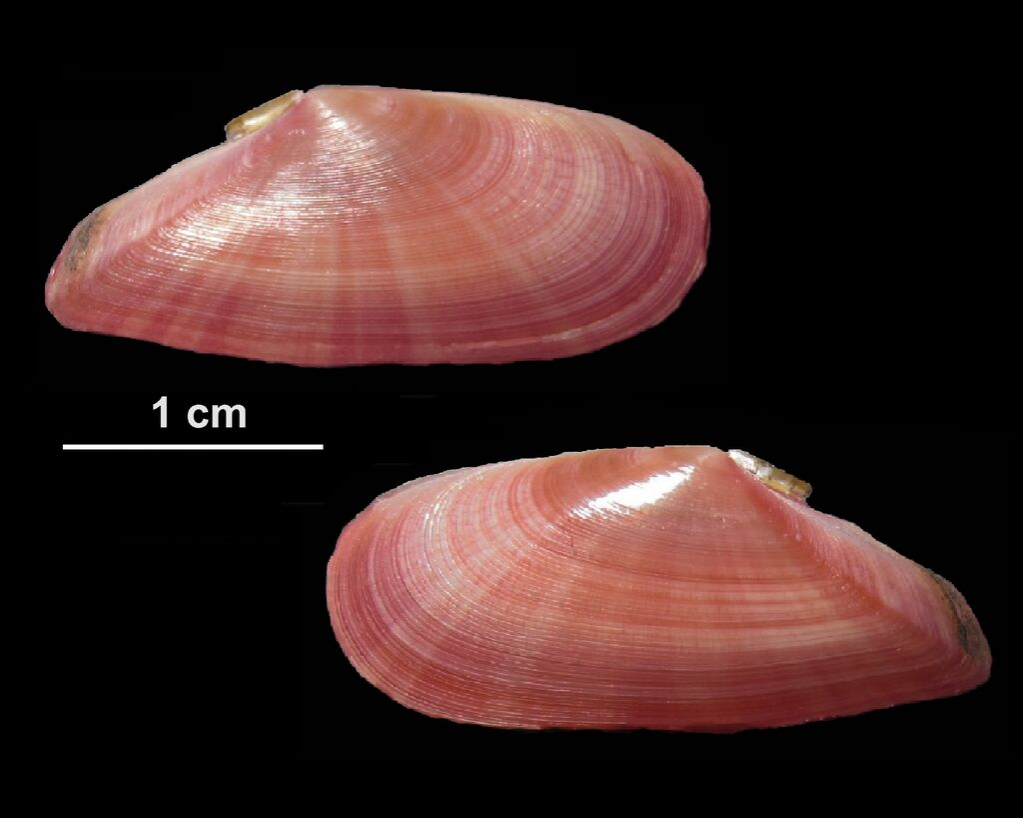
*Moerellapulchella* (Lamarck, 1818). Optical photograph.

**Figure 94.**
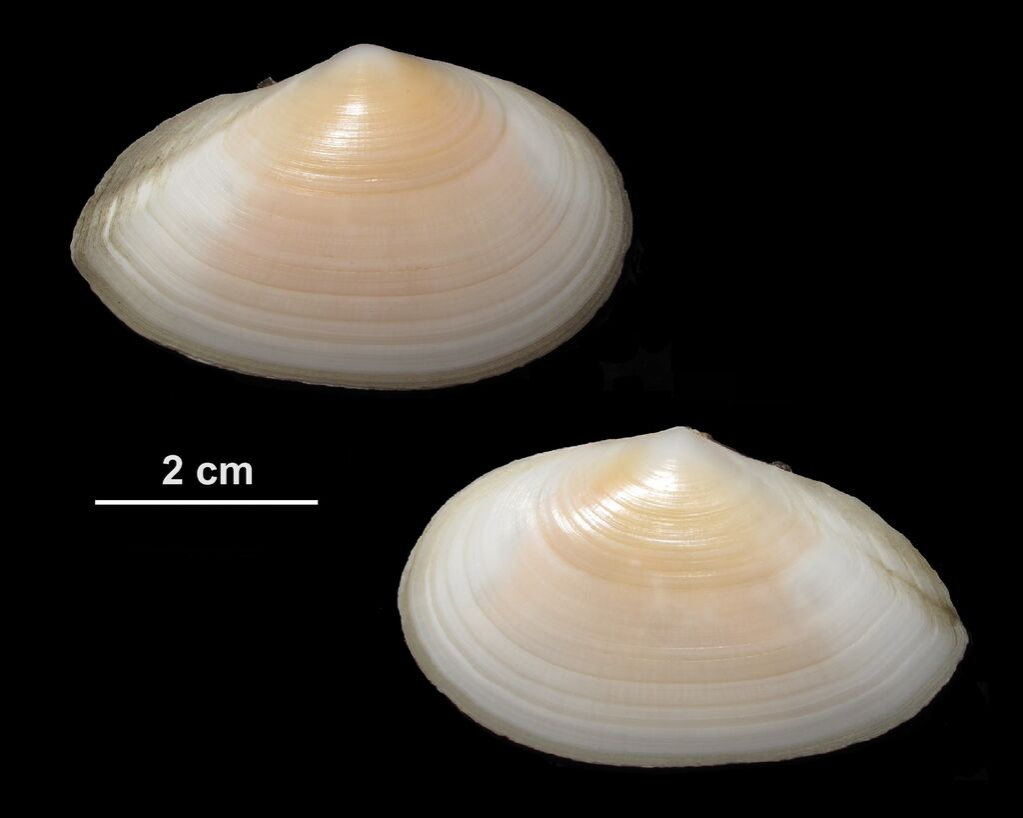
*Peronaeaplanata* (Linnaeus, 1758). Optical photograph.

**Figure 95.**
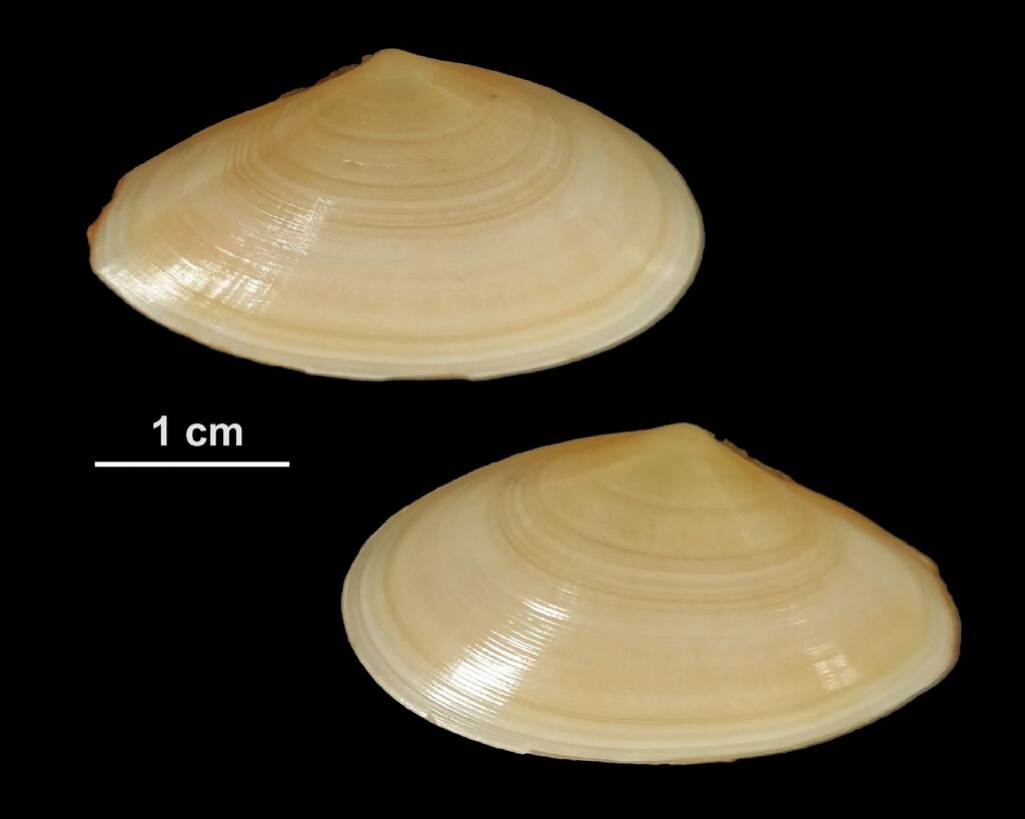
*Peronidiaalbicans* (Gmelin, 1791). Optical photograph.

**Figure 96.**
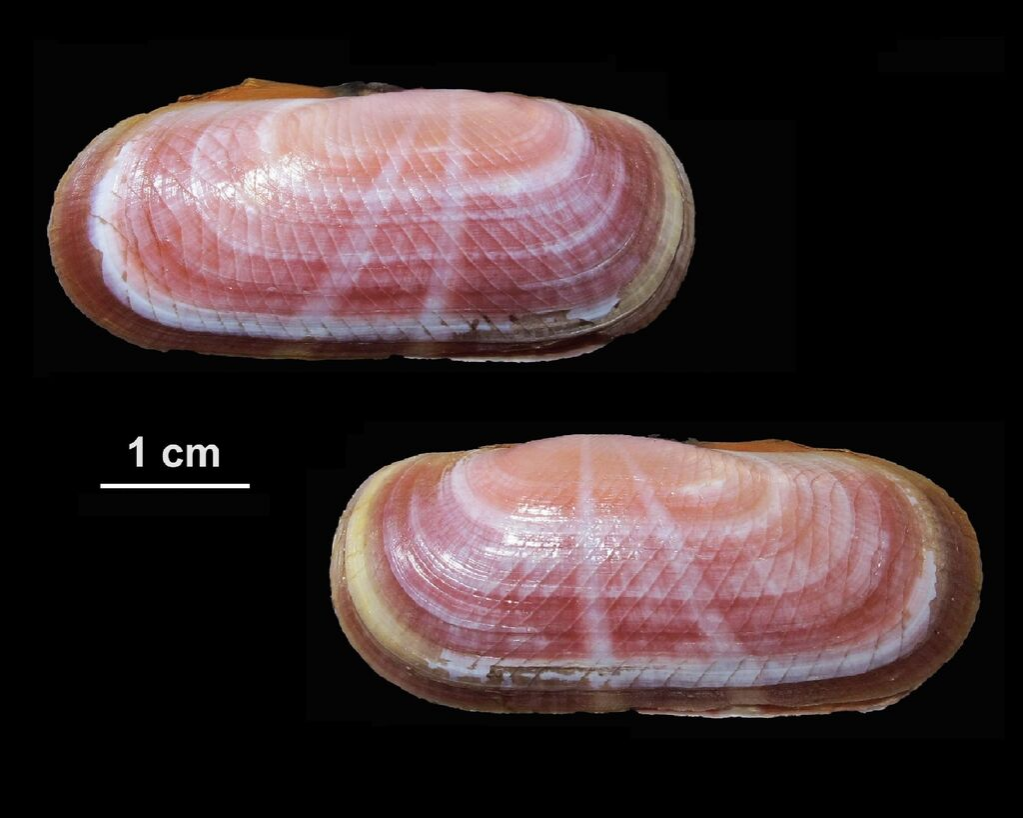
*Solecurtusstrigilatus* (Linnaeus, 1758). Optical photograph.

**Figure 97.**
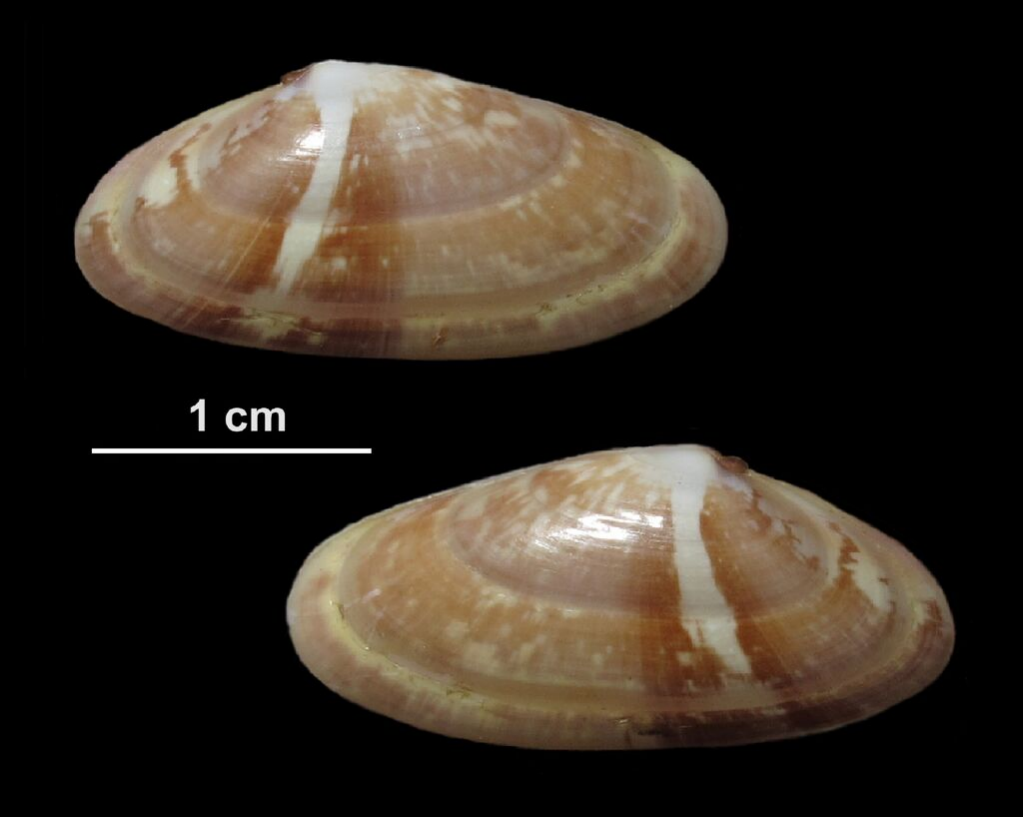
*Donaxvariegatus* (Gmelin, 1791). Optical photograph.

**Figure 98.**
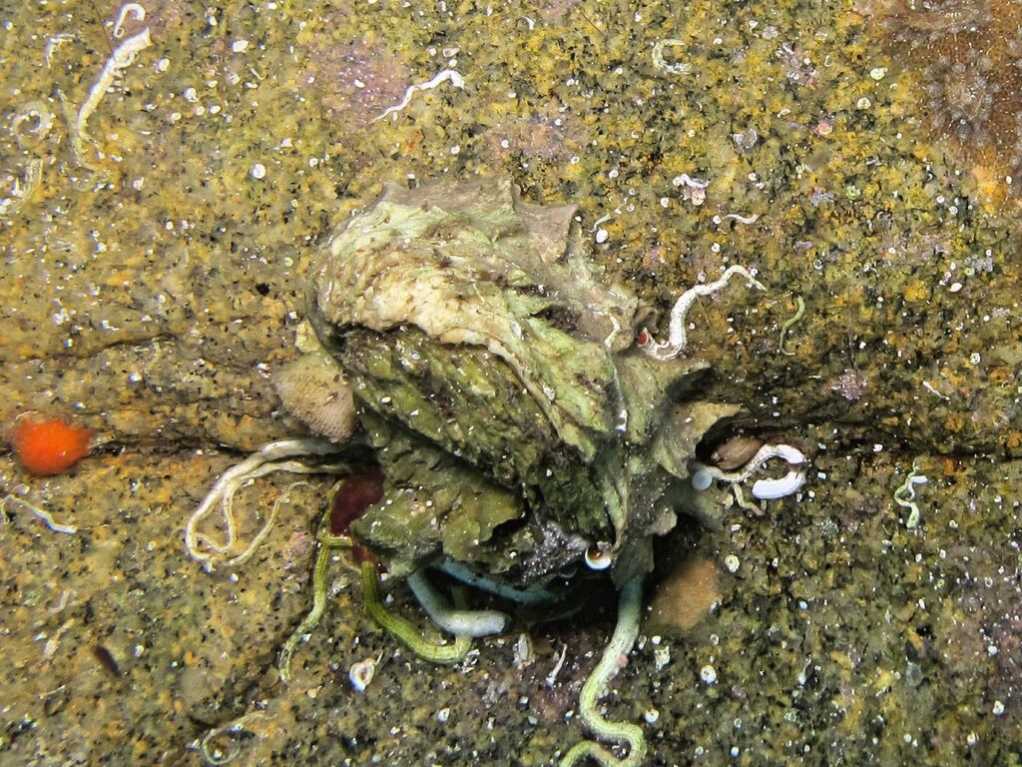
*Chamacircinata* Monterosato, 1878. *In situ* photograph.

**Figure 99.**
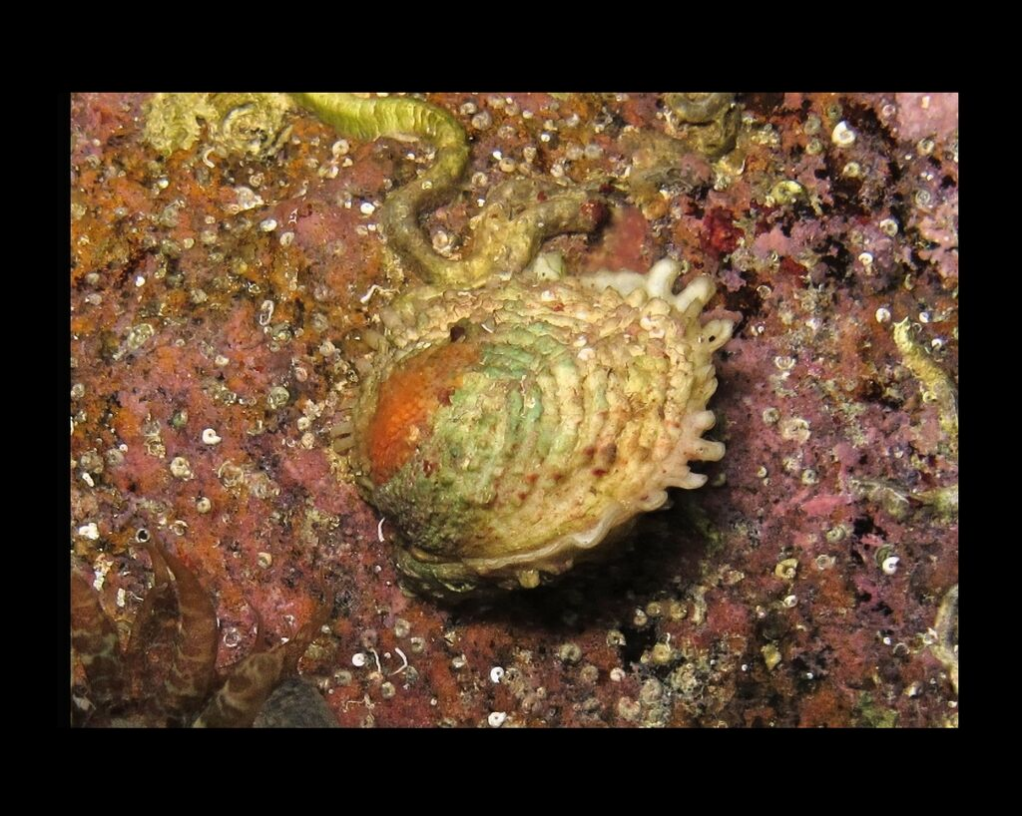
*Chamagryphoides* (Linnaeus, 1758) . *In situ* photograph.

**Figure 100.**
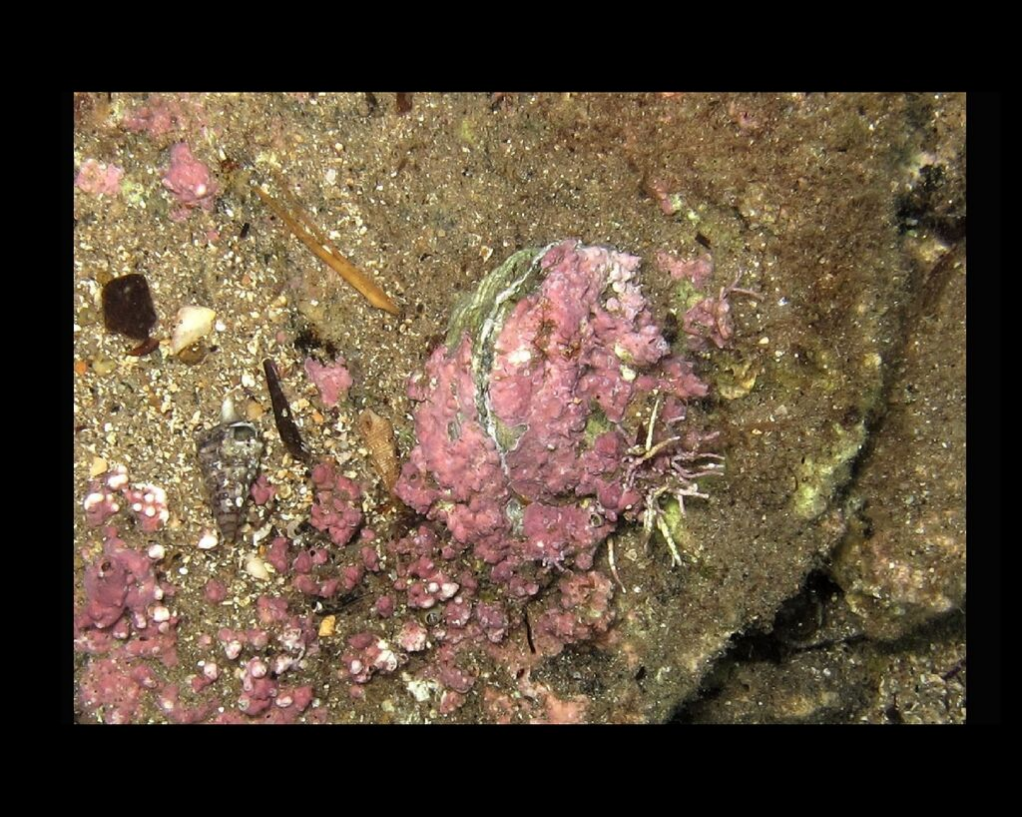
*Pseudochamagryphina* Lamarck, 1819). *In situ* photograph.

**Figure 101.**
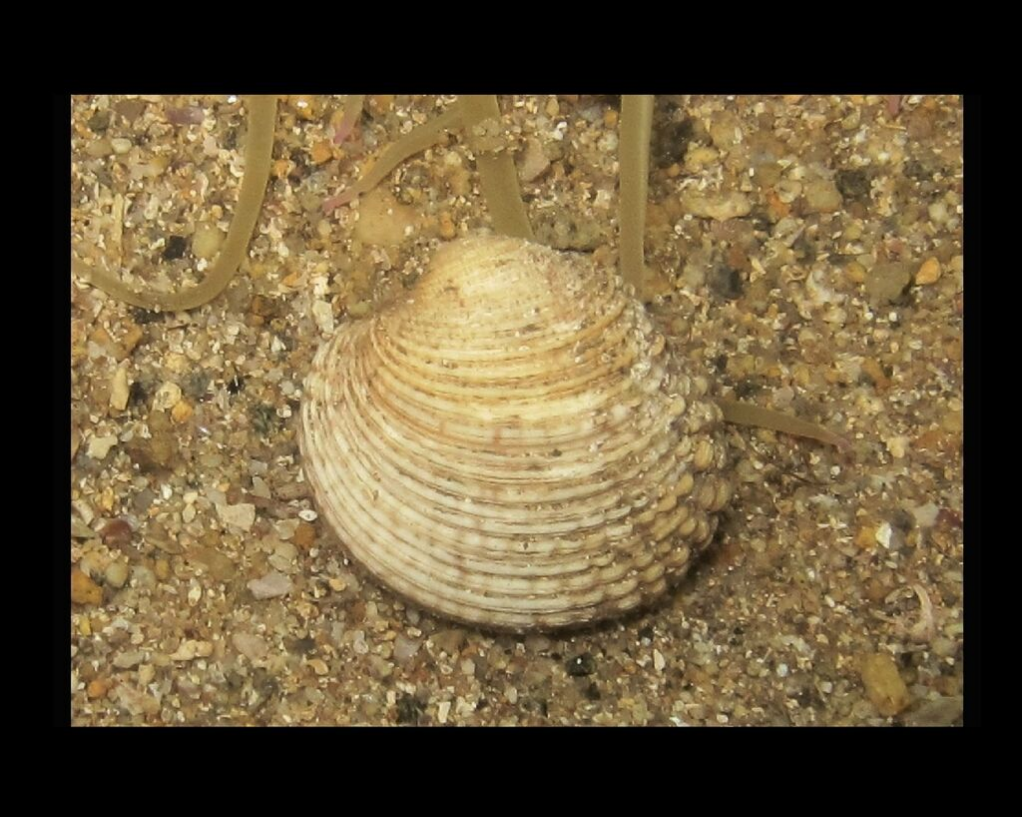
*Venusverrucosa* Linnaeus, 1758. *In situ* photograph.

**Figure 102.**
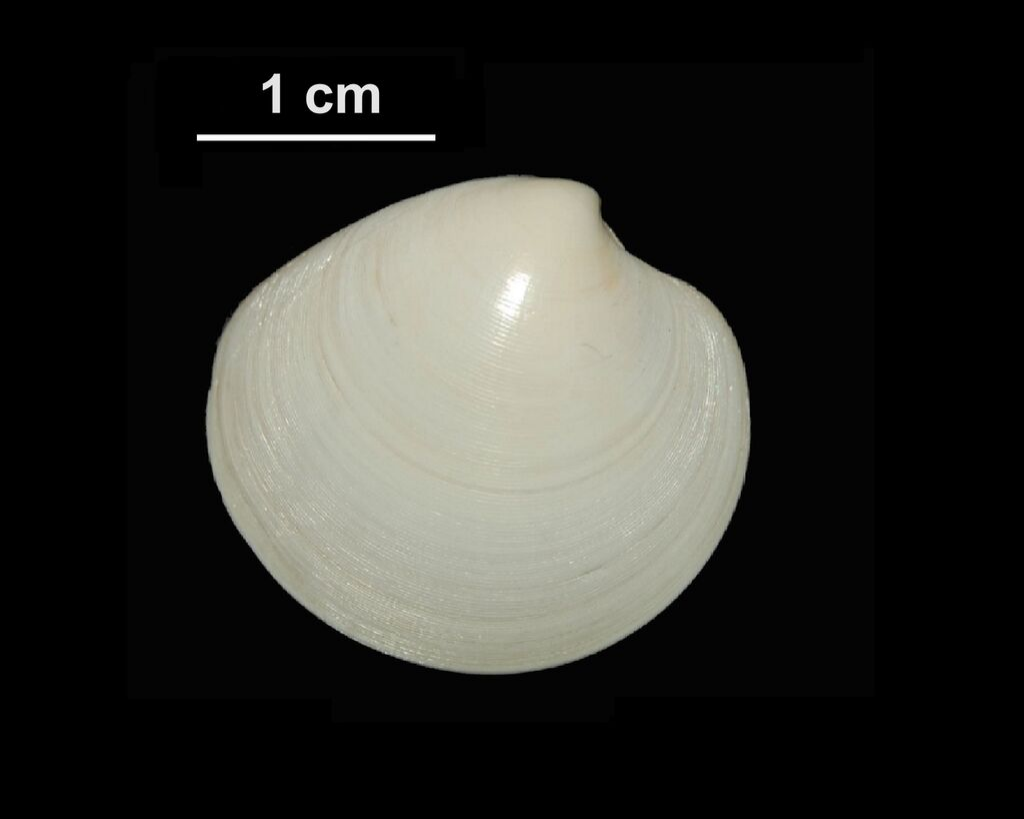
*Dosinialupinus* (Linnaeus, 1758). Optical photograph.

**Figure 103.**
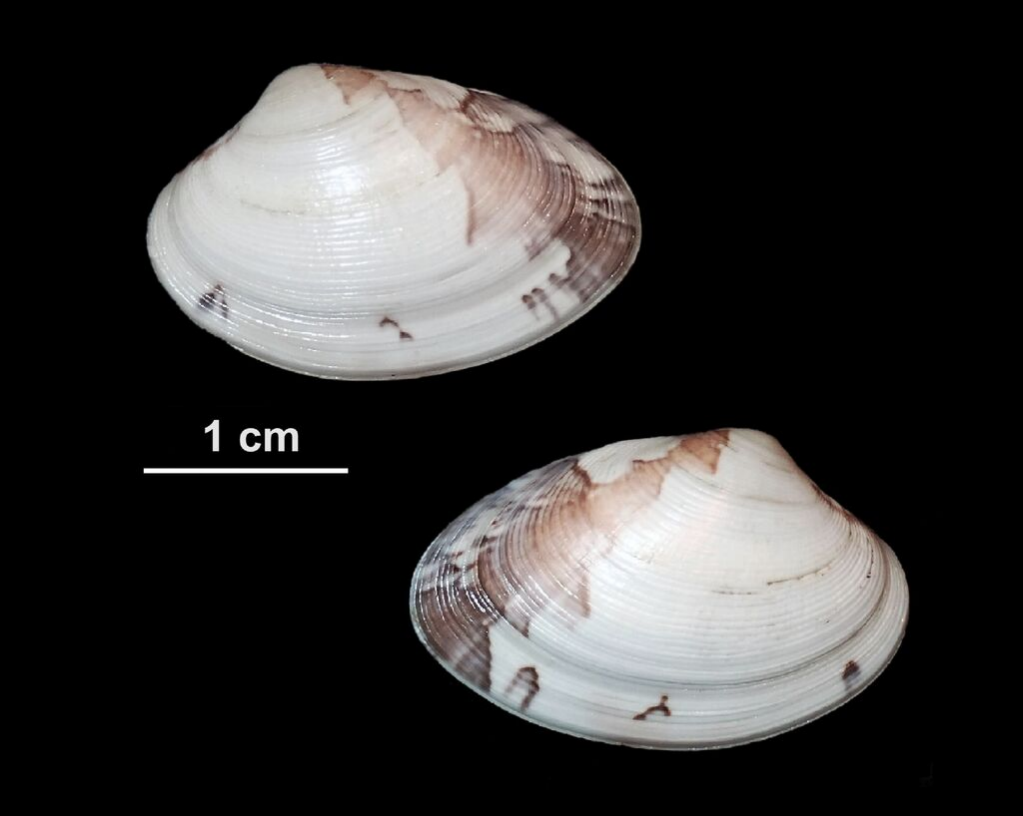
*Polititapesaureus* (Gmelin, 1791). Optical photograph.

**Table 1.** Number of the sampling locality (SS), geographic coordinates, depth range and habitat.

Sampling Site	Latitude - Longitude	Depth range (m)	Habitat
SS1	“41 11 46.79N 9 17 20.34E”	0-10	rocks-pebbles-gravel-sand-mud- *Posidonia* meadow
SS2	“41 11 56.04N 9 17 33.63E”	0-10	rocks-pebbles-gravel-sand- *Posidonia* meadow
SS3	“41 12 13.88N 9 17 56.14E”	0-15	rocks-pebbles-gravel-*Posidonia* meadow
SS4	“41 12 28.07N 9 17 47.62E”	0-15	rocks-pebbles-gravel-*Posidonia* meadow
SS5	“41 12 42.40N 9 17 45.35E”	0-15	rocks-pebbles-gravel-*Posidonia* meadow
SS6	“41 12 51.58N 9 17 31.46E”	0-10	rocks-pebbles-gravel-*Posidonia* meadow
SS7	“41 12 58.68N 9 17 26.51E”	0-15	rocks-pebbles-gravel-*Posidonia* meadow
SS8	“41 13 17.70N 9 17 21.41E”	0-20	rocks-pebbles-gravel-*Posidonia* meadow
SS9	“41 12 58.16N 9 17 09.66E”	0-10	rocks-pebbles-gravel-*Posidonia* meadow
SS10	“41 12 47.63N 9 17 08.68E”	0-10	rocks-pebbles-gravels-*Posidonia* meadow
SS11	“41 12 32.26N 9 16 52.46E”	0-10	rocks-pebbles-gravel-sand
SS12	“41 12 09.61N 9 16 43.24E”	0-5	rocks-pebbles-gravel-sand-mud
SS13	“41 11 46.14N 9 16 40.60E”	0-5	rocks-pebbles-gravel-sand-mud
SS14	“41 11 28.32N 9 16 44.50E”	0-5	rocks-pebbles-gravel-sand-mud
SS15	“41 11 22.14N 9 16 59.05E”	0-5	rocks-pebbles-gravel-sand-mud
